# The Globin Gene Family in Arthropods: Evolution and Functional Diversity

**DOI:** 10.3389/fgene.2020.00858

**Published:** 2020-08-13

**Authors:** Andreas Prothmann, Federico G. Hoffmann, Juan C. Opazo, Peter Herbener, Jay F. Storz, Thorsten Burmester, Thomas Hankeln

**Affiliations:** ^1^Institute of Organismic and Molecular Evolution, Molecular Genetics and Genome Analysis, University of Mainz, Mainz, Germany; ^2^Department of Biochemistry, Molecular Biology, Entomology and Plant Pathology, Mississippi State University, Mississippi, MS, United States; ^3^Institute for Genomics, Biocomputing and Biotechnology, Mississippi State University, Mississippi, MS, United States; ^4^Instituto de Ciencias Ambientales y Evolutivas, Facultad de Ciencias, Universidad Austral de Chile, Valdivia, Chile; ^5^Millennium Nucleus of Ion Channels-Associated Diseases (MiNICAD), Valdivia, Chile; ^6^School of Biological Sciences, University of Nebraska−Lincoln, Lincoln, NE, United States; ^7^Institute of Zoology, University of Hamburg, Hamburg, Germany

**Keywords:** hemoglobin, hypoxia, hexapoda, respiration, intron

## Abstract

Globins are small heme-proteins that reversibly bind oxygen. Their most prominent roles in vertebrates are the transport and storage of O_2_ for oxidative energy metabolism, but recent research has suggested alternative, non-respiratory globin functions. In the species-rich and ecologically highly diverse taxon of arthropods, the copper-containing hemocyanin is considered the main respiratory protein. However, recent studies have suggested the presence of globin genes and their proteins in arthropod taxa, including model species like Drosophila. To systematically assess the taxonomic distribution, evolution and diversity of globins in arthropods, we systematically searched transcriptome and genome sequence data and found a conserved, widespread occurrence of three globin classes in arthropods: hemoglobin-like (HbL), globin X (GbX), and globin X-like (GbXL) protein lineages. These globin types were previously identified in protostome and deuterostome animals including vertebrates, suggesting their early ancestry in Metazoa. The *HbL* genes show multiple, lineage-specific gene duplications in all major arthropod clades. Some *HbL* genes (e.g., *Glob2* and *3* of *Drosophila*) display particularly fast substitution rates, possibly indicating the evolution of novel functions, e.g., in spermatogenesis. In contrast, arthropod *GbX* and *GbXL* globin genes show high evolutionary stability: GbXL is represented by a single-copy gene in all arthropod groups except Brachycera, and representatives of the GbX clade are present in all examined taxa except holometabolan insects. GbX and GbXL both show a brain-specific expression. Most arthropod GbX and GbXL proteins, but also some HbL variants, include sequence motifs indicative of potential N-terminal acylation (i.e., N-myristoylation, 3C-palmitoylation). All arthropods except for the brachyceran Diptera harbor at least one such potentially acylated globin copy, confirming the hypothesis of an essential, conserved globin function associated with the cell membrane. In contrast to other animals, the fourth ancient globin lineage, represented by neuroglobin, appears to be absent in arthropods, and the putative arthropod orthologs of the fifth metazoan globin lineage, androglobin, lack a recognizable globin domain. Thus, the remarkable evolutionary stability of some globin variants is contrasted by occasional dynamic gene multiplication or even loss of otherwise strongly conserved globin lineages in arthropod phylogeny.

## Introduction

Globins are small respiratory proteins that reversibly bind O_2_ and other gaseous ligands by means of a prosthetic heme group. The globin core typically comprises ∼140 amino acids and is built by eight alpha-helices (named A to H), which form a characteristic 3-over-3 alpha-helical sandwich structure (Perutz, 1979; Dickerson and Geis, 1983; Bolognesi et al., 1997). While many members of the globin protein family have a role in O_2_ supply, as classically exemplified by vertebrate hemoglobin (Hb) and myoglobin (Mb), they may also carry out a variety of alternative metabolic functions, such as O_2_ sensing and the detoxification of reactive nitrogen or oxygen species, or they may even be components of intracellular signaling pathways (Weber and Vinogradov, 2001; Hankeln et al., 2005). Globins are evolutionarily ancient proteins and have been identified in a broad range of animals, plants, fungi and bacteria (Hardison, 1996; Weber and Vinogradov, 2001). Phylogenetic analyses have indicated an early divergence of globin lineages in the Metazoa (Roesner et al., 2005; Vinogradov et al., 2005; Blank and Burmester, 2012). Four major ancient globin clades were identified in animals, representing (i) neuroglobin (Ngb) and related proteins, (ii) globin X (GbX), (iii) globin X-like (GbXL) proteins, which both are characterized by N-terminal acylation sites possibly mediating attachment to the cell membrane (Blank et al., 2011; Blank and Burmester, 2012), and (iv) hemoglobin-like globins (HbL), which include the “typical” hemoglobins (Hb), myoglobins (Mb) and their relatives of vertebrates, other deuterostomes and protostomes (Blank and Burmester, 2012). A fifth ancient globin lineage is androglobin (Adgb), a large chimeric protein with an internal globin domain, which is conserved from basal metazoans to humans (Hoogewijs et al., 2012). The unexpected diversity of the metazoan globin family points at important, yet ill-defined physiological roles of this ancient protein class.

The species-rich taxon of arthropods is widely divergent in terms of organismal size, morphology and inhabited ecological niches, thus requiring specific adaptations in oxidative metabolism. Arthropods typically possess copper-containing respiratory proteins in their hemolymph, referred to as hemocyanins, which have been identified in all arthropod subphyla (Burmester, 2002; Burmester and Hankeln, 2007). Thus, there is little doubt that hemocyanins that emerged in the Cambrian or even earlier represent the ancestral respiratory proteins of arthropods (Rehm et al., 2012). Hemocyanins have recently been identified in several “lower” insect taxa, but have been lost in Eumetabola. Certain arthropod taxa lacking hemocyanins (e.g., the branchiopod crustaceans *Daphnia* sp. and *Triops* sp.) have replaced it by expression of Hb (Weber and Vinogradov, 2001). In most hexapods, however, specialized respiratory proteins have long been considered unnecessary, because the tracheal system very efficiently delivers O_2_ to the inner organs. Only in a few exceptional insect taxa that dwell in hypoxic environments, Hb is present at high concentrations and functions as a respiratory protein. For example, in chironomid midges (Nematocera) extracellular Hb variants are used to transport and store O_2_ in the hemolymph to ensure survival of the aquatic larvae under hypoxic conditions (Weber et al., 1985). Larvae of the parasitic botfly *Gasterophilus intestinalis* harbor large quantities of intracellular Hbs, which serves as an O_2_ store in the hypoxic environment of the host’s body (Keilin and Wang, 1946; Dewilde et al., 1998). Additionally, some aquatic backswimmers (Notonectidae) use intracellular Hb to control buoyancy during diving (Matthews and Seymour, 2006; Wawrowski et al., 2012).

While these examples were perceived as unusual exceptions for many years, more recent studies revealed the presence of globin genes in the genomes of a diverse set of insect taxa, including flies, mosquitoes, bees, beetles and lepidopterans (Burmester and Hankeln, 1999, 2007; Hankeln et al., 2002, 2006; Burmester et al., 2006, 2007). These globins are probably expressed at lower concentrations than in the examples cited above and thus do not produce a characteristic red color of the animal tissues. The functions of these insect globins are not yet well established, although there is evidence that at least some of them are involved in oxidative metabolism (Hankeln et al., 2002). For example, *Drosophila melanogaster* Glob1 shows the typical globin fold and displays conservation of the residues important for O_2_ binding (de Sanctis et al., 2005). Deoxygenated Glob1, which has a hexacoordinate binding scheme at the Fe^2+^ ion, binds O_2_ with an affinity similar to that of other respiratory globins in insects. In larval and adult *Drosophila*, Glob1 is mainly expressed in tracheal cells and the fat body (Hankeln et al., 2002). Data from knockdown experiments indicate that Glob1 may play a role in O_2_ metabolism of insects (Gleixner et al., 2016), although a different role for Glob1 in maintenance of the cytoskeleton was recently proposed (Yadav and Sarkar, 2016; Yadav et al., 2018). *Drosophila* has two additional globins, Glob2 and 3, which are structurally derived and specifically expressed in the testis (Burmester et al., 2006; Gleixner et al., 2012). It is unlikely that they play a role in O_2_ supply. Globin expression was also identified in visceral muscles of mosquitoes (*Anopheles gambiae*) (Burmester et al., 2007), and in the tracheal system and Malpighian tubules of the honeybee (*Apis mellifera*) (Hankeln et al., 2006), suggesting involvement in metabolic, possibly respiratory, processes.

Despite such previous work, there is still much to learn about the presence of globins in arthropods and their role in arthropod physiology. To understand the evolution of arthropod globins as an essential prerequisite to unravel their functions, we conducted a systematic survey of the globin gene repertoire of arthropods, with particular emphasis on insects, by making use of the wealth of available genomic and transcriptomic data. In particular, we assessed the phylogenetic diversity of arthropod globins in the context of the well-documented patterns in other metazoans. In addition, we applied structural prediction and gene expression analyses of globin genes to pave the way for understanding globin functions in arthropods.

## Materials and Methods

### Database Searches and Compilation of Globin Sequences

We performed systematic BLAST searches (Altschul et al., 1997) using the amino acid sequences of a representative set of already known arthropod globins plus vertebrate globins as initial query dataset. This dataset was systematically complemented with the sequences found during the initial search phases. The TBLASTN algorithm (with BLOSUM62 and 45 matrices) was used to screen the nucleotide, EST and TSA or whole genome sequences of arthropods available at GenBank^1^. In addition, transcriptomic data of ostracoda (Oakley et al., 2013), accessible via the Dryad Digital Repository^2^, were screened. Candidate globin sequences (e-value threshold < 1) were routinely subjected to a “reverse BLAST” search on the protein level to confirm their globin identity. Incomplete or misannotated sequences were – if possible – manually corrected on the basis of the nucleotide sequences. Incomplete globin sequences were counted as a hit in defining the globin repertoire, but were not considered in phylogenetic reconstructions. The full list of sequences used is given in Supplementary Tables S1, S2.

In the special case of the chimeric Adgb, the newly detected arthropod Adgb sequences were aligned by Clustal with the known Adgbs to identify the position of their putative globin domain. Candidate globin domain parts were extracted from the larger Adgb sequence and rearranged to represent the standard order of globin alpha-helices (A to H) before using CDD^3^ (Marchler-Bauer et al., 2015) and FUGUE^4^ (Shi et al., 2001) to confirm their globin domain identity.

### Multiple Sequence Alignments and Phylogenetic Analyses

The identified arthropod globins with full-length sequence information were complemented with selected deuterostome globins, representing sequences of all known globin clades (Blank and Burmester, 2012), and two plant globins as outgroups. Multiple sequence alignments were constructed using several algorithms: three different strategies of MAFFT v7 (L-INS-i, E-INS-i, and G-INS-i strategy) (Katoh and Stanley, 2013), Muscle (Edgar, 2004), Kalign2 (Lassmann et al., 2009), T-Coffee (Notredame et al., 2000), and Promals3D (Pei et al., 2008). The different alignments were scored using MUMSA (Lassmann and Sonnhammer, 2005, 2006) and the best-scoring alignment, the one obtained under the L-INS-i strategy from MAFFT, was used for further analysis (Supplementary Table S3).

Phylogenetic relationships of globin amino acid sequences were estimated using maximum likelihood (ML) and Bayesian analyses (BA). ML analyses were run using IQ-Tree v1.6.10 (Nguyen et al., 2015) in the implementation of IQ-Tree available from the IQ-Tree web server^5^ (Trifinopoulos et al., 2016) last accessed on March 2019, and support for the nodes was evaluated with the Shimodaira-Hasegawa approximate likelihood-ratio test and the aBayes tests from Anisimova et al. (2011) and 10,000 pseudoreplicates of the ultrafast bootstrap procedure (Hoang et al., 2018). The best-fitting model of substitution (LG + R10) was selected using the ModelFinder subroutine from IQ-Tree (Kalyaanamoorthy et al., 2017). Bayesian reconstructions were performed using MrBayes v3.2.6 (Huelsenbeck and Ronquist, 2001; Ronquist and Huelsenbeck, 2003) implementation available on Cipres^6^ (Miller et al., 2010) last accessed on March 2019, under a mixed model of substitution, running four simultaneous chains for 2 × 10^7^ generations, sampling trees every 1000 generations, and using default priors. Once the analyses were done, we verified that the estimated sample size (ESS) exceeded the recommended value of 200 using Tracer v1.7.1 (Rambaut et al., 2018). We then assessed convergence by measuring the standard deviation of the split frequency among parallel chains. Chains were considered to have converged once the average split frequency was lower than 0.01. We discarded trees collected before the chains reached convergence, and we summarized results with a majority-rule consensus of trees collected after convergence was reached. As a final verification step, we used the GUIDANCE2 server (Sela et al., 2015) to check the reliability of the multiple sequence alignment used for phylogeny estimation (via IQ-Tree with substitution model LG + R6). The overall GUIDANCE score for the alignment (Supplementary Table S4), which is calculated as the average score for all columns, was 0.935 slightly higher than the 0.93 cutoff value recommended by the default settings. A tree with the columns with scores lower than 0.93 removed did not differ much from the tree derived from the full alignment, and the differences were restricted to regions with low bootstrap support in the two trees. Importantly, inferences regarding the principal groupings of arthropod globins were not affected.

### Prediction of Myristoylation and Palmitoylation Motifs in Globin Sequences

The globin amino acid sequences were checked for motifs indicating N-terminal posttranslational acylation. For the prediction of N-myristoylation, we used the online tools Myristoylator^7^ (Bologna et al., 2004), PROSITE^8^ (Sigrist et al., 2002) and NMT-Predictor^9^ (Maurer-Stroh et al., 2002). Potential 3C-palmitoylation sites were predicted employing CSS-Palm 3.0^10^ (Ren et al., 2008).

### Determination of the GbXL Sequence of *Chironomus riparius*

To determine the GbXL sequence of *Chironomus riparius* for subsequent expression analyses, transcriptome data (Schmidt et al., 2013) were searched for GbXL sequences by TBLASTN using the GbXL sequence of *A. mellifera* as query. Two hits were identified, spanning 61 amino acid sites of the 5′ end and 108 sites of the 3′ end of the GbXL coding sequence, respectively. To verify the full length sequence, cDNA of whole L4 larvae was synthesized as described below and a PCR was performed using the following primers: 5′-ATGGGGTGTGAATTGGGAAAATTAG-3′ and 5′- TCATGA GGCATCATTGGATCTTG-3′. After cloning into the pGEM-T Easy vector (Promega, Mannheim, Germany), both strands of the amplicon were Sanger-sequenced by a commercial sequencing service (StarSEQ, Mainz, Germany; GenBank acc. no. MN956541).

### Analysis of Tissue-Specific Expression of Globins

Fat bodies, brains, Malpighian tubules, salivary glands and guts from *n* = 48 *Chironomus riparius* L4 larvae were dissected, and the same tissues were pooled to achieve sufficient amounts and stored in RNA-Later (20 mM EDTA, 25 mM sodium citrate; 5,3M ammonium sulfate, pH 5.2). The RNA from brains, Malpighian tubules, and salivary glands was isolated using the NucleoSpin RNA XS Kit (Macherey-Nagel, Düren, Germany) and diluted in 10 μl RNase-free water. The RNA from the fat body, the gut, and whole larvae was isolated with the RNeasy Kit (Qiagen, Hilden, Germany) and diluted in 30 μl RNase-free water. The quality and concentration of the RNA samples were accurately evaluated using a NanoDrop (ND1000, Thermo Fisher Scientific, Waltham, MA, United States) and a Bioanalyzer (2100 Bioanalyzer, Agilent Technologies, Santa Clara, CA, United States). cDNA was generated with the SuperScript III reverse transcriptase (Life Technologies, Darmstadt, Germany) according to the instructions of the manufacturer using oligo(dT)-primers. For each sample, 500 ng of total RNA was applied. Quantitative real-time reverse transcriptase PCR (qRT-PCR) was performed on a 7500 Fast Real-Time PCR System (Applied Biosystems, Darmstadt, Germany) using the QuantiTect SYBR Green PCR Kit (Qiagen, Hilden, Germany) and the following primers matching the GbXL CDS of *C. riparius*: 5′-AAGTAATGGAGACGATGGATGAG-3′ and 5′-CGGTCACCTAATGTATCTGAAAC-3′. For each sample, 1 μl of cDNA (equivalent to 36 ng of total RNA) were used in a 10 μl PCR volume. In the absence of validated reference genes, normalization between samples was thus performed on total RNA amounts (mostly reflecting rRNA). Gene expression levels were then calculated by the classical “standard-curve” approach by measuring Ct-values. For standard-curve preparation, plasmids containing the GbXL CDS were diluted 10-fold (10^7^ to 10^1^ copies) and measured in parallel to the cDNA samples. Each sample was measured in triplicate (technical replicates) and the mean Ct-value was calculated. To quantify differential gene expression, the expression in each tissue was calculated relative to the expression in the brain. The significance of the data was assessed by a two-tailed Student’s *t*-test employing the Microsoft Excel spreadsheet program.

An *in silico* analysis of globin expression was performed for the honeybee *Apis mellifera*. We downloaded 18 tissue-specific datasets from the NCBI short read archive^11^ (Supplementary Table S5). For Illumina datasets, we trimmed the 5′-ends, adapter sequences and low quality ends and filtered low quality reads (quality score below 20) using the fastx_toolkit^12^. 454 pyrosequencing reads were trimmed and filtered with the CLC Genomics Workbench 5.5 (CLC Bio, Aarhus, Denmark), respectively. To calculate RPKM values (Mortazavi et al., 2008), the reads were mapped against the *Apis mellifera* genome v4.5 (GCF_000002195.4) using the tool *RNA-Seq Analysis* with default parameters from the CLC Genomics Workbench 5.5. To determine the significance of differential GbXL expression in the different datasets, the log likelihood ratio Rj-statistic (Stekel et al., 2000) was applied.

## Results and Discussion

### Respiratory Proteins in Arthropods: Globins Are Present in All the Main Lineages

The availability of complete genomes and transcriptomes of many different species provides the possibility to study systematically a large variety of arthropod taxa for the presence of respiratory proteins and their pattern of expression. The copper-containing oxygen-binding hemocyanins have been identified in all arthropod phyla, but certain taxa have lost this type of respiratory protein. For example, no hemocyanin sequences have been found in the genomes of eumetabolan insects (Holometabola + Heteroptera) (Pick et al., 2009; Burmester, 2015). The reason for the loss of hemocyanin is essentially unknown, but may be related to the development of more efficient mechanisms of O_2_ uptake or other metabolic changes. At least in some arthropod species, hemocyanin has been replaced by hemoglobin as the principal respiratory protein (Weber and Vinogradov, 2001; Burmester and Hankeln, 2007; Burmester, 2015). However, the diversity and evolution of globins in the arthropod phylum have remained poorly studied. Therefore, we carried out a systematic search for globin genes in the genomes and transcriptomes from representative arthropod taxa. We identified 245 globins with 272 globin domains from 84 arthropod species. In addition, three globin sequences were identified in the water bears (Tardigrada). The full list of globin sequences is given in Supplementary Table S1. The results of our bioinformatics searches indicate that all of the arthropod species with a fully sequenced genome include globin genes in their genome, and in fact, at least two distinct globin genes were detected per genome. Thus, in contrast to early interpretations of comparative genome analyses (Rubin et al., 2000), our results indicate that globins are part of the standard gene repertoire of the arthropod genome. In fact, the phylogenies described below indicate the presence of multiple globin genes in the last common ancestor of arthropods.

### Phylogeny Reconstruction Identified Three Major Globin Lineages in Arthropods

For the purpose of phylogeny estimation, we assembled a dataset of sequences from 243 globins that feature the classical globin fold tertiary structure. We excluded the chimeric Adgb gene because of its heavily modified globin fold (see below). The final gene set included 184 arthropod globins and 57 deuterostome globins, plus two plant globins that were included as outgroups: the LegHb1 protein of alfalfa (*Medicago sativa*) and the LegHb2 protein of yellow lupine (*Lupinus luteus*). In the case of arthropod taxa that harbor multiple closely related globin paralogs, such as the HbL paralogs in the midge *Chironomus riparius* and in branchiopods, we included a representative subset of sequences in the analysis. Otherwise, all arthropod globins with complete sequence information were included. The non-vertebrate deuterostome reference set included the full globin complement of the amphioxus *Branchiostoma floridae*, the acorn worm *Saccoglossus kowalevskii* and the sea squirt *Ciona intestinalis*, which have been used in previous analyses (Ebner et al., 2010; Hoffmann et al., 2012; Opazo et al., 2015). The vertebrate gene set was represented by its major vertebrate-specific globins, i.e., Hb, Mb, Cygb, GbE, and GbY, plus the more ancient Ngb and GbX (Burmester and Hankeln, 2014). Together, these proteins cover all major globin clades that have been previously identified in metazoans (Blank and Burmester, 2012; Hoffmann et al., 2012; Hoogewijs et al., 2012). For this set of sequences, alternative multiple sequence alignments were constructed using different programs and algorithms. The alignment obtained by MAFFT using the L-INS-i strategy gave the highest MUMSA score. This alignment, which covers a total of 619 amino acid positions (Supplementary Table S3), was used for phylogenetic reconstruction using Bayesian and maximum likelihood approaches.

**FIGURE 1:**
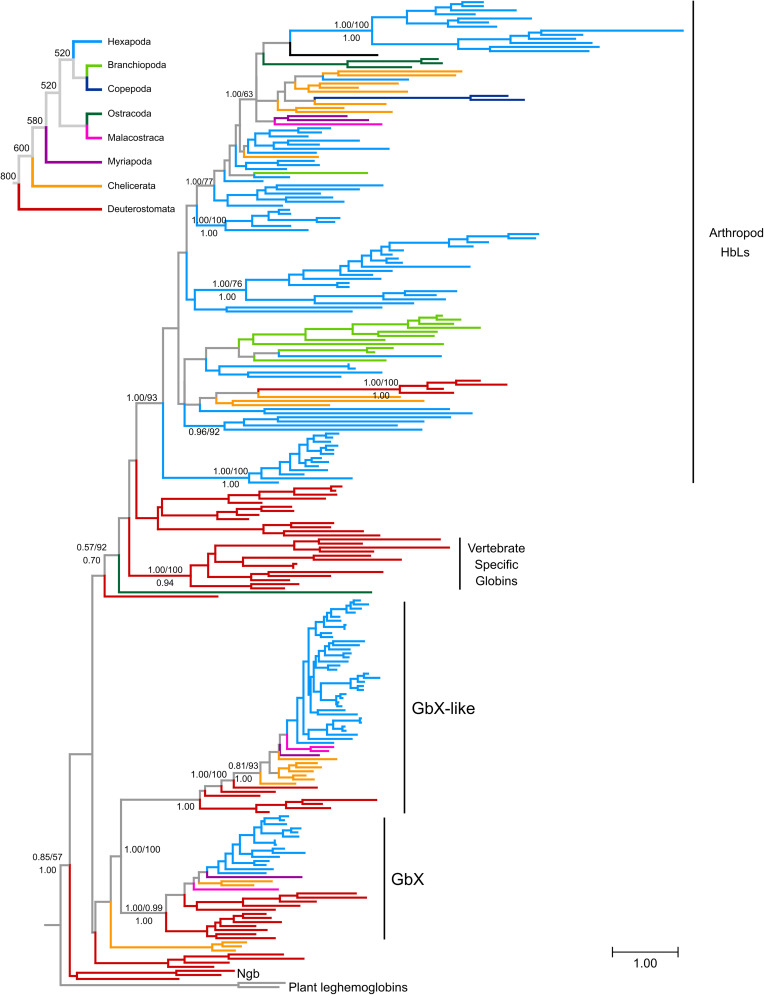
Maximum likelihood phylogram depicting relationships among globin genes from representative arthropods and deuterostome taxa. Numbers above the nodes correspond to support from the aBayes test and 1,000 pseudoreplicates of the ultrafast bootstrap procedure, whereas numbers below the nodes correspond to Bayesian posterior probabilities from MrBayes. The tree was rooted using plant globins as outgroup sequences. The inset on top shows the organismal phylogeny for the representative arthropod lineages included in the analysis, following Misof et al. (2014), with divergence time estimates derived from the TimeTree server (Kumar et al., 2017). Terminal branches were colored following the organismal tree. The full version of this figure is available as Supplementary Data Sheet S1.

Previous studies grouped deuterostome globins into four major clades that diverged before the split of protostomes and deuterostomes (Blank and Burmester, 2012; Hoffmann et al., 2012; Hoogewijs et al., 2012). Three of these clades are defined by the presence of vertebrate genes: neuroglobin (Ngb), globin X (GbX), and the vertebrate specific globins, the group that includes Hb, Mb, and Cygb. In the current study, we refer to this latter group of proteins as hemoglobin-like (HbL) because they are grouped with vertebrate hemoglobins, a designation that has no further functional implications. The fourth clade is defined by the presence of a set of globins from invertebrate deuterostomes that are structurally and phylogenetically similar to GbX, and are labeled as globin X-like globins (GbXL). Overall, our tree is consistent with these previous analyses (Figure 1 and Supplementary Data Sheet S1), recovering the monophyly of the groups defined by GbX, GbXL and vertebrate specific globins, which is labeled as HbL, placing vertebrate Ngb and their putative ortholog from amphioxus as the deepest diverging lineage. Our analyses also identified additional groups of globins with unclear affinities such as the clade of acorn worm Gbs 11–14, a clade of three chelicerate globins (Black-legged tick HbL5, and Bark scorpion HbL2 and HbL3), amphioxus Gb 8 or seed shrimp (Puriana) HbL2. All other arthropod globins fell into the GbX, GbXL or HbL groups of globins. In the case of the GbX and GbXL clades, arthropod GbX and GbXL sequences were recovered in strongly supported clades that largely reflect the arthropod phylogeny (Figure 1 and Supplementary Data Sheet S1). In both cases, arthropod sequences displayed short branch lengths, consistent with the conservative sequence evolution of these globins (see below; Roesner et al., 2005).

The vast majority of arthropod globins were grouped in the HbL clade that also includes vertebrate-specific globins and their deuterostome homologs (Figure 1 and Supplementary Data Sheet S1). Within this large group, relationships among the sequences deviate substantially from the expected arthropod phylogeny, probably reflecting a complicated history of lineage-specific gene gains and losses. In several cases, the grouping of sequences is congruent with conventional taxonomic arrangements. For example, lepidopteran sequences fall into a single clade and dipteran sequences are arranged into two separate clades. However, chelicerate HbL genes fall into 7 distinct clades and hexapod globins fall into 14 distinct clades. Unfortunately, resolution of the key nodes is low, making it impossible to fully reconstruct HbL history at this stage.

**FIGURE 2:**
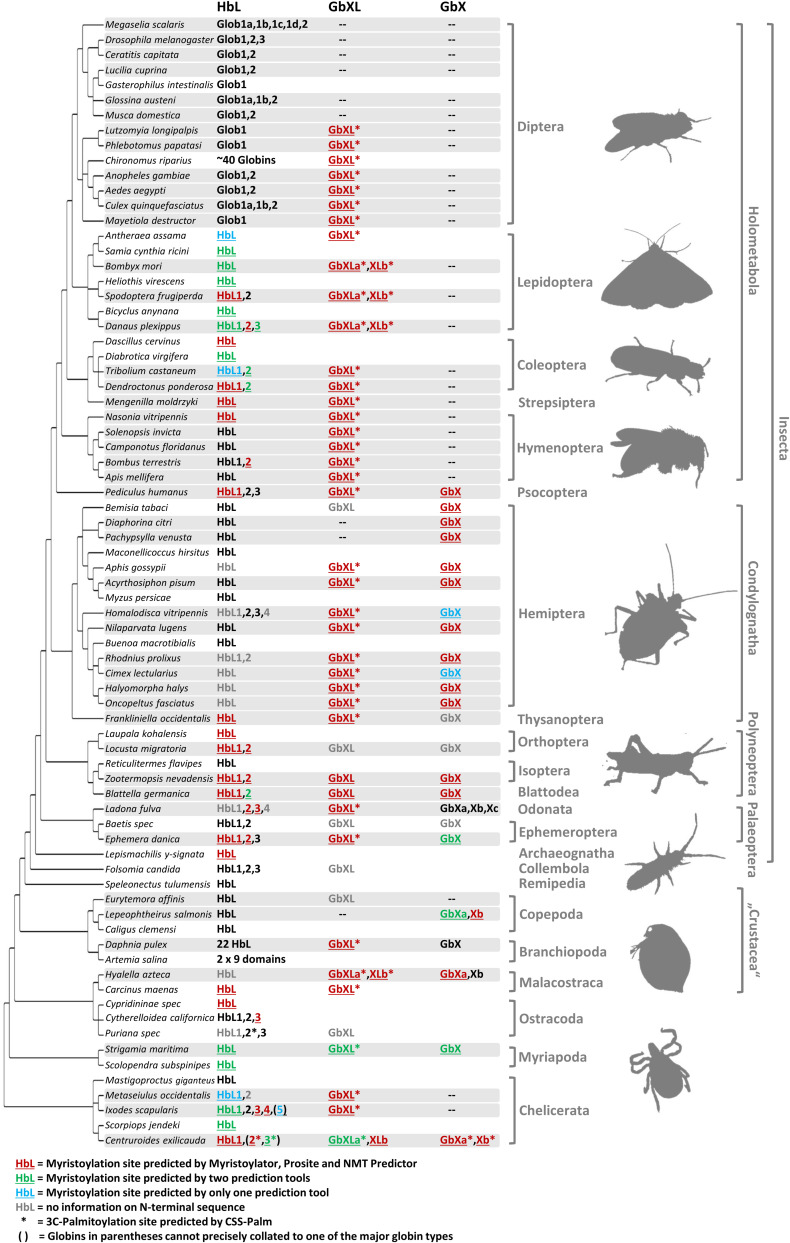
Different globin lineages are present in different arthropod taxa. The organismal phylogeny of arthropods was taken from Misof et al. (2014). Shading indicates taxa with available genome data. Globin sequences containing predicted protein acylation motifs are colored and annotated by underlining (N-myristoylation) and asterisks (3C-palmitoylation). Globins with uncertain N-terminal sequences are shown in gray. Note the following for the designation of the HbL variants: within Diptera, only the name “Glob1” designates putative orthology to the Glob1 gene of *Drosophila melanogaster* (Burmester and Hankeln, 1999), as defined by phylogenetic tree reconstruction. Closely related paralogues of Glob1 are named “Glob1 a, b, c” etc. Outside Diptera, all gene variants have been designated by “HbL” and numbering of paralogues does not indicate orthology. The comparatively rare cases of GbX and GbXL gene duplicates have been assigned as “a, b, c”.

Our reconstruction of arthropod globin relationships confirms the concept of a basic sub-division of the metazoan globin tree and suggests that arthropod globins, which have a classical globin fold, fall into either one of three main globin lineages, representing the HbL, the GbX and the GbXL globin classes (Figures 1, 2 and Supplementary Data Sheet S1). These three globin types (plus a fourth, the Ngb; see below) probably emerged early in metazoan evolution before the split of protostomes and deuterostomes. Within each of the three major globin clades, most of the arthropod globins were strictly separated from those of deuterostomes, suggesting an independent origin of the variants within each taxon.

Taken at face value, reconciling the tree in Figure 1 (also in Supplementary Data Sheet S2) with the organismal tree indicates that the last common ancestor of extant arthropods included at least copies of GbX, GbXL and HbL in its genome, and the HbL subtree suggests a history of multiple rounds of duplication, but further resolution of this issue would require phylogenies with higher levels of support. Thus, these phylogenies indicate that the GbX, GbXL, and HbL gene lineages can be traced back to the last common ancestor of deuterostomes and arthropods, and the presence of additional groups of deuterostome and arthropod globins in our trees is suggestive of the presence of additional uncharacterized globins in the common ancestor of deuterostomes and arthropods, which would indicate that the globin repertoire of early animals is substantially more diverse than previously imagined. The small size of the globin proteins and the antiquity of some of the putative duplication events combine to make this a challenging problem to resolve conclusively.

### Arthropods Lack Neuroglobin and Possess Androglobins With Degenerated Globin Domains

After its discovery in mammals, Ngb has been recognized as an ancient globin type present in many metazoan taxa, including deuterostomes, protostomes (Burmester et al., 2000; Ebner et al., 2010), cnidaria (Blank and Burmester, 2012), and placozoa (Dröge et al., 2012). Its early evolutionary origin and widespread occurrence have suggested an important functional role, which has been supported by many studies showing a cell-protective effect of Ngb in mammalian neurons (Review: Burmester and Hankeln, 2014). We note that we did not find a single putative ortholog of Ngb in arthropods, which suggests that this gene lineage was probably lost in the common ancestor of arthropods, a pattern also observed for cartilaginous fish (Opazo et al., 2015). The complete lack of Ngb in arthropods leads us to speculate that the functions of this globin may have been taken over by other members of the gene family. It should be noted here that some arthropod globins are annotated as “neuroglobin/Ngb” in public databases (e.g., NP_001191927.1, XP_001946608.3), however, they actually represent either GbX or GbXL genes according to our analyses.

Androglobin is the most recently discovered addition to the metazoan globin SUPERFAMILY (Hoogewijs et al., 2012), and it is substantially different from the classical globins discussed above. This multidomain chimeric protein possesses a calpain -domain, a calmodulin-binding motif and a structurally rearranged globin domain. In mammals, Adgb is mainly expressed in the testes and may play an important role in spermatogenesis. Adgb has been found in the genomes of a broad range of metazoan taxa, including arthropods like the honeybee, the carpenter ant and the human louse (Hoogewijs et al., 2012). However, the globin domain in these species proved to be degenerated. We identified twelve additional *Adgb* genes in arthropods, from most of the insect orders (Supplementary Data Sheet S2). However, no *Adgb* gene was found in the dipterans or in any arthropod subphylum other than the hexapods. Moreover, none of the potential globin domains of the hexapod Adgbs seems to be functional. Homology detection by FUGUE showed only weak, degenerated globin domains in some cases, whereas the NCBI CD-search identified none of the sequences as a globin (Supplementary Data Sheet S2). Normally, functioning globin proteins have two conserved histidines at positions E7 and F8, which are important for oxygen binding (see below). In most metazoan Adgbs, the distal E7 histidine is replaced by a functional, conservative glutamine (Hoogewijs et al., 2012). However, all hexapod Adgb proteins lacked both the distal E7 histidine/glutamine and the proximal F8 histidine (Supplementary Data Sheet S2). Thus, the Adgb globin domain in all arthropods appears to be heavily degenerated. The expression patterns of arthropod Adgb remains to be elucidated, so it is unclear whether its name, implying specific functionality in males, is appropriate in non-vertebrates.

### HbL and GbXL Globins Are the Most Widely Distributed Variants in Arthropods

The occurrence of the different types of globins was mapped onto the arthropod tree (Figure 2). In most arthropod species, *GbX* and *GbXL* are single-copy genes; we only found an additional copy of *GbXL* in moths, the scud *Hyalella azteca* and the bark scorpion, *Centruroides sculpturatus* (formerly *exilicauda*). Surprisingly, this GbXL, a paralogous globin class that was first identified in deuterostomes and some protostomes, but which is absent from vertebrates (Blank and Burmester, 2012; Hoffmann et al., 2012), was detected in almost every arthropod taxon with whole genome data except for the brachyceran flies with its iconic model species Drosophila. Only the two psyllids, *Diaphorina* and *Pachypsylla*, and the copepod *Lepeophteirus* apparently also lack GbXL, although the apparent absence of the gene could also be attributed to an incomplete genome assembly. By contrast, GbX was not found in the large group of holometabolan insects, in mites and ticks (Acari) or in the copepod *Eurytemora*. The quantity and quality of sequence data from the Holometabola and Acari makes it unlikely that we could have failed to detect the presence of the *GbX* gene due to assembly gaps. Rather, *GbX* appears to have been lost on several independent occasions in arthropod evolution, suggesting that it is dispensable for some taxa. It may be speculated that GbX and GbXL can substitute for each other in terms of molecular function, but much more detailed knowledge on the functional roles of these paralogs is required. We did not find any evidence for globins with multiple GbX- or GbXL-domains, suggesting that their single chain monomeric structure is important for their functions, which may be unlikely to be related to respiration (Blank et al., 2011).

Hemoglobin-like was found in all arthropod species, suggesting that this globin type is an indispensable cornerstone of the arthropod globin gene repertoire. In contrast to GbX/GbXL, the HbL lineage is characterized by multiple gene duplications which occurred independently in different arthropod lineages. The number of *HbL* copies usually ranges between 1 and 4. In two cases we documented a dramatic increase in the number of *HbL* gene copies: (1) the non-biting midges with more than 40 HbL variants (Kao et al., 1995; Trewitt et al., 1995; Hankeln et al., 1998), which are mostly considered to ensure O_2_ transport in the hemolymph of the aquatic chironomid larvae (Weber and Vinogradov, 2001) and (2) the branchiopod crustaceans, where Hbs are hypoxia-inducible and thus also probably relevant for respiration (Zeis et al., 2003; Guadagnoli et al., 2005).

### Evolution of Arthropod Globin Gene Structure

**FIGURE 3:**
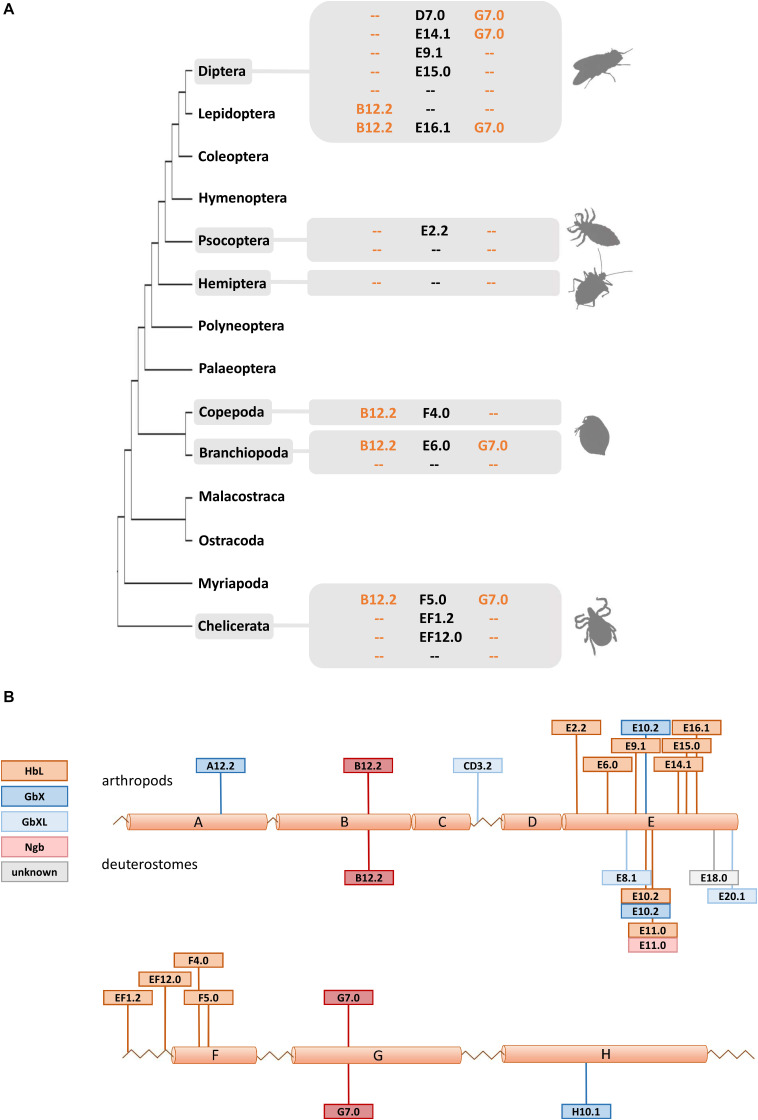
Intron evolution in arthropod globin genes. **(A)** The standard arthropod globin gene harbors the conserved intron positions named B12.2 and G7.0 (see text). Here we highlight that variable intron positions were often found in arthropod HbL genes suggesting multiple cases of intron loss and intron gain. **(B)** Summary of intron positions observed along the arthropod and deuterostome globin fold (with a helices A to H). Note the conservation of the B12.2 and G7.0 intron positions and the positional variability of introns interrupting the central part of the globin fold (a helices E and F).

Due to their phylogenetic ancestry and presence in all kingdoms of life, globins and their genes have been a model for investigating the evolution of intron-exon structure, which is underlying the “introns-early” versus “introns-late” debate (Rogozin et al., 2012). Two ancestral introns at the positions B12.2 (between codon positions 2 and 3 of the 12th amino acid of the B-helix) and G7.0 are highly conserved and present in many globins from animals and even plants. In fact, the possible adaptive value of this extraordinary conservation of intron positions is still not known. In addition, so-called central introns were found at variable positions in the E-helix of globins from a diverse range of taxa and interpreted as evidence for either a sliding of ancient introns or intron gain (Go, 1981; Dixon and Pohajdak, 1992; Stoltzfus and Doolittle, 1993; Hardison, 1996). Investigating arthropod globin genes, we observed in part a very conservative evolution of intron-exon structure, but also a substantial degree of variability (Figure 3 and Supplementary Table S1): Almost all arthropod *GbXL* genes showed the three-exon-two-intron structure with the conserved introns at B12.2 and G7.0. Only *GbXL* of the mite *Metaseiulus occidentalis* had an additional intron inserted between globin helices C and D (CD3.2), but lacked the ancestral B12.2 intron. The B12.2/G7.0 architecture appears to be ancestral for metazoan *GbXL*, since it is shared with deuterostome *GbXL* (some of which display additional central introns at divergent, non-homologous positions).

Globin X of arthropods typically displayed a four-exon-three-intron structure: in addition to the “classical” introns at B12.2 and G7.0, a third central intron at position E10.2 is present in most *GbX* orthologs. *GbX* lacks the E10.2 intron only in the centipede *Strigamia maritima* and the scorpion *Centruroides sculpturatus*, indicating secondary losses in these two taxa. Furthermore, an additional intron was found in *GbX* of the water flea *Daphnia pulex* at position A12.2 and the louse *Pediculus humanus* in the N-terminal extension, respectively. The E10.2 position in arthropod *GbX* is shared with the *GbX* orthologs of vertebrates and other taxa (Roesner et al., 2005; Blank et al., 2011), suggesting that it represents the ancestral state in metazoan *GbX*. However, it is unclear if the E10.2 intron, which distinguishes the *GbX* and *GbXL* genes, was already present before the ancient duplication of these variants.

The intron positions in the arthropod *HbL* genes turned out to be more variable. Most *HbLs* again showed the typical introns at B12.2 and G7.0, but variations were found in several taxa (Figure 3). Examples are the *HbLs* of the dipterans: the *Glob1* genes of the Brachycera and the nematoceran Hessian fly *Mayetiola destructor* lost the intron at B12.2 and gained an intron at D7.0. Likewise, *Glob1* orthologs of the Psychodomorpha (*Lutzomyia longipalpis* and *Phlebotomus papatasi*) lack the intron B12.2, but have acquired a central intron at position E14.1. In mosquitoes, the *Glob1* of *Aedes aegypti* and *Culex quinquefasciatus* gained a third central intron at E16.1, whereas *Glob2* lacks the intron in the G-helix in all three analyzed mosquito species (cf. Burmester et al., 2007). Most of the multiple *HbL* copies of the midge *Chironomus* are intronless or have only a single intron in the E-helix at positions E9.1 or E15.0 (cf. Hankeln et al., 1997). Amongst the ca. 40 *Chironomus riparius HbL* genes, only a variant designated *3A* shows the typical ancestral introns at B12.2 and G7.0, making this gene copy a possible candidate for the ancestral Chironomus *HbL* gene, which gave rise to the multitude of duplicated paralogues in this taxon. Further deviations from the common intron pattern were found in the *HbL* of the louse *Pediculus humanus*, the hemipterans *Homalodisca vitripennis* and *Nilaparvata lugens*, the mayfly *Ephemera danica*, in crustaceans (the salmon louse *Lepeophtheirus salmonis* and the water flea *Daphnia pulex*) and in the Acari *Metaseiulus occidentalis* and *Ixodes scapularis*.

By plotting the intron positions of arthropods and deuterostomes onto the globin fold structure, a considerable concentration of newly evolved globin intron sites around the helices E and F could be observed (Figure 3). Surprisingly, 15 out of 18 variant intron sites are positioned in or between these helices. The reason for this obvious bias is unclear, and the hypothetically underlying mechanisms of intron gain are still a matter of debate (for review, see Roy and Gilbert, 2006; Yenerall and Zhou, 2012). Specifically in globins, different mechanisms of intron gain have been postulated, namely an intron transfer between paralogous genes in the midge *Chironomus thummi* (Hankeln et al., 1997) and an intron gain caused by tandem genomic duplication in the lancelet *Branchiostoma floridae* (Ebner et al., 2010). Whereas the first model leads to new introns at the same position within paralogues, the latter explains the gain of entirely novel intron sites. We suggest that the diversity of gene structure observed in arthropod globins could make them suitable candidates for studying the underlying molecular mechanisms and potential functional consequences of intron gain, intron loss and intron site conservation.

### Structural Diversification of Arthropod Globins and Functional Implications

A comparison of the three globin types with a classical globin fold domain revealed differences in their protein lengths. The shortest arthropod globin variants are the HbL globins, which span on average 171 amino acids. Arthropod GbX (228 aa) and GbXL (199 aa) are considerably longer, mainly due to N-terminal extensions of the globin core. In the case of arthropod GbX, the extensions are substantially longer than those of the vertebrate orthologs, having an average length of 195 aa. Future protein structural studies should address this unexplained, but potentially relevant feature.

Most classical globin proteins have three amino acid positions that are highly conserved because they are directly involved in heme coordination and ligand binding. These are a phenylalanine at position CD1 (i.e., the first position in the loop between the helices C and D), and a distal and proximal histidine at helix positions E7 and F8, respectively. By studying the multiple sequence alignments, we found that these functionally important residues are overall very well conserved in arthropod globins. In 173 of the 187 analyzed globin sequences, all three important amino acids were present, which is indicative of an intact heme and capacity for O_2_-binding. These sites are highly conserved in arthropod GbX and GbXL, but substitutions at the same residue positions were observed in 14 HbL globins from Diptera, Branchiopoda, and Ostracoda. Most deviations affected the distal histidine position E7: three HbL of dipteran flies (MscGb1A, CriHb3A, and AgaGb2) had a glutamine at E7, a replacement that has been observed in various other Hbs and is considered functionally conservative (Nagai et al., 1987). The E7 His > Glu replacement was also found in the di-domain HbL and the monodomain HbB and HbG of the water flea *Daphnia pulex*; other monodomain HbL variants of this species display yet other amino acids at E7, namely serine, alanine, arginine, and leucine. Leucine at E7 was previously observed in globins 6, 12 and 13 of the lancelet *Branchiostoma floridae* and may lead to a reduced O_2_-affinity (Springer et al., 1994; Ebner et al., 2010). Notably, in some monodomain HbLs of *Daphnia*, the otherwise invariant proximal histidine at position F8 was replaced by tyrosine (DpuHbC, E, F, and K) or valine (DpuHbB). It is therefore questionable whether these HbL domains can still bind O_2_. The most striking deviation from the standard globin pattern was found in HbL2 of the ostracod *Puriana*, which turned out to be the only globin that carries mutations at all three functionally important sites (MetCD1, ValE7, ValF8). These drastic changes go along with a deviant position in the phylogenetic tree (Figure 1) and make it likely that this crustacean globin acquired an alternative function, other than classical O_2_ supply, which remains to be investigated. Of note, a non-heme-binding globin domain protein has been reported in *Bacillus anthracis*, where it has been implicated in sensing fatty acid metabolism, chloride ions, and/or pH (Stranzl et al., 2011).

**FIGURE 4:**
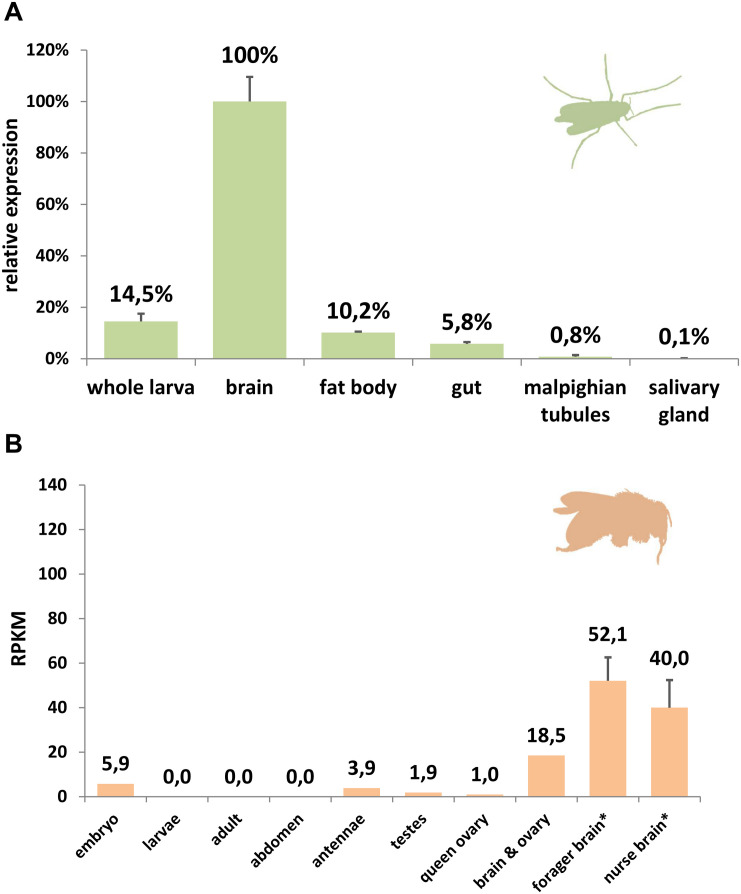
Expression analysis of GbXL. **(A)** qRT-PCR in larval tissues of the dipteran midge *C. riparius*. The expression level in brain was set to 100%. **(B)** RNA-Seq expression analysis of GbXL transcription in different tissues of the honeybee *A. mellifera*. Expression levels are represented by RPKM values. ^∗^: For forager and nurse brain, five datasets were analyzed and the mean value is given. All other datasets were single (Supplementary Table S5).

Comparing rates of amino acid substitution in the arthropod globin variants we observed that HbL, GbX, and GbXL showed substantially different degrees of sequence conservation. GbX and GbXL both turned out to be the most highly conserved globin classes. On average, the globin core of arthropod GbXL displayed an identity score of 67% and a similarity of 83% (based on a BLOSUM62 matrix). For GbX, the average identity/similarity score was 68%/81%. The substitution rate, which is low compared to other globins (Roesner et al., 2005), may indicate that amino acid residues in GbX and GbXL do not only build a globin fold, which is rather flexible in amino acid composition (Lesk and Chothia, 1980), but may also perform a cellular function that involves direct interactions with other proteins. This is in particular true of GbXL, which is ubiquitous in arthropods orders, but has not yet been subject to studies of protein structure. Because of this, we gathered initial information on the dominant expression sites of *GbXL* in arthropod species. qRT-PCR experiments on dissected larval tissues of the dipteran midge *C. riparius* and analysis of public RNA-Seq data from developmental stages and adult organs of the honeybee *A. mellifera* indicated that *GbXL* is mainly expressed in brain tissue (Figure 4). This coincides with the dominant expression pattern of *GbX* in the vertebrate (zebrafish) brain (Blank et al., 2011), where GbX has been hypothesized to protect lipids in cell membranes from oxidation or to act as a redox-sensing signaling protein. It remains to be investigated if the cellular expression sites of *GbX* and *GbXL* overlap in arthropods, which might then indicate a scenario of sub-functionalization between these ancient paralogs.

In contrast to GbX and GbXL, arthropod HbL proteins exhibit an average identity score of only 25% (46% similarity), which is in line with the considerably longer branch lengths observed for HbL in phylogenetic trees (Figure 1). The faster substitution rate is reflected by the lower conservation of crucial amino acid positions and the observed higher variability in gene structure (see above). In contrast to GbX and GbXL, that largely reflect the arthropod phylogeny, the HbL variants are grouped at variable positions in the HbL subtree (comp. Figure 1 and Supplementary Data Sheet S1). It suggests that the cellular functions of HbL variants in arthropods are much more flexible and diverse. In fact, we have previously reported that the HbL variants in *D. melanogaster*, with Glob1 on the one hand and a duplicated gene pair named Glob2 and 3 on the other, display completely different structural characteristics and gene expression profiles. While Glob1 is currently thought to fulfill a role associated with oxidative metabolism in tracheoles and fat body cells of embryonal larval and adult flies (Hankeln et al., 2002; Gleixner et al., 2008, 2016), Glob2 and 3 are exclusively expressed during spermatogenesis and may be important for male fertility (Gleixner et al., 2012). In conclusion, the multiple gene duplication events observed within the HbL class may have provided the basis for neo-functionalization events in the different arthropod lineages, which of course has to be proven experimentally.

### N-Terminal Protein Acylation Motifs Are Widespread in Most Arthropod Globin Classes

Along with the discovery of several new members of the globin gene family in vertebrates, a number of hypotheses have been raised concerning their non-classical functions in vertebrate cells (Burmester and Hankeln, 2014). In this respect, the recent finding that globins may indeed be attached to the cell membrane has received particular interest: using cell biological and biochemical methods, it has been demonstrated that the GbX protein of the zebrafish *Danio rerio* is bound to the cell membrane via N-terminal myristoylation and S-palmitoylation (Blank et al., 2011). Furthermore, it has been shown that GbX is able to protect cells from ROS *in vitro* (Koch and Burmester, 2016). This precludes a standard respiratory function of GbX and instead suggests a role in the protection of membrane lipids from oxidative stress (Koch and Burmester, 2016). Based on these previous findings we performed a systematic bioinformatics search for the presence of candidate protein acylation sites in arthropod GbX and GbXL, but also in the HbL variants. The search for putative myristoylation motifs employing three different algorithmic tools showed a widespread distribution of these sites among all arthropod globin classes (Figure 2). In total, we identified 112 globins with a putative myristoylation motif, of which 86 were predicted by all three tools. Additional 20 globins were predicted to be myristoylated by two tools, and six potential sites were identified by a single prediction tool. In addition, we searched bioinformatically for putative palmitoylation sites. The vast majority of arthropod GbXL proteins was predicted to carry both, an N-terminal myristoylation and palmitoylation motif. Palmitoylation motifs were only absent in the GbXL proteins of the two polyneopteran species *Zootermopsis nevadensis* and *Blattella germanica* (Figure 2). By contrast, most of the arthropod GbX proteins appeared to have lost the palmitoylation site, but retained the myristoylation motif. In fact, only the GbX of the bark scorpion *Centruroides exilicauda* was predicted to be palmitoylated. As other metazoan GbX typically harbor both the myristoylation and the palmitoylation sites (Blank and Burmester, 2012), the loss of the palmitoylation motif may have occurred within the arthropods.

**FIGURE 5:**
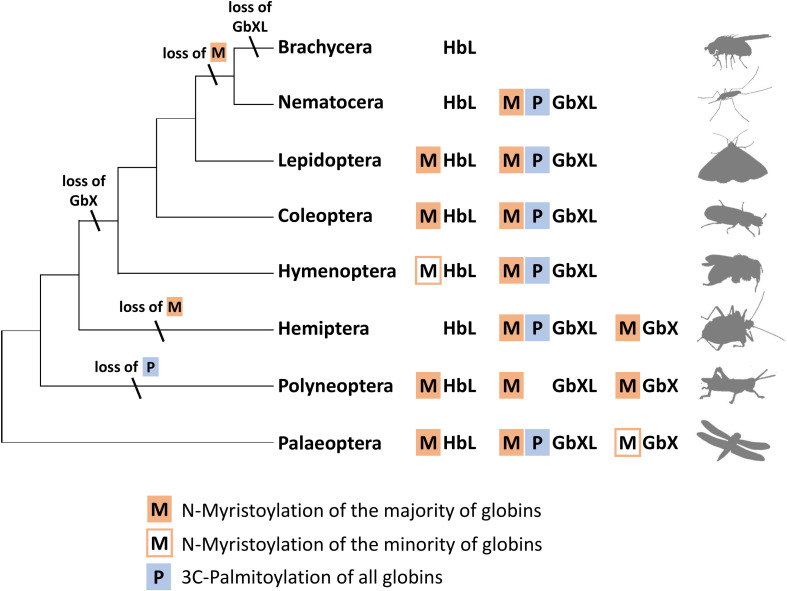
Hypothetical evolution of protein acylation motifs in insect globin genes. The reconstruction suggests that protein acylation was an ancestral state for all three globin types. Differential loss of acylation motifs occurred independently in several lineages. Note the reduced globin complement and complete loss of acylation motifs in brachyceran dipterans including Drosophila (M = N-myristoylation, P = 3C-palmitoylation).

Interestingly, an N-terminal myristoylation motif was also predicted in a larger number of arthropod HbL proteins. The phylogenetic distribution of HbL genes with acylation motifs suggests that this property was present in the ancestral arthropod HbL. In fact, the functionality of a myristoylation motif in membrane-binding has previously been reported for a HbL globin variant of the green shore crab *Carcinus maenas* (Ertas et al., 2011). Independent losses of the myristoylation site probably occurred in several arthropod taxa (Figures 2, 5). Noteworthy examples are the HbL proteins of the Hemiptera and Diptera, where a loss of the acylation motif coincides with an overall elevated rate of sequence change (as evidenced by long branches in the tree reconstruction; Supplementary Data Sheet S1). We also point out that the brachyceran dipterans including its model taxon Drosophila, already having lost globin lineages GbX and GbXL, appear to be the only arthropods, which lack *any* putatively acylated globin. The functional implications of this are currently unclear.

In summary, the overall widespread presence of candidate sequence motifs for protein acylation in all three arthropod globin lineages yields strong support to the hypothesis that a membrane attachment might reflect the ancient functional role of globins in this taxon and possibly also in metazoa (Ertas et al., 2011; Blank and Burmester, 2012). Future functional studies should therefore address this possibility experimentally. The presence of either a combination of the two acylation motifs or their differential loss (Figure 5) opens an additional way for an increased functional variability of arthropod globins during evolution. In the case of zebrafish GbX, *in vitro* experiments revealed that the differential presence or absence of acylation motifs indeed influenced the intracellular localization of the globin (Blank et al., 2011).

## Conclusion

The reported systematic bioinformatics search for globin sequences in arthropod genomes and transcriptomes revealed that all arthropods with available sequence data possess and express globin genes. The distribution of globin variants across the arthropod phylogeny suggests that the last common ancestor of arthropods, as well as the ancestor of the hexapods, harbored at least three distinct globin lineages: the diverse class of HbL genes and GbX and GbXL, which are typically present as single-copy genes.

In contrast to vertebrates (Burmester and Hankeln, 2014) and other deuterostomes and protostomes (Ebner et al., 2010; Dröge and Makalowski, 2011; Hoffmann et al., 2012), arthropods appear to have lost the Ngb lineage and display a heavily rearranged or degenerated globin domain inside the chimeric Adgb protein (Hoogewijs et al., 2012). Arthropods thus display only three of the five putatively ancestral globin types of metazoa. In addition, the holometabolan insects have lost their GbX, and the brachyceran dipterans have additionally lost GbXL, and therefore possess the least diverse globin repertoire within arthropods. To understand the full spectrum of globin function in arthropods, it is therefore dangerous to concentrate merely on the established model *D. melanogaster*. In principle, the remaining globins of the HbL type are possible candidates for compensating for the loss of the other globin paralogs. For example, Glob2 and 3 of Drosophila and their homologs in other Brachycera are candidates for an Adgb-related function due to their conspicuous testes-specific expression pattern. It has also been demonstrated in the mollusc clam *Spisula* that an HbL globin is highly expressed in nerve tissue, thereby possibly replacing neuroglobin (Dewilde et al., 2006).

With respect to possible globin functions, the presence of candidate protein acylation motifs in all three arthropod globin lineages requires particular attention in future experimental work, since it suggests that the arthropod globin ancestor may have been modified by N-myristoylation and, possibly, 3C-palmitoylation. In line with data for GbX in vertebrates (Blank et al., 2011; Blank and Burmester, 2012), our bioinformatics data suggest an ancestral and essential function of globins in relation to cell membranes, conceivably either by a protective role or by intracellular signaling.

The class of HbL globins in arthropods is characterized by multiple gene-duplication events and accelerated sequence evolution, suggesting the possibility of independent neo-functionalization events. Whatever the original cellular role of HbL globins might have been in arthropods, it may be that these genes were recruited for a functional respiratory function. HbL globins thus facilitate O_2_ supply in arthropod lineages adapted to low environmental O_2_ conditions [Branchiopoda, Chironomidae, *Gasterophilus*; for review, see Weber and Vinogradov (2001)] and some have highly specialized functions like regulating diving buoyancy in backswimmers (Matthews and Seymour, 2006; Wawrowski et al., 2012). Whether the HbL-type Glob1 of *D. melanogaster* is indeed also instrumental in oxidative metabolism is currently being investigated, and alternative molecular roles have been proposed on the basis of the modulation of Glob1 expression by RNA inference (Hankeln et al., 2002; Yadav et al., 2015; Gleixner et al., 2016). Genetic ablation tools including CRISPR technology will probably be required to solve these discrepancies.

## Data Availability Statement

The datasets generated for this study can be found in the GenBank acc. no. MN956541.

## Author Contributions

AP, FH, and JO conducted the bioinformatics analyses. AP and PH performed the expression analyses. AP, FH, JO, JS, TB, and TH evaluated and interpreted the data. AP, TH, TB, and FH drafted the manuscript, planned, and supervised the study. All authors read and approved the manuscript.

## Conflict of Interest

The authors declare that the research was conducted in the absence of any commercial or financial relationships that could be construed as a potential conflict of interest.

## Footnotes

1 https://www.ncbi.nlm.nih.gov/genbank/

2 https://datadryad.org/

3 http://structure.ncbi.nlm.nih.gov/Structure/cdd/wrpsb.cgi

4 http://mizuguchilab.org/fugue/prfsearch.html

5 http://iqtree.cibiv.univie.ac.at

6 http://www.phylo.org

7 http://web.expasy.org/myristoylator/

8 http://prosite.expasy.org/

9 http://mendel.imp.ac.at/myristate/SUPLpredictor.htm

10 http://csspalm.biocuckoo.org/

11 http://www.ncbi.nlm.nih.gov/sra/

12 http://hannonlab.cshl.edu/fastx_toolkit/

## Supplementary Material

TABLE S1 List of taxa and globin sequences.

TABLE S2 Amino acid sequences of globins.

TABLE S3 Multiple sequence alignment used for phylogenetic analysis.

TABLE S4 GUIDANCE2-edited multiple sequence alignment.

TABLE S5 Results of RNA-Seq analysis.

DATA SHEET S1 Full version of Figure 1 phylogenetic tree including species names.

DATA SHEET S2 Analysis of globin domains in arthropod Adgb.
