# Supplementary material for: The Globin Gene Family in Arthropods: Evolution and Functional Diversity
Source: Front Genet. 2020 Aug 13;11:858. doi: 10.3389/fgene.2020.00858 (PMC7457136; doi:10.3389/fgene.2020.00858)
Supplement: TABLE S2 — Amino acid sequences of globins. [file Table_2.DOCX]

>DmeGlob1

MNSDEVQLIKKTWEIPVATPTDSGAAILTQFFNRFPSNLEKFPFRDVPLEELSGNARFRA

HAGRIIRVFDESIQVLGQDGDLEKLDEIWTKIAVSHIPRTVSKESYNQLKGVILDVLTAA

CSLDESQAATWAKLVDHVYGIIFKAIDDDGNAK

>DmeGlob2

MSQISKLTHISRISQNNQSDGSDEDKFRRANFPVYPKPLPDRDLSYKADENEFTMVEKAS

LRNAWRLIEPFQRRFGKENFYSFLTRNEDLINFFRKDGKINLSKLHGHAMAMMKLMSKLV

QTLDCNLAFRLALDENLPTHLKNGIDPDYMRMLATALKSYILASSVIENHNSCSLSNGLA

RLVEIVGEYAVVDEARKRAMSTALRTTVDDAGNRIVKVALGT

>DmeGlob3

MMSEEVIAKNISLSSLTYPKRIPKIKFGPIKDEMGFTLSERLALRQAWNLVRPFERRYGQ

DVFYSFLNDYYWGIKKFRNGAELNVKALHSHALRFINFFGLLIEEKDPVVFQLMINDNNH

THNRCHVGSVNIGHLAQALVDYVLKVFHKVSSPSLEQGLSKLVEKFQNYQDQQSNTSGYN

RLSKVNFDSRPPRGNP

>CcaGlob1

MALNAEDIAEIKKTWAIPVATPTDSGAAILIRFFTKYPSNLEKFPFRDVPVAELNNSARF

RAHCGRIIKTFDQSISQLGEEGGLEKIQDIWQGIASSHVQRHNIPKPSYFELREAIVEVL

SEACNLNERQAEAWNKLLDIVYDIIFKKYDDLGAQ

>CcaGlob2

MPPIFPKALEPYDMKPVENELGLTITERRSLQNGWSIIKQKQRRAALTIYVNLFTEHENL

YEVFRSDGVLNIEFASQHQKEVLTVFQMIIEQVDNARFVKTMLKELALRHEAASVTNTQW

QLYTNEVRKYFLETLADAISPTFVHALDKLMNFVCNFNDLTESKEELHRVTRIK*

>GmoGlob1

MNSDEVYEIKRTWEIPATTPTESGVAILIRFFTKYPSNLQKFSTFKDMTLDELKNNPRFK

AHANRIMKVFDDSIKTLDDNCSHLEEIWTKIAQSHFNRQIEKQSFNELKEVILEVLVAAC

NLNDQQTEIWLKLLDFVYEIIFKTIDQLEQDV

>GauGlob1a

MNSDEVYEIKRTWEIPATTPTESGVAILIRFFTKYPSNLQKFSTFKDMPLDELKNNPRFK

AHANRIMKVFDDSIKTLDDNCSHLEEIWTKIAQSHFNRQIEKQSFNELKEVILEVLVAAC

NLNDQQTEIWLKLLDFVYEIIFKTIDQLEQDV*

>GauGlob1b

MNSDEVSEIKKTWEIPAASPTESGVAILIQFFTKYPSNLEKFSTFKDMPLDELKESPRFK

AHANRVIKVFDDSVQALDDDPSQLEEIWVKVTQSHFNRQIEKHSFNELKEVILEVLTAAC

SLNDQQIEIWIKLMDFIYDIIFRTIDELEQAA*

>GauGlob2

MIDSGIQYPKPLPTLNLENVCNEMGFTPLEIVALQNIWRLFKKRFKYHSMQIFLAFFNQN

HKLIERFRLPSGNFQLSHLCQHSEKMLLLYENVIDKCLDNMANFHGVMAEVTVKHQRCGV

KYEEIILKTEHVRRYILEYFANQSSPTLVSALAKLSEHFNDRHRPKEGESDVSEEGD*

>GbrGlob1a

MNSDEVYEIKRTWEIPATTPTESGVAILIQFFTKYPSNLQKFSTFKDMTLDELKDNPRFK

AHANRIMKVFDDSIQTLDGNCSNLEEIWTKIAQSHFNRQIEKQSFKELKEVILEVLVAAC

NLNDKQTEIWQKLLDFIYDIIFKTIDQLAHDV*

>GbrGlob1b

MNSDEVFEIKRTWEIPAASPTESGVAILIQFFTKYPSNLQKFSTFKDMTLDELKDNPRFK

AHANRIMKVFDDSVQALDDDYSQLEEIWIKVAQSHFNRQIEKQSFNELKEVILEVLAAAC

NLNDQQTEIWIKLMDFIYDIIFKTIDELEQDA*

>GbrGlob2

MIDNGGIEYPKPLPKLNLENVCNEMGFTPLEMVALQNIWRLFKKRFKHHSMQIFLAFFNE

NHKLIEKYRLPNGKFQLSYLCHHSEKMLILYENVIDKCLDNMANFHGIMADVTLSHRNYG

VTYEDVKLKCEHVKHYILNYFRNQSSPTLVSALEKLSEHFKERHRVKEGDTKSDEDEAVD

*

>GfuGlob1a

MNSDEVYEIKRTWEIPATTPTESGVAILIRFFTKYPSNLQKFSTFKDMTLDELKNNPRFK

AHANRIMKVFDDSIKTLDDNCSHLEEIWTKIAQSHFNRQIEKQSFNELKEVILEVLVAAC

NLNDQQTEIWLKLLDFVYEIIFKTIDQLEQDV*

>GfuGlob1b

MNSDEISEIKKTWEIPAASPTESGVAILIQFFTKYPSNLEKFSTFRDMTLEELKESPRLK

AHANRVIKVFDDSVQVLDDDSSQLEEIWVKVTQSHFNRQIEKQSFNELKEVILEVLTAAC

SLNDQQIEIWVKLMDFIYDIIFRTIDELEQGA*

>GfuGlob2

MIDSGIEYPKPLPPLNLENVCNEMGFTPLEIVALQNIWRLFKKRFKYHSMQIFLAFFNQN

HKLIERFRLPSGKFQLNYLCQHSEKMLLLYENVIDKCLDNMANFHGIMADVTVSHRHSGV

TYEDVSLKSEHVRRYILDYFANQSSPTLVSALAKLSEHFNDRHRVKEGDSKSDEFEEDD*

>GpaGlob1a

MNSDEVYEIKRTWEIPATTPTESGVAILIRFFTKYPSNLQKFSTFKDMTLDELKNNPRFK

AHANRIMKVFDDSIKTLDNNCSHLEEIWTKIAQSHFNRQIEKQSFNELKEVILEVLVAAC

NLNDQQTEIWLKLLDFVYEIIFKTIDQLEQDV*

>GpaGlob1b

MNSDEVSEIKKTWEIPAASPTESGVAILIQFFTKYPSNLEKFSTFKDIPLDELKESPRFK

AHANRVIKVFDDSVQALDDDPSQLEEIWVKVTQSHFNRQIEKQSFNELKEVILEVLTAAC

SLNDQQIEIWVKLMDFIYDIIFRTIDELEQAA*

>GpaGlob2

MIDSGIEYPKPLPTLNLENVCNEMGFTPLEIVALQNIWRLFKKRFKYHSMQIFLAFFNQN

HKLIERFRLPSGKFQLSHLCQHSEKMLLLHENVIDKCLDNMANFHGIMAEVTVRHRHYGV

KYEDINLKTEHVRRYILDYFANQSSPTLVSALAKLSEHLNDRHRPKEGDSKSDESEEDS*

>LcuGlob1

MNCDEVYEIKKTWEIPAATPTESGVAILLKFFTKYPSNLEKFYSFKDLPIDELKNNARFK

AHAVRIIKVFDESIQMLGHDWSGPKLEEMWSKVAVSHFNRQIEKQSFNELKEVILEVLTA

VCNLNEKQTAAWIKLMDIVYSIVFNTLDKLKNGEQ*

>LcuGlob2

MATNIIYPKPLPKLVKQNIPLETGFTSTEIVALQNGWHLIKRRLYYHSTKIFKDFFSEHY

LLLERFRNVEIGKFNLSNLHQHPGQLMNIYGRLIESGLNDVAFINMLLSDVGQRHKLYEV

TYDDVKLLTNHIRLYVIEFLDKIKSITFVNGLTKLSELINEHHREKAEDAEQTARTQSDD

ED*

>MscGlob1a

MELSDFEVIEIKNTWKIPMADPSGSGQAILLKFFERYPHNKLKFQDFKDQSLDQLKTCPK

FKAHASRIVRTFNEAINVLGTDYTDPALHEIFSKVAISHHKRGISKASYNELKEVILEIV

VAVCEMNDCQKCAWEKLMETIYELISRPLIALKSEVGFLNILKFVCN*

>MscGlob1b

MIELSDNDILEIKATWKIPMANPSESGEAILLKFFERYPSNLEKFKDFKEMTSDELKVCP

RFKAHASKIIRTFDEAINILGTDSTDSALQEIWSKVALSHHKRDISKSSYNELKEIIIEI

LVAVCKMNNDQKKAWEKLLICVYNVIFQTIDNISN*

>MscGlob1c

MDLELSEAEILEIQNTWKIPMADPLASGQAVYLKLFKRYPSNQLKFIDFKDVRYEDLKDS

PRFKFQALRLMRTFDKAINALGTENAGNILHEIFAKVAVSHHKLGITKVAHDQLKEVLIE

ILIEICGMNDFQKTAFEKLMEATFNVIYSENWLL*

>MscGlob1d

MDLDISEKEVLEIKRTWKIPMADPLTSGETMLLKLFERYPANQKKFQDLKDLPFKDLKDS

PKFKFHSVRIMKAFDEAIQSLGTQNAGMVLHEIFEKVAVSHHKRGISKESHNQLKEVIIE

TLVGVCDLNDFQKGAWEKVMESIFNVIYSENWIL*

>MscGlob2 Exon1

MDYPMEIEVLSSKNFIEETGFSAQEIVALTNCWRMLYAHRRKHSVDIAI

>MdoGlob1

MNTDEVLEIKRTWDIPAANPTESGSAILMLFFKRYPSNLQKFSAFKDLPLDELSTNARFR

AHASRIIKVFDESIQMLGHDWAGPKLEETWSKIATSHFNRQIEKKSFNELKEVILEVLTA

ACNLNEKQIQAWTKLMDTVYSIIFNSLDKLEKGEQ

>MdoGlob2

MSFHILYPKPLPPRDNQSLPNENGFTATEIASLRNGWRHFKRRFGYHSKQIFMKFYQEHE

QMLEKFRNRMGKFNMQQLHRHPQELLQVYGNLIEQGLDNMTYMHVLMTAISQRHRMFGVT

GYEIKLQTDHITLYILALLEKIISPTFVSGLEKLSRLINAYHCEDACDELLEESSNEALH

N

>GinGlob1

MNSEEVNDIKRTWEVVAAKMTEAGVEMLKRYFKKYPHNLNHFPWFKEIPFDDLPENARFK

THGTRILRQVDEGVKALSVDFGDKKFDDVWKKLAQTHHEKKVERRSYNELKDIIIEVVCS

CVKLNEKQVHAYHKFFDRAYDIAFAEMAKMG

>AgaGlob1

MSGPGSLVGSDEEEQTNYHTPDETGLTKSQKVALIAAWSIVKKDLVTHGRNIFVMFFEEY

PQYLDYFDFGGGSAGELGENRSLHAHALNVMNFIGTLIDYGLNDPALLKCSLGKLVRNHR

KRNVTKEDVAAVGGVIMRYSLKALEQHKTKTLEEAFGAFLGTVAAAFE

>AgaGlob2

MDQTGLTASEKITLFSAWGLIRKDLDVHGRNVLLLLFHKHPRYIAYFDFTDDPNAQSLVD

NKSLYDQAIHVFKAVGALIEYGFKDPVLFDATLRKITRRHKDRPVYTEDILTIGEVLLNY

LEQALGRQMSDSLPDAFWKLFQTIAGRFPATPKTPIADEGGDDANDPQPTTSQRQDRASD

AP

>AgaGbXL

MGCELTKLASSSNGGNSKSNNLPSLDACGPPPVDSRLPLTAKQKYTMVASWKGISRAMET

TGITMFIKLFEEHADLLNMFAKFKELKTKEEQATSEELQEHANKVMNTLDEGIRGLDDLD

TFFEFIHQVGASHRRIPGFKQEYFWRIEEPFLSAVSTTLGDRYTQNVEGIYKLTIKFIIE

TLVAGYEASANNNVDNTTSSSTPATKLSDEPNRAS

>AaeGlob1

MADTDLSTGITPDQRHVLVDAWKLVKPDVVTHGTNIFLKFFEKNPEYLGYFDFSMDYEAK

ELKDNRSLHAHALNVMNFFGAIIDYGLDHPIMYKSSLSKMVINHKRHGVSKPDVAIVCAI

IKDYCLQTLGHSDELEDAFTALLDSVANAFD

>AaeGlob2

MDESGLTGKQKITLLSAWGLLKQESSLHGRNMMFLLFREHPRYLPYFDFSSDSTNSNLAD

NKSFHLHAVNVMGAIGTLIECGLNDPEVFRKKLFHLVEVHKARGVTPLDVQLFSEIITDY

LVEVLGRQAANSLADALGKLFDQFAEAFAYQD

>AaeGbXL

MGCELTKLASSNGGSKGNNVPSLDACGPPPTDSRLPLTAKQKYTMVASWKGISRAMETTG

IHMFIKLFEEHAELLEMFANFKELKTKEEQATSEELQEHANKVMNTLDEGIRGLDDLDTF

FEFIHQVGASHRRLPGFKQEYFWRIEEPFLSAVSNTLGDRYTQNVEGIYKLTIKFIIETL

VAGYESSANNNEDVNNANLSTTGSAAGPTTSGDKKTS

>CquGlob1a

MFNSEHHEESVLNSPDDTGLTNHQKAALVGAWSLVKQDMVSHGVNVFIRLFEEHPKYLEY

FDFSQDDSAEELRENKSLHAHALNVMHLIGALIDYGLDNPLMFKCSLSKMMKNHKKHGVN

KEDVTIVCGIIMEYCLEALDQRGSTTLEEAFSSFMKSIADTFDE*

>CquGlob1b

MNEIYETEQGEEIVASIPDETGLNNHQKVALIGAWSLVKKDIISHGRNIFVRFFEEHPQY

LNYFDFSQDKTASEIGENKSLHAHALNVMHFIGTLIDYGLHNPLMFKCSLSKMMKNHKKH

GVHKKDVTIVCEVIMKYCLEVLDQHHSTTLEAAFKSLMRSIADTFDE*

>CquGlob2

MDETGLTGKQKITLLSAWGLIKQDLDLHGRNIMLLIFREHPHFIPYFDFSADPNNTSLSE

NRALQAHSLNLIMALGALIEYGLKTPKMFECTLAKLVKNHKTRRVTSQDVKMFGEVILMY

FAQVLGRQSASSLPTAFNRLIEQIAEAFEAAQFT*

>CquGlobXL

MGCELTKLASSNGGGSKGNNVPSLDACGPPPTDSRLPLTAKQKYTMVASWKGISRAMETT

GIDMFIXXXXXXXXXXXXXXXXXXXXXXXXXXXXXXXXXXXXXXXXXXXXXXXXXXXXXX

XXXXXXXXXXXXXXXXXXXXXXXRIEEPFLSAVENTLGDRYTQNVEGIYKLTIKFIIETL

VTGFESSANNNDDVNNANLGTRSSTAATAKATASGSEDKKTS*

>CriHb2B

MKFLVLALCIAAAVAAPLSADEASLVRGSWAQVKHSEVDILYYIFKANPDIMAKFPQFAG

KDLETLKGTGQFATHAGRIVGFVSEIVALMGNSANMPAMETLIKDMAANHKARGIPKAQF

NEFRASLVSYLQSKVSWNDSLGAAWTQGLDNVFNMMFSYL

>CriHb3A

MVATPAMPSMTDAQVAAVKGDWEKIKGSGVEILYFFLNKFPGNFPMFKKLGNDLAAAKGT

AEFKDQADKIIAFLQGVIEKLGSDMGGAKALLNQLGTSHKAMGITKDQFDQFRQALTELL

GNLGFGGNIGAWNATVDLMFHVIFNALDGTPV

>CriHb6

MKFLVLALCIAAASAAVLTTEQADLVKKTWSTVKFNEVDILYAVFKAYPDIMAKFPQFAG

KDLDSIKDSAAFATHATRIVSFLSEVISLAGSDANIPAIQNLAKELATSHKPRGVSKDQF

TEFRTALFTYLKAHINFDGPTETAWTLALDTTYAMLFSAMDS

>CriHbE

MKFIILALCVAAASALSGDQIGLVQSTYGKVKGDSVGILYAVFKADPTIQAAFPQFVGKD

LDAIKGGAEFSTHAGRIVGFLGGVIDDLPNIGKHVDALVATHKPRGVTHAQFNNFRAAFI

AYLKGHVDYTAAVEAAWGATFDAFFGAVFAKM

>CriGbXL

MGCELGKLASSSSSKKEIENGTVSEPAAPAPPDPRLPFTARQKYTMVASWKGISRAIETT

GVNMFIKLFEEHADLLNMFTKFKELKTKEEQATSEELAEHATKVMETMDESIRSLDEIDV

FFQFLHETGAIHTRIPGFTSDLFWKIEKPFLKAVSDTLGDRYTENVEGIYKITIKFVIET

LVEGFERGLQNKNHVGNKGVNSTCNSNVNGANTTTTTTTQNGDTSCNSTSRSNDAS

>LloGlob1

MPGLTPEQIEIVKSTWQLVAKAPEDAGEAILMRFFEKFPDN~QKYFPFRNVPRENLKGSV

MFRSHAGRVVAVFQKAVDAFNTADPVATLVEIWTEIARTHFRRQIKQKSFDELKEVVLEI

LTAACNLDEVQQTAWAVTLDTIFGIISKELANLAESSQ*

>LloGbXL Exon2

LFEEHGDLLSMFTKFKEMKTKEQQATSEELAEHANKVMETLDEGIRSLDDLDAFFEYLHQ

VGASHRRIPNFKADYFW

>PpaGlob1

MPSLTPEQIEIVKSTWVTVANAPEDSGEAILLRFFEKYPHNQKYFPFRNVPRENLKGSAM

FRSHAGRVIAVFQKSVDAFNTADPVGTLVEIWTEIAKTHFKRNITQTSFNELKEVILEVL

TAACKLDGVQQTAWAVTLDTIFEIISKELVKLGQTSQ*

>PpaGbXL

MGCQLGKLSGVSKGEDGQAFAPNEMPKQAKTDSRLPLTAKQKYSMLASWKGISRAMEPTG

VCMFIKLFEEHADLLNMFTKFKEMKTKEQQAKSEELAEHANKVMETLDEGIRSLDDLDAF

FEYLHQVGSSHRRIPNFKADYFWKIEKPFLSAVETTLGDRYTPNVEGIYKLTIKFIIETL

ITGFNKSANSAAPANSDTVNVNTKS*

>MdeGlob1

MASLTPHQIALIQSTWSIPAKVPIDSGEAILLAYFEKYPQNQQKFNAFKNTPLLSLKGTP

GFRTHAGRIITVLDEAISNLSKENYVEELERIWNQIGESHNRRKISRQSFNELRDILVHT

LIQVCSLDDEGKLAWNTLMDIIYHIVFLKLDENNQY*

>MdeGbXL

MGCELGKLAASTKSDGAYGQPDEPPPHDPRLPLTARQKYTVIASWKGIARALQPTGINMF

IELFEEHGELLGLFNKFRELKTKEEQASSEELAEHANKVMETLDEGIRALEDLNTFFPFL

HQVGGSHTRIPGFQAEYFWKIEQPFLSAVKTTLGDRYTENVSGIYIITIKFIIETLITGF

ESANSTQSNSNSDVNKINSKTDAMPAKS*

>BmoHbL

MGTWFSYMWWGGDPDVVNPVSGLTRREIHAVQKSWAPVNANSFATGSELLRRLFNTYPDT

KEYFKMVRKLPEEEYSQNPQFKAHVINLMTSLNLAVNNLNQPEIVAAMMTKLGESHRRRQ

IKEKNFHELKEVIVKLFIDVLRLDDATLSAWGKTVEFWYKHIFVTLNSPEETR

>BmoGbXLa

MGCQLGKLAASERRGNNAGPVTDGPPPATDPRLPLTAKQKYSMLASWKGISRAMEKTGIC

MFIKLFEENQDLLDMFEKFRQCKTKEEQINSMELAEHANNVMNTLDEGIKGLDDLDNFFQ

YIHQVGASHRKIPGFRVEYFWKIEAPFLAAVESTLGDRYTPNVENIYKITIKFILETLIE

GYEKAGNNPSSAS

>BmoGbXLb

MGCELTKMIKSEPHDLMNQPPPPSDPRCPLTTKQQYCMLASWKGIFRQIEKTGVLLFIKL

FEENEDLLHLFEKFQELRTTEDLSQSEELAEHANKVMHTLDEGIKGLGDIDTFLAYIQHV

GATHHQVPGFKAENFWKIEQPFLQAAKTTLGERYTANVENIYKLTIKFILENLVKGYEDS

AGKEIGNNETT

>ScyHbL

MGNWISQFWWGGDPDEVNPISGLTRREIYAVQKSWAPVFANSIPNGAELLRRLFQTFPET

KEFFKMIRKLPDEEYIQNPQFRAHVINLMTSLNLAVNNLNQPEVVAAMMNKLGESHKRRQ

IKERHFGDLKQVIVTMFIEVLHLDGDTLTAWDKTVTFWYKHIFETLNTAEESR

>AasHbL

MGHWLSQYWWGGDPDEANPVSGLTRREIYAVQKSWAPVFANTVANGTEFFRRLFQTSPET

KEFFKMIRQLPEEEYLQSPQFRAHAINLMTSLNLAVNNLHQPEIVAAMMNKLGESHKRRQ

IKKKHFGELKQVIVKIFIEVLNLDGDTLTAWDKTVTFWYKHIFETLNRTEESR

>AasGbXL

MGCQLTKLAASEFNHDLIDRPPPPADPRSPLTTKQQYCMLASWKGIFRQIETTGILLFIK

LFEENEDLLHLFDKFSELRTVEDQAQSEELAEHATKVMHTLDEGIKGLGDIDSFFAYVRH

VGATHHQVPGFKAENFWKIEQPFLQAAKTTLGDRYTPNIENIYKLTIRFILENLVXGYEE

SSGKENGSSQT

>BanHbL

MGSWLTYFWWGGDPDAVNPLTGLTRREVYAVQQSWAPVYANSVANGTELLKRLFRAYSET

KEFFKMVRKASEHEYADNPQFKAHVINLMSSLNLAVNNLNQPEMVAAMMSKLGESHGKRK

IQREHFYDLKDVLVKMFIEVLKLEGATLAAWGKAVEFWYKHIFGTLSQGDTR

>HvirHbL

MGGWLSYFWWGGDPDVVNPVSGLTRREISLVQKSWVPVNADAINTGAELLKRFFIAFPES

KDFFKMLKNTPEDQYLQNPQFKAHVINLMTSLNLAVENLNQPEVVAAMMNKLGESHGRRK

IQEKNFNQLKEVIVKMFIEVLKLDAVTLGAWGKTVEFWYKHIFETLNRAEQTR

>SfrHbL1

MGGLLSYIWWGGDPDEVNPISGLTRREVNLIQKSWAPVNADKAANGAELLRRFFTAYPAA

KEFFKMIKGMPEEQYLENPQFKAHVINLMTALNLAVENLNQPEVVAAMMNKLGESHGRRK

IKEQNFQDLKQVIVKMFIEVLKLDDTTLGAWGKAVDFWYKHIFETLNKAEQTR*

>SfrHbL2

MGVGDIVTRWWWGGDPDERNKVSGMSLRDVHNVQKSWAVIQANSNGNGFLMFFRLFEAEP

ETKLFFKNLAHIHTEAEMSANVSFRAHIINIMSSFDTSIQNLDKPELVVAWMQKLGDSHR

RHRIEKRHFHVFKDVLVTILQKDLKLDPQVVASWDRYVEFIYEHLLSRLAS*

>SfrGbXLa

MGCQLGKLAASERRGNNAGPAISDGPPPATDPRLPLTAKQKYSMLASWKGISRAMEKTGI

CMFIKLFEENQDLLNMFEKFRQCRTKEEQINSMELAEHANNVMNTLDEGIKGLDDLDNFF

GYIHQVGASHRRIPGFKVEYFWKIEAPFLAAVESTLGDRYTPNVENIYKITIKFILETLI

EGYEAAGKHPT*

>SfrGbXLb

MGCELSKLASSEFNNHDALDRPPPPADPRSPLTTKQQYCMLASWKGIFRQIEKTGILLFI

KLFEENEDLLHLFEKFGELKTAEAQMSSEELAEHATKVMHTLDEGIKGLGDIDAFFAYIR

HVGATHHQVPGFKAENFWKIEQPFLQAAKTTLADRYTPNIEEIYKKTIRFILENLVKGYE

DSAVENGNGQS*

>SliHbL1

MGALLTYIWWGGDPDVVNEISGLTRREVYLVQKSWAPVNADKVNNGAELLRRFFTAFPAS

KEFFKMIKNVPDDQYXTNPQFKAHVINLMTSLNLAVENMNQPEIVAAMMNKLGESHGRRK

IQEKNFLELKQVIVKMFIEVLKLDDTTLGAWGKTVDFWYKHLFETLNKAEQTR

>DplHbL1

MGGLLNRMWWGGDPDGVNPVSGLSRRDVFAVQKSWAIVYANPLANGSELLKRYFRAHPES

KEFFRMLRKLNENEFDDNHQFKAHVMSLMSSLNLAITNLDQPEIVVAMMNKLGESHGRRK

IDEQNFHNLKGIIVKMFIDVLKLDDKNLASWGKAVDFLYKHIFVTLKPES*

>DplHbL2

MGGIVSRIWWGGVPDAVQAYSGLSHRDIYTVQKTWAVVYCNAAENGIEIFKRLFHANPET

KNFFINFRNLSDEELDKSHQFRAHVINLMSSLNLAITNLHQPEVTAALMNKLGESHGKRG

IREEHLLSLKDVMLEMLNSLLGLDESALVSWNKTIDFIYKHIFQTLH*

>DplHbL3

MGSWLTYLWWGGDPDLVNPLSGLTKREVYAIQQSWAPVYSNSVANGTELLKRLFRAYPET

KEFFKMVRKSSEDEFIGNPQFRAHVINLMSSLNLAVGNLNQPEVVSAMMNKLGESHGRRK

IQEKHFHDLKDVIVKMFIEVLKLDGTTLNAWGKAVDFWYKHIFETLKQAEIR*

>DplGbXLa

MGCQLGKLAASERRGNNQDLGDGPPPATDPRLPLTAKQKYSMLASWKGISRAMEKTGICM

FIKLFEENQDLLNMFEKFRQYRTKEEQINSMELAEHANNVMNTLDEGIKGLDDLDNFFEY

IHQVGASHRRIPGFKVEYFWKIETPFLAAVESTLGDRYTPNVENIYKITIKFILQTLVEG

YEKAGKSNANTT*

>DplGbXLb

MGCKLSQLASSEFSHDPFEKPPPPSDPRSPLTAKQQYCMLASWKGVFRQVEKTGILLFVK

LFEENEELLHLFEKFRELRTKEAIVSSAELAEHATQVMHTLDEGIKGLADMDSFFTYVRH

VGGTHRQVPGFKAENFMKIEQPFLEAAKTTLGERYTPNIENIYKLTIRFILENLVKGYEE

AGEENGTTQT*

>HmeHbL1

MGGWMTHLIWGGDPDAVNPVSGLSKRDIYVVQKTWAVAATDSVGTGNELLKRYFRAYPET

KDFFRMIKNVPEEKFTENFQFKAHVINLMSALDLAVKNLHQPEVVAAMMAKLGESHGRRK

IQEKQFNELTIVIVQLFKDVLNLDDKTLAAWGRVVGFWYKHIFETLSGGENR

>HmeHbL2

MLYFYLCSTFLLLSAFIVLKRVKIGQKVTMGSWLSYLWWGGDPDAVNPKTGLSRREVYAV

QQSWAPVYASSVTNGTELLRRLFQAYPETKEFFKMIRKCSEEEYSQNPQFKAHVINLMGS

IDLAVTNLNEPDVVAAMMNKLGESHGRRKIQREHFYGLKDVIVKMFIEVLKLDGATLTAW

DKTVDFWYKHIFETLCLGDAR

>HmeGbXLa

MGCQLGKLAASERRVNNQGLGDGPPPATDPRLPLTAKQKYSMLASWKGISRAMEKTGICM

FIKLFEENQDLLNMFEKFRQYRTKEEQINSMELAEHANNVMNTLDEGIKGLDDLDNFFEY

IHQKIETPFLAAVEATLGDRYTPNVENIYKITIKFILQTLVDGYEKAAKTNSA

>HmeGbXLb

MGCKLSQLAASEFSHDPFDRPPPASDPRSPLTAKQQYCIMASWKGIFRQIEKTGIILFIK

LFEENEELMHLFEKFRELKTKEAIVSSAELVEHATKVMHTLDEGIKGLADMDSFFAYVRH

VGGTHRQVPGFKAENFLKIEQPFLEAAKTTLGDRYTPNIENIYKLTIRFILENLVKGYED

AGQENGTTET

>TcaHbL1

MGIITSTLSYYMTKTNDPDPITGLTSRDRYVIQTSWAPVKKDLTGNGVALLLLYFEKFPA

TKNYFVFKDVPNEKLKTDKKFHAHCNSVMVTLDSLIANLNDGELIVSLLEKLGKNHKRHG

IKDDAYDQLKETVIELFSSFMTKEELETWDKLLKVAFSVIIKYL

>TcaHbL2

MGQLISLYKYYTTRTDDPDPLTTLTSREVFLVQSSWDPIKKDLTGYGVQLLLFLFKKYPE

EQQNFPFRDMPFEELGASKKFHAHCSNVMYAVDSIIDSLKDGELLVNILEKIGRNHHRNT

VKPISFWHVKETMLEFFKKMMNDETLKAWDKALQVAFGVVAKELDKKN

>TcaGbXL

MGCELGKLASSGGSGNHRKPDESAPPATVDPRLPLTAKQKYNMLASWKGISRAMESTGVC

MFLKLFEEHAELLTLFEKFKELKTKEDQANSLELAEHASTVMNTLDEGIKELDNLDTFFE

YLHQVGASHRRIPGFKVEYFWKIEKPFLTAVETTLGDRYTENVENIYKITIKFIIETLVK

GYDNANAPT

>DpoHbL1

MGTVLSYITPANSGRSDDPDPVTGLTSKEKYLVRTSWAKIMKNPADSGVALLCLLFERHP

EYVQLFPFSDVPPSEFKTNVRFRAHANSVVYALSSIVDALNDNNLLVQILTKTGSSHVPR

HVTADAFIHLKEVTIELFSTIFKADEVAAWKKTFEVAFSVIIQGIESVN

>DpoHbL2

MGIITSYFGRELERDDDPDPKTGLTSRYISVLKTTWKRITERGGTLEIGTAIFTNLFEKH

PEYQQLFPFKNLRREELKTSNKFRAHCISVMYALTCIVENVSEPLILEQLLIKQSTSHVL

RNVPDQAYWDIKTVILSIVASSMNPSEVFVWEKFLKFAFRIMVATAEETRNN

>DpoGbXL

MGCELGKLASRDNRNGARKFEEPTPPQVQVDPRLPLTAKQKYNMMASWKGISRAMESTGV

CMFLKLFEEHSELLLLFEKFKSLKSKEDQATSLELAEHATTVMSTLDEGIKGLDDLDTFF

EYLNQVGASHRRIPGFKAEYFWXXEKPFLEAVETTLGDRYTSNVENIYKITIKFIIETLV

KGFENANAT*

>DviHbL

MGSVLSYFLGFFVNQGRINDANPVTGLTSRDIYLIKNSWNKVISQPTENGIKFFMRLFEI

APKHKLTFPFRDVPTEDLPRNKKFHAHVNSVMYSISSIVNSLNDVDTVAAIIDKIGRNHA

RRSVDLQALKDVKRALLDIFAFMTTAELAAWNKMLDYFAKTAIK

>DceHbL

MGGLLSYFYGQSNEPDPATGLTPREKSLVVNSWAIVRKDMIGNGTELFILFFTKFPKYLT

YFPFRDVPFDQLRENKKVHAHAVNVMYALSSIVDNLSNVEVLVNLLSKTGEAHGRRKIPE

QSFGDLKVTVLELLRVGLGSKITDEGIDAWNKTLEVAIKVIHDGMKEYYQKQQQKKAAAE

>MmoHbL partial

MGSVISGISRYGSNMDQADAVTGLTGREKLLVRSDWAQVMRNKNENGRTLFLIFFKRFPQ

NQSKFRFLANLTLDELADSKQLIAHGSNVLYSVTNIVENIDEPEIMVEVLKSIGQNHKKR

DIPLQSFD

>MmoGbXL

MGCKMSKSGSTSTASGSKSNNNRNGKVLEEPPPPQPPDPRLPLTAKQKYNMLASWKGISR

AMEPTGVLMFIKLFEEHQELLNMFDKFKQLKTKEEQASSMELVEHATKVMRTLDDSIKSL

DNLDAFFDYVHQVGFSHQRIPGYKSDFFLKIEKPFLEAARTTLGDRYTENIENIYQITIK

FILQTLIEGFEQSPNAVQDASSSSQAPS*

>AmeHbL

MGTFLRFLGISSSDDNRIDQATGLTERQKKLVQNTWAVVRKDEVASGIAVMTAFFKKYPE

YQRYFTAFMDTPLNELPANKRFQAHCAGVITALNNVIDFLHDPGLMEASLIGLVERHKKR

GQTKEEFQNLKEVMLEVLRQALGKQYTPEVAEAWNKTLDMMFGKIYQVFAS

>AmeGbXL

MGCELGKLATPNQPTTDPRLPLTAKQKFTVMASWKAVSRKLETTGVFMLMRLFEENEELV

QMFSRFLDLKSKEERFDMVELGKHAEKVMGALDEGIRGLDNMDDFLTCLHQVGATHTKIP

DFNPQYFWKIEQPFLEAVKRTLEDRYSENVESTYKVTIKFIIETLIDGFDKAQNDKAQTS

TAKS

>BteHbL1

MGTFLRFFGFSSSDDNRIDEATGLTEKQKKLVQNTWAVIRKDEVASGIAVMTTFFKTYPE

YQRYFSAFADVPFDELPANKRFQAHCVSVITALNSVIDSLHDPGLMEASLISLGERHKRR

GQTKEEFENLKGVVLKVLSQALGKQYTPEVAEAWSKTLDGVFAKIYQVFSS

>BteHbL2

MGSVLTYFLGNPDDDVVDPKLGLTNKEKRIIRETWGVLRANSVKVGVDIMISYFKRFPQH

HRAFPPFKDIPADDLLDNKKFHAHCQGIMSTLNDAIDALDDVDLMNAILHTTGKRHGRRG

QGRQEFIDLKGVVLDAMRGAFGSKFTTEVEVAWDKAIDVLFSKIFEGEDMI

>BteGbXL

MGCELSKLATPNQPTTDPRLPLTAKQKFTVMASWKAVSRKLETTGVFMLMRLFEENEELV

QMFSRFLDLKSKEERFDMVELGKHAEKVMAALDEGIRGLDNMDDFLTCLHQVGATHTKIP

DFNPQYFWKIEQPFLEAVKRTLEDRYSENVESTYKVTIKFIIETLIDGFDKAQNDKAQSG

TAKS

>NviHbL

MGSSGSLFWGSANDNVLNPATGLTGRQKKLVQNMWAIVRKEPIPNGVAIMLAYFKKYPEY

QKVFTHFKDVPLEELSANKKFQAHCLNIVTALNNLIDSINDPALLEANLVAIGERHHRRG

QTKEQFLHLKEVIAEVLRQKLGAKFTAETAEAWNKTIDAAYTGIFQTFST

>NviGbXL

MGCKLGKLASSTTSQIQNARSGRESPPPPPATDPRLPLTARQKYLLTASWKAIAKAMEPT

GIYMFVKLFEENAELLNMFSKFKNLTAQEEQSKSVELAEHAEKVMNTLDEGIQGLDNMDA

FLTFIHQIGATHTKIPGFDREYFWKIESPFLAAMQMTLHDRYTENVENIYKLTIKFIIQT

LIDGYDGAKSEKSAYYSTPFENKEKS

>CflHbL

MAWFRGLFNFFLDDNKLDEKIGMTEKQKRLVQNTWAIARKDEVSAGVAIMIALFKQYPEY

QKQFKPFKDVPIDELPKNKRFQAHCVNIISAISKLIEQMCDPELMQATLINLIEKHKNRG

QTQEQFENLRQLLAKLFPSLFGKQYTQEAEEAWKKLLDLMYSVIHEVYKN

>CflGbXL

MGCELSKLATTKSRNQTGNDGSSPPPPAATDPRLPLTARQKFTVIASWKAVSRALEPTGV

YMFIRLFEENAELLNMFTKFRDLKTKEQQSTSMELAEHAKTVMSTLDEGIKSLDDMDAFL

TYLHEVGASHTKIPGFNRQYFWKIEKPFLDAVERTLEDRYSENVENIYKLTIKFIIETLI

DGFDKAQNDKAKS

>SinHbL

MALFRGLFHFFLNDNKVDEKLGLTEKQKRLVQNTWAIVRKDEVSIGVALVLAYFKQYPEA

QKEFKAFKDVPIDELSKNKRFQAHCANIVATIGKVIEQMHDPELMEASVINFTEKHKNRG

QTQKQFENLKQMMLDVFPSVFGKQYTPEVQEAWKKMLGLIYSKIYQTLKD

>SinGbXL

MGCELSKLASSKSRNQAGNDGSSPPPPPAATDPRLPLTARQKFTVIASWKAVSRALEPTG

IYMFIRLFEENAELLNMFTKFRDQKTKEQQSTSMELAEHAKTVMSTLDEGIKSLDDMDAF

LTYLHEVGASHTKIPGFNRQYFWKIEKPFLDAVERTLEDRYSENVENIYKLTIKFIIETL

IDGFDKAQSDKAKS*

>PhuHbL1

MGLGSSKSEPLTADELERVQNSWKVVMENAEENGMFIFKTFLLKHNYFPYFKAFANTPLE

ELEENQAFRNHANNIIQALDNVILNLEDELTIQRELTALGKMHGKKKISEQQFQELKICI

LEILDNEFKLPEDDLQAWSKTLNNAFVFVFEGLAAEV

>PhuHbL2

MNVVLNDWPKIRKNYKKIFIDSFINYFAENPNYKLLFPSFSNVSEDDLPFNHCFRLHCFA

VYKAINFLMSNWLGEYEEDDSKILPVIGKTHFDRGITLEMMNLYKHSIVYSCNNHLKPNL

KRKLSWQTVFDHIFDYYLGSAYPAPIQTVEEDD*

>PhuHbL3

MSKNIKRDINQENLSLAVKIVTPTWESIKEDFDWYCTKIEETFFQNDTTKKELFTLPKFE

EELTDDVVNKRLFKHSSAVLNFMECIVQFMNGNEETKPVLFVLGRNHYTIGVNEKLFLEM

KDAICSVIKYKIGTENAKAWDTILQYILINYVFEGMTM*

>PhuGbX

MGSSVAHHKNHSNSIDGNSTGTEQVQSSSSNQTPIKVKPSNNQKQRRKTFSSLGIRGSFD

FRGSSLSFRSRGSFDVGSSKPDQCSPVLEEQRPPELTTREKELLIETWKELEENIAKVGV

ITFVSLFETHPDVQESFMSFSGVDIEDLKHSKQLRAHALRVMAFVQKAVARLHEPEKLET

LLKELGRKHVGYGAKQKYVELVGPQFILAIKPSLEKQWDEELDDAWTHLFKIIEFVMVSS

MDDDRKDQRTLERYIIYI*

>PhuGbXL

MGCAKSTQNGGRGGGGGSGDLKGDEKPPPQTDPRLPLTAKQKYNLVASWKGISRAMEPTG

ITMFIKLFEQNEELLNLFEKFRELKTKEQQASSMELQEHAMNVMKTVDEAIRELDNLDGF

FIYLHQVGSSHRKIPGFKPDYFLKIEQPFLQAVKDTLGDRYTENVERIYNITIKLIITTL

MEGYTDYGKS

>ApiHbL

MASSLSPLQISQLKDSWSVLAQDPSQLASALVIRLFKENPEYQSLFKRLKNLSIDELASN

PQFMSHASKVGAALASTIDHLDKPEELEKLLTNLGIKHKKYGLSAKHFQVIGDVLIAMIT

EAIGDSEPELLDLWKSSLTSVLSIIIAACH

>ApiGbX

MGNAGTTRRGSIFSKQDSTGDDGSTRHNKRLSSRQNTFLEEEEFPPEPPVLPPPEPLTMR

QKELLTEMWKLLEEDIAKVGVITFVSLFETHPDVQQSFMPFKGVDLEDLKHSRQLRDHAL

RVMAFVQKAVARLYEPDKLETLLRDLGKKHYHYGAKQKYVDLIGPQFIMAIQPSLVDRWT

EEMHSAWTALFLNMAYIMKGSMAAEERFKVKKTAT

>ApiGbXL

MGCDLGKLASSATSGDDRGGKHKDVLEEPPSPGPPDPRLPLTAKQKYSMIASWKGISRAM

EPTGVYMFIKLFEEHQELLQLFTKFGELKTRDAQANSMELAEHANKVMTTLDEGIKELDD

LDNFFQYLTQVGATHKTIPGFNPDYFWKIEVPFLEAVKTTLGDRFTENIETIYKITIKLI

IETLVKGYTEAAGP

>AgoHbL

LASNPQFISHASKVGAALGSTIDHLDKPEELETILTNLGIKHKKYGLTPKHFQVIGNVLV

SMIAEAIGDSDPELLDLWKSSLSSVLNIVIAACNN

>AgoGbX

MGNAGTTRRGSIFSKQDSTGDDGSTRHNKRLSSRQNTFLEEEEFPPEPPALPPPEPLTMR

QKELLTEMWKLLEEDIAKVGVITFVSLFETHPDVQQSFMPFKGVDLEDLKHSRQLRDHAL

RVMAFVQKAVARLYEPDKLETLLRDLGKKHYHYGAKQKYVDLIGPQFIMAIQPSLVDRWT

EEMHSAWTALFLNMAYIMKGSMAAEERFKVKKTAT

>AgoGbXL

MGCDLGKLASSATSGDDRGGKHKDALQEPPSPGPPDPRLPLTAKQKYSMIASWKGISRAM

EPTGVYMFIKLFEEHQELLQLFTKFGELKTRDAQANSMELAEHANKVMTTLDEGIKELDD

LDNFFQYLTQVGATHKTIPGFDPDYFWKIEVPFLEAVKTTLGDRFTENIETIYKITIKLI

IETLIKGYTEAAGP

>BtaHbL

MPDMDEGLSQTEIDLIRESWQPFAKDLQETGITFFLAFFKRQADYQEAFPFRGVPLSELR

QNESFRRHAKAVLQFIDTAIASLENTSEILSMLESNGKSHGRKNLGLTWSHYEHLEFTLL

DVIDEFYEQEKRPLSSLEKETWAKFIRSVTSGIYKTVQIHQNAIQS

>BtaGbX

MGNTGTTRRNSLFTKQDSSSSEVGSTPGGGGGRKMTITSRQSTLKEEENELGPPPKPLTD

AQKSMLVDTWKALENDIAKVGVITFISLFETHPDVQQVFMPFNGIELEDLKHSKQLRAHA

LRVMAFVQKAIARINEPEKLDTLLKDLGRKHYSYGAKVKYVDLIGPQFIQAIQPSLKDRW

NEELHQAWACLFQFMAYIMKNAMLQEEAAQKS

>BtaGbXL

ELRTREAQQNSMELAEHATKVMTTLDEGIKELDNLDSFFQYLTQVGASHKKIPGFKPEYF

WKIEKPFLEAVKMTLGD

>MpeHbL

MASSLSPLQISQLKDSWSVLAQDPSQLASALVIRLFKENPEYQSLFKRLKNLSIDELASN

PQFMSHASKVGAALGLTIDHLDKPEELEKILTNLGIKHKKYGLTAKHFQVIGDVLVAMIS

EAIGNSEPELLDLWKSSLTSVLSIIIAACQ

>MhiHbL

MTDSKKIFADEVIKDVKTTWATINSDLQQVGYEIFNRLFNAFPTYQQLFRAFKDVPFGEL

QSNKDYSKHALAVAKALNASIENLENPEQLVSILTTVGKNHVKRNVTPEHYSNAQKIILE

VIGTKLGDENSDKILSSWNEVLAVAVSTIMKGAQEEEAKYI

>NluHbL

MSGLQILVLRSLFGRISLQNNLPAQTVAQRLFSADCPPKDCCPPPEPSPKDLANVKEAWC

EIDRNKGCYAKAIFTEVFKKYPDYAQLFAKFGRCPTDILKNEKFSEHLKKNVMDEMGNVI

KKMGEDMCEAKSMASDIGKKHVKLCVKPKHFENTEKIFIDVLKKQMGEKLSCDGAKSLEN

VIKSVFKQLKKGAAADSCSS

>NluGbX partial

MGNPATARRNSVLTKQESSSSDGNHGRITSSSRQNTITVTEEEPPQPVSAASSEATASTP

PPSSDPPPQPLTAAQKQILQETWRGVEDDIAKVGVITFIXXXXXXXXXXXXXXXXXXXXX

XXXXXXXXXXXXXXXVMAFVQKAVARLDEPEKLDSLLRELGKKHFTYGAKHKYIDLIGPQ

FIQAIRPSLEQQWTAETAAAWTALFAHMGAIMKAEMVGAEEEANRAKAAQ*

>NluGbXL

MGCELGKLALAQRGGSNGGGDSDVGMQEGGGGQRGGAPAKPDPRLPLTAKQKYSMLASWK

GISRAMEPTGVYMFIKLFEEHRELLELFTKFRELRTRDEQASSMELAEHANKVMTTLDEG

IKELDNLDTFFEYLTNVGASHKKIPGFKPEYFWRIEKPFLEAVKMTLGDRYTENVENIYQ

ITIKLIIETLEKGYNT*

>DciHbL

MTTYEQLVSNISDDRLQAVVLSLDIIKPDINDFGTKVFKTLFKEHPEYQSQFPKLKDIPY

DKLDANKSFTHHVNAVVLAIANSVVNLKNPNAVLPELEKLGTSHQRRNIRPEQFEVVTNI

ILKVLKEKVSDPQVLKTWQEILTILANTIVSFMKPTIK

>DciGbX

MGNTGTTRRNSLFLKQESGDEPPSHKKEGVTHSIARRLTLSGGKGLGLGSRQNTLLEEEI

VPPCVNSPPPEPLTEDEKKLLIETWKILEDDIAKVGVITFISLFETHPDVQQSFMPFNNI

ELEDLKHSKQLRAHALRVMAFVQKAIARLHEPDKLDTLLRDLGKKHYTYGAKAKYVDLIG

PQFISAIQPSLESRWSPELNNAWIHLFGYMAHIMKESMTAEELLNRKS*

>BmaHbL

MPKAFSMTDREVEVINQSWNQIKAQELVVGLQMFKLLFQRYPQYERLFTHLHQSGKSLYE

GDRFQHHVVRNIMSSINKVIEQLNSADSAPRTLQEMGVRHKKLDVHRKHFESFVPFVVDA

MVNVRMSMDKDEVASAWTKLMDAIASNLSKGVES

>CleHbL partial

MFEKFPSYEDFFKKHKESNKSLLDSTTLQPHIRLVMKTLDYLVVNLNDMSVVEERLVKLG

QTHKGRNVKSPQFNHIVEIVLETLRRALGPKLTPGLEASWKAVLNCAMTIAGNEADK*

>CleGbX

MGNAAPGRRNSMLSKEGSVNEASRQNTLTDADQQGPQPRALTKEEMDHLTRTWKLLEDDI

AKVGVITFISLFETHPDVQQVFMPFNGIELEDLKHSKQLRAHALRVMAFVQKAIARLHEP

EKLEQLLKELGKKHHGYKAKVQYVDLVGPQFIQAIQPSLDSEWTEEVADAWKLLFAHVGY

IMKGAMIEAAEEAAKESK*

>CleGbXL

MGCELGKLTRISGSGNSEGREPPPSAPAPSDPRLPLTAKQKYSMVASWKGISRAMEQTGV

FMFIKLFEEHQELLDLFAKLKELRTKEEQEKSLELAEHATKVMATLDEGIKELDDLDTFF

TFLTQIGQTHKKIPGFKPDYFWKIEKPFLEAVKMTLGDRYSENVESIYKVTIKLIIDTLV

KGYNS*

>HhaHbL Exon2

FFGDYPSTIKYFKRFGNDPETIMKHATFIDHAKKNVFNSLDKTIGILDEPEEIKKIELWI

GKVHRSKRISREDFL

>HhaGbX

MGNVAAGRRGSVLSKESSITDSKPSRQNTLAEDSPPEPLSDLQKAELTRTWKLLEEDIAK

VGVITFISLFETHPDVQQVFMPFSGIELEDLKHSKQLRAHALRVMAFVQKCISRLNEPEK

LEQLLRELGKKHHSYKAKAKYVDLVGPQFIQAIQPSLGEEWNEEVSEAWILLFAHINYTM

KGAMNEAAEEAKMKQ*

>HhaGbXL

MGCELGKLTKSGAEQASPPKQDPRLPLTAKQKYSMLASWKGISRAMEQTGVYMFIKLFEE

HEELLELFAKLKELRTKEEQASSLELQEHATKVMNTLDEGIKELDDLDTFFTFLTQIGQS

HKKIPGFKPDYFWKIEKPFLEAVEMTLGDRYTENVENIYKVTIKLIIDTLVNGYNT*

>HvitHbL1 partial E23

LFTTYPDYVKYFERMKSSPDQDIFESARFKKHMVSALFPSIALMLKNLDHPSELSSQLHE

IAVKHKRRGLRRSHFEVLQEVIMTTLKKAFGEAFTADTESAWNKILTVAFDGISTTIEKT

EL*

>HvitHbL2

MAKLKLNCGQSLPLEAITDRDKELAREAWVQVEFNYVLISKNLFVDWFTQYPEHVNFFKH

MMDSSCDDIFTSPKFARHMANSLLPNLGIIIRNLDRPNDFRSHILKVAWSHVERNLDLNS

DHLDILKGLILRTLKDSLGGGIGLDHEVALFKIITAAFKIFGEVLENRVNE*

>HvitHbL3

MASGRRTSDNLRNSRPDGRRQPGAQGVPPLRRSAHERPKDLASLTDRDLRLGRATWFKNV

DATPDFGMVIFKELFRQYPEVESYFLHLRGNSGSIFDSRTFRSHMTERVVPKLKEVFEAL

DKPEHLNEVMTKLGLYHAKLGVSGHLVENMLSVILDALKSVMPTKMQPDEETAVRTCLKS

AFAIAIDTINIYEKENKQAATDS*

>HvitHbL4 Exon2

LFDDYPYFKKFFKSAIGNFDDPFMSPRFQKHMLQVLLPTFGGIMDNLDFPEAVNEAVKRL

AVAHRRKEIGLAKEHID

>HvitGbX partial

MGNTAQGRRNSMLPKQESSSSDTTAAKGGKNSRQNTLQEEEDCVTELQGGGGGGNDGGES

EALTERQKELLTESWKVVEGDIAKVGVITFIXXXXXXXXXXXXXXXXXXXXXXXXXXXXX

XXXXXXXVMAFVQKAVARLNEPEKLTTLLRELGKKHYGYGAKQKYVDLIGPQFILAIKPS

LEAQWSDETHAAWTALFAFMGSIMKDAMSAEEAAQKAQ*

>HvitGbXL

MGCELGKLAQMQRGGSEGGPNRGADDPPAPAPPDPRLPLTAKQKYSMLASWKGISRAMEP

TGVYMFIKLFEEHEELLDLFTRFRELKTRDAQANSMELQEHATKVMSTLDEGIKELDDLD

SFFEYLHQIGASHRKIPGFKPDYFWKIEKPFLEAVKMTLGDRYTDNVENIYKITIKLIIE

TLEKGYKGS*

>OfaHbL Exon2

LFSNYPYMLEFFKNYGETKEDILNNKKFMFHAKERVFKTFDKTVNNLGNEAELNNIASWL

AEVHVSRGIKPPDFS

>OfaGbX

MGNVSAVRRGSVLSKEGSIADSKGGSRQNTVAEEPTPEPLSDQQRAHLTRTWKLLEDDIA

KVGVITFISLFETHPDVQQVFMPFSGIELEDLKHSKQLRAHALRVMAFVQKCISRLHEPE

KLEQLLRELGKKHYGYKAKAKYVDLVGPQFIQAIQPSLGEEWTDEVSAAWVLLFANISYI

MKNAMAEAAEEAAREKQ*

>OfaGbXL

MGCELGKLTKSAGSPVEAARPPPPQQQSDPRLPLTAKQKYNMLASWKGISRAMEQTGVYM

FIKLFEEHEELLGLFEKLKQLRTKEEQAQSLELQEHATKVMHTLDEGIKALDQLDNFFAF

LTGIGQSHKKIPGFKPEYFWKIEQPFLEAVEKTLDDRYTENVENIYKVTIKLIIETLVNG

YNS*

>PveHbL partial

SYEELVKKIPDKDLEAAVDSLTILQNDLQGFALTIFKTLFKEHPDYQKQFPKFDGIPFDK

LEENKSFQNHALAVVSAIATSISHLRNSNAVLADLQKLGEAHVKRQIRPEQFEAVINIIL

KVLSERISDAQVVQTWKDVLHILATTIISFMKPE*

>PveGbX

MGNAGTTRRNSLFLKQESGEEGHKKEGVTHSIARRLTLSGGKGLGLGSRQNTLIEEESVP

PCVNNPPPDPLTDEEKKMLSETWKVLEDDIAKVGVITFISLFETHPDVQQVFMPFSGIEL

EDLKHSKQLRAHALRVMAFVQKAIARLYEPDKLDTLLRDLGKKHFTYGAKAKYVDLIGPQ

FISAIQPALESRWSTELHNAWVHLFAYMAHIMKESMAAEELCHKKS*

>RprHbL1 Exon2

LFELHPDFEKYFARFKSEGAKSLFDNPMFLFHVKHKVMDSLNEVIDNLENDERLLKILKS

VASNHKKRNIKKEEFV

>RprHbL2 Exon2

LFKANPEELLKHNHGQILEELFMDQTNLDYMDKLAEIFSIVVQNIDKSTLCTKLIWELAM

YHRCLDLTESYFQ

>RprGbX partial C-term uncertain

MGNTVAGRRNSVLSKESSLSEAFPPGLGGKTSRQNTLVETAEEAAAAQGPLPPALSDHQM

ELLTKTWKLLEDDIAKVGVITFISLFETHPDVQQVFMPFNGIELEDLKHSKQLRAHALRV

MAFVQKAVARLHEPEKLDQLLKELGKKHYGYKAKAKYVDLVGPQFIQAIQPSLQNEWSPE

VADAWKLLFCHISYIMKGSMAEAAAEDAAKXXXXXXXXKKFNKILCNVSEEDCCPMNDSL

R*

>RprGbXL

MGCELGKLTKGGGNSRDAGDAREPPPQPPAPTDPRLPLTAKQKYSMMASWKGISRAMQPT

GVYMFIKLFEEHEELLGLFAKLKELRTKEEQAESVELQEHATKVMSTLDEGIKELDDLDT

FFSFLTQIGQSHRKIPGFKPDYFWKIEKPFLEAVKMTLGDRYTENVENIYKVTIKLIIET

LEKGYNT*

>FocHbL

MGAILSYLWSPSLSTEVDPATGLSPRDKHLVRTTWAIVKKDASSNGLYLFQLLFTKHTDV

RDMFPFARGKEAAEYRDDPRMRAHANAVMYALTSYIDQLDDVPCLDAMVRKLADSHLKRH

VTPEHFKALGAVVMQALQDLLGASVMTPDAVTAWTRTYGLVLQVVTDQMGKAQS*

>FocGbX Nterm uncertain

MSLAEPGARSSGRSSVGVAGRLSLIPGRGRQASLVEALGCVAQPEEDEEDTEPPPDLTEH

EKELLTTSWKEIENNVAHVGVITFISLFETHPDVQEVFMPFNGIELEDLKHSKQLRAHAL

RVMAFVQKAVARLNEPEKLQTLVRDLGRKHVGYGAKRRYVDLIGPQFIQAIRPSLESQWS

DEMHEAWSRLFQHLSFVMKGSMLEEERRVKTAAKGR*

>FocGbXL

MGCELGKLAASERGGGRGGGGGDGGGGKDDIPAPPATDPRLPLTAKQKYTMLASWKGINR

EMEATGVNMFIKLFEEHKELLNLFEKFIALQTREEQASSEELQEHATKVMNTLDEGIRGL

DDMDAFFSYLGQVGASHRRINGFQSQYFWKIEGPFLKAVEQTLGDRYTANVENIYKVTIK

LIIQTLVDGFEGTGPAAGSAGS*

>LkoHbL

MGSVLSYLWSGSAANAAIECDVPDPDTGLTPRQKKFVSDTWQLVKRDIKGNGIELFIRFF

EMRPEGQNRFSSFVGMPLNELRHSKRLQAHTNSVMYALDGVVMPLDDPEVMHEMLLKIGM

NHGRRGITEEEFHELKIVLMNLLKEKLDIHVNSDGEEAWSKTIDVFYKSMFKGMDMTSH

>LmiHbL1

MGALLSFLWGGGGATPALPEVADPVTGLTPREKHFVVTTWAAVRKDITSNGVQLFLRFFD

KLPAAQKRFSSFADLPRDELAASKRLKAHANSVMYSIDSIVCNLDDPEVLEEMLLKIGNN

HGRRKIPEDEFMVLKDVLMQLLRDILEIHKSPVGEQAWSKAIDVMYKNIFKGMEETRNK*

>LmiHbL2 partial

MGGLMSFLWGGPAAAAAADDDDPALDVPDPVLGVTPRQKRMVADCWGVVRQDAKGNGTEL

LLLFFTNNPESKKRFASFKDVPISDLRTNPKFHAHANSVMYALTAVVDHLDDPEVMIEML

RKQGENHGRRKITEGEFL

>LmiGbX partial

PEISDQQKQLLTDAWKTLEEDIASVGVITFISLFETHPDVQQVFMPFNGIAIEDLKHSKQ

LRAHALRVMAFVQKAVARLYEPEEKLLTLLQDLGRKHVSYGAKQEYVDLIGPQFIQAIKP

SLESQWSDDLQGAWERLFEYIAFHMKAAMERERAEKRR*

>LmiGbXL partial

RIPTARQKYNMLASWKGISRAMEPTGVYMFIRLFEEHAELLELFERFRSLRTREEQAGSM

ELAEHATTVMNSLDEAIRALDDADAFFAFVEQVGASHRRIPGFQPELFWKIEKPFLEAVK

TTLGDRYTDNVDTLYRATIKLIIETLINGYNNAQDNPQAQTKQPSS*

>RflHbL

MSGQTDESNLDTPGPSTGLTPRERQIVVDTWGVVKRNAKEAGVEMFTRLFEAHPQYQKLF

PNFEGLTLSVLRTSKKLAAHATNVMYSLTSVIDNLDDPECLKELLIKLGKNHGRHKVYEK

QFHDLELVLMELLKEKLGNQLTPQGEVAWKKTIDIVYKGIFQGMRAYDASSVK

>ZneHbL1

MGSVLTYFWGREPNDDPELDIPHPTTGLTPRERQAVVDTWAIMKQDAKRAGVELFIQLFE

AHPEYQKLFRVFESLSLQELEKSAKLSAHATNVMYSLTSVIDNLEDPECLTELLIKLGQN

HDRHGVSEKEFNDLKVVLMKLLKQKLGKKLTSQAEAAWSKTIDVAYQVIFEGLKTSDVAS

VK*

>ZneHbL2

MGSLLSSLVGGNSNPALDIPDPDTGLTQREKMAVRRNWDIVKANIKQNGIELLIMFFEAN

PSHQRYFKSFRDVPLKDLPSNNRFQAHCTSVMYALTSIVDNLDDTGCLVEMLTKLGQNHR

KHGISRQEFNDLKITLLKLLKKKLGSKFTCEDEAAWKKMLDVAFSVIFTGMDEDQGSES*

>ZneGlobX

MGNASSHHKVGGIGGVSAGSEKTVRGDVTASGNEQAKEGDTKPGSKVAANNHQDHQQHRK

RSSVIGGGEREGDIAVVRPEELTERQKELLEETWKELEGNIAKVGVITFISLFETHPDVQ

QVFMPFNGIELEDLKHSKQLRAHALRVMAFVQKAVARLHEPEKLETILQELGKKHYTYGA

KQKYVDLIGPQFIQAIQPSLEDRWTPELQEAWIHLFKYMAYVMKTSMGEEEQRINSQQ*

>ZneGlobXL

MGCELGKLVMVHRDAGEGSKGRHGEAGPPPPPEPPDPRLPLTAKQKYSMMASWKGISRAM

EPTGVYMFIKLFEEHQELLNLFEKFRELRTREEQANSMELAEHANTVMTTLDEGIKGLDN

LDSFFDFLTQVGASHHRIPGFKPEYFWRIERPFLEAVQMTLGDRYTENIESIYKITIKFI

IETLVRGYEEKKPDT*

>BgeHbL1

MGGILSYFLGSKDDPSMDIPDKATGLTPRERQIVKDTWALAYKNSKSVGVELFIQLFTTY

PHHQQKFPSFKNVPLSEMKLGNKKLEAHATNVMYSLATLVDNLEDVECLIELCSKIGENH

LRRKVEQQAFLDVKTVLMKLLKEKLGSSLTPQGEEAWNKTLDLANKCIFQAMEDKKNKA

>BgeHbL2

MGNLLNALSGSGSNANLDVPDPATGLTPREKNAIRRNWELVKGDIKQNGIDLLMLFFEEN

PSYQQFFNSFKDVPLKELPKNPKFHAHCTSVMYALSSVVDNLDDPGCLVEMLSKLGENHH

RRGISRQEFINLKAVVLKLLKTKLGSKFTSEDEAAWNKTLDVAYSVIFKGLDKAEEDASQ

KA

>BgeGbX

MGNASSHHKAGGVAEKVPGGDLGKNGAEFEEQEQMRKRSSVGVESDRDVIPLVCPEPLSE

RQKELLVETWKELEQNIAQVGVITFISLFETHPDVQQVFMPFKEIELEELKHSKQLRAHA

LRVMAFVQKAVARLHEPEKLEKLLQDLGKKHYAYGAKQKYVDLIGPQFIQAIQPSLAEQW

TPELNTAWVQLFQYMAFVMKTSMNEEEQRVRAQQ*

>BgeGbXL

MGCELGKLVAAPRGEEEPPAPPQADPRLPLTAKQKYTMMASWKGISRAMEPTGVYMFIKL

FEEHGELLNLFEKFRELRTREEQANSMELQEHATTVMTTLDEGIRSLDNLDSFFQYLTQV

GASHHRIPGFKPEYFWKIEKPFLEAVKMTLEDRYTENVENIYKVTIKFIIETLVRGYEEN

KPAS*

>LfuHbL1 partial

FFDAHPQYQKLFRVFGNLPRSELPSNKRFLAHATTVVYSLMSVIDNLNDKNECLVEMLFR

IGQNHGRVNVPPESFAHLKVVILELLQRRLKDRFTPFAEESWSKTLDVANSVFLKGLEDG

QKEKIVLKTENAS*

>LfuHbL2

MGLRHSTAVDNTPDPVTGLTPAQIQAVRSTFDVLRSDPKDFGVDLFLSLFDAHPNYQKLF

RVFGNVPRSELPGNKRFLAHASTVVYSLMSVIDNLND~SECLVEMLVRIGQNHGRMNVQP

ESFEHLKSVIMGLLKRRLKDRFTPFAEESWSKTLDVANSVIFKGLEDGQKEKIVLKSENA

S*

>LfuHbL3

MGGILSLLYRSGDGPEEMDGPMVDVPDEATGLTPRQKRAVAVTWDIVKKDLKGNGVELLH

RFFTKHPQYQKNFKAFADVPLDELPNSKKFQAHANSVVYAVTSIVDNLDDPGCLVEMLRK

LGQNHGQRHIPEQAFLDLKAVLMKMLKEKLEGHFTAYEEESWDKTMDTAFSVIFQGLKDQ

ELTAT*

>LfuHbL4 partial Exon2

LFEAHPEHVKSYPAYAGVPLSELATNKRFMALTATVVYNIMSVIDNLDDKEVLVEILLRL

GRTHARINVGPDAFE

>LfuGbXa

MGNSAHSRMSKEGSDGSPKSLRQKSLSCAESTEEPKAANDATAAAENGPKSKSVVMELNV

KPEPLTEEQMGLLQENWKELEDNIAKVGVITFISLFETHPDVQEVFLPFQGMELEELRHS

KQLKAHALRVMAFVQKAVARLHEPDKLDVLLRELGKKHYTYGAKQEYIDYIAPQFIQAIK

PSMEDKWTKELEGAWTALFNYMGVIMKAAMDLEERRAAAESLPPYNVPLPTTQQSCSALP

PTQPFAPSKGLLHGSRRASSDVGGDGVSAAGGTSLLASKRRGTLY*

>LfuGbXb

MGNSVHSRNAKDNGQESASKAAPPKPVDDGNDAGFDKNSESALKHRAFEMELDVKPEPLT

EKQMAMLQENWKELEDNIAKVGVITFISLFETHPDVQDVFIPFKGMGLDELRHSKQLKAH

ALRVMAFVQKAVARLYEPEKLDLLLRELGKKHYSYGAKQQYIDYIAPQFIQAIKPSLEDR

WTGELQDAWIALFNYMGVIMKAAMDWEEKRAAAATIPYALPPSSHPSKTGAPAPVNPTAP

PVTPAPPTNPTPPINPPPPVAAATNITKEPNRRASDVITNTRKGTGVRTEALRRGTLN*

>LfuGbXc

MGNSAHSRLAKEGSADCSPRSFRHKSLSRRESSSEQNKRSKDASGATPAENGLEVKVIER

DPVTAKKLTERQKDLLTETWKELEENIARVGVIMYIGLFETHPDVQEAFMPFKGIELTEL

KHSKQLRSHALRVMGFVQKAVARLHEPEKLDVLLRDLGKKHFSYAAKPEYVDLIGPQFIQ

AIKPSLEDKWNEEIHEAWTTLFEYMASMMKTEMAYEEKKAIANSATETRVQNTNNRPTAS

SKTSTSSHNNPKNHGSRRASSDAATIGGTVAAVNNRRGTLH*

>LfuGbXL

MGCELGKLGLVQRERKSGDEVPEPPAPAPTDPRLPLSAKQKYNIMASWKGISRAMEPTGV

NMFVKLFEEHQELLSLFEKFKELRTKEEQRNSMELAEHATQVMRTLDEGIKGLDNVEFFL

EFVRQVGGTHHRIPGFHKDYFWRIETPFLDSVKTTLGDRYSDNMDTIYKVTIKFIICTLV

EGFEREEARAKERDAQEKKEA*

>BspHbL1

MSGIVNTILGLFGLRQNAGLDVEDPATGLTLRQKGLVRSTWALVMPNIRTVSVDVLITFF

ETFPQYYPLFTSFAGLEVTDLRSNKKFTAHATTVFHALASLVDALDDTEVLVELCEKVGK

AHISRKVPPGAFDDLKVTALKVLSTKLGNKLSPAAAEAWDKTLTAAFSLIKKELPAEDQE

>BspHbL2

MAGLFESLMGMLGIGQRDLDAEDPATGLTLRQRNYVRTTWALVVPNIKQVGVDLMATFFE

MYPHNQKLFPHFANSSVQELRKSKKFMAHASNVMFSITGLVDALDDTEVLVELCAKIGNS

HARHRVPPKAFDELKTAALKTLSEKLGKKLTPSAAEAWDKTLSVAFSLIKKDLPAQ

>BspGbX partial

AKVGVITFISLFETHPDVQETFLPFNGIELEELKQSKQLKAHALRVMAFVQKAIARIHEP

EKLEALLKDLGQKHYFYGAKPAYVDLIGPQFIKAIRPSLEEKWTEELQEAWLQLFRYMSW

VMKHSIEQERLNSGSGVAAAN

>BspGbXL partial

YNIMASWKGISRAMEPTGVYMFIKLFEEHEELLTLFTKLAELKTKEQQQGSLELAEHATK

VMSTLDEAIRTLDNLDQTIQFLXXXXXXXXXXXXXXXXXXXRIEKPFLDAVKTTLGDRYT

DNVDVIYQ

>EdaHbL1

MGGFLSYLLGSDGGAPQEELPAKWDEPEPATGLTPRQKRAVVDTWALVQPDLKATGIAVL

IALFEAHPEHQRLFSAFRDVPLSELRGSKRFAAHASSVMHAIASLVDTLEDDTEVLVELL

TKIGVNHAKHSVPPHAFSDLQAVILKLFQEKLGDRLSPEAAEAWDKTLTVANSVVTKQLS

APEPSKPQQQVEMKTEERSQEE*

>EdaHbL2

MGGLMSRGEPVDVWPKGVDPDLIDPETGMTPRDRRVVSRTFAVCVPKVREVAMDIFVTLF

TKHPEHQKLFPQFADLKTPEELRTSKRLTAHASTAAHGLAAIVECIGDTECLTAMLNKLG

DNHKRHHVSPSAFPDFKDVLMEVLKRHLGALFTSEAELSWDRAMNFVNRTVTARLV*

>EdaHbL3

MRFLISCVLSMVFLLETTIANPNDLHSEEISGVEDIIKNVLAPDLATHGPALFLELFKLE

PGYQKLFTKFADVPMDSLKDNAALKAHSVNILGKFAGCVLENIKSADNMQGCFKGQAETH

KKLNVGYDHAKVLSKAIQNYLSANLPHGYGGDVKTGWNKVLYVMTEAFK*

>EdaGbX

MGVKNSKLIEAKCPGAPNSPARKSLEPVPEPAKTPENMTSCCETIPIPVLAPLELESIGP

PPLTEEQCVLLAETWKELESNIAKVGVITFISLFETHPDVQQTFMPFSGMGLEELKQSKQ

LRAHALRVMAFVQKAVARLHEPEKLEALLRDLGQKHYFYGAKPEYVELIGPQFIQAIRPS

LEYRWTEELQDTWLQLFRYISDIMMAAIKQESLNHISNNPVEH*

>EdaGbXL

MGCELGKLSSKGNRAGKDTAKDGNLNPPAPAPPDPRLPLTAKQKYNIIASWKGISRAMEP

TGVYMFIKLFEEHEDLLNLFSKLGELRTKEQQQSSLELAEHATKVMGTLDEAIRALDSLD

SLIAYLEAVGASHRRIPTFERNHFHRIEKPFLDAVRTTLGDRYTDNVDVIYQATIKFIID

SLQAGFDKAEGNPEMVNRPPPAHIEDIS*

>LysHbL

MGGVVSYFFAPGNDPNDDIPESITGLTPREKRAVTESWAIIRTDLKGNGMIFLLMFFDDY

PDYQKFFRSFAEVPRSQLPDDKRLMAHVTSVMYALSNIVDNLDDPECLVETLKKLGENHG

RRNITLQEFENLKAVVIKFLKHCVGSKFSSTAEAAWVKTLDSAVSVINKGIP

>FcaHbL1

MSLTAEEKTAVQDTWGVVAKDLKGNSIKVFLHFFTMFPEYQKLFRGFADTPMDQLPENRR

FKAHAFTVVSAINGLIDNLDDPEMLCELLVKTGQNHAKRSIKIGDFKNLNDCLMDLFSKI

FGEAWTPVAKSGWSKVFSVVLEKVAEGLKLNE

>FcaHbL2

MINLTPEETSAVELTWSEIAKDLKGNSVKAFMSFFRAYPHHQMKFQRFSSLPLDELPQNK

RFQAHTFTAVSTLNSLLTTLDDEDMFDEMIRKIGENHIKRHGVVQADFMDFMRILTELMS

TSLGEELWTEHAKSGWEKVSKLIGKGIGDEM

>FcaHbL3

MASSLWKQIFDSFIAGWVWLLGWILGTPSTYAPSTIKYVQSASSALTGEELEAVKDSWAL

VKKDVKTHSINLFLHFFSLYPQYQSKFPAFRDVKKAELTTNRAFKAHAFTVATALSGFVD

HLEDSEVLGELLFKTGVNHKGRGLGLNDFKNLNNAILDYFSIALGKLFTPVVMRGWTKTL

NVVLGKVGEGLNSVED

>FcaGbXL partial

ADPRIPMTAKQKYSLVASWKGIHRALEPTGILMFVKLFEENAELLDLFEKFQNLRTRQEQ

EESLELAEHATMVMNTLDNAIRTLDNPDAFFQFVEQVGSTHRRIPGYNKDYFWKIEKPFL

EAVKTTLDERYTENIENIYKIAIRLIIETLVAGFEKAAG

>StuHbL

MSLLLGFMYTGEGDDPDPATGLSSAQGKAVADTWAVVRKDLKQHGTKILIELFKAHPQYQ

AKFKGFANVPLEDLPRNKKLHAHACNIMFTIDNMICNLNDIDVLTEVVMKVGRSHKPRDL

TMSDLQNLAKVAQDYLAHTLGGQMTAAGKEGWAKVFAVVTKVMDEGMRA

>CclHbL

MSILTSNELSLISESWKLVVPDLEHHGLSFFLKLFEEYPTYQEKFFPELHQDERKIQRHG

AIVLKSVGKLVAFLEANKVIALVDAIKRLATNHSRRGVLREQFYPACRILLEYLAQALGT

HLSTEGALAWKRFLGTFVELMQEGYAQLDASK

>LsaHbL

MVTMSLFCEHEVKLISESWKLMALDLDNHGLNFFLKLFKEYPVYEEKFFPDINGDRKKLK

RHGGIVMKALGKLVGFLETGKIIAIVNTIKGIANSHSKRGVLVQQFTPICDILLKYLGEA

FGDQLSNEGTATWKKFLDIFVSVINEAYDEIKNKK*

>LsaGbXa

MGNAGVKEKSNILGGRGPVVETIAQLQTHQAMSSQPKSSVDTTNYLSKKQKDLLKRAWVA

LHNNLSSVGMTTFIKMFETHPEALKFMIPKLTQEEEKKTQSNYSLDSRLDPWHSEKLREH

AHRIMKTVSDVISLLNKDEEKIEEMLVALGGKHHGFGVHIEILELMGPHFISAIYPTLKE

TWTEELQEAWQCLFNYIIALMHIGFDQALLAKVKPTWINYFD*

>LsaGbXb

MGQRQSSHCKYLEKSKCSRNSVPIQSSPNTCSVEHHPSLSNEQLPVDKIDLKVLMEETKS

DWEISKEYKALLRQAWRLIQSDLRSVGTVTFLKLFETHPETLTAFIPKVNSLKEIEMNEW

YSENLKSHAIRVMAIIEKVMHRLDDEERAARILIAYGKKHFQIGVKTEMLEKMGESFVTA

VEPSLEKVWTKKMAEAWLSLFYFITYFMKLAYPNKPESVDSL*

>EafHbL partial

LNSRAHAGLVMSTLNFWVSNLDDIDLVVASVQKTGRNHSGLNCNNNMKSRLSHIF

>EafGbXL partial

MLEGAFLDSIRSILEDRYTPQMETIYSILISKFYQLINKVVLQGVPKNMGI

>DpuHb1-1

MQFLKIALFFAFVALASSSSSCSQAPGTTITSVTTTVTTVTADEDSDSGLLSSHDRSIIR

KTWDQAKKDGDVPPKILFRFIVANPEYQKMFKSFATVPQNELLGNGNFLAQAYTILAGLN

VVIQSLSSQELLANQINALGGAHQPRGATPIMFEQFGAITEEVLAEELGIAFNAEARQAW

KNGMRALVAGISKNLKKAEDLA

>DpuHb1-2

DPQTKLTPHQIHDVQRSWENIRANRNSLVSAIFVKLFKETPRVQKHFAKFANVAVDSLPG

NADYEKQVALVADRLDTIISAMDDKLQLLGNINYMRYTHQPPRAIPRQTFEDFARLLIDG

LTASGVSGDDMDSWKGVLTIFVNGVSPKQ

>DpuHbA

MDVLNSVNVAAVQSTWAVIKSDINTFAPQFYVALLTAHPEYQAMFPTIANVPSGQLLNNA

ALITLSVNVVTKLSEIIDSLGNPGALNGKLVDLANQHKQRGTTRAHFDNMATVLLGFLAA

TLGSAFTPEAKQAWTSTMQGINTVVEASA

>DpuHbB

MKEADRTLVQGTWRIAKKNGNIAPKAFIRYFKLKPEAQKQFAAFADVELADLPTNSHFLN

QVYTCLAGLNAYMENLGKNPKQCPHLNSPVFKAVKPDDLKLFGEVMFTVMEEELGQSFST

EARKAWKDGLIACDVAFRKSH

>DpuHbC

MTRLILALCALLSLAVGQSPYNDRFSGGIGSGSWYRGGGMSGSGGPFVTTTTVTTVLDLS

TGMTKTFHSSNNPSSGGSSGGERYSSYGGGYGGGEKNKFTEGSSGTSPLMNILSENDISV

LVNSWHILKKRSDFAPKVFMRYFKAKPEAQKLFSEFANVSVTDLPNNHDFLNAAYSCISS

LEFILPHLRFQHPERCPALTDLKNKYSVVDLKRFVPIWMAAMQEEMGNAYSNEVRDVWKK

AFSAFTDYASTP

>DpuHbD

MDVLKSVNVAAVQSTWAIVKADLNTHAPKFYVALLTAHPEYQPMFPTIANVPAGELLNNA

ALKTLSVNVLSKLSELIDGMSNPDGLNAQLVELAKQHKNRGTTRTHFDNLAKVLVDFLAA

NLGAAFTPDAKQAWTATMQGINTVVEANA

>DpuHbE

MSWLILVFCSILSLSAGQSPFNEGIPGGVESGGYGDSTMRGSGPFVTTTTVTTILDFNPA

GMSRSMRSYPSASERSYHYGDKKFGSFSQKDVDVIVNTWNTLKRRGDFAPKVFIRYFKAK

PESQKMFPAFANVPITELPTNHDFLNSAYTCITSLNYLIPYLKFDHPERCPAFPKHLKDK

YNAVDLKKLGSIWMTAMQEEMGNAFTNDVRDVWKKAVMAVIEYASK

>DpuHbF

MSMFILVLCAVLSLSAGQSILFKEGTQGPFIATTTVTTTFDFNPAGVPRARSSACDRTHY

DSDKKYGSLSQMDVDIIVNSWNILKKRGNFAPKVFIRYFKAKPESQKLFPAIANVSITDL

PTNPDFLNSAFTCVNSLNYLIPFLKYDHPERCPSFPKQIKDNYNEVDVKKLGSIWMMAMQ

EEMGSDFTNDVRDAWKKAVMAVIEYVSK

>DpuHbG

MLVHYILTAILVVFLETRPGRAECPRGYKASSSGQRDPSTKSESQRILENFLNERDEATI

RSTWNTAKKNGNIGPKTFLRYFELKPEAQKMFPAFAEVDHMKLPTNEDFLAQAQNCVSGL

NSYVEHLGKNPKNCPFIAKAKGKYHHEDLKLLGVTLMGVLEEELGKGFTDETKEAWKKGL

RAMNEAVTKRPNPSRR

>DpuHbH

MSHTSPNGSQLLEDLQREQPETIASSDDNPIDPVTGLSQRERDYIQQSWHHVRQDLKAAG

LGFFQAFFKAHPDYQLKFKKFADVPADQLADNKSFLVHAMSVMNAVTMVVDSLDDIPKLV

NELKNLGKNHGRHNIKTENFRNLTVVLVAFLESALGSQLFPEDVKQSWIKALDVVVGVVA

TGLPQPSPDDDAGSAM

>DpuHbIPartial

MAFKLALLLAVVAFASVSATTTTTVTTSVTTVSADDDSEGGLLLSAHDRSVIRKTWDQAR

RDGDVPHPQILFRFIKAHSKYQKMFSKFASVPQNEYQRIEITKFGKPSAHHRGEMHVRRN

QVDIT

>DpuHbJPartial

MAFKLALLLAVVAFASVSATTTTTVTTTVTTVSVDDDSEGGLSLSAHDRSVIRKTWDQAI

WRDGDVPPQILFRFIKAHSECQKMFSKFASDPQNELLTNL

>DpuK

MSKALRFYAPGSDRRHHGGSEKKLFEEFGLQRSLSDSDINLIVSSWNFLKKRLSSFAPKV

FIGYLEARTDSKKMFPDFAHVNIAELATNVEFRSRACNCVASLNYIIPHLKRSFPVLQCP

ALKNLKTKYNQHIDILKSLGIIWVKAMQEELDKKIFTDDVRVVWKKLFSVLKEHVSEF

>DpuGbX

MGNAHVTHGGNGKNKTANGKDEVRNGGDRIFAPDSTAFPPAKKAMASVAELTDLQKTLLQ

ESWKRLEKDIAQVGIIVFINLFETHPDMQSVFLPFTGVVLDDLKKSKLLSEHALRVMGAV

QRAVHRLQEPEKLHAFLSELGRKHEKNGAKLEYIDYIGPQFLCAIRPILGDDWTLETEKA

WTLLLDYMTATMKESLVEARNASAAESSKPLTLPPSSSSSSSAATDD

>DpuGbXL

MGCVQSQSNGQPAAKDKGGGAAGGVINDNNGQGGASAVIADPRLPLNARQKYSMLASWKG

ISRALEPTGVYMFIKLFEEHKELLNLFTKFHKLTTRDEQAGSEELAEHAVSVMTTLDESI

RSLDNVDTFILYLHQVGQSHYKIPGFQKEYFWKIRNPFLEAVKMTLGDRYTDNIENIYKV

SINLVIETLVEGYEKAHQQHLAGPSS

>AsaHbT1-9

PQNAFSAYDIQAVQRTWALAKPDLMGKGAMVFKQLFTDHGYQPLFSNLAQYEITGLEGSP

ELNTHARNVMAQLDTLVGSLQNSIELGQSLAQLGKDHVPRKVNRVHFKDFAEHFIPLMKA

DLGDEFTPLAESAWKKAFDVMIATIEQGQRARRSVATFLTNPVA

>CmaHbL

MGAVLSVVWGWLSPGTQVGAVTFPEEGSLGPEADVPDKTTGLTLRHRTAIYRTWDLVRPN

PKLHGINLFLTMFQEEPVLQTRFKGFAGKSIEELKNSKRLAAHGTTVVMAITAMVDNLED

VSVLVELLKNTGANHRDRGVPKGDFELLAPVLVRFLKDNLGSAWSPVAEEAWTQAMKVIN

AVIFTSYDA

>CmaGbXL

MGCHITKNKKNKETEEVKVIDLPEPPEPPPPDPRLPLTARQRFNIIKSWKGIARAIEPTG

VNMFVKLFENHSELITFFTKFRQLRTRDEQAESLELAEHATIVMNSIDEGIKAMDNVDFF

FDLLHQIGASHRKIPGFKKEYFWKIEHPFLEAVRLTLGDRYTDNMDNIYRITIKLLIETV

VRGYELAELKEPNDNV

>HazHbL partial

RTITDKETWFLQYPALGPNPDKPNDITGLTPREARAVVVTWDIVKPNMAHHGVQFFLRLF

ERHPNIQKKFSGFDGKPLAVLQEDKRLRAHATTVMNAINSVVESLGDPETLVEILKTTGK

NHKRRGIEKTHFE

>HazGbXa partial

MGGAHSKDAPNNQDSGAKDADSDRASASEGKNVKKFDREEILGQKPDDLTDQEKTLIRDT

WQDVETSVARVGVVMLSWLFETHPDVQESFMSFRGVPLPEVQQSKQLRNHALXXXXXXXX

XXXXXXXXXXXXXXXXXXXXXXXXXXXXXXXXXLIGPQFIAAMQGSLQRRWEACVEEAWL

RLFRCLSFSMKAAMLDLTGD*

>HazGbXb

MKKKTSIDVDWSKFKFASSDGNSVSRKPSQDCDDTSAAPSWHRPSKGSFDLGRVALRFTK

RSSTDKGIDGPALEEEPPPAELTQEQKIIIKETWAIVKQNVERVGVIMFTNLFETHPDVQ

EVFLPLRGMEKNALLDNKKLRNHALRVMGFVEKAVGRLEEPAQLQALLETCGRNHCGYGA

ALHHIDLVGPQLLEAIKPSLEDRWSPEISTAWTLLMDNIAYAMKAAMRLQMRQA*

>HazGbXLa

MGCQLTKALTEGDGKSPTSPNAPNGPKNNKKKKKKDKDIDANGKLEEPPPPPALDPRLPL

TARQKFSILKSWKGISRALEPTGVTMFVKLFERNAELLSLFVKFRELKTPDEQAESLELA

EHATVVMSSIDEGIRAMDNVDFFFDLLHQIGGSHVKIPGFKKEYFWKIERPFLEAVRLTL

GDRYTDNMDQIYQLTIKFVLETVVKGYEMAVEREVNENVQQLDLSSEPAMNNGSDVPAQT

TSAQNGANSAESSAVRAQGDAAPATAGGGKCPAVCPYVSSGSLSPTTS*

>HazGbXLb partial

MGCRMTKDSGRDHRPLVAPRPVIPPDDRLPLTIKQRFLILKSWKAISRAIEPTGVAMFI

>SsuHbL

MDPSLDISDPVTGITARQKLVVRENYGRGAKNLKSNGVEFFVALFTKHESLKKYFVTLVD

LPIEALPSSKKLQAHSTTVMMAISGLVDNLEDVECLKELLLKIGENHSRRKVSIEEFKKL

AVVFVDFLEEKLGDGFTSFARKAWEEVFRVMNSIIEEGLKSATGR

>SmaHbL

MGGWISYFWPQKSAEFDVSPGLDEVESASGLTLRQKKVVTEIWDLVK

IDIKQNGIDFFIEFFKAFPLNLNNFKAFQNMTDDQLRKSKKLEAHATNVMYAISTVVDNL

QDVECLTELLSTIGRNHIKRKITPVQFDQVGITFIKFLENKLGSRITPFCRNAWEVTFKV

MNSIIVAGLQSNDD

>SmaGbX

MGVESSKTSKLCRHGQNAAPACVNSGMSTSMTGKEFCENENAYDDVESWVLDEREIEHVI

FTWKLVERNIAKVGVITFLGLFETHPAVQSVFLPLSHMSREQLGSSAKLEAHALKVMNFI

QKIIARIDNPSKVHALLRQLGKNHFHYGVKREYIDLVGPQFVIAIRPLLEMESAWTAAIQ

DSWLHLFASMSAIMKKSMGIEEEQYLITK*

>SmaGbXL

MGCSFVKHSNGAGEEGRSAKSSAGKSGVVNAVDTPAAPAPVDPRLPLNARQLFQIGKSWK

GISRAMEYTGVNMFIKLFEEHNELLNLFTKFSDLKTKEQQAESLELQEHATLVMTTLDES

IQALENVDAFTAYLHQVGRSHTRVPGYKKEYFWRIQKPFLEAVSETLGDRYTENMETIYT

VTIQFILETLVKGFEIGEKEKGV*

>MgiHbL

MSAGVKNGAQEILMGITLREKTLIRESWDLIRPDLKGNGIAFFIKLFDEFPEYQKLFKSF

DKVPKEELPTNKRMIAHATTVMYGFASFVDSLDDPDLLEGLIEKIVTNHARRQITKENFK

NIGIVLENYLKDRLGIKLTEEGIQAWRKLCDVVQAVAEEILVK

>SjeHbL

MGIVWSVFTGKPEYHLDIPDPITGLSLRDRKEITDSWHILRKDIKSAGTQFFIKLFIEHP

TLQKLFPAFSDEPFSELQTNKKLIAHGTIVMYSISSMVDNLEDAECFKVLAANVAQSHYN

RGVTYEHFSKLGPVMLSLLEEFLHRSLSVPTEVAWQKFLSVLVATAKDIADRDQNQTNSS

>CexHbL1

MGSSWSTLLVSKSDTSADSVDPATGLTKKEKDGIKYTWDIVRKDIPKNGVALFIMFFKTN

PDHQKVFTSFADVPLSELPKNKKLMAHASSVLYSISSLVDSLDDVECLKEMVIKIAHNHL

RRKVDDKHFSSLGESIISFMEEKLGSKFTSHKEAWQKFYSVVVTIVKEVQEEEHYES*

>CexHbL2

MGCNVGRQTVLKGKTEEQLLANLTSRQIELVTETWQIVSQDMANVGVIIFXXLLTQHPEL

CKLFKKFMTLKEDGTYDWDLGGMERHALLVMQALEAAIDNLDDSRVLSGILFELGCKHAR

YNVQEDMFDKLWDALKSGLEETLQEQMTKEVTQAWFSVFRYISHHIVKGMRDYRKRSQ*

>CexHbL3

MGCSVGRQSVLKGNIEPQLSKNLTPQQIQLVRSTWSIVSQDMVGVGTIVFQRFLTRHPEL

CKMFRKFMTLKDDGTYDWDLEELQRHALLVMQALEAAIDNLDDSRVLAGILYDLGRRHAR

YNVQEQMFDKLWEALRYGLEKSLQNRMTREVNQAWFAVFKYISWQVIKGMRDTYAKGID*

>CexGbXa

MGCVPAKSVSNLPYGLGGFKGGVPFVTYQQKVALVQTWNVLMENLSRVGVIAFMRLFETH

PDVQEIFIPFKGLDHESLRNSKELRAHALRVMSFVQKVVARLEQPRKLEMLLGELGKSHL

NYGAKAEYIEKIGPQFIYAVKPMLEDHWNPGVENAWLQLFRYITHYMKVTMERSDKETVD

DDNRKKNKMKIMRHSFRGKLYK*

>CexGbXb

MGCKYTKTVNANLKKKEANKKNSPLLKDEKPDLTNQQKALVLNTWKLLVENISRVGVITF

MSLFETHPDVQEVFMPFRDLTHEELSRSTDLRAHALRVMGFVQKIVARLDEPEKAEQLLG

DLGKKHVMYGAKPDYVDLIGPQFVYAVKPSLEDHWTNEIEEAWLQLFRFIAYCMKESMTE

CSRPAQQ*

>CexGbXLa

MGCRFGKTVPSVKDQNDQTDPPPPAPTDPRIPLTAKQCFSISKSWKGIARAMEPTGINMF

VKLFQDNEDLLDLFEKFQSLKISESNFESMELAQHASIVMSTLDESIRSLDNVDYLLDYL

HSVGKLHHKIPGFQREYFWRIEKPFLAAVQETLGDRYTENMETIYKITIHFILETVIAGY

DMGPETNQNSL*

>CexGbXLb

MGTSLSKGSYGFSKKGQDGISPLDDPPIPAAPDPRLPLTARQRFSISKSWKGISRAMEST

GINMFIKLFEDNEDILHLFKKFQYLKTHEQQRDSMELAQHASIVMSTLDEGIRSLDNMDY

FLDYLHSVGKLHRKIQGFNRDLXXKIEKPFLSAVQETLGDRYTDNMDSIYKVTIRFILET

VIKGYDMAEDETNGTPSEENDV*

>IscHbL1

MGNELGANTQHRDTITEMTSQEKHVVRDTWAIFKKEVQTSGVAIFVVLFFKHPAYQKLFV

AFAADPIAELPQNPRAIAHALTVAYAITSIIDTLDEPETSAELVRKVATNHVRHPTISGA

QFEHMGQAVVEVLAEKLGSAMNHQAVGSWQKFFAFVVRVSQGVFKKRPFRKARSEDT

>IscHbL2

MSWLFGSASADMPSTKTGLTTSDKCAIKDTWTMFRRETRTNALSLFVALFSRYPEYQKMF

PNFADVALKDMMQCPSLTAHALTVIYALASIIESIDDENTMVELIKKNIRNHVRRSVTPE

HFVNINNLLIEVMQVKLRSRMTASVIVSWKKFFAMHDAVTRQTYDEFRAQSAVERAAGTS

NSGV

>IscHbL3

MGSIVSSQQDVPDAKTGLTPREKGLVRDTWALVRKDVKANAIAIFLTLFQRHPEYQKLFS

GFADVPPEALSTNPRLGAHAMSVAYAITSLVDSLDDAECLVELVRKVAVSHTRRPVSVTH

FENLTVVIVDTLKERLGGKMSPAAVAAWEKTLRLVVTVTADVYKEQRK

>IscHbL4

MGNILTKSLPDSRTGLSKRDTKLIRNSWSMLCKQHPKADQLIFKALFTKHPDFMALFQHF

KDKDLGVVLSDPQFALHSSAIIQAFGTIIRSLDDPAGVVALIRKNATDHTTRKGVQPSHF

EAMLNVVLEVLQDKLGSRFKPEAITAWEKFIEVGKLLWSEEKKRFVVFW

>IscHbL5

MGYGVSRQSVLRGEVDPDLSNSLTARQAELVRSTWAIVSQDLAGTGVVVFKRLLTRYPEL

CRLFRKFMTLRDDGTYDWDMEGLQRHALLVMQGLEAAVENLDDSRVLADILYELGRKHAR

FNVHEDMFDKLWHALKFGLEDALQDRFNREVAQAWFIIFRFLSRKIIEGMLEHRAKMAEE

KAKQEIGDKPDKITDKGKPR

>IscGbXL

MGCTLSKTLSSLVRGSEGRKGSNSGTGGSEADPVADPPPPPPPDPRQPLTARQIFSISKS

WKAIARAMEPTGIEMFVRLFQEKEDLLDLFEKFQALRTKESQRESMELAQHASVVMTTLD

EGINALDNLDYFMSYLHNAGRLHYKIKGFKKEYFWHIEGPFLAAVSDTLGDRYTDNIENI

YKITIRFILQTLIEGFEEAEKEAAQQAR

>MocHbL1

MGNIASPHKLSTDEVDAVQAAWQVVRQDQRSIGQQVMMTLFSENPEYIHKFKHLQMIAAD

QLPYHTALRAHSLSILYVIHSLIDSMDDEETMRELIRKVALTHKPRSVNRDNFQRFEDAF

ILVLKKYGIDRRTEEAFHKCILYFTDIYEKAEDT

>MocHbL2 partial

DQTRFLSFAEVPLDEFGRHPKLLAHALSVVSALSSIIESIDCGDIVGALIIKQVTNHFPK

CITLQHYE

>MocGbXL

MGCTLSKAVTSLVHKGSERSAGVGRVGDVEDPPPPPPPDPRSPLTTRQIFSISKSWKAIA

RAMEPTGVEMFVRLFKQNEELLDLFTSFQALKTEESQRESMELGQHASLVMTTLDEGINS

LDNLDYFLEYLHNAGGMHYKIKGFKKEYFWLIEKPFLEAVKLTLGDRYTDNIENIYNTTI

HFILETLVEGFALAETKAKCSE

>SscHbL1

MSLTNRDKEIIVSTWSLIRKDSDQAGIHLFKRFFEANPDYVKYFPFGDLDDLEKILVDPR

LKWHASRVMAALSTIVDNLDDPVCFEDSLQKVLSSHLNRKIQLYHFENLKKALVCLFMDK

LGPDIMNDETIEAWSKAYDVILDTYRSRLSEAKSSIS

>SscHbL2

MTEFEREEIEVLREQWDRIVHYHQECFGMKLFQRLLQLHPEYRPLFGFEETVEEIQNTQR

LKAHGINVVYMLNMLFDNFDDMDMIDELIFKLVKLHMMRGIDQIWLDDIIEPFELVLEEF

NAKIQIERIEVLRKAFIFIKNRMQELYDENVVAKMLDTSDISEY

>SscHbL3

MFQKLRSRLSNLSDSDDELKAENQTELTSKEIDFVRNTWSLLRNDIARFKFLGGELFVRF

FTKYPDYQRQFKSFKDVPMDFQRNRDIRFNKKLMAHGTYVMYTIGMLVDNLERPLMMEQM

LKRLARNHYRRKISLIAFDRLRNTFLEHLAEILGPKIFTKKVSIAWSKAFNYLLMEIEKN

FKILESDLERSGSYCRLNSMHLAARNDLVRLQRLKESGKKKTLNVNNDCCGSSSEQNLIH

QRIRIESPAANIKQIRHNSQCSAPIIDERREAERSKSFLTKTISFLRKNRL

>SscGbXL

MGCPLSKTSDENDVQSKQNDFSNEVMVTNKTVDPRLPLTVRQKFNLSKSWKGISREMEMT

GVLMFVKLFEETPEILNLFTKFQELKTKDSQMKSMELAEHATKVMTNLDEMINSLDDMDY

FFRHLHSLGKYHRRIPGFHKDNFLKLEKPFIEAVKEVLQERYTENMANIYNIIIKLILQT

ISEGFEKDFD

>SmiHbL

MMGFAVSKLSWFWTSAGYDTPDPATGLTPRQKDIVRNTWKSIRADTRNNGIKLFLKFFEA

YPEYQLLFKSFANVPLSDLPRNGRLLGHVTSVMYALNSVVDNLEDPECLIEILQKTGISH

RPRNVNRQHFNNLKVVLIKLLVEILGSNVMNESAVEAWEKTLDVANSIIIKSLEAEGDA

>SmiGbXLa

MGGNLSKALTALQKKGEPETVDAPLEDPPTPPAPDPRLPLTARQLFNISKSWKGIARAME

PTGITMFVKLFEDNEDILHLFQKFQYKRFHEFHRDSMELAQHAGIVMSTLDESIKKLNNV

DYFMDYLHSVGKLHTKIPGFQRDYFWRIERPFLEAVQDTLGDRYTDNMENIYKITIRYIL

DTVVKGFDLGALQTKTSEVPPPKASPEPSPSPQEHRDEQEGKCVNSQCPAVTNGQS

>SmiGbXLb

MGCTFAKVPKDGKGSVQDLNHAGDAPSAPPAQDPRIPLTARQKFSISKSWKAIARAMEQT

GVTMFTKLFEENEELLELFEKFKHMKSREEREQSEELREHATTVMTTLDESIMSLDNVDQ

CIDYLRNVGRSHRKIKGFKSEYFWKMEAPFLAAVKETLEDRYTENMESIYKITIHFILQT

VIEGFEGTQQQNSV

>CspHbL1 partial

MGNVWSWIGWDAVDPAENEPDPVLGLTPREMKAMAESWQPIRKNMKYIGLKFFSSLFIQN

PKFKPLFPIITGLSDDQLMTSKKLQAHGTTFMLSITNIVEHLDEPEVIVALCHKLGQTHN

KFGLTEKDF

>PspHbL1 partial

SGINAEYMGKKYPQYQQKFPQFCNMTPECAMSSPALXNFTCQKFTGAMNNLIDSMDDKEL

FDATLKGIAEKHKEKGLKKQDSKKFLCLFLETLSRHEATKSCKEETVKAWELLLNNMAET

VGKYNRQMLIL*

>PspHbL2

MACLTDDQKCLIHQTWCCSIKKRPTVGVAYLLTVFSTYPKTQCFMPTAAENMCTNPELRT

VAWQIMQKMSNLIESLDDTPAFEDLVCQEMTELVCRYKIYTPDFRRLIDLFLCIMEDHEC

VKRSNDCRGAVMAWRTFRDIIIQRLCTVQQQYGIMQAKENFTC*

>PspHbL3 partial

MCDATGLTNEQKQALCSSWTILPKIAKEMAQPFCKKCSDACPKSRAKFLDLVESALQIDK

EPQFRSMVVQATGDMNSIIESLNDPEVSQEIMKNSLAKLTKAGCNKDCIKKGLEVFLSFI

ECHPAVTRTQCPVDTLRAWRQLAASIL

>PspGbXL partial

PEPPTPEPLDPRNPLSARQNFSVVKSWKGIKRAMEATGVFMFIKLFEDHVELLNLFEKFQ

DLHSRDEQAESTTLAEHATLVMKTLDNGINSLADIDKFESFLYQVGESHRKL

>CcalHbL1

MAAVIAPPPVVKEGDSAYNTPCTKSGLTPHQKAAMRESWSKLSADKRTNGTDFFAQLITR

YPEYQKFFGRQVAGMSPDQFRSSRKLQAHSMQFMHGVSNLLDNVDDMDAFGELMDKMALR

HRPFPEGKLGKDDFDKATNLLIEVLLGHSDVKSCKDNAEFLKEAWSKAFEVINSQFASRM

*

>CcalHbL2

MATKIAAPSCAGEGDAGYNNPCPRTGLTPHQKAGLRESWLKLATDMRSNGTTFFAQLITR

YPEYQKFFARQVGGLSPDQYTNSRKLQAHSMQFMRGVTNLLDNLDDLDALGELMDKMALR

HKPFPEGKLGKDDFNKATNLLIEILLANPGVQNCRDNAEFLKDAWTKTFELVNGQFAARM

*

>CcalHbL3

MGATVSTTDQTHANDPAWNTPDSTTGLTPRDKDILRKSWGVAAQDFRGNGTEFFYQFFKR

YPKYIDTFKALRGLTADEAKANKRLQVHAMGFMHGVANVLENLDEPEGLIELLERLGRRH

APFELKKEDMENATDLFSELLLTHPNLPTIEYSKDDVASTWEKAFKVINTVISLIDWSDI

MAQC*

>RcoHbL partial

MGGRASRILSSGSANVPDKVTGLSDVEKRAIQENWRIVYRDLKGNGVELFVRYFSRYPEY

KDAFESLRDIRMQDIGKSHKLRAHSVQVMQ

>MtaHbL partial

ISVLILQKIGRDHVKRGLQMKQFDDLTEVMVDLLKGLMGPAFTDQVLNAWRKALVVVMDV

VYKQMYQTEQEQLQTLGYTTNDDNSKMQQMVNNAK*

>MtaGbXL

MGSKPSKASKSKDKLNAKDGNNKASAVPQPNAAAAIDPRFPLTARDKFSLVKSWKNVSRN

LEQTGKEMLIRLMQENDDLRPYFPRYKDKTADQLRTDEDFEEFAGNVMAAYDQAIQESDN

VDAAIKTLETAGKKHKKLPNFREEYFVRMEKALIHALQSTLGDAYTDNMDRIYKQWISWT

TTHVQNGFRT*

>SkoGb1

MADPVTTLTSDEVAAIKSSWSAVYDKKKESGVTLFVKLFTENPSFKSQFGYMSGVADGDM

KTLPALENHGVKVMDRINEWMGNLTNGAELVKQLKHLGTTHIALKVTEDNFNAMDSVLMY

TLQEQGGSAFTPAAKAAWQKAWGVMKSVIVGALKG

>SkoGb2

MESTESNTPVKETTEINPDDIPDEVTILTPKEVKAISESWKVVYAKKKENGVALFIRLFQ

SVPGSKSLFKNLDGIDDEEKLRNHPRLKAHGFRVMSSVNSLIESLEEGELLVQLLKDLGS

SHSKNKVTSSHFDALGPVIIWLLQKENGDSFTPAVKNAWLKGWGVMKSVIVGSLEEAYAK

MKT

>SkoGb3

MALSAGEIKLVTDSWTAVYANKKANGVALFVRLFSENPGFQSQFRYLDGVSGLAAIEKTP

ALGDHAVKVMDTINSWIGSLGDSSAMVAKLTALGTSHIALKVTPANFDAMGPVLLWMLQE

KAGGAFTPAAKDAWAKGWDLMKSHIVKALQG

>SkoGb4

MSLSAGEIKLVKDSWAPVYANKKESGIALFVRLFSENPGFQSQFRYLDGVSGLAAIEKTP

ALADHGVKVMDTVNSWVGSLGDAPALVKQLTALGTSHIALKVTPANFDAMGPVLLWTLQE

KAGGAFTPAAKDAWAKGWDLMKSHIVKALQG

>SkoGb5

MHRKAVAILYVVVMAELMSYAVCLPVNGTSDALTFDEFAKIERANLMQYQSAKLAKLEVP

NAVTTLTPSEAIAIQSTWLFVYEDKEENGVELFVKLFTEHPDYQALFGYLEGIVGIENIK

NVPFLRVHASHVLIYLNTMLESLNDGTILVELLKTLGYTHVGLNLTPEHFDALGPILISL

LQEKGGDSFTPFAEKAWLKGWGVMKSVIVGALENGYQLEDGLGY

>SkoGb6

MGCTSSAASDRPSKNDPLLDPPPPQELDPRIPLTARQKFSIQKSWKAIQRNMEGVGMDIF

IRLFKAHPEYQDLFPEFKGMSEEKLRNSINFETHVGIFMNVIDECIDSLEDADHVINLLT

KKGRKHANYGVKPEFISDIEEPFLASVKQLLEDRYSEKIEEIYKLTIKFILEHFINGLKE

SVG

>SkoGb7

MGCSNSSHNCVSPKKEDSMEQLPPTSLTDQHRVILLDSWKVIQEDIAKVGVIMFMGLFET

HPECKEVFMPFKELQGDDLRWSSALKAHGLRVMAVIERVLARIDSDEKIEEHLKALAKKH

VEYGANSDLVRLFGPQFIGSMKRQLHKSWSDEMQDAWTVLFDIIIYHMTTNMVPEQPENN

NIAKRQKSSRKSRTKYMIDNGHSQ

>SkoGb8

MSRFTSRLSSSTLDNFEAISNLGWEKKLYEHSSTRTFRTRKRKLTIHNYTIYPAGLFLLT

VYNAMGCGSSKINGNVVEEKPELTKEQKDTLIQTWQNLHADLERIGMLMFMGLFEHNPEI

KEFFVGADSRDMKTEELRYNEKLQEHGIRVMGLVEKIISSMGFEDEKIDQMVVDLGKRHL

GYDVHIPFIDLFGRQFVFAIKPTLHTHWTANVEEAWTQLFKYIGYLMRYGYHTKLQQVQK

KNS

>SkoGb9

MGNEVAKSSRSSTSQSLSKEQEKILVQTWLSIRGDLERIGLLMFTGLFEHHPEAKVMFGL

SDTAMSPKDKENTALIKEHGLRFMNVVRDVLTLISEKNGSQAECVLIDLGRRHCSYNADI

NLIDVFGQQFIASIQPTLTGSWDKKVEDAWIQLFKYIAFTMKQGLAAELIDKSLKLNGKP

>SkoGb10

MGCSNSTADKPKLTVEQKRLIIDSWKELHIDLERIGMLMFMGMFGTHPQTREFFNFRGTS

DDPKNTQRLREHGLRFMSLVKKILVFIDEKPRLDAMLLDLGRRHQEYKADFNLIDVFGEQ

FILSVRPTLKHSWNPDVESAWAQLFKYISYMMKKGMMQTDKNK

>SkoGb11

MGCANSHHVQTNSSLKLQKQKTVDSAVSFTDRETAILRSTWPLLASDMTRNGGKIFLQIF

AVAPHVKDLFPFRYVPNDMLQQNEIFKMHGRRFMQSVGAVIENIDNLDGDISILLHNLGK

RHTDFDEVDGAYFDIYTDCMMHTWRSSLGNELFTPDVGQVWHKLFDFIINCIKDGYFLAM

KNKKNNSDEVKKI

>SkoGb12

MGCTPSINERDFQTPVDDKHLLDDRQKRIVRKTWRPLANDMTENGQKIFINIFESHPEIK

YMFPTRDIEGRDNLSANPHFRMHSSRFMQSVGAAIDNLNDLDNALRPLLVKLAKTHVRFK

GFKPDYFDAFEEAMLSVWQEELGQRFTTEVEESWKLLFFYIKDCLKEGYDIAMNEKTSGE

LNNSDFINQ

>SkoGb13

MGCSVSTSTENGKNFVQLSDSVDIFTERQRRIVRKTWRPLANDMTGNGTKVFLHIFEMNP

KVKQLFPCRDKTGEELLKDLNFKGHASRFMQSVGAAVDNLDNLETSLAPLLMNLGKSHNH

FSGFELNYFDSFTGAMLHVWELELQDRFTPEVMEAWKLVFDYMMGKMKDGYITRRDEKLN

ETNEQKNKIIV

>SkoGb14

MGCTESIGTAVAGMRGNNVHIANLSKSPRFSSEQISELRRTWPKLACDLTGNGAQVFLQI

FAINENIKILFPFRYVPVDILSQNEVFRGHSRRFMQAVGACVENLENLDGDVTTLFVGLG

KKHIHFEGFKVDYFSTYVTSMQTVWDIALTGHHYDKQTKQSWTQIFEFVITRMAEGYHIA

MDEQEAKKKNQLAHENGKII

>SkoGb15

MGCTVSTKPDLYSQNGDADPYKKKKVAPVDSRLPLTARQKFQITKSWKGIARNMENTGKS

MFMRLFQSNIELKNMFTGFEEFDDLEDMRESQQLENHASLVMYTIDEAIASIDDIDFVVE

LLGKIGRTHTRTDFNPQLFWRIEQPFLSAVKETLEDRYTKNIEEIYKITFRFIVDALIDG

VVAGVNERKAEAEAEAEAKAEAEDEQDNEMMKKTMHNGDKEDNCIESSKQKDITVIASTK

QR

>SkoGb16

MGCLISSNGEHPMITKEQTKILTSTWHSIHGDLEKIGLLMFMGMFDNYPETRQFFGLSGG

SIVLEDPAVIQKIREHGLRFMTTARKLVMNLDDKDKFDRILLDLGRRHHGYKADVDLIEV

FGQQFIASIQPTLKDNWNPAVGEAWEQLFKCVSSRMKDGFLQAQSSPSNTELLK

>CinGb1

MPFTDEELKLLRDSWDEVKKLGMKEVGLHIFTGLLNAAPSLRTLFYTIDLPDEEELTIDV

MRENKKVVAHATRIANAISKFIKFLDQPDELEKLLTSLGESHARRQVDPESFEYVAPVIL

SVIGGHLKLPSNSPTLQAWVKAYGVLRNGIVSAMEA

>CinGb2

MGLTTEEIGLLRSSWNEMKTIGMKELGLLIFHRLFSDVPRIRKMFYNLELPDDETLTMEA

MRSNQKMSRHATRIATSISTYLKLADQPEELKTFLNGLGELHAGHNVEPEDFEYLAPVML

AVIGGQLNLNSNSSILQAWVKAYGVLRNGIVRGMYAYQG

>CinGb3

MSLTSEQVVLLRSSWQTIGKLGMSNVGLAVLHRLFNDVPETLPFFHSVLSPTQQTEIEVL

KSNAKVVRHASRVGLSIDKIINLLDNGEELVKYLLFLGQVHVKRSIPRKYFSAMGPVLLS

VISAVLEKDLDAPVMQAWATAYGVIEQGIIDGM

>CinGb4

MKIICGLILFSTFAIIFVSGLNCWTCNVLGGNNVCRRSGGLRTCFNNQVCYNEVRRRGNT

INIRKGCKNSGVCENHIMQSMSTPNPNNQCVNGPNYFCSCCCGNSVCNSNWLTCVTAGSV

APTVSTTVPPADEGLKRSDIINIQDSWNTLKGFGYETVGMLVLHRLFNDAPQTRYLFSQL

SLSSNESFTLEQMRNNSRVVYHANRVARAVGRLVDLIELPTNFTDHLVWLGQRHAYHGVA

PVNFDYMGPVLLETIKVNLELPSDSPTLSAWAKAYGVIKNGIKDAIIATYAEG

>BflGb1

MGAFLTKPFSLVGRLLWKVLFSWWVKQIETPSDVTGLTPTQSRLVKESWKMFLSKKRENG

FVIFRVLFTDYPVTRKLFKGVEQLDLDAPGQLESSITLRAHVTRFMHSFDTYMESLDDPE

DLKQLLYDTGKSHLIHDIKPEYFDVLETVLMKSLRIVFGSKLTPQLEEAWQTAYSHLKVT

IKQGLEDAIQKRDQADTSVVVTVE

>BflGb2

MGGALGKPLSLVKTLLWKVLFSWWVKPIETPNDVTGLTPTQVRLLQQTWKVILLHKKQNG

FLIFKILFTDYPMTKKLFKGIDKVDPEQYEKTTSMRAHVTRFINSFDSFMECLEDPEALK

SLLYDTGKAHLRHNTKPEHFDDLEVVMMKSLKAVLGLKFTESVEEAWRTAFAFFVVHLKM

GVEDGLRGREKKNTSVVGDVE

>BflGb3

MGCSASMTGMGRAGPALPEPEAPPPVDPRLPLDARQKFHLEKSWKSVARNIDRAGMFMFL

RLFRDCPEMIEKYPELRGMDDQEELRNSQFLQEHSQRVLDAFDHTIDSLDDVDYVIQLLK

KIGQMHADLELKPDDMWKLEQPFLAAVAECLEDRYTPKFQEIYSKLITFIIEHVVNGFDP

H

>BflGb4

MGTIADGEGTELNGYGGEKEPGGGHGGPLTQEQVHGIKETWAILAQDPVERGVDLFMKIF

EEDPDLKKLFYFADDGRELSREDQRMRSHGERVMEAVGGAVDSLGDLTAVVPVLTELGAL

HHKYGVQPSYFDTVGAALIYILETNLGDKLTPNIRQGWVLVYGIVGATMKKGMQQAMDHQ

NMAKTRP

>BflGb5

MGSLSAKEDGTPDDVTGLTANQIRHIRETWQVVLSNKRANGFAIFRILFTDYPFTKKLFR

SMDQVDIDVPEQFEKNIALRAHITRFLHSFDTYVSNLDEPADLQQLLYDTGKSHLRHSVK

PEYFDALGNVLMKGLTAVLGKDFTEEVQGAWGTAWGFFVIHLKQGLEDAVRHGAETNGTA

AGTDE

>BflGb6

MGCAASIKFADLKNESLPEPEAPLPPDPRLPLDPWQKFYLEKSWKTVARNIDKAGMIMFV

KLLRDYPEIQQKWPQLKHLTDEEVTKSVYLMNLATRIFDTLDHAIDSLGDLDYLIPLLKR

LGQMHADMKIMDPEDIWKMERPFLESVRACLEDRFTYKYEEIYSKFIIFIIETVVIGFDP

H

>BflGb7

MSLSAADKKLVQESWDKVSKPSFADAGERVFLKLFQRNESTKAHFKKFKDIPSDQLAGQA

VVRDHGEKVCKVLDDFIKGLDGSGDEAVKKVGRMHKGLGMSNEQIDQMKGAIIEVLADAG

FGDANYKGAWGKLWDRFMAIHRAAY

>BflGb8

MSTDRSAVVSLTEGEKATIRRTWAVASRDMMGNGANILLKMFEINPDTKKVFAKFRNIPD

NQLQSTPRFRAHVTRVMASIGTVVNSLDDQEVLLDLFKDIGKKHYPARVPTEYFDVIAGA

ILCMLQQCLGTGYTAEVDSAWTKLYGSLGRHAKDGLREAAAMGTP

>BflGb9

MGSWWGKPVDNTPDDITGLTANQIQLIRDTWQIVYKNKRENCFAIFRILFTDHPSTKSLF

RLMDAVDLDVPGEFEKNVAARAHMVRFMHSFATFMDTLDEPAELRQLLYDLGKNHAKHQV

GPELFDALGPILMKALPIVLDGKFTPEVKTAWLTAYTFMSTHLKEGVEEGQRQLADSK

>BflGb10

MSLSAADKKAVSDSWAKMSKPSFQDAGERVFLKLLKKDSTKAMFKKFKDIPRERLAGNAA

LREHGGKVVQALDDFIKGLDGSGHETVRNVGRIHKAAGMTNDNINLMKPILLELLDEVGC

GDAKAAWDKLWNLFMTVHGDGC

>BflGb11

MALSAAELATVKQAWAKLTASSFEDAGEKVFLALLKDPNIKANFKKFKDIPEASLPGNTD

MRAHGKKVCTVLDKFIKGDEGAAKSTGTMHKGLGMSNDQIGAMRGALVAVLNDAGEGGAV

PAWNKLFDHFMEVHKTGY

>BflGb12

MGSGASRPTPRKRKAKKGPLPSPQPPKPLDPRLKLDAKEKFFLEKSWKTVARNEDVAAMA

MFINLFRSSPEIKDKWPQLRKLSEDEMRDSPYLQKLSVRILGAMDHVIDSLDDPDYLIPA

LEKLGQMHADMTNPIILPEDLWKLEGPFLRAVGEVLEDRFTRKYQDIYQDIYQKFIIFVL

ESIVIGFDPF

>BflGb13

MGCEMSTDGQALSSVIRKDRSELYKSPGIGDREDWRLPLDAWQRFYLQKSWKTVARKSDQ

AARTVFLRMLQDNPGLRQKWPRISLLTEEEIPTSPYIKFLGERIFDCLDYIIDNLGDLDH

VISELTKLGRQHSDMNVMTPEDVWAIEAAFLAGVQECLEDRFTIKYEEIYSRFIVFVIET

MVIGFDPH

>BflGb14

MGANMGCSNSKKMSHESESANSGDSTPPKSSTPSALDERLPLTQKQKFLLLKSWKGVARQ

ISQCGKTMLIRLFKDDPQLMAVFNQKFRHLRERDADVLYQDAILDAHAATVMEALHEAIT

HLDDSVFVMKVLHDVGKMHQRYNVDPSVFLKVEKPFLTAVSEVLGDRYTKNMEEIYTITI

KFILATLSEGATMELTEDEQKNLGRLWRPPGRVHKFVRPEKVAAIVDAQSEENGVH

>BflGb15

MGLTSEDKSAVLDSWAKMSGPTFQDAGEKVFLLLLKTDSTKALFPKFRDIPYDQLAGHPD

VRDHGGKVMQVLDDFIKGLDNGGDGAVQKVGLLHKGVGVSHDNINLMKPVLMTLLGELGC

SSAAGAWENLWARFMDVHRTCY

>HsaNgb

MERPEPELIRQSWRAVSRSPLEHGTVLFARLFALEPDLLPLFQYNCRQFSSPEDCLSSPE

FLDHIRKVMLVIDAAVTNVEDLSSLEEYLASLGRKHRAVGVKLSSFSTVGESLLYMLEKC

LGPAFTPATRAAWSQLYGAVVQAMSRGWDGE

>HsaHbA

MVLSPADKTNVKAAWGKVGAHAGEYGAEALERMFLSFPTTKTYFPHFDLSHGSAQVKGHG

KKVADALTNAVAHVDDMPNALSALSDLHAHKLRVDPVNFKLLSHCLLVTLAAHLPAEFTP

AVHASLDKFLASVSTVLTSKYR

>HsaHbB

MVHLTPEEKSAVTALWGKVNVDEVGGEALGRLLVVYPWTQRFFESFGDLSTPDAVMGNPK

VKAHGKKVLGAFSDGLAHLDNLKGTFATLSELHCDKLHVDPENFRLLGNVLVCVLAHHFG

KEFTPPVQAAYQKVVAGVANALAHKYH

>HsaCygb

MEKVPGEMEIERRERSEELSEAERKAVQAMWARLYANCEDVGVAILVRFFVNFPSAKQYF

SQFKHMEDPLEMERSPQLRKHACRVMGALNTVVENLHDPDKVSSVLALVGKAHALKHKVE

PVYFKILSGVILEVVAEEFASDFPPETQRAWAKLRGLIYSHVTAAYKEVGWVQQVPNATT

PPATLPSSGP

>HsaMb

MGLSDGEWQLVLNVWGKVEADIPGHGQEVLIRLFKGHPETLEKFDKFKHLKSEDEMKASE

DLKKHGATVLTALGGILKKKGHHEAEIKPLAQSHATKHKIPVKYLEFISECIIQVLQSKH

PGDFGADAQGAMNKALELFRKDMASNYKELGFQG

>DreNgb

MEKLSEKDKGLIRDSWESLGKNKVPHGIVLFTRLFELDPALLTLFSYSTNCGDAPECLSS

PEFLEHVTKVMLVIDAAVSHLDDLHTLEDFLLNLGRKHQAVGVNTQSFALVGESLLYMLQ

SSLGPAYTTSLRQAWLTMYSIVVSAMTRGWAKNGEHKSN

>DreHbA

MSLSDTDKAVVKAIWAKISPKADEIGAEALARMLTVYPQTKTYFSHWADLSPGSGPVKKH

GKTIMGAVGEAVSKIDDLVGGLAALSELHAFKLRVDPANFKILSHNVIVVIAMLFPADFT

PEVHVSVDKFFNNLALALSEKYR

>DreHbB

MVEWTDAERTAILGLWGKLNIDEIGPQALSRCLIVYPWTQRYFATFGNLSSPAAIMGNPK

VAAHGRTVMGGLERAIKNMDNVKNTYAALSVMHSEKLHVDPDNFRLLADCITVCAAMKFG

QAGFNADVQEAWQKFLAVVVSALCRQYH

>DreCygb1

MEGDGGVQLTQSPDSLTEEDVCVIQDTWKPVYAERDNAGVAVLVRFFTNFPSAKQYFEHF

RELQDPAEMQQNAQLKKHGQRVLNALNTLVENLRDADKLNTIFNQMGKSHALRHKVDPVY

FKILAGVILEVLVEAFPQCFSPAEVQSSWSKLMGILYWQMNRVYAEVGWENSKK

>DreMb

MADHDLVLKCWGAVEADYAANGGEVLNRLFKEYPDTLKLFPKFSGISQGDLAGSPAVAAH

GATVLKKLGELLKAKGDHAALLKPLANTHANIHKVALNNFRLITEVLVKVMAEKAGLDAA

GQGALRRVMDAVIGDIDGYYKEIGFAG

>DreGlbX

MGCAISGSGLTAGAPEIRPGEEETPAGLTTNHIRLIKESWRLIQEDIAKVGIIMFVRLFE

THPECKDVFFLFRDVEDLERLRTSRELRAHGLRVMSFIEKSVARLDQLERLETLALELGK

SHYRYNAPPKYYGYVGAEFICAVRPILKDRWTPELEEAWKTLFQYVTSIMREGFLEEERN

KRSNTQTSSRERPDKRSTAI

>CmiHbA

MVLSKTDKALLSSSVGKIQAQATGSDVLARMFASFPQTKVYFVGFSDYTAKGPRVQKHGL

TVMTKIIEGIQYLDSLRSFLDALSAKHAHELMVDPVNFGFLGECVLSSLAYQLPDFSPEM

HCAWDKYLCEFAYLLAEKYR

>CmiHbB

MVQWSQAELDVIQGKWAALDPEKFGGKALARMFVVYPWTKRYFGKFGGRFKASDPVVMEH

GAKVMGKMQVAAKDPGKIKEIFEYLSKRHSDTIHVDPENFKLLGSCMLVEMAMTKGDWSP

EIEAINRKFVDVSIAALSRKYH

>CmiCygb

MEEAAGRSEQRRPDPGQYLSDTDRDIIRQTWSRVFSCCEDVGVRVLIRFFSKFPSAKQYF

SQFRHLQEPQEMQHSSQLRQHARRVMGAINSVVEKLGDPEQVRSVLALVGRAHAIKHKVD

PMYFQLLSGVILEVFVEDYAEYFTTEAQSAWSQLMALICVQVLAAYTELGWAQNSSV

>CmiMb

MCDWDLINKVWAKVEEDLAGNGQTVLLRLFEEHPETKAHFPKFKDIPLGQLTSNADVKTH

GNTVFKALGDVVKQKGKHASNLQALATTHINKHKIPPQNFTLITNVILKVFAEKFPGEMT

APAQEAFSKAFKAICSELEDLYKKGGFQS

>CmiGbY

MTGITEADKENIHFIWEKLYENPEENGKTIVLRMFTDYPETKMYFQHFKNISTLEEMKKS

PQIKRHGKIVMSALNKLIANLDNGEELSSLLAKMAERHINVHKVDLHNFQIIFNIIIAIL

EETFGNAFTPEIRETWTKLFGVIYACLESHYKDAGFYP

>CmiGbX1

MGCAISGPGQYPASGREDVVAVASLSLSDRQTQLVKETWRLVQEDIAKVGIIMFVRLFET

HPECKDAFFLFRDIDDLQQLRKSKGLRAHGLRVMSFIEKTVARLDQEDRLQQLALELGKS

HFRYSAAPKYYPYVGNEFICAVQPILKEKWTAEVEEAWKGLFHYLTSVMKKGYQDEERGS

CPREKPKHGPNSV

>CmiGbX2

MGCALSGPEEEPERASPDGKGSGMGDGSERANESGLGHGETKAEAVCSRLPLTLSVEQKD

LVRQSWERLHQDIARVGIVLFIGLFETHPECKEVFFRFRDIELQQLKTRKELQSHGLRVM

SFIEKSVARLGQEEKLEQLIFDLGRSHQRYNVDPKYYEFVGKEFIDAVKPILKEEWTTEV

EGAWKCLFLYLTTMMKMGYEDEKERGGGKRVGDRHQLKVTSPTLTPPSPHKVRL

>LocGbX1

MGCALSGSGGGARGPGFRLKSEPVPLTESQKDLIRESWKVVHQDIARLGIIMFIRLFETH

PECKDVFFIFREIDDLQELKMSKELQAHGLRVMSFIEKSVARLAQEDKLEQIALELGKCH

CRYNAPPKYYEYVGVQFISAVKPILKDSWSPQVEQAWESLFAYLAAVMKRGYHEEEHKDG

VNKASYARKRPPQSPAEEAPPNCI

>LocGbX2

MGCAISGLGLAPKQIDATEEEALPHLSDHHIEQIKDSWKVIQEDIAKVGIIMFVRLFETH

PECKDVFFLFRDVEDLERLRASKELRAHGLNFRVMSFIEKTVARLDQLDRLDQLALELGK

SHYRYNAPPKYYGYVGTEFICAVQPILKEKWTSEVEEAWQTLFLYVTRIMKRGYQEEEKS

KRNNVVIASKERPEKKGTAI

>LcaGBX1

MGCTVSTDEHTGAQSSSEGQSQPSRKQQQQPEQQQQQPQQQHAAGGEGHQLPGPPQAPSE

SQRRLVRDSWLALQGDIARVGVIMFVRLFETHPECKDVFYQFRDCEDLQKLKMNKQLQAH

GLRVMSFIEKSVARLEQECVLEQLIVEMGRKHYKYNASPKYYSFVGIEFIATVQPFLQEK

WTNEVEDAWQCLFRYIAAVMKRGYLEEEAASNGVNTANYDRGQGNHGATAM

>LcaGBX2 partial

RVMSLIEKTVARLDQDTVLEQLIFELGRKHYKYNAPPKYYFVGAEFISAVKPVLGDRWTQ

DVDDAWQAPSSSTSRRTWSAASGRKSPASCWARSPAATASATRTPAS

>XtrGbX

MGCILSSLGWQWRDSLDHTETSPLLPTLNLSEQQQQLLVESWRLIQHDIAKVGVILFVRL

FETHPECKDVFFLFRDVDDLQALRANKDLRAHGLRVLSFVEKSVARIADCARLEELALEL

GRSHYRYNAPPRYYQYVGTEFISAVCPMLHDKWTAEVEEAWKGLFAYICTVMERGYQEEE

RRHSDGRSLIDGLQGNKGLI

>XtrGbY

MADLTGADIENINEVWSKIYANPEESGRTVVISLFLTYPQTKIYFKNLKNISTLQEMQDN

AGIRAHGKRVMGALNHVIENLKDWDAVCSALSHLAKRHQDVHKVEVNNFELLFLVIISVF

KEALGSGFTPEQSKSWEKLFSITYKYLESCYANTDS

>OanGbY

MVQVTDVEKANIQSIWSKMMENLEKNGIDIFTRLFREYPETKKYFKNIPLEGNLQEDPLL

RSHGRRVMVALNRIIQNLDNWKQVCKILNPLAEKHKIIHSVDVENFQFMLKCVGDVCQDY

LGPCYTPEIAESFQKLQSSLYDQVVITYLHSGSD

>GgaGbE

MSFSEAEVQSARGAWEKMYVDAEDNGTAVLVRMFTEHPDTKSYFTHFKGMDSAEEMKQSD

QVRGHGKRVFTAINDMVQHLDNTEAFLGILNPLGQKHATQLKIDPKNFRIICDIILQLME

EKFGGDCKASFEKVTNEICTHLTNIYKEAGW

>MgaGbE

MSFSEAEVQSARGAWEKIYVDAEDNGTAVLIRMFTEHPDTKSYFTHFKGMDSAEEMKQSD

QVRGHGKRVFTAINDMVQHLDNTEAFLGILNPLGQKHATQLKIDPKNFRIICDIILQLME

EKFGGDCKTSFEKVTNEICTHLTNIYKEAG

>MsaLegHb

MSFTDKQEALVNSSWEAFKQNLPRYSVFFYTVVLEKAPAAKGLFSFLKNSAEVQDSPQLQ

AHAEKVFGLVRDSAVQLRATGGVVLGDATLGAIHVRKGVVDPHFVVVKEALLKTIKEAAG

DKWSEELNTAWEVAYDALATAIKKAMS

>LluLegHb

MGALTESQAALVKSSWEEFNANIPKHTHRFFILVLEIAPAAKDLFSFLKGTSEVPQNNPE

LQAHAGKVFKLVYEAAIQLQVTGVVVTDATLKNLGSVHVSKGVADAHFPVVKEAILKTIK

EVVGAKWSEELNSAWTIAYDELAIVIKKEMNDAA

>CglHb1

MALTEKQEALLKQSWEVLKQNIPAHSLRLFALIIEAAPESKYVFSFLKDSNEIPENNPKL

KAHAAVIFKTICESATELRQKGHAVWDNNTLKRLGSIHLKNKITDPHFEVMKGALLGTIK

EAIKENWSDEMGQAWTEAYNQLVATIKAEMKE
