# Supplementary material for: The Globin Gene Family in Arthropods: Evolution and Functional Diversity
Source: Front Genet. 2020 Aug 13;11:858. doi: 10.3389/fgene.2020.00858 (PMC7457136; doi:10.3389/fgene.2020.00858)
Supplement: TABLE S3 — Multiple sequence alignment used for phylogenetic analysis. [file Table_3.DOCX]

>Aedes_mosquito_GbXL

-----------------------------------------------------------------------------------MGCELTK-----------------------------LASSN--------------------------GGSKG---------------------------------------------------------NNVPSLDAC--GP-PPT-------DSRLPLTAKQKYTMVASWKGI--SRAMET---TGIHMFIKLFEEHAELLEMF----ANFK-----EL-K----TKEEQA---TSEELQEHAN--KVMNTLDE-GIRG-L---D---DL--DTFFEFI-HQVGASHRRLP--GFKQEYFWRIEEPFLSAVSN--TLGDR-YTQN----VE-GIYKLTIKFII------------ETL-------VA-GYES---S-----ANNNE------------------------------DV-----NNANLSTTGSAAGPTTSG----------------DKKTS-------------------------------------------------------------------------

>Aedes_mosquito_Glob1

----------------------------------------------------------------------------------------------------------------------------------------------------------------------------------------------------------------------------------------MADTDLSTGITPDQRHVLVDAWKLV--KPDVVT---HGTNIFLKFFEKNPEYLGYF----DFSM-----DY-E----A-KELK---DNRSLHAHAL--NVMNFFGA-IIDYGL---D---HP--IMYKSSL-SKMVINHKRHG---VSKPDVAIVCAIIKDYCLQ--TLG---HSDE----LE-DAFTALLDSVA------------NAF-------D-------------------------------------------------------------------------------------------------------------------------------------------------------------------------

>Aedes_mosquito_Glob2

--------------------------------------------------------------------------------------------------------------------------------------------------------------------------------------------------------------------------------------------MDESGLTGKQKITLLSAWGLL--KQESSL---HGRNMMFLLFREHPRYLPYF----DFSS-----DS-T----N-SNLA---DNKSFHLHAV--NVMGAIGT-LIECGL---N---DP--EVFRKKL-FHLVEVHKARG---VTPLDVQLFSEIITDYLVE--VLGRQ-AANS----LA-DALGKLFDQFA------------EAF----------AYQD-------------------------------------------------------------------------------------------------------------------------------------------------------------------

>Muga_silkmoth_GbXL

-----------------------------------------------------------------------------------MGCQLTK-----------------------------LAASEF---------------------------------------------------------------------------------------NHDLIDRPP-----PPA-------DPRSPLTTKQQYCMLASWKGI--FRQIET---TGILLFIKLFEENEDLLHLF----DKFS-----EL-R----TVEDQA---QSEELAEHAT--KVMHTLDE-GIKG-L---G---DI--DSFFAYV-RHVGATHHQVP--GFKAENFWKIEQPFLQAAKT--TLGDR-YTPN----IE-NIYKLTIRFIL------------ENL-------VX-GYEE---S-----SGKENG-----------------------------S-----------SQT--------------------------------------------------------------------------------------------------------

>Muga_silkmoth_HbL

-----------------------------------------------------------------------------------MGHWLSQ-----------------------------Y-----------------------------WWGGD----------------------------------------------------------------------P-DEA-------NPVSGLTRREIYAVQKSWAPV--FANTVA---NGTEFFRRLFQTSPETKEFF----KMIR-----QL-P----E-EEYL---QSPQFRAHAI--NLMTSLNL-AVNN-L---H---QP--EIVAAMM-NKLGESHKRRQ---IKKKHFGELKQVIVKIFIE--VLN---LDGD----TL-TAWDKTVTFWY------------KHI-------FE-TLNR---T-----EESR------------------------------------------------------------------------------------------------------------------------------------------------------

>Anopheles_mosquito_GbXL

-----------------------------------------------------------------------------------MGCELTK-----------------------------LASSSN------------------------GGNSKS---------------------------------------------------------NNLPSLDAC--GP-PPV-------DSRLPLTAKQKYTMVASWKGI--SRAMET---TGITMFIKLFEEHADLLNMF----AKFK-----EL-K----TKEEQA---TSEELQEHAN--KVMNTLDE-GIRG-L---D---DL--DTFFEFI-HQVGASHRRIP--GFKQEYFWRIEEPFLSAVST--TLGDR-YTQN----VE-GIYKLTIKFII------------ETL-------VA-GYEA---S-----ANNNV------------------------------D-----------NTTSSSTPATKLS----------------DEPNRAS-----------------------------------------------------------------------

>Anopheles_mosquito_Glob1

-------------------------------MS---------------------------------------------G----PGSLVGS----------------------------------------------------------------------------------------------------------------------------------------DEEEQTNYHTPDETGLTKSQKVALIAAWSIV--KKDLVT---HGRNIFVMFFEEYPQYLDYF----DFG------GG-S----A-GELG---ENRSLHAHAL--NVMNFIGT-LIDYGL---N---DP--ALLKCSL-GKLVRNHRKRN---VTKEDVAAVGGVIMRYSLK--ALEQH-KTKT----LE-EAFGAFLGTVA------------AAF-------E-------------------------------------------------------------------------------------------------------------------------------------------------------------------------

>Anopheles_mosquito_Glob2

--------------------------------------------------------------------------------------------------------------------------------------------------------------------------------------------------------------------------------------------MDQTGLTASEKITLFSAWGLI--RKDLDV---HGRNVLLLLFHKHPRYIAYF----DFTD-----DP-N----A-QSLV---DNKSLYDQAI--HVFKAVGA-LIEYGF---K---DP--VLFDATL-RKITRRHKDRP---VYTEDILTIGEVLLNYLEQ--ALGRQ-MSDS----LP-DAFWKLFQTIA------------GRF-------PA--------TPKTPIADEGGD----------------------------------------DANDPQPTTSQRQDRASDA-----------------------------------------------------------P-----------------------------

>Cotton_aphid_GbX

-----------------------------------------------------------------------------------MGNAGTT-----------------------------RRGSIF--------------------SKQDSTGDD-------------------------G-----------STRHNKRLSSRQNT---FLEEEEFPP------EP-PAL-------PPPEPLTMRQKELLTEMWKLL--EEDIAK---VGVITFVSLFETHPDVQQSF----MPFK-----GV-D----L-EDLK---HSRQLRDHAL--RVMAFVQK-AVAR-L---Y---EP--DKLETLL-RDLGKKHYHYG---AKQKYVDLIGPQFIMAIQP--SLVDR-WTEE----MH-SAWTALFLNMA------------YIM-------KG-SMAA---E-----ERFKV------------------------------K-----------KTAT-------------------------------------------------------------------------------------------------------

>Cotton_aphid_GbXL

-----------------------------------------------------------------------------------MGCDLGK-----------------------------LASSAT------------------------SGDDRGG-------------------------------------------------------KHKDALQEPP--SP-GPP-------DPRLPLTAKQKYSMIASWKGI--SRAMEP---TGVYMFIKLFEEHQELLQLF----TKFG-----EL-K----TRDAQA---NSMELAEHAN--KVMTTLDE-GIKE-L---D---DL--DNFFQYL-TQVGATHKTIP--GFDPDYFWKIEVPFLEAVKT--TLGDR-FTEN----IE-TIYKITIKLII------------ETL-------IK-GYTE---A-----AGP-------------------------------------------------------------------------------------------------------------------------------------------------------

>Honey_bee_GbXL

-----------------------------------------------------------------------------------MGCELGK-----------------------------LAT--------------------------------------------------------------------------------------------------P--NQ-PTT-------DPRLPLTAKQKFTVMASWKAV--SRKLET---TGVFMLMRLFEENEELVQMF----SRFL-----DL-K----SKEERF---DMVELGKHAE--KVMGALDE-GIRG-L---D---NM--DDFLTCL-HQVGATHTKIP--DFNPQYFWKIEQPFLEAVKR--TLEDR-YSEN----VE-STYKVTIKFII------------ETL-------ID-GFDK---A-----QNDKA------------------------------Q-----------TSTAKS-----------------------------------------------------------------------------------------------------

>Honey_bee_HbL

-----------------------------------------------------------------------------------MGTFLRF-----------------------------LG----------------------------ISSSD----------------------------------------------------------------------D-NRI-------DQATGLTERQKKLVQNTWAVV--RKDEVA---SGIAVMTAFFKKYPEYQRYF----TAFM-----DT-P----L-NELP---ANKRFQAHCA--GVITALNN-VIDF-L---H---DP--GLMEASL-IGLVERHKKRG---QTKEEFQNLKEVMLEVLRQ--ALGKQ-YTPE----VA-EAWNKTLDMMF------------GKI-------YQ-VFAS-------------------------------------------------------------------------------------------------------------------------------------------------------------------

>Pea_aphid_GbX

-----------------------------------------------------------------------------------MGNAGTT-----------------------------RRGSIF--------------------SKQDSTGDD-------------------------G-----------STRHNKRLSSRQNT---FLEEEEFPP------EP-PVL-------PPPEPLTMRQKELLTEMWKLL--EEDIAK---VGVITFVSLFETHPDVQQSF----MPFK-----GV-D----L-EDLK---HSRQLRDHAL--RVMAFVQK-AVAR-L---Y---EP--DKLETLL-RDLGKKHYHYG---AKQKYVDLIGPQFIMAIQP--SLVDR-WTEE----MH-SAWTALFLNMA------------YIM-------KG-SMAA---E-----ERFKV------------------------------K-----------KTAT-------------------------------------------------------------------------------------------------------

>Pea_aphid_GbXL

-----------------------------------------------------------------------------------MGCDLGK-----------------------------LASSAT------------------------SGDDRGG-------------------------------------------------------KHKDVLEEPP--SP-GPP-------DPRLPLTAKQKYSMIASWKGI--SRAMEP---TGVYMFIKLFEEHQELLQLF----TKFG-----EL-K----TRDAQA---NSMELAEHAN--KVMTTLDE-GIKE-L---D---DL--DNFFQYL-TQVGATHKTIP--GFNPDYFWKIEVPFLEAVKT--TLGDR-FTEN----IE-TIYKITIKLII------------ETL-------VK-GYTE---A-----AGP-------------------------------------------------------------------------------------------------------------------------------------------------------

>Pea_aphid_HbL

---------------------------------------------------------------------------------------------------------------------------------------------------------------------------------------------------------------------------------------------MASSLSPLQISQLKDSWSVL--AQDPSQ---LASALVIRLFKENPEYQSLF----KRLK-----NL-S----I-DELA---SNPQFMSHAS--KVGAALAS-TIDH-L---D---KP--EELEKLL-TNLGIKHKKYG---LSAKHFQVIGDVLIAMITE--AIGDS--EPE----LL-DLWKSSLTSVL------------SII-------IA-ACH--------------------------------------------------------------------------------------------------------------------------------------------------------------------

>Artic_lamprey_GBX1

-----------------------------------------------------------------------------------MGCTVST-----------------------------DEHT----------------------GAQSSSEGQSQPSRKQ----------------------------QQQPEQQQQQPQQQHAA------------GGE--GH-QLP-------GPPQAPSESQRRLVRDSWLAL--QGDIAR---VGVIMFVRLFETHPECKDVF----YQFR-----DCED----L-QKLK---MNKQLQAHGL--RVMSFIEK-SVAR-L---E---QE--CVLEQLI-VEMGRKHYKYN---ASPKYYSFVGIEFIATVQP--FLQEK-WTNE----VE-DAWQCLFRYIA------------AVM-------KR-GYLE---E-----EAASN------------------------------G-----------VNTA--------------------------------------------------NYDRGQGNHGATAM---------------------------------------

>Artic_lamprey_GBX2

------------------------------------------------------------------------------------------------------------------------------------------------------------------------------------------------------------------------------------------------------------------------------------------------------------------------------------------RVMSLIEK-TVAR-L---D---QD--TVLEQLI-FELGRKHYKYN---APPKYY-FVGAEFISAVKP--VLGDR-WTQD----VD-DAWQAPS---S------------STS-------RR-TWSA---A-----SGRKS------------------------------P-----------ASCW---------------------------------------------------ARSPAATASATRTPAS------------------------------------

>Brine_shrimp_HbT1-9

---------------------------------------------------------------------------------------------------------------------------------------------------------------------------------------------------------------------------------------------PQNAFSAYDIQAVQRTWALA--KPDLMG---KGAMVFKQLFTDH-GYQPLF----SNLA-----QY-E----I-TGLE---GSPELNTHAR--NVMAQLDT-LVGS-L---Q---NS--IELGQSL-AQLGKDHVPRK---VNRVHFKDFAEHFIPLMKA--DLGDE-FTPL----AE-SAWKKAFDVMI------------ATI-------EQ-GQRA---R-----RSVAT------------------------------F-----------LTNPVA-----------------------------------------------------------------------------------------------------

>Squinting_Bush_Brown_HbL

-----------------------------------------------------------------------------------MGSWLTY-----------------------------F-----------------------------WWGGD----------------------------------------------------------------------P-DAV-------NPLTGLTRREVYAVQQSWAPV--YANSVA---NGTELLKRLFRAYSETKEFF----KMVR-----KA-S----E-HEYA---DNPQFKAHVI--NLMSSLNL-AVNN-L---N---QP--EMVAAMM-SKLGESHGKRK---IQREHFYDLKDVLVKMFIE--VLK---LEGA----TL-AAWGKAVEFWY------------KHI-------FG-TLSQ---G-----DTR-------------------------------------------------------------------------------------------------------------------------------------------------------

>Amphioxus_Gb1

-----------------------------------------------------------------------------------MGAFLTK--------------------------PFSLVGRLL------------------WKVLFSWWVKQ----------------------------------------------------------------------I-ETP-------SDVTGLTPTQSRLVKESWKMF--LSKKRE---NGFVIFRVLFTDYPVTRKLF----KGVE-----QL-DLD--APGQLE---SSITLRAHVT--RFMHSFDT-YMES-L---D---DP--EDLKQLL-YDTGKSHLIHD---IKPEYFDVLETVLMKSLRI--VFGSK-LTPQ----LE-EAWQTAYSHLK------------VTI-------KQ-GLED---A-----IQKRD------------------------------Q-----------ADTSVVVTVE-------------------------------------------------------------------------------------------------

>Amphioxus_Gb10

-----------------------------------------------------------------------------------------------------------------------------------------------------------------------------------------------------------------------------------------------MSLSAADKKAVSDSWAKM-SKPSFQD---AGERVFLKLLKK-DSTKAMF----KKFK-----DI-P----R-ERLA---GNAALREHGG--KVVQALDD-FIKG-L---DG-------SGHETV-RNVGRIHKAAG---MTNDNINLMKPILLELL-D--EVG---CGDA-----K-AAWDKLWNLFM------------TVH------GDG-C----------------------------------------------------------------------------------------------------------------------------------------------------------------------

>Amphioxus_Gb11

-----------------------------------------------------------------------------------------------------------------------------------------------------------------------------------------------------------------------------------------------MALSAAELATVKQAWAKL-TASSFED---AGEKVFLALLKD-PNIKANF----KKFK-----DI-P----E-ASLP---GNTDMRAHGK--KVCTVLDK-FIKG-D---EG-----------AA-KSTGTMHKGLG---MSNDQIGAMRGALVAVL-N--DAG---EGGA-----V-PAWNKLFDHFM------------EVH-------KT-GY---------------------------------------------------------------------------------------------------------------------------------------------------------------------

>Amphioxus_Gb12

-----------------------------------------------------------------------------------MGSGASR-----------------------------PTPRKR-------------------------------------------------------------------------------------KAKKGPLPSPQ--PP-KPL-------DPRLKLDAKEKFFLEKSWKTV--ARNEDV---AAMAMFINLFRSSPEIKDKW----PQLR-----KL-S----E-DEMR---DSPYLQKLSV--RILGAMDH-VIDS-L---D---DP--DYLIPAL-EKLGQMHADMTNPIILPEDLWKLEGPFLRAVGE--VLEDR-FTRKYQDIYQ-DIYQKFIIFVL------------ESI-------VI-GFDP---F---------------------------------------------------------------------------------------------------------------------------------------------------------------

>Amphioxus_Gb13

-----------------------------------------------------------------------------------MGCEMSTDGQ-------------------------ALSSVIR-------------------------------------------------------------------------------------KDRSELYKSPG--IG-DRE-------DWRLPLDAWQRFYLQKSWKTV--ARKSDQ---AARTVFLRMLQDNPGLRQKW----PRIS-----LL-T----E-EEIP---TSPYIKFLGE--RIFDCLDY-IIDN-L---G---DL--DHVISEL-TKLGRQHSDMN--VMTPEDVWAIEAAFLAGVQE--CLEDR-FTIK----YE-EIYSRFIVFVI------------ETM-------VI-GFDP---H---------------------------------------------------------------------------------------------------------------------------------------------------------------

>Amphioxus_Gb14

M-----------------------------------------------GAN--------------------------------MGCSNSK-----------------------------KMSHES------------------------ESANSG----------------------------------------------------------DSTPPKSS--TP-SAL-------DERLPLTQKQKFLLLKSWKGV--ARQISQ---CGKTMLIRLFKDDPQLMAVF---NQKFR-----HL-R-ERDA-DVLY---QDAILDAHAA--TVMEALHE-AITH-L---D---DS--VFVMKVL-HDVGKMHQRYN---VDPSVFLKVEKPFLTAVSE--VLGDR-YTKN----ME-EIYTITIKFIL------------ATL-------SE-GATM---EL---TEDEQKNLGRLWRPPG----------RVHKFVRPE-KVAAIV-----DAQSEENGVH--------------------------------------------------------------------------------------------------

>Amphioxus_Gb15

-----------------------------------------------------------------------------------------------------------------------------------------------------------------------------------------------------------------------------------------------MGLTSEDKSAVLDSWAKM-SGPTFQD---AGEKVFLLLLKT-DSTKALF----PKFR-----DI-P----Y-DQLA---GHPDVRDHGG--KVMQVLDD-FIKG-L---DN-------GGDGAV-QKVGLLHKGVG---VSHDNINLMKPVLMTLL-G--ELG---CSSA-----A-GAWENLWARFM------------DVH-------RT-CY---------------------------------------------------------------------------------------------------------------------------------------------------------------------

>Amphioxus_Gb2

-----------------------------------------------------------------------------------MGGALGK--------------------------PLSLVKTLL------------------WKVLFSWWVKP----------------------------------------------------------------------I-ETP-------NDVTGLTPTQVRLLQQTWKVI--LLHKKQ---NGFLIFKILFTDYPMTKKLF----KGID-----KV-D----P-EQYE---KTTSMRAHVT--RFINSFDS-FMEC-L---E---DP--EALKSLL-YDTGKAHLRHN---TKPEHFDDLEVVMMKSLKA--VLGLK-FTES----VE-EAWRTAFAFFV------------VHL-------KM-GVED---G-----LRGRE------------------------------K-----------KNTSVVGDVE-------------------------------------------------------------------------------------------------

>Amphioxus_Gb3

-----------------------------------------------------------------------------------MGCSASMTGM---------------------------------------------------------------------------------------------------------------------GRAGPALPEPE--AP-PPV-------DPRLPLDARQKFHLEKSWKSV--ARNIDR---AGMFMFLRLFRDCPEMIEKY----PELR-----GM-D----DQEELR---NSQFLQEHSQ--RVLDAFDH-TIDS-L---D---DV--DYVIQLL-KKIGQMHADLE---LKPDDMWKLEQPFLAAVAE--CLEDR-YTPK----FQ-EIYSKLITFII------------EHV-------VN-GFDP---H---------------------------------------------------------------------------------------------------------------------------------------------------------------

>Amphioxus_Gb4

-----------------------------------------------------------------------------------MGTIADG-----------------------------------------------------EGTELNGYGGE------------------------------------------------------------------------KEPG-G----GHGGPLTQEQVHGIKETWAIL--AQDPVE---RGVDLFMKIFEEDPDLKKLF-----YFA-----DD-G----R-ELSR---EDQRMRSHGE--RVMEAVGG-AVDS-L---G---DL--TAVVPVL-TELGALHHKYG---VQPSYFDTVGAALIYILET--NLGDK-LTPN----IR-QGWVLVYGIVG------------ATM-------KK-GMQQ---A-----MDHQNM-----------------------------------------AKTRP------------------------------------------------------------------------------------------------------

>Amphioxus_Gb5

-----------------------------------------------------------------------------------MGS---------------------------------------------------------------LSAKE----------------------------------------------------------------------D-GTP-------DDVTGLTANQIRHIRETWQVV--LSNKRA---NGFAIFRILFTDYPFTKKLF----RSMD-----QV-DID--VPEQFE---KNIALRAHIT--RFLHSFDT-YVSN-L---D---EP--ADLQQLL-YDTGKSHLRHS---VKPEYFDALGNVLMKGLTA--VLGKD-FTEE----VQ-GAWGTAWGFFV------------IHL-------KQ-GLED---A-----VRHGA------------------------------E-----------TNGTAAGTDE-------------------------------------------------------------------------------------------------

>Amphioxus_Gb6

-----------------------------------------------------------------------------------MGCAASIKFA---------------------------------------------------------------------------------------------------------------------DLKNESLPEPE--AP-LPP-------DPRLPLDPWQKFYLEKSWKTV--ARNIDK---AGMIMFVKLLRDYPEIQQKW----PQLK-----HL-T----D-EEVT---KSVYLMNLAT--RIFDTLDH-AIDS-L---G---DL--DYLIPLL-KRLGQMHADMK--IMDPEDIWKMERPFLESVRA--CLEDR-FTYK----YE-EIYSKFIIFII------------ETV-------VI-GFDP---H---------------------------------------------------------------------------------------------------------------------------------------------------------------

>Amphioxus_Gb7

-----------------------------------------------------------------------------------------------------------------------------------------------------------------------------------------------------------------------------------------------MSLSAADKKLVQESWDKV-SKPSFAD---AGERVFLKLFQRNESTKAHF----KKFK-----DI-P----S-DQLA---GQAVVRDHGE--KVCKVLDD-FIKG-L---DG-------SGDEAV-KKVGRMHKGLG---MSNEQIDQMKGAIIEVL-A--DAG---FGDAN---YK-GAWGKLWDRFM------------AIH-------RA-AY---------------------------------------------------------------------------------------------------------------------------------------------------------------------

>Amphioxus_Gb8

-----------------------------------------------------------------------------------MS---------------------------------------------------------------------------------------------------------------------------------------------TDR-------SAVVSLTEGEKATIRRTWAVA--SRDMMG---NGANILLKMFEINPDTKKVF----AKFR-----NI-P----D-NQLQ---STPRFRAHVT--RVMASIGT-VVNS-L---D---DQ--EVLLDLF-KDIGKKHYPAR---VPTEYFDVIAGAILCMLQQ--CLGTG-YTAE----VD-SAWTKLYGSLG------------RHA-------KD-GLRE---A-----AAMGTP----------------------------------------------------------------------------------------------------------------------------------------------------

>Amphioxus_Gb9

-----------------------------------------------------------------------------------MGS---------------------------------------------------------------WWGKP---------------------------------------------------------------------VD-NTP-------DDITGLTANQIQLIRDTWQIV--YKNKRE---NCFAIFRILFTDHPSTKSLF----RLMD-----AV-DLD--VPGEFE---KNVAARAHMV--RFMHSFAT-FMDT-L---D---EP--AELRQLL-YDLGKNHAKHQ---VGPELFDALGPILMKALPI--VLDGK-FTPE----VK-TAWLTAYTFMS------------THL-------KE-GVEE---G-----QRQLA------------------------------D-----------SK---------------------------------------------------------------------------------------------------------

>German_cockroach_GbX

-----------------------------------------------------------------------------------MGNASSH-----------------------------HKAGGV--------------------AEKVPGGDLGKNGAE--------------------------------FEEQEQMRKRSSV---GVESDRD---------V-IPL-------VCPEPLSERQKELLVETWKEL--EQNIAQ---VGVITFISLFETHPDVQQVF----MPFK-----EI-E----L-EELK---HSKQLRAHAL--RVMAFVQK-AVAR-L---H---EP--EKLEKLL-QDLGKKHYAYG---AKQKYVDLIGPQFIQAIQP--SLAEQ-WTPE----LN-TAWVQLFQYMA------------FVM-------KT-SMNE---E-----EQRVR------------------------------A-----------QQ---------------------------------------------------------------------------------------------------------

>German_cockroach_GbXL

-----------------------------------------------------------------------------------MGCELGK-----------------------------LVAAPR------------------------------------------------------------------------------------------GEEEPP--AP-PQA-------DPRLPLTAKQKYTMMASWKGI--SRAMEP---TGVYMFIKLFEEHGELLNLF----EKFR-----EL-R----TREEQA---NSMELQEHAT--TVMTTLDE-GIRS-L---D---NL--DSFFQYL-TQVGASHHRIP--GFKPEYFWKIEKPFLEAVKM--TLEDR-YTEN----VE-NIYKVTIKFII------------ETL-------VR-GYEE---N-----KPAS------------------------------------------------------------------------------------------------------------------------------------------------------

>German_cockroach_HbL1

-----------------------------------------------------------------------------------MGGILSY-----------------------------FLGS--------------------------KDDPS----------------------------------------------------------------------M-DIP-------DKATGLTPRERQIVKDTWALA--YKNSKS---VGVELFIQLFTTYPHHQQKF----PSFK-----NV-P----L-SEMK--LGNKKLEAHAT--NVMYSLAT-LVDN-L---E---DV--ECLIELC-SKIGENHLRRK---VEQQAFLDVKTVLMKLLKE--KLGSS-LTPQ----GE-EAWNKTLDLAN------------KCI-------FQ-AMED---K-----KNKA------------------------------------------------------------------------------------------------------------------------------------------------------

>German_cockroach_HbL2

-----------------------------------------------------------------------------------MGNLLNA-----------------------------LSGS--------------------------GSNAN----------------------------------------------------------------------L-DVP-------DPATGLTPREKNAIRRNWELV--KGDIKQ---NGIDLLMLFFEENPSYQQFF----NSFK-----DV-P----L-KELP---KNPKFHAHCT--SVMYALSS-VVDN-L---D---DP--GCLVEML-SKLGENHHRRG---ISRQEFINLKAVVLKLLKT--KLGSK-FTSE----DE-AAWNKTLDVAY------------SVI-------FK-GLDK---A-----EEDAS------------------------------Q-----------KA---------------------------------------------------------------------------------------------------------

>Backswimmer_HbL

-----------------------------------------------------------------------------------M--------------------------------------------------------------------------------------------------------------------------------------------------------PKAFSMTDREVEVINQSWNQI--KAQELV---VGLQMFKLLFQRYPQYERLF----THLH-----QS-G------KSLY---EGDRFQHHVVR-NIMSSINK-VIEQ-L---N---SA--DSAPRTL-QEMGVRHKKLD---VHRKHFESFVPFVVDAMVN--VRMSM-DKDE----VA-SAWTKLMDAIA------------SNL-------SK-GVES-------------------------------------------------------------------------------------------------------------------------------------------------------------------

>Silkmoth_GbXLa

-----------------------------------------------------------------------------------MGCQLGK-----------------------------LAASER------------------------RGNNAG-------------------------------------------------------------PVTDG--PP-PAT-------DPRLPLTAKQKYSMLASWKGI--SRAMEK---TGICMFIKLFEENQDLLDMF----EKFR-----QC-K----TKEEQI---NSMELAEHAN--NVMNTLDE-GIKG-L---D---DL--DNFFQYI-HQVGASHRKIP--GFRVEYFWKIEAPFLAAVES--TLGDR-YTPN----VE-NIYKITIKFIL------------ETL-------IE-GYEK---A-----GNNPS------------------------------S-----------AS---------------------------------------------------------------------------------------------------------

>Silkmoth_GbXLb

-----------------------------------------------------------------------------------MGCELTK-----------------------------MIKSE----------------------------------------------------------------------------------------PHDLMNQPP-----PPS-------DPRCPLTTKQQYCMLASWKGI--FRQIEK---TGVLLFIKLFEENEDLLHLF----EKFQ-----EL-R----TTEDLS---QSEELAEHAN--KVMHTLDE-GIKG-L---G---DI--DTFLAYI-QHVGATHHQVP--GFKAENFWKIEQPFLQAAKT--TLGER-YTAN----VE-NIYKLTIKFIL------------ENL-------VK-GYED---S-----AGKEIG-----------------------------N-----------NETT-------------------------------------------------------------------------------------------------------

>Silkmoth_HbL

-----------------------------------------------------------------------------------MGTWFSY-----------------------------M-----------------------------WWGGD----------------------------------------------------------------------P-DVV-------NPVSGLTRREIHAVQKSWAPV--NANSFA---TGSELLRRLFNTYPDTKEYF----KMVR-----KL-P----E-EEYS---QNPQFKAHVI--NLMTSLNL-AVNN-L---N---QP--EIVAAMM-TKLGESHRRRQ---IKEKNFHELKEVIVKLFID--VLR---LDDA----TL-SAWGKTVEFWY------------KHI-------FV-TLNS---P-----EETR------------------------------------------------------------------------------------------------------------------------------------------------------

>Blue_winged_olives_HbL

-----------------------------------------------------------------------------------MSGIVNT--------------------------ILGLFGL--------------------------RQNAG----------------------------------------------------------------------L-DVE-------DPATGLTLRQKGLVRSTWALV--MPNIRT---VSVDVLITFFETFPQYYPLF----TSFA-----GL-E----V-TDLR---SNKKFTAHAT--TVFHALAS-LVDA-L---D---DT--EVLVELC-EKVGKAHISRK---VPPGAFDDLKVTALKVLST--KLGNK-LSPA----AA-EAWDKTLTAAF------------SLI-------KK-ELPA---E-----DQE-------------------------------------------------------------------------------------------------------------------------------------------------------

>Silverleaf_whitefly_GbX

-----------------------------------------------------------------------------------MGNTGTT-----------------------------RRNSLF--------------------TKQDSSSSEVGSTPG-------------------G-----------GGGRKMTITSRQST---LKEEE-----------N-ELG-------PPPKPLTDAQKSMLVDTWKAL--ENDIAK---VGVITFISLFETHPDVQQVF----MPFN-----GI-E----L-EDLK---HSKQLRAHAL--RVMAFVQK-AIAR-I---N---EP--EKLDTLL-KDLGRKHYSYG---AKVKYVDLIGPQFIQAIQP--SLKDR-WNEE----LH-QAWACLFQFMA------------YIM-------KN-AMLQ---E-----EAAQK------------------------------S----------------------------------------------------------------------------------------------------------------------

>Silverleaf_whitefly_HbL

-----------------------------------------------------------------------------------------------------------------------------------------------------------------------------------------------------------------------------------MP-------DMDEGLSQTEIDLIRESWQPF--AKDLQE---TGITFFLAFFKRQADYQEAF-----PFR-----GV-P----L-SELR---QNESFRRHAK--AVLQFIDT-AIAS-L---E---NT--SEILSML-ESNGKSHGRKN-LGLTWSHYEHLEFTLLDVIDE--FYEQE-KRPL-SS-LEKETWAKFIRSVT------------SGI-------YK-TVQI---H-----QNAIQ------------------------------S----------------------------------------------------------------------------------------------------------------------

>Bumblebee_GbXL

-----------------------------------------------------------------------------------MGCELSK-----------------------------LAT--------------------------------------------------------------------------------------------------P--NQ-PTT-------DPRLPLTAKQKFTVMASWKAV--SRKLET---TGVFMLMRLFEENEELVQMF----SRFL-----DL-K----SKEERF---DMVELGKHAE--KVMAALDE-GIRG-L---D---NM--DDFLTCL-HQVGATHTKIP--DFNPQYFWKIEQPFLEAVKR--TLEDR-YSEN----VE-STYKVTIKFII------------ETL-------ID-GFDK---A-----QNDKA------------------------------Q-----------SGTAKS-----------------------------------------------------------------------------------------------------

>Bumblebee_HbL1

-----------------------------------------------------------------------------------MGTFLRF-----------------------------FG----------------------------FSSSD----------------------------------------------------------------------D-NRI-------DEATGLTEKQKKLVQNTWAVI--RKDEVA---SGIAVMTTFFKTYPEYQRYF----SAFA-----DV-P----F-DELP---ANKRFQAHCV--SVITALNS-VIDS-L---H---DP--GLMEASL-ISLGERHKRRG---QTKEEFENLKGVVLKVLSQ--ALGKQ-YTPE----VA-EAWSKTLDGVF------------AKI-------YQ-VFSS-------------------------------------------------------------------------------------------------------------------------------------------------------------------

>Bumblebee_HbL2

-----------------------------------------------------------------------------------MGSVLTY-----------------------------F-----------------------------LGNPD----------------------------------------------------------------------D-DVV-------DPKLGLTNKEKRIIRETWGVL--RANSVK---VGVDIMISYFKRFPQHHRAF----PPFK-----DI-P----A-DDLL---DNKKFHAHCQ--GIMSTLND-AIDA-L---D---DV--DLMNAIL-HTTGKRHGRRG---QGRQEFIDLKGVVLDAMRG--AFGSK-FTTE----VE-VAWDKAIDVLF------------SKI-------FE-GEDM---I---------------------------------------------------------------------------------------------------------------------------------------------------------------

>Mediterranean_fruit_fly_Glob1

-----------------------------------------------------------------------------------------------------------------------------------------------------------------------------------------------------------------------------------------------MALNAEDIAEIKKTWAIP--VATPTD---SGAAILIRFFTKYPSNLEKF----P-FR-----DV-P----V-AELN---NSARFRAHCG--RIIKTFDQ-SISQ-LGEEG---GL--EKIQDIW-QGIASSHVQRH--NIPKPSYFELREAIVEVLSE--ACN---LNER----QA-EAWNKLLDIVY------------DII-------FK-KYDD---L-----GAQ-------------------------------------------------------------------------------------------------------------------------------------------------------

>Mediterranean_fruit_fly_Glob2

-----------------------------------------------------------------------------------MPP----------------------------------------------------------------------------------------------------------------------------------------IFPKALEPYDMKPVENELGLTITERRSLQNGWSII--KQKQRR---AALTIYVNLFTEHENLYEVF---R-----------------S-DGVL---NIEFASQHQK--EVLTVFQM-IIEQ-V---D---NA--RFVKTML-KELALRHEAAS---VTNTQWQLYTNEVRKYFLE--TLADA-ISPT----FV-HALDKLMNFVCNF----------NDL-------TE-SKEE--------LHRVTRI----------------------------------K-----------------------------------------------------------------------------------------------------------------

>Mussel_shrimp_HbL1

-----------------------------------------------------------------------------------MAAVIAP------------------------------------------------------PPVVKEGDSA--------------------------------------------Y---------------------------NTP-------CTKSGLTPHQKAAMRESWSKL--SADKRT---NGTDFFAQLITRYPEYQKFF---GRQVA-----GM-S----P-DQFR---SSRKLQAHSM--QFMHGVSN-LLDN-V---D---DM--DAFGELM-DKMALRHRPFPEGKLGKDDFDKATNLLIEVLLG--HSDVK-SCKDNAEFLK-EAWSKAFEVIN------------SQF-------AS-------------------------------------------------------------------------------------------------------------------RM---------------------------------------------------

>Mussel_shrimp_HbL2

-----------------------------------------------------------------------------------MATKIAA------------------------------------------------------PSCAGEGDAG--------------------------------------------Y---------------------------NNP-------CPRTGLTPHQKAGLRESWLKL--ATDMRS---NGTTFFAQLITRYPEYQKFF---ARQVG-----GL-S----P-DQYT---NSRKLQAHSM--QFMRGVTN-LLDN-L---D---DL--DALGELM-DKMALRHKPFPEGKLGKDDFNKATNLLIEILLA--NPGVQ-NCRDNAEFLK-DAWTKTFELVN------------GQF-------AA-------------------------------------------------------------------------------------------------------------------RM---------------------------------------------------

>Mussel_shrimp_HbL3

-----------------------------------------------------------------------------------MGATVST------------------------------------------------------TDQTHANDPA--------------------------------------------W---------------------------NTP-------DSTTGLTPRDKDILRKSWGVA--AQDFRG---NGTEFFYQFFKRYPKYIDTF----KALR-----GL-T----A-DEAK---ANKRLQVHAM--GFMHGVAN-VLEN-L---D---EP--EGLIELL-ERLGRRHAPFE---LKKEDMENATDLFSELLLTHPNLPTIEYSKDD---VA-STWEKAFKVIN------------TVISL--IDWSD-IMAQ-----------------------------------------------------------------------------------------------------------------------------------------------------C-------------

>Sea_louse_HbL

----------------------------------------------------------------------------------------------------------------------------------------------------------------------------------------------------------------------------------------------MSILTSNELSLISESWKLV--VPDLEH---HGLSFFLKLFEEYPTYQEKF------FP-----EL-H---------Q---DERKIQRHGA--IVLKSVGK-LVAF-L---EA-NKV--IALVDAI-KRLATNHSRRG---VLREQFYPACRILLEYLAQ--ALGTH-LSTE----GA-LAWKRFLGTFV------------ELM-------QE-GYAQ---L-----DASK------------------------------------------------------------------------------------------------------------------------------------------------------

>Bark_scorpion_GbX1

-----------------------------------------------------------------------------------MGCVPAK-----------------------------SVS---------------------------------------------------------------------------------NLPYGL---------------G-GFK-------GGVPFVTYQQKVALVQTWNVL--MENLSR---VGVIAFMRLFETHPDVQEIF----IPFK-----GL-D----H-ESLR---NSKELRAHAL--RVMSFVQK-VVAR-L---E---QP--RKLEMLL-GELGKSHLNYG---AKAEYIEKIGPQFIYAVKP--MLEDH-WNPG----VE-NAWLQLFRYIT------------HYM-------KV-TMER---S-----DKETV------------------------------D-----------DD-------------------------------------------------------NRKKNKMKI-----------------------------MRHSFRGK-LYK

>Bark_scorpion_GbX2

-----------------------------------------------------------------------------------MGCKYTK-----------------------------TVNANL----------------------------------------------------------------------KKKEANKKNSPL--------------------LK-------DEKPDLTNQQKALVLNTWKLL--VENISR---VGVITFMSLFETHPDVQEVF----MPFR-----DL-T----H-EELS---RSTDLRAHAL--RVMGFVQK-IVAR-L---D---EP--EKAEQLL-GDLGKKHVMYG---AKPDYVDLIGPQFVYAVKP--SLEDH-WTNE----IE-EAWLQLFRFIA------------YCM-------KE-SMTE---C-----SRPAQ------------------------------Q----------------------------------------------------------------------------------------------------------------------

>Bark_scorpion_GbXLa

-----------------------------------------------------------------------------------MGCRFGK-----------------------------TVPSVK------------------------DQNDQ---------------------------------------------------------------TDPP--PP-APT-------DPRIPLTAKQCFSISKSWKGI--ARAMEP---TGINMFVKLFQDNEDLLDLF----EKFQ-----SL-K----ISESNF---ESMELAQHAS--IVMSTLDE-SIRS-L---D---NV--DYLLDYL-HSVGKLHHKIP--GFQREYFWRIEKPFLAAVQE--TLGDR-YTEN----ME-TIYKITIHFIL------------ETV-------IA-GYDM---G-----PETNQ------------------------------N-----------SL---------------------------------------------------------------------------------------------------------

>Bark_scorpion_GbXLb

-----------------------------------------------------------------------------------MGTSLSK-----------------------------GSYGFS------------------------KKGQDG----------------------------------------------------------ISPLDDPP--IP-AAP-------DPRLPLTARQRFSISKSWKGI--SRAMES---TGINMFIKLFEDNEDILHLF----KKFQ-----YL-K----THEQQR---DSMELAQHAS--IVMSTLDE-GIRS-L---D---NM--DYFLDYL-HSVGKLHRKIQ--GFNRDLXXKIEKPFLSAVQE--TLGDR-YTDN----MD-SIYKVTIRFIL------------ETV-------IK-GYDM---A-----EDETN------------------------------G-----------TPSEENDV---------------------------------------------------------------------------------------------------

>Bark_scorpion_HbL1

-----------------------------------------------------------------------------------MGSSWST-----------------------------LLVS--------------------------KSDTS----------------------------------------------------------------------A-DSV-------DPATGLTKKEKDGIKYTWDIV--RKDIPK---NGVALFIMFFKTNPDHQKVF----TSFA-----DV-P----L-SELP---KNKKLMAHAS--SVLYSISS-LVDS-L---D---DV--ECLKEMV-IKIAHNHLRRK---VDDKHFSSLGESIISFMEE--KLGSK-FTSH----K--EAWQKFYSVVV------------TIV-------KE-VQEE---E-----HYES------------------------------------------------------------------------------------------------------------------------------------------------------

>Bark_scorpion_HbL2

-----------------------------------------------------------------------------------MGCNVGR------------------------------------------------------------------------------------------------------------------------QTV---------LKGKTEE-------QLLANLTSRQIELVTETWQIV--SQDMAN---VGVIIFXXLLTQHPELCKLF----KKFM-----TL-K----E-DGTYD-WDLGGMERHAL--LVMQALEA-AIDN-L---D---DS--RVLSGIL-FELGCKHARYN---VQEDMFDKLWDALKSGLEE--TLQEQ-MTKE----VT-QAWFSVFRYIS------------HHI-------VK-GMRD---Y-----RKRSQ-----------------------------------------------------------------------------------------------------------------------------------------------------

>Bark_scorpion_HbL3

-----------------------------------------------------------------------------------MGCSVGR------------------------------------------------------------------------------------------------------------------------QSV---------LKGNIEP-------QLSKNLTPQQIQLVRSTWSIV--SQDMVG---VGTIVFQRFLTRHPELCKMF----RKFM-----TL-K----D-DGTYD-WDLEELQRHAL--LVMQALEA-AIDN-L---D---DS--RVLAGIL-YDLGRRHARYN---VQEQMFDKLWEALRYGLEK--SLQNR-MTRE----VN-QAWFAVFKYIS------------WQV-------IK-GMRD---T-----YAKGID----------------------------------------------------------------------------------------------------------------------------------------------------

>Carpenter_ant_GbXL

-----------------------------------------------------------------------------------MGCELSK-----------------------------LATTKS------------------------RNQTGN------------------------------------------------------------DGSSPP--PP-AAT-------DPRLPLTARQKFTVIASWKAV--SRALEP---TGVYMFIRLFEENAELLNMF----TKFR-----DL-K----TKEQQS---TSMELAEHAK--TVMSTLDE-GIKS-L---D---DM--DAFLTYL-HEVGASHTKIP--GFNRQYFWKIEKPFLDAVER--TLEDR-YSEN----VE-NIYKLTIKFII------------ETL-------ID-GFDK---A-----QNDKA------------------------------K-----------S----------------------------------------------------------------------------------------------------------

>Carpenter_ant_HbL

-----------------------------------------------------------------------------------MAWFRGL-----------------------------F-----------------------------NFFLD----------------------------------------------------------------------D-NKL-------DEKIGMTEKQKRLVQNTWAIA--RKDEVS---AGVAIMIALFKQYPEYQKQF----KPFK-----DV-P----I-DELP---KNKRFQAHCV--NIISAISK-LIEQ-M---C---DP--ELMQATL-INLIEKHKNRG---QTQEQFENLRQLLAKLFPS--LFGKQ-YTQE----AE-EAWKKLLDLMY------------SVI-------HE-VYKN-------------------------------------------------------------------------------------------------------------------------------------------------------------------

>Sea_squirt_Gb1

-----------------------------------------------------------------------------------------------------------------------------------------------------------------------------------------------------------------------------------------------MPFTDEELKLLRDSWDEV-KKLGMKE---VGLHIFTGLLNAAPSLRTLF----YTIDLPDEEEL-T----I-DVMR---ENKKVVAHAT--RIANAISK-FIKF-L---D---QP--DELEKLL-TSLGESHARRQ---VDPESFEYVAPVILSVIGG--HLKLP-SNSP----TL-QAWVKAYGVLR------------NGI-------VS-AMEA-------------------------------------------------------------------------------------------------------------------------------------------------------------------

>Sea_squirt_Gb2

-----------------------------------------------------------------------------------------------------------------------------------------------------------------------------------------------------------------------------------------------MGLTTEEIGLLRSSWNEM-KTIGMKE---LGLLIFHRLFSDVPRIRKMF----YNLELPDDETL-T----M-EAMR---SNQKMSRHAT--RIATSIST-YLKL-A---D---QP--EELKTFL-NGLGELHAGHN---VEPEDFEYLAPVMLAVIGG--QLNLN-SNSS----IL-QAWVKAYGVLR------------NGI-------VR-GMYA--YQG--------------------------------------------------------------------------------------------------------------------------------------------------------------

>Sea_squirt_Gb3

-----------------------------------------------------------------------------------------------------------------------------------------------------------------------------------------------------------------------------------------------MSLTSEQVVLLRSSWQTI-GKLGMSN---VGLAVLHRLFNDVPETLPFF----HSVLSP-TQQT-E----I-EVLK---SNAKVVRHAS--RVGLSIDK-IINL-L---D---NG--EELVKYL-LFLGQVHVKRS---IPRKYFSAMGPVLLSVISA--VLEKD-LDAP----VM-QAWATAYGVIE------------QGI-------ID-GM---------------------------------------------------------------------------------------------------------------------------------------------------------------------

>Sea_squirt_Gb4

MKIICGLI-------------------LFSTFAIIFVSGLNCWTCNVLGGNNVCRRSGGLRTCFNNQVCYNEVRRRGNTINIRKGCKNSGVCENH------------------------IMQSMSTPN----------------PNNQCVNGPN------------------YFCSCCCG-------------------NSVCNSN--WLTCVTAGSVAPT-VST-TVP-------PADEGLKRSDIINIQDSWNTL-KGFGYET---VGMLVLHRLFNDAPQTRYLF----SQLS-----LS-SNESFTLEQMR---NNSRVVYHAN--RVARAVGR-LVDL-I---E---LP--TNFTDHL-VWLGQRHAYHG---VAPVNFDYMGPVLLETIKV--NLELP-SDSP----TL-SAWAKAYGVIK------------NGI-------KD-AIIA---T-----YAEG------------------------------------------------------------------------------------------------------------------------------------------------------

>Bed_bug_GbX

-----------------------------------------------------------------------------------MGNAAPG-----------------------------RRNSML--------------------SKEGSVNEA----------------------------------------------SRQNT---LTDAD-------------QQG-------PQPRALTKEEMDHLTRTWKLL--EDDIAK---VGVITFISLFETHPDVQQVF----MPFN-----GI-E----L-EDLK---HSKQLRAHAL--RVMAFVQK-AIAR-L---H---EP--EKLEQLL-KELGKKHHGYK---AKVQYVDLVGPQFIQAIQP--SLDSE-WTEE----VA-DAWKLLFAHVG------------YIM-------KG-AMIE---A-----AEEAA------------------------------K-----------ESK--------------------------------------------------------------------------------------------------------

>Bed_bug_GbXL

-----------------------------------------------------------------------------------MGCELGK-----------------------------LTRISG------------------------SGNSEG--------------------------------------------------------------REPPPSAP-APS-------DPRLPLTAKQKYSMVASWKGI--SRAMEQ---TGVFMFIKLFEEHQELLDLF----AKLK-----EL-R----TKEEQE---KSLELAEHAT--KVMATLDE-GIKE-L---D---DL--DTFFTFL-TQIGQTHKKIP--GFKPDYFWKIEKPFLEAVKM--TLGDR-YSEN----VE-SIYKVTIKLII------------DTL-------VK-GYNS-------------------------------------------------------------------------------------------------------------------------------------------------------------------

>Green_crab_GbXL

-----------------------------------------------------------------------------------MGCHITK-----------------------------NKKNKE------------------------TEEVKV-----------------------------------------------------------IDLPEPP--EP-PPP-------DPRLPLTARQRFNIIKSWKGI--ARAIEP---TGVNMFVKLFENHSELITFF----TKFR-----QL-R----TRDEQA---ESLELAEHAT--IVMNSIDE-GIKA-M---D---NV--DFFFDLL-HQIGASHRKIP--GFKKEYFWKIEHPFLEAVRL--TLGDR-YTDN----MD-NIYRITIKLLI------------ETV-------VR-GYEL---A-----ELKEP------------------------------N-----------DNV--------------------------------------------------------------------------------------------------------

>Green_crab_HbL

-----------------------------------------------------------------------------------MGAVLSV-----------------------------VWGWLSPGTQ---VG-------AVTFPEEGSLGPE----------------------------------------------------------------------A-DVP-------DKTTGLTLRHRTAIYRTWDLV--RPNPKL---HGINLFLTMFQEEPVLQTRF----KGFA-----GK-S----I-EELK---NSKRLAAHGT--TVVMAITA-MVDN-L---E---DV--SVLVELL-KNTGANHRDRG---VPKGDFELLAPVLVRFLKD--NLGSA-WSPV----AE-EAWTQAMKVIN------------AVI-------FT-SYDA-------------------------------------------------------------------------------------------------------------------------------------------------------------------

>Culex_mosquito_Glob1a

--------------------------------------------------------------------------------------MFNS----------------------------------------------------------------------------------------------------------------------------------------EHHEESVLNSPDDTGLTNHQKAALVGAWSLV--KQDMVS---HGVNVFIRLFEEHPKYLEYF----DFSQ-----DD-S----A-EELR---ENKSLHAHAL--NVMHLIGA-LIDYGL---D---NP--LMFKCSL-SKMMKNHKKHG---VNKEDVTIVCGIIMEYCLE--ALDQR-GSTT----LE-EAFSSFMKSIA------------DTF-------DE------------------------------------------------------------------------------------------------------------------------------------------------------------------------

>Culex_mosquito_Glob1b

-----------------------------------------------------------------------------------MNEIYET----------------------------------------------------------------------------------------------------------------------------------------EQGEEIVASIPDETGLNNHQKVALIGAWSLV--KKDIIS---HGRNIFVRFFEEHPQYLNYF----DFSQ-----DK-T----A-SEIG---ENKSLHAHAL--NVMHFIGT-LIDYGL---H---NP--LMFKCSL-SKMMKNHKKHG---VHKKDVTIVCEVIMKYCLE--VLDQH-HSTT----LE-AAFKSLMRSIA------------DTF-------DE------------------------------------------------------------------------------------------------------------------------------------------------------------------------

>Culex_mosquito_Glob2

--------------------------------------------------------------------------------------------------------------------------------------------------------------------------------------------------------------------------------------------MDETGLTGKQKITLLSAWGLI--KQDLDL---HGRNIMLLIFREHPHFIPYF----DFSA-----DP-N----N-TSLS---ENRALQAHSL--NLIMALGA-LIEYGL---K---TP--KMFECTL-AKLVKNHKTRR---VTSQDVKMFGEVILMYFAQ--VLGRQ-SASS----LP-TAFNRLIEQIA------------EAF-------EA--------AQFT------------------------------------------------------------------------------------------------------------------------------------------------------------

>Midge_GbXL

-----------------------------------------------------------------------------------MGCELGK-----------------------------LASSSS------------------------SKKEIE----------------------------------------------------------NGTVSEPA--AP-APP-------DPRLPFTARQKYTMVASWKGI--SRAIET---TGVNMFIKLFEEHADLLNMF----TKFK-----EL-K----TKEEQA---TSEELAEHAT--KVMETMDE-SIRS-L---D---EI--DVFFQFL-HETGAIHTRIP--GFTSDLFWKIEKPFLKAVSD--TLGDR-YTEN----VE-GIYKITIKFVI------------ETL-------VE-GFER---G-----LQNKN------------------------------H-----------VGNKGVNSTCNSNVNGAN-----------TTTTTTT----QN-----GDTSC-NSTSRSNDAS--------------------------------------------

>Midge_Hb2B

MKFLV------------------------------------------------------------------------------LALCIAA--------------------------------------------------------------------------------------------------------------------------------------------------AVAAPLSADEASLVRGSWAQV--KHS-------EVDILYYIFKANPDIMAKF----PQFA-----GK-D----L-ETLK---GTGQFATHAG--RIVGFVSE-IVAL-M---GNSANM--PAMETLI-KDMAANHKARG---IPKAQFNEFRASLVSYLQS--KVS---WNDS----LG-AAWTQGLDNVF------------NMM-------FS--------------------------------------------------------------------------------------------------------------------------------------------Y------------------------L--

>Midge_Hb3A

-----------------------------------------------------------------------------------MVA-----------------------------------------------------------------------------------------------------------------------------------------------------TPAMPSMTDAQVAAVKGDWEKI--KGS-------GVEILYFFLNKFPGNFPMF----KKLG----NDL--------AAAK---GTAEFKDQAD--KIIAFLQG-VIEK-LG--S---DM--GGAKALL-NQLGTSHKAMG---ITKDQFDQFRQALTELLG---NLG---FGGN-I-----GAWNATVDLMF------------HVI-------FN-ALDG---T---------------------------------------------------------------------------------------------------------------------------------PV----------------------------

>Midge_Hb6

MKFLV------------------------------------------------------------------------------LALCIAA--------------------------------------------------------------------------------------------------------------------------------------------------ASAAVLTTEQADLVKKTWSTV--KFN-------EVDILYAVFKAYPDIMAKF----PQFA-----GK-D----L-DSIK---DSAAFATHAT--RIVSFLSE-VISL-A---GSDANI--PAIQNLA-KELATSHKPRG---VSKDQFTEFRTALFTYLKA--HIN---FDGP----TE-TAWTLALDTTY------------AML-------FS-AMDS-------------------------------------------------------------------------------------------------------------------------------------------------------------------

>Midge_HbE

MKFII------------------------------------------------------------------------------LALCV----------------------------------------------------------------------------------------------------------------------------------------------------AAASALSGDQIGLVQSTYGKV--KGD-------SVGILYAVFKADPTIQAAF----PQFV-----GK-D----L-DAIK---GGAEFSTHAG--RIVGFLGG-VIDD-L-----------PNIGKHV-DALVATHKPRG---VTHAQFNNFRAAFIAYLKG--HVD---YTAA----VE-AAWGATFDAFF------------GAV-------FA-KM---------------------------------------------------------------------------------------------------------------------------------------------------------------------

>Orchid_beetle_HbL

-----------------------------------------------------------------------------------MGGLLSY-----------------------------FYGQ-----------------------------------------------------------------------------------------------------S-NEP-------DPATGLTPREKSLVVNSWAIV--RKDMIG---NGTELFILFFTKFPKYLTYF-----PFR-----DV-P----F-DQLR---ENKKVHAHAV--NVMYALSS-IVDN-L---S---NV--EVLVNLL-SKTGEAHGRRK---IPEQSFGDLKVTVLELLRV--GLGSK-ITDE----GI-DAWNKTLEVAI------------KVI-------HD-GMKE---Y-----YQKQQ------------------------------Q-----------KKAAAE-----------------------------------------------------------------------------------------------------

>Asian_citrus_psyllid_GbX

-----------------------------------------------------------------------------------MGNTGTT-----------------------------RRNSLF--------------------LKQESGDEPPSHKKE-------------------GVTHSIARRLTLSGGKGLGLGSRQNT---LLEEEIVPP-------C-VNS-------PPPEPLTEDEKKLLIETWKIL--EDDIAK---VGVITFISLFETHPDVQQSF----MPFN-----NI-E----L-EDLK---HSKQLRAHAL--RVMAFVQK-AIAR-L---H---EP--DKLDTLL-RDLGKKHYTYG---AKAKYVDLIGPQFISAIQP--SLESR-WSPE----LN-NAWIHLFGYMA------------HIM-------KE-SMTA---E-----ELLNR------------------------------K-----------S----------------------------------------------------------------------------------------------------------

>Asian_citrus_psyllid_HbL

-----------------------------------------------------------------------------------M--------------------------------------------------------------------------------------------------------------------------------------------T-TYE-------QLVSNISDDRLQAVVLSLDII--KPDIND---FGTKVFKTLFKEHPEYQSQF----PKLK-----DI-P----Y-DKLD---ANKSFTHHVN--AVVLAIAN-SVVN-L---K---NP--NAVLPEL-EKLGTSHQRRN---IRPEQFEVVTNIILKVLKE--KVSDP----Q----VL-KTWQEILTILA------------NTI-------VS-FMKP---T-----IK--------------------------------------------------------------------------------------------------------------------------------------------------------

>Fruit_fly_Glob1

-------------------------------------------------------------------------------------------------------------------------------------------------------------------------------------------------------------------------------------------------MNSDEVQLIKKTWEIP--VATPTD---SGAAILTQFFNRFPSNLEKF----P-FR-----DV-P----L-EELS---GNARFRAHAG--RIIRVFDE-SIQV-LGQDG---DL--EKLDEIW-TKIAVSHIPRT---VSKESYNQLKGVILDVLTA--ACS---LDES----QA-ATWAKLVDHVY------------GII-------FK-AIDD---D-----GNAK------------------------------------------------------------------------------------------------------------------------------------------------------

>Fruit_fly_Glob2

-----------------------------------------------------------------------------------MSQISKL-------------------------------------------------THISRISQNNQSDGSDEDKFRR----------ANFP-----------------------------------------------VYPKPLPDRDLSYKADENEFTMVEKASLRNAWRLI--EPFQRR---FGKENFYSFLTRNEDLINFF----RKDG-----KI---------------NLSKLHGHAM--AMMKLMSK-LVQT-L---D---CN--LAFRLAL-DENLPTHLKNG---IDPDYMRMLATALKSYILASSVIENH-NSCS----LS-NGLARLVEIVGEY----------AVV---DEARKR-AMST---ALRTTVDDAGNRIV---------------------------KVAL-------------------------------------------------------GT----------------------------------------------------------

>Fruit_fly_Glob3

M----------------------------------------------------------------------------------MSEEVIA--------------------------------------------------------------------------------------------------------------------------KNISLSSL--TYPKRIPKIKFGPIKDEMGFTLSERLALRQAWNLV--RPFERR---YGQDVFYSFLNDYYWGIKKF----RNGA-----EL---------------NVKALHSHAL--RFINFFGL-LIEE-----K---DP--VVFQLMI-NDNNHTHNRCH---VGSVNIGHLAQALVDYVLK--VFHKV-SSPS----LE-QGLSKLVEKFQNYQDQQSNTSGYNRL-------SKVNFDS-------------------------------------------------RP----PRGNP-------------------------------------------------------------------------------------------------------

>Monarch_butterfly_GbXLa

-----------------------------------------------------------------------------------MGCQLGK-----------------------------LAASER------------------------RGNNQD--------------------------------------------------------------LGDG--PP-PAT-------DPRLPLTAKQKYSMLASWKGI--SRAMEK---TGICMFIKLFEENQDLLNMF----EKFR-----QY-R----TKEEQI---NSMELAEHAN--NVMNTLDE-GIKG-L---D---DL--DNFFEYI-HQVGASHRRIP--GFKVEYFWKIETPFLAAVES--TLGDR-YTPN----VE-NIYKITIKFIL------------QTL-------VE-GYEK---A-----GKSNA------------------------------N-----------TT---------------------------------------------------------------------------------------------------------

>Monarch_butterfly_GbXLb

-----------------------------------------------------------------------------------MGCKLSQ-----------------------------LASSEF---------------------------------------------------------------------------------------SHDPFEKPP-----PPS-------DPRSPLTAKQQYCMLASWKGV--FRQVEK---TGILLFVKLFEENEELLHLF----EKFR-----EL-R----TKEAIV---SSAELAEHAT--QVMHTLDE-GIKG-L---A---DM--DSFFTYV-RHVGGTHRQVP--GFKAENFMKIEQPFLEAAKT--TLGER-YTPN----IE-NIYKLTIRFIL------------ENL-------VK-GYEE---A-----GEENG------------------------------T-----------TQT--------------------------------------------------------------------------------------------------------

>Monarch_butterfly_HbL1

-----------------------------------------------------------------------------------MGGLLNR-----------------------------M-----------------------------WWGGD----------------------------------------------------------------------P-DGV-------NPVSGLSRRDVFAVQKSWAIV--YANPLA---NGSELLKRYFRAHPESKEFF----RMLR-----KL-N----E-NEFD---DNHQFKAHVM--SLMSSLNL-AITN-L---D---QP--EIVVAMM-NKLGESHGRRK---IDEQNFHNLKGIIVKMFID--VLK---LDDK----NL-ASWGKAVDFLY------------KHI-------FV-TLKP---E-----S---------------------------------------------------------------------------------------------------------------------------------------------------------

>Monarch_butterfly_HbL2

-----------------------------------------------------------------------------------MGGIVSR-----------------------------I-----------------------------WWGGV----------------------------------------------------------------------P-DAV-------QAYSGLSHRDIYTVQKTWAVV--YCNAAE---NGIEIFKRLFHANPETKNFF----INFR-----NL-S----D-EELD---KSHQFRAHVI--NLMSSLNL-AITN-L---H---QP--EVTAALM-NKLGESHGKRG---IREEHLLSLKDVMLEMLNS--LLG---LDES----AL-VSWNKTIDFIY------------KHI-------FQ-TLH--------------------------------------------------------------------------------------------------------------------------------------------------------------------

>Monarch_butterfly_HbL3

-----------------------------------------------------------------------------------MGSWLTY-----------------------------L-----------------------------WWGGD----------------------------------------------------------------------P-DLV-------NPLSGLTKREVYAIQQSWAPV--YSNSVA---NGTELLKRLFRAYPETKEFF----KMVR-----KS-S----E-DEFI---GNPQFRAHVI--NLMSSLNL-AVGN-L---N---QP--EVVSAMM-NKLGESHGRRK---IQEKHFHDLKDVIVKMFIE--VLK---LDGT----TL-NAWGKAVDFWY------------KHI-------FE-TLKQ---A-----EIR-------------------------------------------------------------------------------------------------------------------------------------------------------

>Mountain_pine_beetle_GbXL

-----------------------------------------------------------------------------------MGCELGK-----------------------------LASRDN------------------------RNGARK-------------------------------------------------------------FEEPT--PPQVQV-------DPRLPLTAKQKYNMMASWKGI--SRAMES---TGVCMFLKLFEEHSELLLLF----EKFK-----SL-K----SKEDQA---TSLELAEHAT--TVMSTLDE-GIKG-L---D---DL--DTFFEYL-NQVGASHRRIP--GFKAEYFWXXEKPFLEAVET--TLGDR-YTSN----VE-NIYKITIKFII------------ETL-------VK-GFEN---A-----NAT-------------------------------------------------------------------------------------------------------------------------------------------------------

>Mountain_pine_beetle_HbL1

-----------------------------------------------------------------------------------MGTVLSY-----------------------------ITP---------------------------ANSGR----------------------------------------------------------------------S-DDP-------DPVTGLTSKEKYLVRTSWAKI--MKNPAD---SGVALLCLLFERHPEYVQLF-----PFS-----DV-P----P-SEFK---TNVRFRAHAN--SVVYALSS-IVDA-L---N---DN--NLLVQIL-TKTGSSHVPRH---VTADAFIHLKEVTIELFST--I-----FKAD----EV-AAWKKTFEVAF------------SVI-------IQ-GIES---V-----N---------------------------------------------------------------------------------------------------------------------------------------------------------

>Mountain_pine_beetle_HbL2

-----------------------------------------------------------------------------------MGIITSY-----------------------------F----------------------------GRELER----------------------------------------------------------------------D-DDP-------DPKTGLTSRYISVLKTTWKRITERGGTLE---IGTAIFTNLFEKHPEYQQLF-----PFK-----NL-R----R-EELK---TSNKFRAHCI--SVMYALTC-IVEN-V---S---EP--LILEQLL-IKQSTSHVLRN---VPDQAYWDIKTVILSIVAS--S-----MNPS----EV-FVWEKFLKFAF------------RIM-------VA-TAEE---T-----RNN-------------------------------------------------------------------------------------------------------------------------------------------------------

>Water_flea_Hb1-1

MQFLKIALF---------------------------------------------------------------------FAFVALASSSSS----------------------CSQAPGTTITSVTTTVT---------------------------------------------------------------------------------------------TVTADED-------SDSGLLSSHDRSIIRKTWDQA--KKD-GD---VPPKILFRFIVANPEYQKMF----KSFA-----TV-P----Q-NELL---GNGNFLAQAY--TILAGLNV-VIQS-L---S---SQ--ELLANQI-NALGGAHQPRG---ATPIMFEQFGAITEEVLAE--ELGIA-FNAE----AR-QAWKNGMRALV------------AGI-------SK-NLKK---A-----EDLA------------------------------------------------------------------------------------------------------------------------------------------------------

>Water_flea_Hb1-2

--------------------------------------------------------------------------------------------------------------------------------------------------------------------------------------------------------------------------------------------DPQTKLTPHQIHDVQRSWENI--RANRNS---LVSAIFVKLFKETPRVQKHF----AKFA-----NV-A----V-DSLP---GNADYEKQVA--LVADRLDT-IISA-M---D---DK--LQLLGNI-NYMRYTHQPPR--AIPRQTFEDFARLLIDGLTA-----SG-VSGD----DM-DSWKGVLTIFV------------NGVS-----PKQ------------------------------------------------------------------------------------------------------------------------------------------------------------------------

>Water_flea_HbA

----------------------------------------------------------------------------------------------------------------------------------------------------------------------------------------------------------------------------------------------MDVLNSVNVAAVQSTWAVI--KSDINT---FAPQFYVALLTAHPEYQAMF----PTIA-----NV-P----S-GQLL---NNAALITLSV--NVVTKLSE-IIDS-L---G---NP--GALNGKL-VDLANQHKQRG---TTRAHFDNMATVLLGFLAA--TLGSA-FTPE----AK-QAWTSTMQGIN------------TVV-------EA-SA---------------------------------------------------------------------------------------------------------------------------------------------------------------------

>Water_flea_HbB

-------------------------------------------------------------------------------------------------------------------------------------------------------------------------------------------------------------------------------------------------MKEADRTLVQGTWRIA--KKN-GN---IAPKAFIRYFKLKPEAQKQF----AAFA-----DV-E----L-ADLP---TNSHFLNQVY--TCLAGLNA-YMEN-L---G---KN--PKQCPHLNSPVFKA--------VKPDDLKLFGEVMFTVMEE--ELGQS-FSTE----AR-KAWKDGLIACD------------VAF-------RK--------SH--------------------------------------------------------------------------------------------------------------------------------------------------------------

>Water_flea_HbC

MTRLILALC----------------------------------------------------------------------ALLSLAVGQSP-YNDRFSGGIGSGSWYRGGGMSGSGGPFVTTTTVTTVLDLST-GMTKTFHSSNNPSSGGSSGGERYSSYGG----------------------------GYGGGEKNKFT----------------------EGSSGTS-------PLMNILSENDISVLVNSWHIL--KKR-SD---FAPKVFMRYFKAKPEAQKLF----SEFA-----NV-S----V-TDLP---NNHDFLNAAY--SCISSLEF-ILPH-L---RF--QH--PERCPAL-TDLKNK--------YSVVDLKRFVPIWMAAMQE--EMGNA-YSNE----VR-DVWKKAFSAFT------------DYA-------ST-------------------P----------------------------------------------------------------------------------------------------------------------------------------------------

>Water_flea_HbD

----------------------------------------------------------------------------------------------------------------------------------------------------------------------------------------------------------------------------------------------MDVLKSVNVAAVQSTWAIV--KADLNT---HAPKFYVALLTAHPEYQPMF----PTIA-----NV-P----A-GELL---NNAALKTLSV--NVLSKLSE-LIDG-M---S---NP--DGLNAQL-VELAKQHKNRG---TTRTHFDNLAKVLVDFLAA--NLGAA-FTPD----AK-QAWTATMQGIN------------TVV-------EA-NA---------------------------------------------------------------------------------------------------------------------------------------------------------------------

>Water_flea_HbE

MSWLILVFC----------------------------------------------------------------------SILSLSAGQSP-FNEGIPGGVESGGY--GDSTMRGSGPFVTTTTVTTILDFNPAGMSRSMRSY-------PSASERSYHYGD---------------------------------------------------------------------------KKFGSFSQKDVDVIVNTWNTL--KRR-GD---FAPKVFIRYFKAKPESQKMF----PAFA-----NV-P----I-TELP---TNHDFLNSAY--TCITSLNY-LIPY-L---KF--DH--PERCPAFPKHLKDK--------YNAVDLKKLGSIWMTAMQE--EMGNA-FTND----VR-DVWKKAVMAVI------------EYA-------SK------------------------------------------------------------------------------------------------------------------------------------------------------------------------

>Water_flea_HbF

MSMFILVLC----------------------------------------------------------------------AVLSLSAGQSILFKEG------------------TQGPFIATTTVTTTFDFNPAGVPRAR----------SSACDRTHYDSD---------------------------------------------------------------------------KKYGSLSQMDVDIIVNSWNIL--KKR-GN---FAPKVFIRYFKAKPESQKLF----PAIA-----NV-S----I-TDLP---TNPDFLNSAF--TCVNSLNY-LIPF-L---KY--DH--PERCPSFPKQIKDN--------YNEVDVKKLGSIWMMAMQE--EMGSD-FTND----VR-DAWKKAVMAVI------------EYV-------SK------------------------------------------------------------------------------------------------------------------------------------------------------------------------

>Water_flea_HbG

MLVHYI-----------------------------------------------------------------------------LTAILVV---------------------F---------------LETRP-GRAECPRGY-----KASSSGQRDPS----------------------------------------------------------------TKSESQR-------ILENFLNERDEATIRSTWNTA--KKN-GN---IGPKTFLRYFELKPEAQKMF----PAFA-----EV-D----H-MKLP---TNEDFLAQAQ--NCVSGLNS-YVEH-L---G---KN--PKNCPFIAKAKGKYH---------HEDLKLLGVTLMGVLEE--ELGKG-FTDE----TK-EAWKKGLRAMN------------EAV-------TK-RPNP---SRR-------------------------------------------------------------------------------------------------------------------------------------------------------------

>Water_flea_HbH

---------------------------------------------------------------------------------------MSH-----------------------------TSPNGSQLLE---DL-------QREQP-ETIASSD----------------------------------------------------------------------D-NPI-------DPVTGLSQRERDYIQQSWHHV--RQDLKA---AGLGFFQAFFKAHPDYQLKF----KKFA-----DV-P----A-DQLA---DNKSFLVHAM--SVMNAVTM-VVDS-L---D---DI--PKLVNEL-KNLGKNHGRHN---IKTENFRNLTVVLVAFLES--ALGSQ-LFPE----DVKQSWIKALDVVV------------GVV-------AT-GLPQ---PS---PDDDAG------------------------------S-----------AM---------------------------------------------------------------------------------------------------------

>Water_flea_HbK

-----------------------------------------------------------------------------------MSKALRF---------------------------------------------------Y-------APGSDRRHH---------------------------------GGSEKKLFEEF----------------------------------GLQRSLSDSDINLIVSSWNFL--KKRLSS---FAPKVFIGYLEARTDSKKMF----PDFA-----HV-N----I-AELA---TNVEFRSRAC--NCVASLNY-IIPH-L---K---RSFPVLQCPAL-KNLKTKYNQHI------DILKSLGIIWVKAMQE--ELDKKIFTDD----VR-VVWKKLFSVLK------------EHV-------SE------------------F-----------------------------------------------------------------------------------------------------------------------------------------------------

>Western_corn_rootworm_HbL

-----------------------------------------------------------------------------------MGSVLSY-----------------------------FLGFF-------------------------VNQGR----------------------------------------------------------------------I-NDA-------NPVTGLTSRDIYLIKNSWNKV--ISQPTE---NGIKFFMRLFEIAPKHKLTF-----PFR-----DV-P----T-EDLP---RNKKFHAHVN--SVMYSISS-IVNS-L---N---DV--DTVAAII-DKIGRNHARRS---VDLQALKDVKRALLDIFAF--------MTTA----EL-AAWNKMLDYFA------------KTA-------IK------------------------------------------------------------------------------------------------------------------------------------------------------------------------

>Mayfly_GbX

-----------------------------------------------------------------------------------MGVKNSK-----------------------------LIEAKC------------------PGAPNSPARKSLEPVPE-------------------------------PAKTPENMTSCCET---------IPIPVLA-PLE-LES-------IGPPPLTEEQCVLLAETWKEL--ESNIAK---VGVITFISLFETHPDVQQTF----MPFS-----GM-G----L-EELK---QSKQLRAHAL--RVMAFVQK-AVAR-L---H---EP--EKLEALL-RDLGQKHYFYG---AKPEYVELIGPQFIQAIRP--SLEYR-WTEE----LQ-DTWLQLFRYIS------------DIM-------MA-AIKQ---E-----SLNHI------------------------------S-----------NNPVEH-----------------------------------------------------------------------------------------------------

>Mayfly_GbXL

-----------------------------------------------------------------------------------MGCELGK-----------------------------LSSKGN------------------------RAGKDT---------------------------------------------------------AKDGNLNPP--AP-APP-------DPRLPLTAKQKYNIIASWKGI--SRAMEP---TGVYMFIKLFEEHEDLLNLF----SKLG-----EL-R----TKEQQQ---SSLELAEHAT--KVMGTLDE-AIRA-L---D---SL--DSLIAYL-EAVGASHRRIP--TFERNHFHRIEKPFLDAVRT--TLGDR-YTDN----VD-VIYQATIKFII------------DSL-------QA-GFDK---A-----EGNPE------------------------------M-----------VNRPPPAHIEDIS----------------------------------------------------------------------------------------------

>Mayfly_HbL1

-----------------------------------------------------------------------------------MGGFLSY-----------------------------LLGSDGG-------------------APQEELPAK----------------------------------------------------------------------W-DEP-------EPATGLTPRQKRAVVDTWALV--QPDLKA---TGIAVLIALFEAHPEHQRLF----SAFR-----DV-P----L-SELR---GSKRFAAHAS--SVMHAIAS-LVDT-L--ED---DT--EVLVELL-TKIGVNHAKHS---VPPHAFSDLQAVILKLFQE--KLGDR-LSPE----AA-EAWDKTLTVAN------------SVV-------TK-QLSA---P-----EPSKP------------------------------Q-----------QQVEM-------------------------------------------------KTEERSQEE--------------------------------------------

>Mayfly_HbL2

-----------------------------------------------------------------------------------MGGLMSR-----------------------------GEPVDVW-----------------------PKGVD----------------------------------------------------------------------P-DLI-------DPETGMTPRDRRVVSRTFAVC--VPKVRE---VAMDIFVTLFTKHPEHQKLF----PQFA-----DL-K----TPEELR---TSKRLTAHAS--TAAHGLAA-IVEC-I---G---DT--ECLTAML-NKLGDNHKRHH---VSPSAFPDFKDVLMEVLKR--HLGAL-FTSE----AE-LSWDRAMNFVN------------RTV-------TA-RLV--------------------------------------------------------------------------------------------------------------------------------------------------------------------

>Mayfly_HbL3

MRFL-------------------------------------------------------------------------------ISCVLSMVF-----------------------------------------------------------------------------------------------------------------------LL-------------ETT------IANPNDLHSEEISGVEDIIKNV-LAPDLAT---HGPALFLELFKLEPGYQKLF----TKFA-----DV-P----M-DSLK---DNAALKAHSV--NILGKFAGCVLEN-I---K---SA--DNMQGCF-KGQAETHKKLN---VGYDHAKVLSKAIQNYLSA--NLPHG-YGGD----VK-TGWNKVLYVMT------------EAF-------K-------------------------------------------------------------------------------------------------------------------------------------------------------------------------

>Elephant_shark_Cygb

-----------------------------------------------------------------------------------MEEAAGR------------------------------------------------------------------------------------------------------------------------------------SEQ-RRP-------DPGQYLSDTDRDIIRQTWSRV--FSCCED---VGVRVLIRFFSKFPSAKQYF----SQFR-----HL-Q----EPQEMQ---HSSQLRQHAR--RVMGAINS-VVEK-L---G---DP--EQVRSVL-ALVGRAHAIKH--KVDPMYFQLLSGVILEVFVE--DYAEY-FTTE----AQ-SAWSQLMALIC------------VQV-------LA-AYTE---LG---WAQNSSV----------------------------------------------------------------------------------------------------------------------------------------------------

>Elephant_shark_GbX1

-----------------------------------------------------------------------------------MGCAISG-------------------------------------------------------PGQYPASGR---------------------------------------------------------------------ED-VVA-------VASLSLSDRQTQLVKETWRLV--QEDIAK---VGIIMFVRLFETHPECKDAF----FLFR-----DIDD----L-QQLR---KSKGLRAHGL--RVMSFIEK-TVAR-L---D---QE--DRLQQLA-LELGKSHFRYS---AAPKYYPYVGNEFICAVQP--ILKEK-WTAE----VE-EAWKGLFHYLT------------SVM-------KK-GYQD---E-----ERGSC------------------------------P--------------------------------------------------------------------REKPKHGPNSV---------------------------------------

>Elephant_shark_GbX2

-----------------------------------------------------------------------------------MGCALSG-----------------------------PEEE----------------------PERASPDGKGS----G----------------------------MGDGSERANESGLGHGE------------TKA--EA-VCS-------RLPLTLSVEQKDLVRQSWERL--HQDIAR---VGIVLFIGLFETHPECKEVF----FRFR-----DI-E----L-QQLK---TRKELQSHGL--RVMSFIEK-SVAR-L---G---QE--EKLEQLI-FDLGRSHQRYN---VDPKYYEFVGKEFIDAVKP--ILKEE-WTTE----VE-GAWKCLFLYLT------------TMM-------KM-GYED---E-----KERGG------------------------------G-----------KRVG---------------------------------------------------DRHQLKVTSPTLTPPSPHKVRL------------------------------

>Elephant_shark_GbY

----------------------------------------------------------------------------------------------------------------------------------------------------------------------------------------------------------------------------------------------MTGITEADKENIHFIWEKL--YENPEE---NGKTIVLRMFTDYPETKMYF----QHFK-----NI-S----TLEEMK---KSPQIKRHGK--IVMSALNK-LIAN-L---D---NG--EELSSLL-AKMAERHINVH--KVDLHNFQIIFNIIIAILEE--TFGNA-FTPE----IR-ETWTKLFGVIY------------ACL-------ES-HYKD---AG---FYP--------------------------------------------------------------------------------------------------------------------------------------------------------

>Elephant_shark_HbA

-----------------------------------------------------------------------------------------------------------------------------------------------------------------------------------------------------------------------------------------------MVLSKTDKALLSSSVGKI----QAQA---TGSDVLARMFASFPQTKVYF----VGFS-----DY----------TA---KGPRVQKHGL--TVMTKIIE-GIQY-L-----------DSLRSFL-DALSAKHAHEL--MVDPVNFGFLGECVLSSLAY--QLPD--FSPE----MH-CAWDKYLCEFA------------YLL-------AE-KYR--------------------------------------------------------------------------------------------------------------------------------------------------------------------

>Elephant_shark_HbB

----------------------------------------------------------------------------------------------------------------------------------------------------------------------------------------------------------------------------------------------MVQWSQAELDVIQGKWAAL----DPEK---FGGKALARMFVVYPWTKRYF----GKFG-----GR----------FKA--SDPVVMEHGA--KVMGKMQV-AAK----------DP--GKIKEIF-EYLSKRHSDTI--HVDPENFKLLGSCMLVEMAM--TKGD--WSPE----IE-AINRKFVDVSI------------AAL-------SR-KYH--------------------------------------------------------------------------------------------------------------------------------------------------------------------

>Elephant_shark_Mb

---------------------------------------------------------------------------------------------------------------------------------------------------------------------------------------------------------------------------------------------------MCDWDLINKVWAKV--EEDLAG---NGQTVLLRLFEEHPETKAHF----PKFK-----DI-P----L-GQLT---SNADVKTHGN--TVFKALGD-VVKQ-K---G---KH--A---SNL-QALATTHINKH--KIPPQNFTLITNVILKVFAE--KFPGE-MTAP----AQ-EAFSKAFKAIC------------SEL-------ED-LYKK---GG---FQS--------------------------------------------------------------------------------------------------------------------------------------------------------

>Flower_thrips_GbXL

-----------------------------------------------------------------------------------MGCELGK-----------------------------LAASER------------------------GGGRGGGGG-----------------------------------------------------DGGGGKDDIP--AP-PAT-------DPRLPLTAKQKYTMLASWKGI--NREMEA---TGVNMFIKLFEEHKELLNLF----EKFI-----AL-Q----TREEQA---SSEELQEHAT--KVMNTLDE-GIRG-L---D---DM--DAFFSYL-GQVGASHRRIN--GFQSQYFWKIEGPFLKAVEQ--TLGDR-YTAN----VE-NIYKVTIKLII------------QTL-------VD-GFEG---T-----GPAAG------------------------------S-----------AGS--------------------------------------------------------------------------------------------------------

>Flower_thrips_HbL

-----------------------------------------------------------------------------------MGAILSY-----------------------------L-----------------------------WSPSL----------------------------------------------------------------------S-TEV-------DPATGLSPRDKHLVRTTWAIV--KKDASS---NGLYLFQLLFTKHTDVRDMF----PFAR-----GK-E----A-AEYR---DDPRMRAHAN--AVMYALTS-YIDQ-L---D---DV--PCLDAMV-RKLADSHLKRH---VTPEHFKALGAVVMQALQD--LLGASVMTPD----AV-TAWTRTYGLVL------------QVV-------TD-QMGK---A-----QS--------------------------------------------------------------------------------------------------------------------------------------------------------

>Gar_GbX1

-----------------------------------------------------------------------------------MGCALSG-----------------------------SGG---------------------------GARGP---------------------------------------------------------------------GF-RLK-------SEPVPLTESQKDLIRESWKVV--HQDIAR---LGIIMFIRLFETHPECKDVF----FIFR-----EIDD----L-QELK---MSKELQAHGL--RVMSFIEK-SVAR-L---A---QE--DKLEQIA-LELGKCHCRYN---APPKYYEYVGVQFISAVKP--ILKDS-WSPQ----VE-QAWESLFAYLA------------AVM-------KR-GYHE---E-----EHKDG------------------------------V-----------NKAS---------------------------------------------------YARKRPPQSPAEEAP-PNCI--------------------------------

>Gar_GbX2

-----------------------------------------------------------------------------------MGCAISG-----------------------------LGLA----------------------PKQ--------------------------------------------------------------------------IDA-TEE-------EALPHLSDHHIEQIKDSWKVI--QEDIAK---VGIIMFVRLFETHPECKDVF----FLFR-----DVED----L-ERLR---ASKELRAHGLNFRVMSFIEK-TVAR-L---D---QL--DRLDQLA-LELGKSHYRYN---APPKYYGYVGTEFICAVQP--ILKEK-WTSE----VE-EAWQTLFLYVT------------RIM-------KR-GYQE---E-----EKSKR------------------------------N-----------NVVI---------------------------------------------------ASKERPEKKGTAI---------------------------------------

>Tsetse_fly_Glob1A

-------------------------------------------------------------------------------------------------------------------------------------------------------------------------------------------------------------------------------------------------MNSDEVYEIKRTWEIP--ATTPTE---SGVAILIRFFTKYPSNLQKF----STFK-----DM-P----L-DELK---NNPRFKAHAN--RIMKVFDD-SIKT-LDDNC-------SHLEEIW-TKIAQSHFNRQ---IEKQSFNELKEVILEVLVA--ACN---LNDQ----QT-EIWLKLLDFVY------------EII-------FK-TIDQ---L-----EQDV------------------------------------------------------------------------------------------------------------------------------------------------------

>Tsetse_fly_Glob1B

-------------------------------------------------------------------------------------------------------------------------------------------------------------------------------------------------------------------------------------------------MNSDEVSEIKKTWEIP--AASPTE---SGVAILIQFFTKYPSNLEKF----STFK-----DM-P----L-DELK---ESPRFKAHAN--RVIKVFDD-SVQA-LDDDP-------SQLEEIW-VKVTQSHFNRQ---IEKHSFNELKEVILEVLTA--ACS---LNDQ----QI-EIWIKLMDFIY------------DII-------FR-TIDE---L-----EQAA------------------------------------------------------------------------------------------------------------------------------------------------------

>Tsetse_fly_Glob2

M----------------------------------------------------------------------------------IDSGI--------------------------------------------------------------------------------------------------------------------------------------QYPKPLPTLNLENVCNEMGFTPLEIVALQNIWRLF--KKRFKY---HSMQIFLAFFNQNHKLIERF----RLPS----GNF---------------QLSHLCQHSE--KMLLLYEN-VIDKCL---D---NM--ANFHGVM-AEVTVKHQRCG---VKYEEIILKTEHVRRYILE--YFANQ-SSPT----LV-SALAKLSEHFNDRHRPKEGE---SDV-------SE----------------EGD-----------------------------------------------------------------------------------------------------------------------------------------------------

>Chicken_GbE

-----------------------------------------------------------------------------------------------------------------------------------------------------------------------------------------------------------------------------------------------MSFSEAEVQSARGAWEKM--YVDAED---NGTAVLVRMFTEHPDTKSYF----THFK-----GM-D----SAEEMK---QSDQVRGHGK--RVFTAIND-MVQH-L---D---NT--EAFLGIL-NPLGQKHATQL--KIDPKNFRIICDIILQLMEE--KFGG-----D----CK-ASFEKVTNEIC------------THL-------TN-IYKE---AG---W----------------------------------------------------------------------------------------------------------------------------------------------------------

>Bot_fly_Glob1

-------------------------------------------------------------------------------------------------------------------------------------------------------------------------------------------------------------------------------------------------MNSEEVNDIKRTWEVV--AAKMTE---AGVEMLKRYFKKYPHNLNHF----PWFK-----EI-P----F-DDLP---ENARFKTHGT--RILRQVDE-GVKA-LSVDF---GD--KKFDDVW-KKLAQTHHEKK---VERRSYNELKDIIIEVVCS--CVK---LNEK----QV-HAYHKFFDRAY------------DIA-------FA-EMAK---M-----G---------------------------------------------------------------------------------------------------------------------------------------------------------

>Scud_GbXb

MKKKTS------ID-------VDWSK--FKFAS-------------------------------------------------SDGNSVSR-----------------------------KPSQ---------------------DCDDTSAAPSWHRPSK----------------------GSFD-----LGRVALRFTKRSSTDKGI---------DGP--AL-EEE-------PPPAELTQEQKIIIKETWAIV--KQNVER---VGVIMFTNLFETHPDVQEVF----LPLR-----GM-E----K-NALL---DNKKLRNHAL--RVMGFVEK-AVGR-L---E---EP--AQLQALL-ETCGRNHCGYG---AALHHIDLVGPQLLEAIKP--SLEDR-WSPE----IS-TAWTLLMDNIA------------YAM-------KA-AMRL---Q-----MRQA------------------------------------------------------------------------------------------------------------------------------------------------------

>Scud_GbXLa

-----------------------------------------------------------------------------------MGCQLTK-----------------------------ALTEGDGKSPTSP--------NAPNGPKNNKKKKKKDKD----------------------------------------------------IDANGKLEEPP--PP-PAL-------DPRLPLTARQKFSILKSWKGI--SRALEP---TGVTMFVKLFERNAELLSLF----VKFR-----EL-K----TPDEQA---ESLELAEHAT--VVMSSIDE-GIRA-M---D---NV--DFFFDLL-HQIGGSHVKIP--GFKKEYFWKIERPFLEAVRL--TLGDR-YTDN----MD-QIYQLTIKFVL------------ETV-------VK-GYEM---A-----VEREV------------------------------N-----------ENVQQLDLSSEPAMNNGS-----------DVPAQTTS--AQN-----GANS---AESSAVRAQGDAA----------PATA----GGGKCPAVCPYVSSGSLSPTTS

>Postman_butterfly_GbXLb

-----------------------------------------------------------------------------------MGCKLSQ-----------------------------LAASEF---------------------------------------------------------------------------------------SHDPFDRPP-----PAS-------DPRSPLTAKQQYCIMASWKGI--FRQIEK---TGIILFIKLFEENEELMHLF----EKFR-----EL-K----TKEAIV---SSAELVEHAT--KVMHTLDE-GIKG-L---A---DM--DSFFAYV-RHVGGTHRQVP--GFKAENFLKIEQPFLEAAKT--TLGDR-YTPN----IE-NIYKLTIRFIL------------ENL-------VK-GYED---A-----GQENG------------------------------T-----------TET--------------------------------------------------------------------------------------------------------

>Postman_butterfly_GbXLa

-----------------------------------------------------------------------------------MGCQLGK-----------------------------LAASER------------------------RVNNQG--------------------------------------------------------------LGDG--PP-PAT-------DPRLPLTAKQKYSMLASWKGI--SRAMEK---TGICMFIKLFEENQDLLNMF----EKFR-----QY-R----TKEEQI---NSMELAEHAN--NVMNTLDE-GIKG-L---D---DL--DNFFEYI-HQ-------------------KIETPFLAAVEA--TLGDR-YTPN----VE-NIYKITIKFIL------------QTL-------VD-GYEK---A-----AKTNS------------------------------A----------------------------------------------------------------------------------------------------------------------

>Postman_butterfly_HbL2

MLYFYLCSTFLLLSAFI----------VLKRVKI--------------------------------------------GQKVTMGSWLSY-----------------------------L-----------------------------WWGGD----------------------------------------------------------------------P-DAV-------NPKTGLSRREVYAVQQSWAPV--YASSVT---NGTELLRRLFQAYPETKEFF----KMIR-----KC-S----E-EEYS---QNPQFKAHVI--NLMGSIDL-AVTN-L---N---EP--DVVAAMM-NKLGESHGRRK---IQREHFYGLKDVIVKMFIE--VLK---LDGA----TL-TAWDKTVDFWY------------KHI-------FE-TLCL---G-----DAR-------------------------------------------------------------------------------------------------------------------------------------------------------

>Postman_butterfly_HbL1

-----------------------------------------------------------------------------------MGGWMTH-----------------------------L-----------------------------IWGGD----------------------------------------------------------------------P-DAV-------NPVSGLSKRDIYVVQKTWAVA--ATDSVG---TGNELLKRYFRAYPETKDFF----RMIK-----NV-P----E-EKFT---ENFQFKAHVI--NLMSALDL-AVKN-L---H---QP--EVVAAMM-AKLGESHGRRK---IQEKQFNELTIVIVQLFKD--VLN---LDDK----TL-AAWGRVVGFWY------------KHI-------FE-TLSG---G-----ENR-------------------------------------------------------------------------------------------------------------------------------------------------------

>Brown_marmorated_stink_bug_GbX

-----------------------------------------------------------------------------------MGNVAAG-----------------------------RRGSVL--------------------SKESSITDSK--------------------------------------------PSRQNT---LAED------------------------SPPEPLSDLQKAELTRTWKLL--EEDIAK---VGVITFISLFETHPDVQQVF----MPFS-----GI-E----L-EDLK---HSKQLRAHAL--RVMAFVQK-CISR-L---N---EP--EKLEQLL-RELGKKHHSYK---AKAKYVDLVGPQFIQAIQP--SLGEE-WNEE----VS-EAWILLFAHIN------------YTM-------KG-AMNE---A-----AEEAK------------------------------M-----------KQ---------------------------------------------------------------------------------------------------------

>Brown_marmorated_stink_bug_GbXL

-----------------------------------------------------------------------------------MGCELGK-----------------------------LTKS---------------------------------------------------------------------------------------------GAEQA--SP-PKQ-------DPRLPLTAKQKYSMLASWKGI--SRAMEQ---TGVYMFIKLFEEHEELLELF----AKLK-----EL-R----TKEEQA---SSLELQEHAT--KVMNTLDE-GIKE-L---D---DL--DTFFTFL-TQIGQSHKKIP--GFKPDYFWKIEKPFLEAVEM--TLGDR-YTEN----VE-NIYKVTIKLII------------DTL-------VN-GYNT-------------------------------------------------------------------------------------------------------------------------------------------------------------------

>Human_Cygb

-----------------------------------------------------------------------------------MEKVPGE------------------------------------------------------------------------------------------------------------------------------------MEI-ERR-------ERSEELSEAERKAVQAMWARL--YANCED---VGVAILVRFFVNFPSAKQYF----SQFK-----HM-E----DPLEME---RSPQLRKHAC--RVMGALNT-VVEN-L---H---DP--DKVSSVL-ALVGKAHALKH--KVEPVYFKILSGVILEVVAE--EFASD-FPPE----TQ-RAWAKLRGLIY------------SHV-------TA-AYKE---VG---WVQQVP------------------------------N-----------ATTPPATLPSSGP----------------------------------------------------------------------------------------------

>Human_HbA

----------------------------------------------------------------------------------------------------------------------------------------------------------------------------------------------------------------------------------------------MV-LSPADKTNVKAAWGKV--GAHAGE---YGAEALERMFLSFPTTKTYF----PHF----------------DLSH---GSAQVKGHGK--KVADALTN-AVAH-V---D---DM--P---NAL-SALSDLHAHKL--RVDPVNFKLLSHCLLVTLAA--HLPAE-FTPA----VH-ASLDKFLASVS------------TVL-------TS-KYR--------------------------------------------------------------------------------------------------------------------------------------------------------------------

>Human_HbB

----------------------------------------------------------------------------------------------------------------------------------------------------------------------------------------------------------------------------------------------MVHLTPEEKSAVTALWGKV----NVDE---VGGEALGRLLVVYPWTQRFF----ESFG-----DL-S----TPDAVM---GNPKVKAHGK--KVLGAFSD-GLAH-L---D---NL--K---GTF-ATLSELHCDKL--HVDPENFRLLGNVLVCVLAH--HFGKE-FTPP----VQ-AAYQKVVAGVA------------NAL-------AH-KYH--------------------------------------------------------------------------------------------------------------------------------------------------------------------

>Human_Mb

-----------------------------------------------------------------------------------------------------------------------------------------------------------------------------------------------------------------------------------------------MGLSDGEWQLVLNVWGKV--EADIPG---HGQEVLIRLFKGHPETLEKF----DKFK-----HL-K----SEDEMK---ASEDLKKHGA--TVLTALGG-ILKK-K---G---HH--E---AEI-KPLAQSHATKH--KIPVKYLEFISECIIQVLQS--KHPGD-FGAD----AQ-GAMNKALELFR------------KDM-------AS-NYKE---LG---FQG--------------------------------------------------------------------------------------------------------------------------------------------------------

>Human_Ngb

-------------------------------------------------------------------------------------------------------------------------------------------------------------------------------------------------------------------------------------------------MERPEPELIRQSWRAV--SRSPLE---HGTVLFARLFALEPDLLPLFQYNCRQFS-------------SPEDCL---SSPEFLDHIR--KVMLVIDA-AVTN-V---E---DL--SSLEEYL-ASLGRKHRAVG---VKLSSFSTVGESLLYMLEK--CLGPA-FTPA----TR-AAWSQLYGAVV------------QAM-------SR-GWDG---E---------------------------------------------------------------------------------------------------------------------------------------------------------------

>Tobacco_budworm_HbL

-----------------------------------------------------------------------------------MGGWLSY-----------------------------F-----------------------------WWGGD----------------------------------------------------------------------P-DVV-------NPVSGLTRREISLVQKSWVPV--NADAIN---TGAELLKRFFIAFPESKDFF----KMLK-----NT-P----E-DQYL---QNPQFKAHVI--NLMTSLNL-AVEN-L---N---QP--EVVAAMM-NKLGESHGRRK---IQEKNFNQLKEVIVKMFIE--VLK---LDAV----TL-GAWGKTVEFWY------------KHI-------FE-TLNR---A-----EQTR------------------------------------------------------------------------------------------------------------------------------------------------------

>Glassy-winged_sharpshooter_GbXL

-----------------------------------------------------------------------------------MGCELGK-----------------------------LAQMQR------------------------GGSEGG---------------------------------------------------------PNRGADDPP--AP-APP-------DPRLPLTAKQKYSMLASWKGI--SRAMEP---TGVYMFIKLFEEHEELLDLF----TRFR-----EL-K----TRDAQA---NSMELQEHAT--KVMSTLDE-GIKE-L---D---DL--DSFFEYL-HQIGASHRKIP--GFKPDYFWKIEKPFLEAVKM--TLGDR-YTDN----VE-NIYKITIKLII------------ETL-------EK-GYKG---S---------------------------------------------------------------------------------------------------------------------------------------------------------------

>Glassy-winged_sharpshooter_HbL2

MAKLK------------------------------------------------------------------------------LNCGQS---------------------------------------------------------------------------------------------------------------------------------------------------LPLEAITDRDKELAREAWVQV--EFNYVL---ISKNLFVDWFTQYPEHVNFF----KHMM-----DS-S----C-DDIF---TSPKFARHMAN-SLLPNLGI-IIRN-L---D---RP--NDFRSHI-LKVAWSHVERN-LDLNSDHLDILKGLILRTLKD--SLGGG-IGLD----HE-VALFKIITAAFKI--------FGEVL-------EN-RVNE-------------------------------------------------------------------------------------------------------------------------------------------------------------------

>Glassy-winged_sharpshooter_HbL3

-----------------------------------------------------------------------------------MASGRRTSDNLR----------------------------------------------NSRPDGRRQPGAQGVPPLR--------------------------------------------------------------RSAHERP-------KDLASLTDRDLRLGRATWFKN--VDATPD---FGMVIFKELFRQYPEVESYF----LHLR-----GN-S------GSIF---DSRTFRSHMTE-RVVPKLKE-VFEA-L---D---KP--EHLNEVM-TKLGLYHAKLG---VSGHLVENMLSVILDALKS--VMPTK-MQPD----EE-TAVRTCLKSAFAI--------AIDTINIYEKENKQAATDS-------------------------------------------------------------------------------------------------------------------------------------------------------------------

>Black-legged_tick_GbXL

-----------------------------------------------------------------------------------MGCTLSK-----------------------------TLSSLV-------------------RGSEGRKGSNSGTG----------------------------------------------------GSEADPVADPP--PP-PPP-------DPRQPLTARQIFSISKSWKAI--ARAMEP---TGIEMFVRLFQEKEDLLDLF----EKFQ-----AL-R----TKESQR---ESMELAQHAS--VVMTTLDE-GINA-L---D---NL--DYFMSYL-HNAGRLHYKIK--GFKKEYFWHIEGPFLAAVSD--TLGDR-YTDN----IE-NIYKITIRFIL------------QTL-------IE-GFEE---A-----EKEAA------------------------------Q-----------QAR--------------------------------------------------------------------------------------------------------

>Black-legged_tick_HbL1

-----------------------------------------------------------------------------------MGN---------------------------------------------------------------ELGAN----------------------------------------------------------------------T-QHR-------DTITEMTSQEKHVVRDTWAIF--KKEVQT---SGVAIFVVLFFKHPAYQKLF----VAFA-----AD-P----I-AELP---QNPRAIAHAL--TVAYAITS-IIDT-L---D---EP--ETSAELV-RKVATNHVRHP--TISGAQFEHMGQAVVEVLAE--KLGSA-MNHQ----AV-GSWQKFFAFVV------------RVS-------QG-VFKK---RP---FRKARS------------------------------E-----------DT---------------------------------------------------------------------------------------------------------

>Black-legged_tick_HbL2

---------------------------------------------------------------------------------------MSW-----------------------------L-----------------------------FGSAS----------------------------------------------------------------------A-DMP-------STKTGLTTSDKCAIKDTWTMF--RRETRT---NALSLFVALFSRYPEYQKMF----PNFA-----DV-A----L-KDMM---QCPSLTAHAL--TVIYALAS-IIES-I---D---DE--NTMVELI-KKNIRNHVRRS---VTPEHFVNINNLLIEVMQV--KLRSR-MTAS----VI-VSWKKFFAMHD------------AVT-------RQ-TYDE---F-----RAQSA------------------------------V-----------E-----------------------------------------------------RAAGTSNSGV-------------------------------------------

>Black-legged_tick_HbL3

-----------------------------------------------------------------------------------MGS---------------------------------------------------------------IVSSQ----------------------------------------------------------------------Q-DVP-------DAKTGLTPREKGLVRDTWALV--RKDVKA---NAIAIFLTLFQRHPEYQKLF----SGFA-----DV-P----P-EALS---TNPRLGAHAM--SVAYAITS-LVDS-L---D---DA--ECLVELV-RKVAVSHTRRP---VSVTHFENLTVVIVDTLKE--RLGGK-MSPA----AV-AAWEKTLRLVV------------TVT-------AD-VYKE---Q-----RK--------------------------------------------------------------------------------------------------------------------------------------------------------

>Black-legged_tick_HbL5

-----------------------------------------------------------------------------------MGYGVSR------------------------------------------------------------------------------------------------------------------------QSV---------LRGEVDP-------DLSNSLTARQAELVRSTWAIV--SQDLAG---TGVVVFKRLLTRYPELCRLF----RKFM-----TL-R----D-DGTYD-WDMEGLQRHAL--LVMQGLEA-AVEN-L---D---DS--RVLADIL-YELGRKHARFN---VHEDMFDKLWHALKFGLED--ALQDR-FNRE----VA-QAWFIIFRFLS------------RKI-------IE-GMLE---H-----RAKMAE-----------------------------------------EKAKQEIGDKPDKITDKG------------------------------------K----------------------PR----------------------------

>Black-legged_tick_HLb4

-----------------------------------------------------------------------------------MGNILT---------------------------------------------------------------------------------------------------------------------------------------K-SLP-------DSRTGLSKRDTKLIRNSWSML--CKQHPK---ADQLIFKALFTKHPDFMALF----QHFK-----DK-D----L-GVVL---SDPQFALHSS--AIIQAFGT-IIRS-L---D---DP--AGVVALI-RKNATDHTTRK--GVQPSHFEAMLNVVLEVLQD--KLGSR-FKPE----AI-TAWEKFIEVGK------------LLW-------SE-EKKR---------------------------------------------------------------------------------------------------------------------------------------------------FVVFW-----------

>Australian_sheep_blowfly_Glob1

-------------------------------------------------------------------------------------------------------------------------------------------------------------------------------------------------------------------------------------------------MNCDEVYEIKKTWEIP--AATPTE---SGVAILLKFFTKYPSNLEKF----YSFK-----DL-P----I-DELK---NNARFKAHAV--RIIKVFDE-SIQM-LGHDW---SG--PKLEEMW-SKVAVSHFNRQ---IEKQSFNELKEVILEVLTA--VCN---LNEK----QT-AAWIKLMDIVY------------SIV-------FN-TLDK---L-----KNGEQ-----------------------------------------------------------------------------------------------------------------------------------------------------

>Australian_sheep_blowfly_Glob2

-----------------------------------------------------------------------------------MATNI--------------------------------------------------------------------------------------------------------------------------------------IYPKPLPKLVKQNIPLETGFTSTEIVALQNGWHLI--KRRLYY---HSTKIFKDFFSEHYLLLERF----RNVE---IGKF---------------NLSNLHQHPG--QLMNIYGR-LIESGL---N---DV--AFINMLL-SDVGQRHKLYE---VTYDDVKLLTNHIRLYVIE--FLDKI-KSIT----FV-NGLTKLSELINEHHREKAEDA--EQT-----ARTQ-SDDE-------------D-----------------------------------------------------------------------------------------------------------------------------------------------------

>Scarce_chaser_GbXa

-----------------------------------------------------------------------------------MGNSAHS-----------------------------RM------------------------SKEGSDGSPKSLRQK-----------------------------SLSCAESTEEPKAAND--ATAAAENGPKSKSV--VM-ELN-------VKPEPLTEEQMGLLQENWKEL--EDNIAK---VGVITFISLFETHPDVQEVF----LPFQ-----GM-E----L-EELR---HSKQLKAHAL--RVMAFVQK-AVAR-L---H---EP--DKLDVLL-RELGKKHYTYG---AKQEYIDYIAPQFIQAIKP--SMEDK-WTKE----LE-GAWTALFNYMG------------VIM-------KA-AMDL---E-----ERRAA------------------------------A-----------ESLP----PYNVPLPT--------TQQSCSALPPTQPFAP---------SKGLLHGSRRASSDV-----GGDGVSAA-------------GGTSLLASKRRGT-LY-

>Scarce_chaser_GbXb

-----------------------------------------------------------------------------------MGNSVHS-----------------------------RNAK---------------------DNGQESASKAAPPKPV-------------------------------DDGNDAGFDKNSES---------ALKHRAF--EM-ELD-------VKPEPLTEKQMAMLQENWKEL--EDNIAK---VGVITFISLFETHPDVQDVF----IPFK-----GM-G----L-DELR---HSKQLKAHAL--RVMAFVQK-AVAR-L---Y---EP--EKLDLLL-RELGKKHYSYG---AKQQYIDYIAPQFIQAIKP--SLEDR-WTGE----LQ-DAWIALFNYMG------------VIM-------KA-AMDW---E-----EKRAA------------------------------A-----------ATIPYALPPSSHPSKTGAPAPVNPTAPPVTPAPPTNPTPPINPPPPVAAATNITKEPNRRASDVITNTRKGTGVRT--------------------EALRRGT-LN-

>Scarce_chaser_GbXc

-----------------------------------------------------------------------------------MGNSAHS-----------------------------RL------------------------AKEGSADCSPRSFRH----------------------KSLSRR-----ESSSEQNKRSKDASGATPAENGLEVK-V--IE-RDP-------VTAKKLTERQKDLLTETWKEL--EENIAR---VGVIMYIGLFETHPDVQEAF----MPFK-----GI-E----L-TELK---HSKQLRSHAL--RVMGFVQK-AVAR-L---H---EP--EKLDVLL-RDLGKKHFSYA---AKPEYVDLIGPQFIQAIKP--SLEDK-WNEE----IH-EAWTTLFEYMA------------SMM-------KT-EMAY---E-----EKKAI------------------------------A-----------NSAT----ETRVQNTN--------NRPTASSKTSTSSHNN---------PKN--HGSRRASSDAAT---IGGTVAA--------------------VNNRRGT-LH-

>Scarce_chaser_GbXL

-----------------------------------------------------------------------------------MGCELGK-----------------------------LGLVQR------------------------ERKSGD------------------------------------------------------------EVPEPP--AP-APT-------DPRLPLSAKQKYNIMASWKGI--SRAMEP---TGVNMFVKLFEEHQELLSLF----EKFK-----EL-R----TKEEQR---NSMELAEHAT--QVMRTLDE-GIKG-L---D---NV--EFFLEFV-RQVGGTHHRIP--GFHKDYFWRIETPFLDSVKT--TLGDR-YSDN----MD-TIYKVTIKFII------------CTL-------VE-GFER---E-----EARAK------------------------------E-----------RDAQEKKEA--------------------------------------------------------------------------------------------------

>Scarce_chaser_HbL2

-----------------------------------------------------------------------------------MGLR--------------------------------------------------------------HSTAV----------------------------------------------------------------------D-NTP-------DPVTGLTPAQIQAVRSTFDVL--RSDPKD---FGVDLFLSLFDAHPNYQKLF----RVFG-----NV-P----R-SELP---GNKRFLAHAS--TVVYSLMS-VIDN-L---N---DS--ECLVEML-VRIGQNHGRMN---VQPESFEHLKSVIMGLLKR--RLKDR-FTPF----AE-ESWSKTLDVAN------------SVI-------FK-GLED---G-----QKEKI------------------------------V-----------L-----------------------------------------------------KSENAS-----------------------------------------------

>Scarce_chaser_HbL3

-----------------------------------------------------------------------------------MGGILSL-----------------------------LYRSGDG--------------------PEEMDGPM----------------------------------------------------------------------V-DVP-------DEATGLTPRQKRAVAVTWDIV--KKDLKG---NGVELLHRFFTKHPQYQKNF----KAFA-----DV-P----L-DELP---NSKKFQAHAN--SVVYAVTS-IVDN-L---D---DP--GCLVEML-RKLGQNHGQRH---IPEQAFLDLKAVLMKMLKE--KLEGH-FTAY----EE-ESWDKTMDTAF------------SVI-------FQ-GLKD---Q-----ELTAT-----------------------------------------------------------------------------------------------------------------------------------------------------

>Cricket_HbL

-----------------------------------------------------------------------------------MGSVLSY-----------------------------LWSGS-------------------------AANAA--------------------------------------------------------------------IEC-DVP-------DPDTGLTPRQKKFVSDTWQLV--KRDIKG---NGIELFIRFFEMRPEGQNRF----SSFV-----GM-P----L-NELR---HSKRLQAHTN--SVMYALDG-VVMP-L---D---DP--EVMHEML-LKIGMNHGRRG---ITEEEFHELKIVLMNLLKE--KLDIH-VNSD----GE-EAWSKTIDVFY------------KSM-------FK-GMDM---T-----SH--------------------------------------------------------------------------------------------------------------------------------------------------------

>Lutzomyia_sandfly_Glob1

----------------------------------------------------------------------------------------------------------------------------------------------------------------------------------------------------------------------------------------------MPGLTPEQIEIVKSTWQLV--AKAPED---AGEAILMRFFEKFPDNQKYF-----PFR-----NV-P----R-ENLK---GSVMFRSHAG--RVVAVFQK-AVDA-FNTAD---PV--ATLVEIW-TEIARTHFRRQ---IKQKSFDELKEVVLEILTA--ACN---LDEV----QQ-TAWAVTLDTIF------------GII-------SK-ELAN---L-----AESSQ-----------------------------------------------------------------------------------------------------------------------------------------------------

>Yellow_lupin_LegHb

-----------------------------------------------------------------------------------MG-----------------------------------------------------------------------------------------------------------------------------------------------------------ALTESQAALVKSSWEEF--NANIPK---HTHRFFILVLEIAPAAKDLF----SFLK-----GT-S------EVPQ---NNPELQAHAG--KVFKLVYE-AAIQ-L---Q---VTGVVVTDATL-KNLGSVHVSKG---VADAHFPVVKEAILKTIKE--VVGAK-WSEE----LN-SAWTIAYDELA------------IVI-------KK-EMND---AA--------------------------------------------------------------------------------------------------------------------------------------------------------------

>Migratory_locust_HbL1

-----------------------------------------------------------------------------------MGALLSF-----------------------------LWGGGG------------------------ATPAL----------------------------------------------------------------------P-EVA-------DPVTGLTPREKHFVVTTWAAV--RKDITS---NGVQLFLRFFDKLPAAQKRF----SSFA-----DL-P----R-DELA---ASKRLKAHAN--SVMYSIDS-IVCN-L---D---DP--EVLEEML-LKIGNNHGRRK---IPEDEFMVLKDVLMQLLRD--ILEIH-KSPV----GE-QAWSKAIDVMY------------KNI-------FK-GMEE---T-----RNK-------------------------------------------------------------------------------------------------------------------------------------------------------

>Salmon_louse_HbL

-----------------------------------------------------------------------------------M--------------------------------------------------------------------------------------------------------------------------------------------------------VTMSLFCEHEVKLISESWKLM--ALDLDN---HGLNFFLKLFKEYPVYEEKF------FP-----DI-N---------G---DRKKLKRHGG--IVMKALGK-LVGF-L---ET-GKI--IAIVNTI-KGIANSHSKRG---VLVQQFTPICDILLKYLGE--AFGDQ-LSNE----GT-ATWKKFLDIFV------------SVI-------NE-AYDE---I-----KNKK------------------------------------------------------------------------------------------------------------------------------------------------------

>Jumping_bristletail_HbL

-----------------------------------------------------------------------------------MGGVVSY-----------------------------FFAP--------------------------GNDPN----------------------------------------------------------------------D-DIP-------ESITGLTPREKRAVTESWAII--RTDLKG---NGMIFLLMFFDDYPDYQKFF----RSFA-----EV-P----R-SQLP---DDKRLMAHVT--SVMYALSN-IVDN-L---D---DP--ECLVETL-KKLGENHGRRN---ITLQEFENLKAVVIKFLKH--CVGSK-FSST----AE-AAWVKTLDSAV------------SVI-------NK-GIP--------------------------------------------------------------------------------------------------------------------------------------------------------------------

>Hessian_fly_GbXL

-----------------------------------------------------------------------------------MGCELGK-----------------------------LAASTK---------------------------------------------------------------------------------------SDGAYGQPD--EP-PPH-------DPRLPLTARQKYTVIASWKGI--ARALQP---TGINMFIELFEEHGELLGLF----NKFR-----EL-K----TKEEQA---SSEELAEHAN--KVMETLDE-GIRA-L---E---DL--NTFFPFL-HQVGGSHTRIP--GFQAEYFWKIEQPFLSAVKT--TLGDR-YTEN----VS-GIYIITIKFII------------ETL-------IT-GFES---A-----NSTQS------------------------------N-----------SNSDVNKINSKTD----------------AMPAKS------------------------------------------------------------------------

>Hessian_fly_Glob1

----------------------------------------------------------------------------------------------------------------------------------------------------------------------------------------------------------------------------------------------MASLTPHQIALIQSTWSIP--AKVPID---SGEAILLAYFEKYPQNQQKF----NAFK-----NT-P----L-LSLK---GTPGFRTHAG--RIITVLDE-AISN-LSKEN---YV--EELERIW-NQIGESHNRRK---ISRQSFNELRDILVHTLIQ--VCS---LDDE----GK-LAWNTLMDIIY------------HIV-------FL-KLDE---N-----NQY-------------------------------------------------------------------------------------------------------------------------------------------------------

>House_fly_Glob1

-------------------------------------------------------------------------------------------------------------------------------------------------------------------------------------------------------------------------------------------------MNTDEVLEIKRTWDIP--AANPTE---SGSAILMLFFKRYPSNLQKF----SAFK-----DL-P----L-DELS---TNARFRAHAS--RIIKVFDE-SIQM-LGHDW---AG--PKLEETW-SKIATSHFNRQ---IEKKSFNELKEVILEVLTA--ACN---LNEK----QI-QAWTKLMDTVY------------SII-------FN-SLDK---L-----EKGEQ-----------------------------------------------------------------------------------------------------------------------------------------------------

>House_fly_Glob2

-----------------------------------------------------------------------------------MSFHI--------------------------------------------------------------------------------------------------------------------------------------LYPKPLPPRDNQSLPNENGFTATEIASLRNGWRHF--KRRFGY---HSKQIFMKFYQEHEQMLEKF----RNRM----GKF---------------NMQQLHRHPQ--ELLQVYGN-LIEQGL---D---NM--TYMHVLM-TAISQRHRMFG---VTGYEIKLQTDHITLYILA--LLEKI-ISPT----FV-SGLEKLSRLINAYHCEDACD---ELL-------EE-SSNE---A----LHN--------------------------------------------------------------------------------------------------------------------------------------------------------

>Turkey_GbE

-----------------------------------------------------------------------------------------------------------------------------------------------------------------------------------------------------------------------------------------------MSFSEAEVQSARGAWEKI--YVDAED---NGTAVLIRMFTEHPDTKSYF----THFK-----GM-D----SAEEMK---QSDQVRGHGK--RVFTAIND-MVQH-L---D---NT--EAFLGIL-NPLGQKHATQL--KIDPKNFRIICDIILQLMEE--KFGG-----D----CK-TSFEKVTNEIC------------THL-------TN-IYKE---AG--------------------------------------------------------------------------------------------------------------------------------------------------------------

>Whipscorpion_HbL

-----------------------------------------------------------------------------------MSAGVK---------------------------------------------------------------------------------------------------------------------------------------N-GAQ-------EILMGITLREKTLIRESWDLI--RPDLKG---NGIAFFIKLFDEFPEYQKLF----KSFD-----KV-P----K-EELP---TNKRMIAHAT--TVMYGFAS-FVDS-L---D---DP--DLLEGLI-EKIVTNHARRQ---ITKENFKNIGIVLENYLKD--RLGIK-LTEE----GI-QAWRKLCDVVQ------------AVA-------EE-ILVK-------------------------------------------------------------------------------------------------------------------------------------------------------------------

>Hibiscus_mealybug_HbL

-----------------------------------------------------------------------------------MT-------------------------------------------------------------------------------------------------------------------------------------------------------DSKKIFADEVIKDVKTTWATI--NSDLQQ---VGYEIFNRLFNAFPTYQQLF----RAFK-----DV-P----F-GELQ---SNKDYSKHAL--AVAKALNA-SIEN-L---E---NP--EQLVSIL-TTVGKNHVKRN---VTPEHYSNAQKIILEVIGT--KLGDE-NSDK----IL-SSWNEVLAVAV------------STI-------MK-GAQE---E-----EAKYI-----------------------------------------------------------------------------------------------------------------------------------------------------

>Twisted-wing_parasite_GbXL

-----------------------------------------------------------------------------------MGCKMSK-----------------------------SGSTST------------------------ASGSKSNNN-----------------------------------------------------RNGKVLEEPP--PP-QPP-------DPRLPLTAKQKYNMLASWKGI--SRAMEP---TGVLMFIKLFEEHQELLNMF----DKFK-----QL-K----TKEEQA---SSMELVEHAT--KVMRTLDD-SIKS-L---D---NL--DAFFDYV-HQVGFSHQRIP--GYKSDFFLKIEKPFLEAART--TLGDR-YTEN----IE-NIYQITIKFIL------------QTL-------IE-GFEQ---S-----PNAVQ------------------------------D-----------ASSSSQAPS--------------------------------------------------------------------------------------------------

>Western_predatory_mite_GbXL

-----------------------------------------------------------------------------------MGCTLSK-----------------------------AVTSLV------------------------HKGSERSAG----------------------------------------------------VGRVGDVEDPP--PP-PPP-------DPRSPLTTRQIFSISKSWKAI--ARAMEP---TGVEMFVRLFKQNEELLDLF----TSFQ-----AL-K----TEESQR---ESMELGQHAS--LVMTTLDE-GINS-L---D---NL--DYFLEYL-HNAGGMHYKIK--GFKKEYFWLIEKPFLEAVKL--TLGDR-YTDN----IE-NIYNTTIHFIL------------ETL-------VE-GFAL---A-----ETKAK------------------------------C-----------SE---------------------------------------------------------------------------------------------------------

>Western_predatory_mite_HbL1

-----------------------------------------------------------------------------------MG-----------------------------------------------------------------------------------------------------------------------------------------------------NIASPHKLSTDEVDAVQAAWQVV--RQDQRS---IGQQVMMTLFSENPEYIHKF----KHLQ-----MI-A----A-DQLP---YHTALRAHSL--SILYVIHS-LIDS-M---D---DE--ETMRELI-RKVALTHKPRS---VNRDNFQRFEDAFILVLKK--YGIDR--------RTE-EAFHKCILYFT------------DIY-------EK-AEDT-------------------------------------------------------------------------------------------------------------------------------------------------------------------

>Peach_aphid_HbL

---------------------------------------------------------------------------------------------------------------------------------------------------------------------------------------------------------------------------------------------MASSLSPLQISQLKDSWSVL--AQDPSQ---LASALVIRLFKENPEYQSLF----KRLK-----NL-S----I-DELA---SNPQFMSHAS--KVGAALGL-TIDH-L---D---KP--EELEKIL-TNLGIKHKKYG---LTAKHFQVIGDVLVAMISE--AIGNS--EPE----LL-DLWKSSLTSVL------------SII-------IA-ACQ--------------------------------------------------------------------------------------------------------------------------------------------------------------------

>Alfalfa_LegHb

-----------------------------------------------------------------------------------------------------------------------------------------------------------------------------------------------------------------------------------------------MSFTDKQEALVNSSWEAF--KQNLPR---YSVFFYTVVLEKAPAAKGLF----SFLK-----NS--------AEVQ---DSPQLQAHAE--KVFGLVRD-SAVQ-L---R---AT--GGVVLGD-ATLGAIHVRKG---VVDPHFVVVKEALLKTIKE--AAGDK-WSEE----LN-TAWEVAYDALA------------TAI-------KK-AMS--------------------------------------------------------------------------------------------------------------------------------------------------------------------

>Humpbacked_fly_Glob1a

-----------------------------------------------------------------------------------------------------------------------------------------------------------------------------------------------------------------------------------------------MELSDFEVIEIKNTWKIP--MADPSG---SGQAILLKFFERYPHNKLKF----QDFK-----DQ-S----L-DQLK---TCPKFKAHAS--RIVRTFNE-AINV-LGTDY---TD--PALHEIF-SKVAISHHKRG---ISKASYNELKEVILEIVVA--VCE---MNDC----QK-CAWEKLMETIY------------ELI-------SR-PLIA---L-----KSEVGFLN------------------ILKFV---CN----------------------------------------------------------------------------------------------------------------------

>Humpbacked_fly_Glob1b

----------------------------------------------------------------------------------------------------------------------------------------------------------------------------------------------------------------------------------------------MIELSDNDILEIKATWKIP--MANPSE---SGEAILLKFFERYPSNLEKF----KDFK-----EM-T----S-DELK---VCPRFKAHAS--KIIRTFDE-AINI-LGTDS---TD--SALQEIW-SKVALSHHKRD---ISKSSYNELKEIIIEILVA--VCK---MNND----QK-KAWEKLLICVY------------NVI-------FQ-TIDN---I-----SN--------------------------------------------------------------------------------------------------------------------------------------------------------

>Humpbacked_fly_Glob1c

---------------------------------------------------------------------------------------------------------------------------------------------------------------------------------------------------------------------------------------------MDLELSEAEILEIQNTWKIP--MADPLA---SGQAVYLKLFKRYPSNQLKF----IDFK-----DV-R----Y-EDLK---DSPRFKFQAL--RLMRTFDK-AINA-LGTEN---AG--NILHEIF-AKVAVSHHKLG---ITKVAHDQLKEVLIEILIE--ICG---MNDF----QK-TAFEKLMEATF------------NVI-------YS-ENWL---L---------------------------------------------------------------------------------------------------------------------------------------------------------------

>Humpbacked_fly_Glob1d

---------------------------------------------------------------------------------------------------------------------------------------------------------------------------------------------------------------------------------------------MDLDISEKEVLEIKRTWKIP--MADPLT---SGETMLLKLFERYPANQKKF----QDLK-----DL-P----F-KDLK---DSPKFKFHSV--RIMKAFDE-AIQS-LGTQN---AG--MVLHEIF-EKVAVSHHKRG---ISKESHNQLKEVIIETLVG--VCD---LNDF----QK-GAWEKVMESIF------------NVI-------YS-ENWI---L---------------------------------------------------------------------------------------------------------------------------------------------------------------

>Brown_planthopper_GbXL

-----------------------------------------------------------------------------------MGCELGK-----------------------------LALAQR------------------------GGSNGGGDSDVG-------------------------------------------------MQEGGGGQRGG--AP-AKP-------DPRLPLTAKQKYSMLASWKGI--SRAMEP---TGVYMFIKLFEEHRELLELF----TKFR-----EL-R----TRDEQA---SSMELAEHAN--KVMTTLDE-GIKE-L---D---NL--DTFFEYL-TNVGASHKKIP--GFKPEYFWRIEKPFLEAVKM--TLGDR-YTEN----VE-NIYQITIKLII------------ETL-------EK-GYNT-------------------------------------------------------------------------------------------------------------------------------------------------------------------

>Brown_planthopper_HbL

MSGLQILV---------------------------------------------------------------------------LRSLFGRISL--------------------------------------------------QNNLPAQTVAQR-------------------------------------------------------------------LFSADCPPKDC---CPPPEPSPKDLANVKEAWCEI--DRNKGC---YAKAIFTEVFKKYPDYAQLF----AKFG-----RC-P------TDIL---KNEKFSEHLKK-NVMDEMGN-VIKK-MG--E---DM--CEAKSMA-SDIGKKHVKLC---VKPKHFENTEKIFIDVLKK--QMGEK-LSCD----GA-KSLENVIKSVF------------KQL-------KK-GAAA---------DSCSS-----------------------------------------------------------------------------------------------------------------------------------------------------

>Nasonia_wasp_GbXL

-----------------------------------------------------------------------------------MGCKLGK-----------------------------LASSTT------------------------SQIQNA----------------------------------------------------------RSGRESPP--PP-PAT-------DPRLPLTARQKYLLTASWKAI--AKAMEP---TGIYMFVKLFEENAELLNMF----SKFK-----NL-T----AQEEQS---KSVELAEHAE--KVMNTLDE-GIQG-L---D---NM--DAFLTFI-HQIGATHTKIP--GFDREYFWKIESPFLAAMQM--TLHDR-YTEN----VE-NIYKLTIKFII------------QTL-------ID-GYDG---A-----KSEKS------------------------------A-----------YYSTPFENKEKS-----------------------------------------------------------------------------------------------

>Nasonia_wasp_HbL

-----------------------------------------------------------------------------------MGSSGSL-----------------------------F-----------------------------WGSAN----------------------------------------------------------------------D-NVL-------NPATGLTGRQKKLVQNMWAIV--RKEPIP---NGVAIMLAYFKKYPEYQKVF----THFK-----DV-P----L-EELS---ANKKFQAHCL--NIVTALNN-LIDS-I---N---DP--ALLEANL-VAIGERHHRRG---QTKEQFLHLKEVIAEVLRQ--KLGAK-FTAE----TA-EAWNKTIDAAY------------TGI-------FQ-TFST-------------------------------------------------------------------------------------------------------------------------------------------------------------------

>Platypus_GbY

----------------------------------------------------------------------------------------------------------------------------------------------------------------------------------------------------------------------------------------------MVQVTDVEKANIQSIWSKM--MENLEK---NGIDIFTRLFREYPETKKYF-------K-----NI-P----LEGNLQ---EDPLLRSHGR--RVMVALNR-IIQN-L---D---NW--KQVCKIL-NPLAEKHKIIH--SVDVENFQFMLKCVGDVCQD--YLGPC-YTPE----IA-ESFQKLQSSLY------------DQV-------VI-TYLH---SG---SD---------------------------------------------------------------------------------------------------------------------------------------------------------

>Milkweed_bug_GbX

-----------------------------------------------------------------------------------MGNVSAV-----------------------------RRGSVL--------------------SKEGSIADSKG-------------------------------------------GSRQNT---VAEE------------------------PTPEPLSDQQRAHLTRTWKLL--EDDIAK---VGVITFISLFETHPDVQQVF----MPFS-----GI-E----L-EDLK---HSKQLRAHAL--RVMAFVQK-CISR-L---H---EP--EKLEQLL-RELGKKHYGYK---AKAKYVDLVGPQFIQAIQP--SLGEE-WTDE----VS-AAWVLLFANIS------------YIM-------KN-AMAE---A-----AEEAA------------------------------R-----------EKQ--------------------------------------------------------------------------------------------------------

>Milkweed_bug_GbXL

-----------------------------------------------------------------------------------MGCELGK-----------------------------LTKSAG--------------------------------------------------------------------------------------SPVEAARPPP--PQ-QQS-------DPRLPLTAKQKYNMLASWKGI--SRAMEQ---TGVYMFIKLFEEHEELLGLF----EKLK-----QL-R----TKEEQA---QSLELQEHAT--KVMHTLDE-GIKA-L---D---QL--DNFFAFL-TGIGQSHKKIP--GFKPEYFWKIEQPFLEAVEK--TLDDR-YTEN----VE-NIYKVTIKLII------------ETL-------VN-GYNS-------------------------------------------------------------------------------------------------------------------------------------------------------------------

>Body_louse_GbX

-----------------------------------------------------------------------------------MGSSVAH-----------------------------HKNHSNSID-----G-------NSTGTEQVQSSSSNQTPIKVKPSNNQKQRRKTFSS--LGIRGSFDFR-----GSSLSFRSRGSFDVGSSKPDQCSP-------V-LEE-------QRPPELTTREKELLIETWKEL--EENIAK---VGVITFVSLFETHPDVQESF----MSFS-----GV-D----I-EDLK---HSKQLRAHAL--RVMAFVQK-AVAR-L---H---EP--EKLETLL-KELGRKHVGYG---AKQKYVELVGPQFILAIKP--SLEKQ-WDEE----LD-DAWTHLFKIIE------------FVM-------VS-SMDD---D-----RKDQR------------------------------T-----------LE--------------------------------------------------------------------------------------------------RYI-IYI

>Body_louse_GbXL

-----------------------------------------------------------------------------------MGCAKST-----------------------------QNGGRG------------------------GGGGSG-----------------------------------------------------------DLKGDEK--PP-PQT-------DPRLPLTAKQKYNLVASWKGI--SRAMEP---TGITMFIKLFEQNEELLNLF----EKFR-----EL-K----TKEQQA---SSMELQEHAM--NVMKTVDE-AIRE-L---D---NL--DGFFIYL-HQVGSSHRKIP--GFKPDYFLKIEQPFLQAVKD--TLGDR-YTEN----VE-RIYNITIKLII------------TTL-------ME-GYTD---Y-----GKS-------------------------------------------------------------------------------------------------------------------------------------------------------

>Body_louse_HbL1

-----------------------------------------------------------------------------------MG-----------------------------------------------------------------------------------------------------------------------------------------LGS-----------SKSEPLTADELERVQNSWKVV--MENAEE---NGMFIFKTFLLKH-NYFPYF----KAFA-----NT-P----L-EELE---ENQAFRNHAN--NIIQALDN-VILN-L---E---DE--LTIQREL-TALGKMHGKKK---ISEQQFQELKICILEILDN--EFK---LPED----DL-QAWSKTLNNAF------------VFV-------FE-GLAA---EV--------------------------------------------------------------------------------------------------------------------------------------------------------------

>Body_louse_HbL2

------------------------------------------------------------------------------------------------------------------------------------------------------------------------------------------------------------------------------------------------------MNVVLNDWPKI--RKNYKK---IFIDSFINYFAENPNYKLLF----PSFS-----NV-S----E-DDLP---FNHCFRLHCF--AVYKAINF-LMSNWL---G---EYE-EDDSKIL-PVIGKTHFDRG---ITLEMMNLYKHSIVYSCNN--HLKPN-LKRK-------LSWQTVFDHIFDYY-------LGSAY----PAPIQ-TVEE---------DD--------------------------------------------------------------------------------------------------------------------------------------------------------

>Body_louse_HbL3

-----------------------------------------------------------------------------------MSKNIKR--------------------------------------------------------------------------------------------------------------------------------------------------DINQENLSLAVKIVTPTWESI--KEDFDW---YCTKIEETFFQNDTTKKELFTL--PKFE---------------EELTDDVVNKRLFKHSS--AVLNFMEC-IVQF-M---N---GN--EETKPVL-FVLGRNHYTIG---VNEKLFLEMKDAICSVIKY--KIGTE---------NA-KAWDTILQYIL-----------INYV-------FE-GMTM-------------------------------------------------------------------------------------------------------------------------------------------------------------------

>Phlebotomus_sandfly_GbXL

-----------------------------------------------------------------------------------MGCQLGK-----------------------------LSGVSK------------------------GEDGQA-----------------------------------------------------------FAPNEMP--KQ-AKT-------DSRLPLTAKQKYSMLASWKGI--SRAMEP---TGVCMFIKLFEEHADLLNMF----TKFK-----EM-K----TKEQQA---KSEELAEHAN--KVMETLDE-GIRS-L---D---DL--DAFFEYL-HQVGSSHRRIP--NFKADYFWKIEKPFLSAVET--TLGDR-YTPN----VE-GIYKLTIKFII------------ETL-------IT-GFNK---S-----ANSAA------------------------------P-----------ANSDTVNVNTKS-----------------------------------------------------------------------------------------------

>Phlebotomus_sandfly_Glob1

----------------------------------------------------------------------------------------------------------------------------------------------------------------------------------------------------------------------------------------------MPSLTPEQIEIVKSTWVTV--ANAPED---SGEAILLRFFEKYPHNQKYF-----PFR-----NV-P----R-ENLK---GSAMFRSHAG--RVIAVFQK-SVDA-FNTAD---PV--GTLVEIW-TEIAKTHFKRN---ITQTSFNELKEVILEVLTA--ACK---LDGV----QQ-TAWAVTLDTIF------------EII-------SK-ELVK---L-----GQTSQ-----------------------------------------------------------------------------------------------------------------------------------------------------

>Seed_shrimp_Puriana_HbL2

-----------------------------------------------------------------------------------MAC-----------------------------------------------------------------------------------------------------------------------------------------------------------LTDDQKCLIHQTWCCS--IKKRPT---VGVAYLLTVFSTYPKTQCFM-------------PT-A----A-ENMC---TNPELRTVAW--QIMQKMSN-LIES-L---D---DT--PAFEDLVCQEMTELVCRYK---IYTPDFRRLIDLFLCIMED--HECVK-RSNDCRG-AV-MAWRTFRDIII------------QRL---CTVQQQYGIMQ---A-----KENFTC----------------------------------------------------------------------------------------------------------------------------------------------------

>Hackberry_petiole_gall_psyllid_GbX

-----------------------------------------------------------------------------------MGNAGTT-----------------------------RRNSLF--------------------LKQESGEE--GHKKE-------------------GVTHSIARRLTLSGGKGLGLGSRQNT---LIEEESVPP-------C-VNN-------PPPDPLTDEEKKMLSETWKVL--EDDIAK---VGVITFISLFETHPDVQQVF----MPFS-----GI-E----L-EDLK---HSKQLRAHAL--RVMAFVQK-AIAR-L---Y---EP--DKLDTLL-RDLGKKHFTYG---AKAKYVDLIGPQFISAIQP--ALESR-WSTE----LH-NAWVHLFAYMA------------HIM-------KE-SMAA---E-----ELCHK------------------------------K-----------S----------------------------------------------------------------------------------------------------------

>Subterranean_termite_HbL

-----------------------------------------------------------------------------------MSG--------------------------------------------------------------QTDESN----------------------------------------------------------------------L-DTP-------GPSTGLTPRERQIVVDTWGVV--KRNAKE---AGVEMFTRLFEAHPQYQKLF----PNFE-----GL-T----L-SVLR---TSKKLAAHAT--NVMYSLTS-VIDN-L---D---DP--ECLKELL-IKLGKNHGRHK---VYEKQFHDLELVLMELLKE--KLGNQ-LTPQ----GE-VAWKKTIDIVY------------KGI-------FQ-GMRA---Y-----DASSV------------------------------K----------------------------------------------------------------------------------------------------------------------

>Kissing_bug_GbXL

-----------------------------------------------------------------------------------MGCELGK-----------------------------LTKGGG------------------------NSRDAG------------------------------------------------------------DAREPPPQPP-APT-------DPRLPLTAKQKYSMMASWKGI--SRAMQP---TGVYMFIKLFEEHEELLGLF----AKLK-----EL-R----TKEEQA---ESVELQEHAT--KVMSTLDE-GIKE-L---D---DL--DTFFSFL-TQIGQSHRKIP--GFKPDYFWKIEKPFLEAVKM--TLGDR-YTEN----VE-NIYKVTIKLII------------ETL-------EK-GYNT-------------------------------------------------------------------------------------------------------------------------------------------------------------------

>Itch_mite_HbL2

----------------------------------------------------------------------------------------------------------------------------------------------------------------------------------------------------------------------------------------------MTEFEREEIEVLREQWDRI-VHYHQEC---FGMKLFQRLLQLHPEYRPLF-----GFE-----ET------V-EEIQ---NTQRLKAHGI--NVVYMLNM-LFDN-F---D---DM--DMIDELI-FKLVKLHMMRG---IDQIWLDDIIEPFELVLEE---FNAK-IQIERI-----EVLRKAFIFIK------------NRM-------QE-LYDENVVAKMLDTSDISEY----------------------------------------------------------------------------------------------------------------------------------------------------

>Itch_mite_GbXL

-----------------------------------------------------------------------------------MGCPLSK-----------------------------TSDEND------------------------VQSKQN----------------------------------------------------------DFSNEVMV--TN-KTV-------DPRLPLTVRQKFNLSKSWKGI--SREMEM---TGVLMFVKLFEETPEILNLF----TKFQ-----EL-K----TKDSQM---KSMELAEHAT--KVMTNLDE-MINS-L---D---DM--DYFFRHL-HSLGKYHRRIP--GFHKDNFLKLEKPFIEAVKE--VLQER-YTEN----MA-NIYNIIIKLIL------------QTI-------SE-GFEK---D-----FD--------------------------------------------------------------------------------------------------------------------------------------------------------

>Itch_mite_HbL1

-----------------------------------------------------------------------------------------------------------------------------------------------------------------------------------------------------------------------------------------------MSLTNRDKEIIVSTWSLI--RKDSDQ---AGIHLFKRFFEANPDYVKYF-----PFG-----DLDD----L-EKIL---VDPRLKWHAS--RVMAALST-IVDN-L---D---DP--VCFEDSL-QKVLSSHLNRK---IQLYHFENLKKALVCLFMD--KLGPDIMNDE----TI-EAWSKAYDVIL------------DTY-------RS-RLSE---A-----KSSIS-----------------------------------------------------------------------------------------------------------------------------------------------------

>Itch_mite_HbL3

M---------------------------FQK----------------------------------------------------LRSRLSN------------------------------------------------------------------------------------------------------------------------------------LSDSDDE----LKAENQTELTSKEIDFVRNTWSLL--RNDIARFKFLGGELFVRFFTKYPDYQRQF----KSFK-----DV-PMDFQRNRDIR---FNKKLMAHGT--YVMYTIGM-LVDN-L---E---RP--LMMEQML-KRLARNHYRRK---ISLIAFDRLRNTFLEHLAE--ILGPKIFTKK----VS-IAWSKAFNYLL------------MEI-------EK-NFKI--------LESDLERSGSYCRLNSMHLAARNDLVRLQRLKESGKKKTLNVNNDCCGSSSEQNLIHQRIRIESPA-----------------------------------ANIKQIRHNSQCSA----------PIIDERREAERSKSFLTKTISFLRKNRL--

>Eri_silkmoth_HbL

-----------------------------------------------------------------------------------MGNWISQ-----------------------------F-----------------------------WWGGD----------------------------------------------------------------------P-DEV-------NPISGLTRREIYAVQKSWAPV--FANSIP---NGAELLRRLFQTFPETKEFF----KMIR-----KL-P----D-EEYI---QNPQFRAHVI--NLMTSLNL-AVNN-L---N---QP--EVVAAMM-NKLGESHKRRQ---IKERHFGDLKQVIVTMFIE--VLH---LDGD----TL-TAWDKTVTFWY------------KHI-------FE-TLNT---A-----EESR------------------------------------------------------------------------------------------------------------------------------------------------------

>Armyworm_GbXLa

-----------------------------------------------------------------------------------MGCQLGK-----------------------------LAASER------------------------RGNNAG------------------------------------------------------------PAISDG--PP-PAT-------DPRLPLTAKQKYSMLASWKGI--SRAMEK---TGICMFIKLFEENQDLLNMF----EKFR-----QC-R----TKEEQI---NSMELAEHAN--NVMNTLDE-GIKG-L---D---DL--DNFFGYI-HQVGASHRRIP--GFKVEYFWKIEAPFLAAVES--TLGDR-YTPN----VE-NIYKITIKFIL------------ETL-------IE-GYEA---A-----GKHPT-----------------------------------------------------------------------------------------------------------------------------------------------------

>Armyworm_GbXLb

-----------------------------------------------------------------------------------MGCELSK-----------------------------LASSEF--------------------------------------------------------------------------------------NNHDALDRPP-----PPA-------DPRSPLTTKQQYCMLASWKGI--FRQIEK---TGILLFIKLFEENEDLLHLF----EKFG-----EL-K----TAEAQM---SSEELAEHAT--KVMHTLDE-GIKG-L---G---DI--DAFFAYI-RHVGATHHQVP--GFKAENFWKIEQPFLQAAKT--TLADR-YTPN----IE-EIYKKTIRFIL------------ENL-------VK-GYED---S-----AVENG------------------------------N-----------GQS--------------------------------------------------------------------------------------------------------

>Armyworm_HbL1

-----------------------------------------------------------------------------------MGGLLSY-----------------------------I-----------------------------WWGGD----------------------------------------------------------------------P-DEV-------NPISGLTRREVNLIQKSWAPV--NADKAA---NGAELLRRFFTAYPAAKEFF----KMIK-----GM-P----E-EQYL---ENPQFKAHVI--NLMTALNL-AVEN-L---N---QP--EVVAAMM-NKLGESHGRRK---IKEQNFQDLKQVIVKMFIE--VLK---LDDT----TL-GAWGKAVDFWY------------KHI-------FE-TLNK---A-----EQTR------------------------------------------------------------------------------------------------------------------------------------------------------

>Armyworm_HbL2

---------------------------------------------------------------------------------MGVGDIVTR-----------------------------W-----------------------------WWGGD----------------------------------------------------------------------P-DER-------NKVSGMSLRDVHNVQKSWAVI--QANSNG---NGFLMFFRLFEAEPETKLFF----KNLA-----HIHT----E-AEMS---ANVSFRAHII--NIMSSFDT-SIQN-L---D---KP--ELVVAWM-QKLGDSHRRHR---IEKRHFHVFKDVLVTILQK--DLK---LDPQ----VV-ASWDRYVEFIY------------EHL-------LS-RLAS-------------------------------------------------------------------------------------------------------------------------------------------------------------------

>Red_fire_ant_GbXL

-----------------------------------------------------------------------------------MGCELSK-----------------------------LASSKS------------------------RNQAGN-----------------------------------------------------------DGSSPPP--PP-AAT-------DPRLPLTARQKFTVIASWKAV--SRALEP---TGIYMFIRLFEENAELLNMF----TKFR-----DQ-K----TKEQQS---TSMELAEHAK--TVMSTLDE-GIKS-L---D---DM--DAFLTYL-HEVGASHTKIP--GFNRQYFWKIEKPFLDAVER--TLEDR-YSEN----VE-NIYKLTIKFII------------ETL-------ID-GFDK---A-----QSDKA------------------------------K-----------S----------------------------------------------------------------------------------------------------------

>Red_fire_ant_HbL

-----------------------------------------------------------------------------------MALFRGL-----------------------------F-----------------------------HFFLN----------------------------------------------------------------------D-NKV-------DEKLGLTEKQKRLVQNTWAIV--RKDEVS---IGVALVLAYFKQYPEAQKEF----KAFK-----DV-P----I-DELS---KNKRFQAHCA--NIVATIGK-VIEQ-M---H---DP--ELMEASV-INFTEKHKNRG---QTQKQFENLKQMMLDVFPS--VFGKQ-YTPE----VQ-EAWKKMLGLIY------------SKI-------YQ-TLKD-------------------------------------------------------------------------------------------------------------------------------------------------------------------

>Scorpion_HbL

-----------------------------------------------------------------------------------MGIVWSV-----------------------------FTG---------------------------KPEYH----------------------------------------------------------------------L-DIP-------DPITGLSLRDRKEITDSWHIL--RKDIKS---AGTQFFIKLFIEHPTLQKLF----PAFS-----DE-P----F-SELQ---TNKKLIAHGT--IVMYSISS-MVDN-L---E---DA--ECFKVLA-ANVAQSHYNRG---VTYEHFSKLGPVMLSLLEE--FLHRS-LSVP----TE-VAWQKFLSVLV------------ATA-------KD-IADR---D-----QNQTN------------------------------S-----------S----------------------------------------------------------------------------------------------------------

>Acorn_worm_Gb1

-----------------------------------------------------------------------------------MA-------------------------------------------------------------------------------------------------------------------------------------------------------DPVTTLTSDEVAAIKSSWSAV--YDKKKE---SGVTLFVKLFTENPSFKSQF----GYMS-----GV-A----D-GDMK---TLPALENHGV--KVMDRINE-WMGN-L---T---NG--AELVKQL-KHLGTTHIALK---VTEDNFNAMDSVLMYTLQE--QGGSA-FTPA----AK-AAWQKAWGVMK------------SVI-------VG-ALKG-------------------------------------------------------------------------------------------------------------------------------------------------------------------

>Acorn_worm_Gb10

-----------------------------------------------------------------------------------MGCSNST--------------------------------------------------------------------------------------------------------------------------------------------------ADKPKLTVEQKRLIIDSWKEL--HIDLER---IGMLMFMGMFGTHPQTREFF-----NFR-----GT-S------DDPK---NTQRLREHGL--RFMSLVKK-ILVF-I---D---EK--PRLDAML-LDLGRRHQEYK---ADFNLIDVFGEQFILSVRP--TLKHS-WNPD----VE-SAWAQLFKYIS------------YMM-------KK-GMMQ---T-----DKNK------------------------------------------------------------------------------------------------------------------------------------------------------

>Acorn_worm_Gb11

-----------------------------------------------------------------------------------MGCANSH---------------------------------------------------------HVQTNSSLKL-----------------------------------------------------------------QKQ-KTV-------DSAVSFTDRETAILRSTWPLL--ASDMTR---NGGKIFLQIFAVAPHVKDLF-----PFR-----YV-P----N-DMLQ---QNEIFKMHGR--RFMQSVGA-VIEN-I---D---NLD-GDISILL-HNLGKRHTDFD--EVDGAYFDIYTDCMMHTWRS--SLGNELFTPD----VG-QVWHKLFDFII------------NCI-------KD-GYFL---A-----MKNKK------------------------------N-----------NSDEVKKI---------------------------------------------------------------------------------------------------

>Acorn_worm_Gb12

-----------------------------------------------------------------------------------MGCTPSI----------------------------------------------------------------------------------N---------------------------------------------ERD--FQ-TPV-------DDKHLLDDRQKRIVRKTWRPL--ANDMTE---NGQKIFINIFESHPEIKYMF-----PTR-----DI-E----GRDNLS---ANPHFRMHSS--RFMQSVGA-AIDN-L---N---DLD-NALRPLL-VKLAKTHVRFK--GFKPDYFDAFEEAMLSVWQE--ELGQR-FTTE----VE-ESWKLLFFYIK------------DCL-------KE-GYDI---A-----MNEKT------------------------------SGELN-------NSDFIN----------------------------------------------------------------------------------------------------Q

>Acorn_worm_Gb13

-----------------------------------------------------------------------------------MGCSVST---------------------------------------------------------STENGKN---------------------------------------------------------------------FV-QLS-------DSVDIFTERQRRIVRKTWRPL--ANDMTG---NGTKVFLHIFEMNPKVKQLF-----PCR-----DK-T----G-EELL---KDLNFKGHAS--RFMQSVGA-AVDN-L---D---NLE-TSLAPLL-MNLGKSHNHFS--GFELNYFDSFTGAMLHVWEL--ELQDR-FTPE----VM-EAWKLVFDYMM------------GKM-------KD-GYIT---R-----RDEKL------------------------------N-----------ETNEQK------------------------------------------------------------------------------------NKIIV------------

>Acorn_worm_Gb14

-----------------------------------------------------------------------------------MGCTESI-----------------------------------------------------GTAVAGMRGNNVH----------------------------------------------------------------------------IANLSKSPRFSSEQISELRRTWPKL--ACDLTG---NGAQVFLQIFAINENIKILF-----PFR-----YV-P----V-DILS---QNEVFRGHSR--RFMQAVGA-CVEN-L---E---NLD-GDVTTLF-VGLGKKHIHFE--GFKVDYFSTYVTSMQTVWDI--ALTGHHYDKQ----TK-QSWTQIFEFVI------------TRM-------AE-GYHI---A-----MDEQEA-----------------------------K-----------KKNQLA--HEN-------------------------------------------------------------------------------GKII-------------

>Acorn_worm_Gb15

-----------------------------------------------------------------------------------MGCTVST-----------------------------KPDLYS-------------------------------------------------------------------------------------QNGDADPYKKK--KV-APV-------DSRLPLTARQKFQITKSWKGI--ARNMEN---TGKSMFMRLFQSNIELKNMF----TGFE-----EF-D----DLEDMR---ESQQLENHAS--LVMYTIDE-AIAS-I---D---DI--DFVVELL-GKIGRTHTRTD---FNPQLFWRIEQPFLSAVKE--TLEDR-YTKN----IE-EIYKITFRFIV------------DAL-------ID-GVVA---G-----VNERKA-----------------------------------------EAEAEAEAKAEAEDEQDN-----------EMMKKTM----HN-----GD-----KEDNCIESS-------------------------KQKDITVIASTKQR-----

>Acorn_worm_Gb16

-----------------------------------------------------------------------------------MGCLISS-----N--------------------------------------------------------------------------------------------------------------------------------------------GEHPMITKEQTKILTSTWHSI--HGDLEK---IGLLMFMGMFDNYPETRQFF---GLSGG-----SI-V----L-EDPA---VIQKIREHGL--RFMTTARK-LVMN-L---D---DK--DKFDRIL-LDLGRRHHGYK---ADVDLIEVFGQQFIASIQP--TLKDN-WNPA----VG-EAWEQLFKCVS------------SRM-------KD-GFLQ---A-----QSSPS------------------------------N-----------TELLK------------------------------------------------------------------------------------------------------

>Acorn_worm_Gb2

-----------------------------------------------------------------------------------MESTESN-----------------------------------------------------TPVKETTEINP----------------------------------------------------------------------D-DIP-------DEVTILTPKEVKAISESWKVV--YAKKKE---NGVALFIRLFQSVPGSKSLF----KNLD-----GI-D----DEEKLR---NHPRLKAHGF--RVMSSVNS-LIES-L---E---EG--ELLVQLL-KDLGSSHSKNK---VTSSHFDALGPVIIWLLQK--ENGDS-FTPA----VK-NAWLKGWGVMK------------SVI-------VG-SLEE---A-----YAKMKT----------------------------------------------------------------------------------------------------------------------------------------------------

>Acorn_worm_Gb3

-----------------------------------------------------------------------------------------------------------------------------------------------------------------------------------------------------------------------------------------------MALSAGEIKLVTDSWTAV--YANKKA---NGVALFVRLFSENPGFQSQF----RYLD-----GV-S----GLAAIE---KTPALGDHAV--KVMDTINS-WIGS-L---G---DS--SAMVAKL-TALGTSHIALK---VTPANFDAMGPVLLWMLQE--KAGGA-FTPA----AK-DAWAKGWDLMK------------SHI-------VK-ALQG-------------------------------------------------------------------------------------------------------------------------------------------------------------------

>Acorn_worm_Gb4

-----------------------------------------------------------------------------------------------------------------------------------------------------------------------------------------------------------------------------------------------MSLSAGEIKLVKDSWAPV--YANKKE---SGIALFVRLFSENPGFQSQF----RYLD-----GV-S----GLAAIE---KTPALADHGV--KVMDTVNS-WVGS-L---G---DA--PALVKQL-TALGTSHIALK---VTPANFDAMGPVLLWTLQE--KAGGA-FTPA----AK-DAWAKGWDLMK------------SHI-------VK-ALQG-------------------------------------------------------------------------------------------------------------------------------------------------------------------

>Acorn_worm_Gb5

MHRKAVAIL----------------------------------------------------------------------YVVVMAELMSYAV---------------------CLPVNGTSDALTFDEF---AKI----ERANLMQYQSAKLAKL-----------------------------------------------------------------------EVP-------NAVTTLTPSEAIAIQSTWLFV--YEDKEE---NGVELFVKLFTEHPDYQALF----GYLE-----GI-V----GIENIK---NVPFLRVHAS--HVLIYLNT-MLES-L---N---DG--TILVELL-KTLGYTHVGLN---LTPEHFDALGPILISLLQE--KGGDS-FTPF----AE-KAWLKGWGVMK------------SVI-------VG-ALEN---G-----YQLEDGLG--------------------------Y-----------------------------------------------------------------------------------------------------------------------

>Acorn_worm_Gb6

-----------------------------------------------------------------------------------MGCTSSA-----------------------------ASDRPS---------------------------------------------------------------------------------------KNDPLLDPP--PP-QEL-------DPRIPLTARQKFSIQKSWKAI--QRNMEG---VGMDIFIRLFKAHPEYQDLF----PEFK-----GM-S----E-EKLR---NSINFETHVG--IFMNVIDE-CIDS-L---E---DA--DHVINLL-TKKGRKHANYG---VKPEFISDIEEPFLASVKQ--LLEDR-YSEK----IE-EIYKLTIKFIL------------EHF-------IN-GLKE---SV--------------------------------------------------------------------------------------------------G-----------------------------------------------------------

>Acorn_worm_Gb7

-----------------------------------------------------------------------------------MGCSNSS--------------------------------------------------------------------------------------------------------------------------HNCVSPKKE--DS-MEQ-------LPPTSLTDQHRVILLDSWKVI--QEDIAK---VGVIMFMGLFETHPECKEVF----MPFK-----EL-Q----G-DDLR---WSSALKAHGL--RVMAVIER-VLAR-I---D---SD--EKIEEHL-KALAKKHVEYG---ANSDLVRLFGPQFIGSMKR--QLHKS-WSDE----MQ-DAWTVLFDIII------------YHM-------TT-NMVP---E-----QPENN------------------------------N------------------------------------------------------------------IAKRQKSSRKSRT--------------KY-----------MIDNGHSQ----

>Acorn_worm_Gb8

MSRFTSRLSSSTLDNFEAISNLGWEKKLYEHSSTRTF------------------RTRKRKLTIHNYTIY-PAGLFLLTVYNAMGCGSSK------------------------------------------------------------------------------------------------------------------------------------ING-NVV-------EEKPELTKEQKDTLIQTWQNL--HADLER---IGMLMFMGLFEHNPEIKEFFV--GADSR-----DM-K----T-EELR---YNEKLQEHGI--RVMGLVEK-IISS-M---GF--ED--EKIDQMV-VDLGKRHLGYD---VHIPFIDLFGRQFVFAIKP--TLHTH-WTAN----VE-EAWTQLFKYIG------------YLM-------RY-GYHT---K-----LQQVQ------------------------------K-----------KNS--------------------------------------------------------------------------------------------------------

>Acorn_worm_Gb9

-----------------------------------------------------------------------------------MGNE------------------------------------------------------------------------------------------------------------------------------------VA--KS-SRS-------STSQSLSKEQEKILVQTWLSI--RGDLER---IGLLMFTGLFEHHPEAKVMF---GLSDT-----AM-S----P-KDKE---NTALIKEHGL--RFMNVVRD-VLTL-I---S---EKNGSQAECVL-IDLGRRHCSYN---ADINLIDVFGQQFIASIQP--TLTGS-WDKK----VE-DAWIQLFKYIA------------FTM-------KQ-GLAA---E-----LIDKS----------------------------------------------------------------------------------------------------LKLNGKP------------------------------------------

>Cotton_leafworm_HbL

-----------------------------------------------------------------------------------MGALLTY-----------------------------I-----------------------------WWGGD----------------------------------------------------------------------P-DVV-------NEISGLTRREVYLVQKSWAPV--NADKVN---NGAELLRRFFTAFPASKEFF----KMIK-----NV-P----D-DQYX---TNPQFKAHVI--NLMTSLNL-AVEN-M---N---QP--EIVAAMM-NKLGESHGRRK---IQEKNFLELKQVIVKMFIE--VLK---LDDT----TL-GAWGKTVDFWY------------KHL-------FE-TLNK---A-----EQTR------------------------------------------------------------------------------------------------------------------------------------------------------

>European_centipede_GbX

-----------------------------------------------------------------------------------MGVESSK-----------------------------TSKL----------------------CRHGQNAAPA----------------------------------CVNSGMSTSMTGKEFC-------------ENE--NA-YDD-------VESWVLDEREIEHVIFTWKLV--ERNIAK---VGVITFLGLFETHPAVQSVF----LPLS-----HM-S----R-EQLG---SSAKLEAHAL--KVMNFIQK-IIAR-I---D---NP--SKVHALL-RQLGKNHFHYG---VKREYIDLVGPQFVIAIRPLLEMESA-WTAA----IQ-DSWLHLFASMS------------AIM-------KK-SMGI---E-----EEQYL------------------------------I-----------TK---------------------------------------------------------------------------------------------------------

>European_centipede_GbXL

-----------------------------------------------------------------------------------MGCSFVK-----------------------------HSNGAG---------------------EEGRSAKSSAGK----------------------------------------------------SGVVNAVDTPA--AP-APV-------DPRLPLNARQLFQIGKSWKGI--SRAMEY---TGVNMFIKLFEEHNELLNLF----TKFS-----DL-K----TKEQQA---ESLELQEHAT--LVMTTLDE-SIQA-L---E---NV--DAFTAYL-HQVGRSHTRVP--GYKKEYFWRIQKPFLEAVSE--TLGDR-YTEN----ME-TIYTVTIQFIL------------ETL-------VK-GFEI---G-----EKEKGV----------------------------------------------------------------------------------------------------------------------------------------------------

>Vietnamese_centipede_HbL

------------------------------------------------------------------------------------------------------------------------------------------------------MDPS----------------------------------------------------------------------L-DIS-------DPVTGITARQKLVVRENYGRG--AKNLKS---NGVEFFVALFTKHESLKKYF----VTLV-----DL-P----I-EALP---SSKKLQAHST--TVMMAISG-LVDN-L---E---DV--ECLKELL-LKIGENHSRRK---VSIEEFKKLAVVFVDFLEE--KLGDG-FTSF----AR-KAWEEVFRVMN------------SII-------EE-GLKS---A-----TGR-------------------------------------------------------------------------------------------------------------------------------------------------------

>Velvet_spider_GbXLb

-----------------------------------------------------------------------------------MGCTFAK-----------------------------VPKDGK------------------------GSVQDL----------------------------------------------------------NHAGDAPS--AP-PAQ-------DPRIPLTARQKFSISKSWKAI--ARAMEQ---TGVTMFTKLFEENEELLELF----EKFK-----HM-K----SREERE---QSEELREHAT--TVMTTLDE-SIMS-L---D---NV--DQCIDYL-RNVGRSHRKIK--GFKSEYFWKMEAPFLAAVKE--TLEDR-YTEN----ME-SIYKITIHFIL------------QTV-------IE-GFEG---T-----QQQNSV----------------------------------------------------------------------------------------------------------------------------------------------------

>Velvet_spider_HbL

M----------------------------------------------------------------------------------MGFAVSK-----------------------------LSWF--------------------------WTSAG----------------------------------------------------------------------Y-DTP-------DPATGLTPRQKDIVRNTWKSI--RADTRN---NGIKLFLKFFEAYPEYQLLF----KSFA-----NV-P----L-SDLP---RNGRLLGHVT--SVMYALNS-VVDN-L---E---DP--ECLIEIL-QKTGISHRPRN---VNRQHFNNLKVVLIKLLVE--ILGSN-VMNE----SAVEAWEKTLDVAN------------SII-------IK-SLEA---E-----GDA-------------------------------------------------------------------------------------------------------------------------------------------------------

>Velvet_spider_GbXLa

-----------------------------------------------------------------------------------MGGNLSK-----------------------------ALTALQ------------------------KKGEPE--------------------------------------------------------TVDAPLEDPP--TP-PAP-------DPRLPLTARQLFNISKSWKGI--ARAMEP---TGITMFVKLFEDNEDILHLF----QKFQ-----YK-R----FHEFHR---DSMELAQHAG--IVMSTLDE-SIKK-L---N---NV--DYFMDYL-HSVGKLHTKIP--GFQRDYFWRIERPFLEAVQD--TLGDR-YTDN----ME-NIYKITIRYIL------------DTV-------VK-GFDL---G-----ALQTK------------------------------T-----------SEVPPPKASPEPSPSPQEH---------RDE-----------------------QEGKCVNSQ------------------------------CPAVTNGQS-----

>European_centipede_HbL

MDDIFHHIK----------------------------------------------------------------------SIVTMGGWISY-----------------------------FWPQKSA---------------------EFDVSPG----------------------------------------------------------------------L-DEV-------ESASGLTLRQKKVVTEIWDLV--KIDIKQ---NGIDFFIEFFKAFPLNLNNF----KAFQ-----NM-T----D-DQLR---KSKKLEAHAT--NVMYAIST-VVDN-L---Q---DV--ECLTELL-STIGRNHIKRK---ITPVQFDQVGITFIKFLEN--KLGSR-ITPF----CR-NAWEVTFKVMN------------SII-------VA-GLQS---N-----DD--------------------------------------------------------------------------------------------------------------------------------------------------------

>Remipedia_Speleonectes_HbL

-----------------------------------------------------------------------------------MSLLLGF-----------------------------------------------------------MYTGE----------------------------------------------------------------------G-DDP-------DPATGLSSAQGKAVADTWAVV--RKDLKQ---HGTKILIELFKAHPQYQAKF----KGFA-----NV-P----L-EDLP---RNKKLHAHAC--NIMFTIDN-MICN-L---N---DI--DVLTEVV-MKVGRSHKPRD---LTMSDLQNLAKVAQDYLAH--TLGGQ-MTAA----GK-EGWAKVFAVVT------------KVM-------DE-GMRA-------------------------------------------------------------------------------------------------------------------------------------------------------------------

>Red_flour_beetle_GbXL

-----------------------------------------------------------------------------------MGCELGK-----------------------------LASSGG------------------------SGNHRK-------------------------------------------------------------PDESA--PP-ATV-------DPRLPLTAKQKYNMLASWKGI--SRAMES---TGVCMFLKLFEEHAELLTLF----EKFK-----EL-K----TKEDQA---NSLELAEHAS--TVMNTLDE-GIKE-L---D---NL--DTFFEYL-HQVGASHRRIP--GFKVEYFWKIEKPFLTAVET--TLGDR-YTEN----VE-NIYKITIKFII------------ETL-------VK-GYDN---A-----NAPT------------------------------------------------------------------------------------------------------------------------------------------------------

>Red_flour_beetle_HbL1

-----------------------------------------------------------------------------------MGIITS------------------------------TLS---------------------------YYMTK----------------------------------------------------------------------T-NDP-------DPITGLTSRDRYVIQTSWAPV--KKDLTG---NGVALLLLYFEKFPATKNYF-----VFK-----DV-P----N-EKLK---TDKKFHAHCN--SVMVTLDS-LIAN-L---N---DG--ELIVSLL-EKLGKNHKRHG---IKDDAYDQLKETVIELFSS--F-----MTKE----EL-ETWDKLLKVAF------------SVI-------IK-YL---------------------------------------------------------------------------------------------------------------------------------------------------------------------

>Red_flour_beetle_HbL2

-----------------------------------------------------------------------------------MGQLIS------------------------------LYK---------------------------YYTTR----------------------------------------------------------------------T-DDP-------DPLTTLTSREVFLVQSSWDPI--KKDLTG---YGVQLLLFLFKKYPEEQQNF-----PFR-----DM-P----F-EELG---ASKKFHAHCS--NVMYAVDS-IIDS-L---K---DG--ELLVNIL-EKIGRNHHRNT---VKPISFWHVKETMLEFFKK--M-----MNDE----TL-KAWDKALQVAF------------GVV-------AK-ELDK---K-----N---------------------------------------------------------------------------------------------------------------------------------------------------------

>Frog_GbX

-----------------------------------------------------------------------------------MGCILSS-----------------------------LGW-----------------------QWRDSLDHT---------------------------------------------------------------------ET-SPL-------LPTLNLSEQQQQLLVESWRLI--QHDIAK---VGVILFVRLFETHPECKDVF----FLFR-----DVDD----L-QALR---ANKDLRAHGL--RVLSFVEK-SVAR-I---A---DC--ARLEELA-LELGRSHYRYN---APPRYYQYVGTEFISAVCP--MLHDK-WTAE----VE-EAWKGLFAYIC------------TVM-------ER-GYQE---E-----ERRHS------------------------------D-----------GRSL---------------------------------------------------IDGLQGNKGLI-----------------------------------------

>Frog_GbY

----------------------------------------------------------------------------------------------------------------------------------------------------------------------------------------------------------------------------------------------MADLTGADIENINEVWSKI--YANPEE---SGRTVVISLFLTYPQTKIYF----KNLK-----NI-S----TLQEMQ---DNAGIRAHGK--RVMGALNH-VIEN-L---K---DW--DAVCSAL-SHLAKRHQDVH--KVEVNNFELLFLVIISVFKE--ALGSG-FTPE----QS-KSWEKLFSITY------------KYL-------ES-CYAN---TD---S----------------------------------------------------------------------------------------------------------------------------------------------------------

>Zebrafish_Ngb

----------------------------------------------------------------------------------------------------------------------------------------------------------------------------------------------------------------------------------------------MEKLSEKDKGLIRDSWESL--GKNKVP---HGIVLFTRLFELDPALLTLF----SYST-----NCGD----A-PECL---SSPEFLEHVT--KVMLVIDA-AVSH-L---D---DL--HTLEDFL-LNLGRKHQAVG---VNTQSFALVGESLLYMLQS--SLGPA-YTTS----LR-QAWLTMYSIVV------------SAM-------TR-GWAK---N-----GEHKS------------------------------N----------------------------------------------------------------------------------------------------------------------

>Termite_GbX

-----------------------------------------------------------------------------------MGNASSH-----------------------------HKVGGIG-------G-------VSAGSEKTVRGDVTASGNEQ-------------AK--EGDTKPGSKVAANNHQDHQQHRKRSSVI-GGGEREGD-----------IAV-------VRPEELTERQKELLEETWKEL--EGNIAK---VGVITFISLFETHPDVQQVF----MPFN-----GI-E----L-EDLK---HSKQLRAHAL--RVMAFVQK-AVAR-L---H---EP--EKLETIL-QELGKKHYTYG---AKQKYVDLIGPQFIQAIQP--SLEDR-WTPE----LQ-EAWIHLFKYMA------------YVM-------KT-SMGE---E-----EQRIN------------------------------S-----------QQ---------------------------------------------------------------------------------------------------------

>Termite_GbXL

-----------------------------------------------------------------------------------MGCELGK-----------------------------LVMVHR---------------DA-------GEGSKG---------------------------------------------------------RHGEAGPPP--PP-EPP-------DPRLPLTAKQKYSMMASWKGI--SRAMEP---TGVYMFIKLFEEHQELLNLF----EKFR-----EL-R----TREEQA---NSMELAEHAN--TVMTTLDE-GIKG-L---D---NL--DSFFDFL-TQVGASHHRIP--GFKPEYFWRIERPFLEAVQM--TLGDR-YTEN----IE-SIYKITIKFII------------ETL-------VR-GYEE---K-----KPDT------------------------------------------------------------------------------------------------------------------------------------------------------

>Termite_HbL1

-----------------------------------------------------------------------------------MGSVLTY-----------------------------FWGR------------------------EPNDDPE----------------------------------------------------------------------L-DIP-------HPTTGLTPRERQAVVDTWAIM--KQDAKR---AGVELFIQLFEAHPEYQKLF----RVFE-----SL-S----L-QELE---KSAKLSAHAT--NVMYSLTS-VIDN-L---E---DP--ECLTELL-IKLGQNHDRHG---VSEKEFNDLKVVLMKLLKQ--KLGKK-LTSQ----AE-AAWSKTIDVAY------------QVI-------FE-GLKT---S-----DVASV------------------------------K----------------------------------------------------------------------------------------------------------------------
