# Supplementary material for: The Globin Gene Family in Arthropods: Evolution and Functional Diversity
Source: Front Genet. 2020 Aug 13;11:858. doi: 10.3389/fgene.2020.00858 (PMC7457136; doi:10.3389/fgene.2020.00858)
Supplement: TABLE S4 — GUIDANCE2-edited multiple sequence alignment. [file Table_4.DOCX]

>Aedes_mosquito_GbXL

--------------------------------------------PLTAKQKYTMVASWKGI-AMET---TGIHMFIKLFEEHAELLEMF-A-EEQA-TSEELQEHAN-KVMNTLDE-GIRGL--FFEFIHQVGASHRRGFKQEYFWRIEEPFLSAVSLRVE-GIYKLTIKFII----------------------------------

>Anopheles_mosquito_GbXL

--------------------------------------------PLTAKQKYTMVASWKGI-AMET---TGITMFIKLFEEHADLLNMF-A-EEQA-TSEELQEHAN-KVMNTLDE-GIRGL--FFEFIHQVGASHRRGFKQEYFWRIEEPFLSAVSLRVE-GIYKLTIKFII----------------------------------

>Cotton_aphid_GbXL

--------------------------------------------PLTAKQKYSMIASWKGI-AMEP---TGVYMFIKLFEEHQELLQLF-T-DAQA-NSMELAEHAN-KVMTTLDE-GIKEL--FFQYLTQVGATHKTGFDPDYFWKIEVPFLEAVKLRIE-TIYKITIKLII----------------------------------

>Pea_aphid_GbXL

--------------------------------------------PLTAKQKYSMIASWKGI-AMEP---TGVYMFIKLFEEHQELLQLF-T-DAQA-NSMELAEHAN-KVMTTLDE-GIKEL--FFQYLTQVGATHKTGFNPDYFWKIEVPFLEAVKLRIE-TIYKITIKLII----------------------------------

>German_cockroach_GbXL

--------------------------------------------PLTAKQKYTMMASWKGI-AMEP---TGVYMFIKLFEEHGELLNLF-E-EEQA-NSMELQEHAT-TVMTTLDE-GIRSL--FFQYLTQVGASHHRGFKPEYFWKIEKPFLEAVKLRVE-NIYKVTIKFII----------------------------------

>Termite_GbXL

--------------------------------------------PLTAKQKYSMMASWKGI-AMEP---TGVYMFIKLFEEHQELLNLF-E-EEQA-NSMELAEHAN-TVMTTLDE-GIKGL--FFDFLTQVGASHHRGFKPEYFWRIERPFLEAVQLRIE-SIYKITIKFII----------------------------------

>Bed_bug_GbXL

-------------------------------------------PPLTAKQKYSMVASWKGI-AMEQ---TGVFMFIKLFEEHQELLDLF-A-EEQE-KSLELAEHAT-KVMATLDE-GIKEL--FFTFLTQIGQTHKKGFKPDYFWKIEKPFLEAVKLRVE-SIYKVTIKLII----------------------------------

>Brown_marmorated_stink_bug_GbXL

--------------------------------------------PLTAKQKYSMLASWKGI-AMEQ---TGVYMFIKLFEEHEELLELF-A-EEQA-SSLELQEHAT-KVMNTLDE-GIKEL--FFTFLTQIGQSHKKGFKPDYFWKIEKPFLEAVELRVE-NIYKVTIKLII----------------------------------

>Kissing_bug_GbXL

-------------------------------------------PPLTAKQKYSMMASWKGI-AMQP---TGVYMFIKLFEEHEELLGLF-A-EEQA-ESVELQEHAT-KVMSTLDE-GIKEL--FFSFLTQIGQSHRKGFKPDYFWKIEKPFLEAVKLRVE-NIYKVTIKLII----------------------------------

>Milkweed_bug_GbXL

--------------------------------------------PLTAKQKYNMLASWKGI-AMEQ---TGVYMFIKLFEEHEELLGLF-E-EEQA-QSLELQEHAT-KVMHTLDE-GIKAL--FFAFLTGIGQSHKKGFKPEYFWKIEQPFLEAVELRVE-NIYKVTIKLII----------------------------------

>Glassy-winged_sharpshooter_GbXL

--------------------------------------------PLTAKQKYSMLASWKGI-AMEP---TGVYMFIKLFEEHEELLDLF-T-DAQA-NSMELQEHAT-KVMSTLDE-GIKEL--FFEYLHQIGASHRKGFKPDYFWKIEKPFLEAVKLRVE-NIYKITIKLII----------------------------------

>Brown_planthopper_GbXL

--------------------------------------------PLTAKQKYSMLASWKGI-AMEP---TGVYMFIKLFEEHRELLELF-T-DEQA-SSMELAEHAN-KVMTTLDE-GIKEL--FFEYLTNVGASHKKGFKPEYFWRIEKPFLEAVKLRVE-NIYQITIKLII----------------------------------

>Silkmoth_GbXLa

--------------------------------------------PLTAKQKYSMLASWKGI-AMEK---TGICMFIKLFEENQDLLDMF-E-EEQI-NSMELAEHAN-NVMNTLDE-GIKGL--FFQYIHQVGASHRKGFRVEYFWKIEAPFLAAVELRVE-NIYKITIKFIL----------------------------------

>Armyworm_GbXLa

--------------------------------------------PLTAKQKYSMLASWKGI-AMEK---TGICMFIKLFEENQDLLNMF-E-EEQI-NSMELAEHAN-NVMNTLDE-GIKGL--FFGYIHQVGASHRRGFKVEYFWKIEAPFLAAVELRVE-NIYKITIKFIL----------------------------------

>Monarch_butterfly_GbXLa

--------------------------------------------PLTAKQKYSMLASWKGI-AMEK---TGICMFIKLFEENQDLLNMF-E-EEQI-NSMELAEHAN-NVMNTLDE-GIKGL--FFEYIHQVGASHRRGFKVEYFWKIETPFLAAVELRVE-NIYKITIKFIL----------------------------------

>Postman_butterfly_GbXLa

--------------------------------------------PLTAKQKYSMLASWKGI-AMEK---TGICMFIKLFEENQDLLNMF-E-EEQI-NSMELAEHAN-NVMNTLDE-GIKGL--FFEYIHQ---------------KIETPFLAAVELRVE-NIYKITIKFIL----------------------------------

>Mountain_pine_beetle_GbXL

--------------------------------------------PLTAKQKYNMMASWKGI-AMES---TGVCMFLKLFEEHSELLLLF-E-EDQA-TSLELAEHAT-TVMSTLDE-GIKGL--FFEYLNQVGASHRRGFKAEYFWXXEKPFLEAVELRVE-NIYKITIKFII----------------------------------

>Red_flour_beetle_GbXL

--------------------------------------------PLTAKQKYNMLASWKGI-AMES---TGVCMFLKLFEEHAELLTLF-E-EDQA-NSLELAEHAS-TVMNTLDE-GIKEL--FFEYLHQVGASHRRGFKVEYFWKIEKPFLTAVELRVE-NIYKITIKFII----------------------------------

>Phlebotomus_sandfly_GbXL

--------------------------------------------PLTAKQKYSMLASWKGI-AMEP---TGVCMFIKLFEEHADLLNMF-T-EQQA-KSEELAEHAN-KVMETLDE-GIRSL--FFEYLHQVGSSHRRNFKADYFWKIEKPFLSAVELRVE-GIYKLTIKFII----------------------------------

>Flower_thrips_GbXL

--------------------------------------------PLTAKQKYTMLASWKGI-EMEA---TGVNMFIKLFEEHKELLNLF-E-EEQA-SSEELQEHAT-KVMNTLDE-GIRGL--FFSYLGQVGASHRRGFQSQYFWKIEGPFLKAVELRVE-NIYKVTIKLII----------------------------------

>Hessian_fly_GbXL

--------------------------------------------PLTARQKYTVIASWKGI-ALQP---TGINMFIELFEEHGELLGLF-N-EEQA-SSEELAEHAN-KVMETLDE-GIRAL--FFPFLHQVGGSHTRGFQAEYFWKIEQPFLSAVKLRVS-GIYIITIKFII----------------------------------

>Honey_bee_GbXL

--------------------------------------------PLTAKQKFTVMASWKAV-KLET---TGVFMLMRLFEENEELVQMF-S-EERF-DMVELGKHAE-KVMGALDE-GIRGL--FLTCLHQVGATHTKDFNPQYFWKIEQPFLEAVKLRVE-STYKVTIKFII----------------------------------

>Bumblebee_ GbXL

--------------------------------------------PLTAKQKFTVMASWKAV-KLET---TGVFMLMRLFEENEELVQMF-S-EERF-DMVELGKHAE-KVMAALDE-GIRGL--FLTCLHQVGATHTKDFNPQYFWKIEQPFLEAVKLRVE-STYKVTIKFII----------------------------------

>Carpenter_ant_GbXL

--------------------------------------------PLTARQKFTVIASWKAV-ALEP---TGVYMFIRLFEENAELLNMF-T-EQQS-TSMELAEHAK-TVMSTLDE-GIKSL--FLTYLHEVGASHTKGFNRQYFWKIEKPFLDAVELRVE-NIYKLTIKFII----------------------------------

>Red_fire_ant_GbXL

--------------------------------------------PLTARQKFTVIASWKAV-ALEP---TGIYMFIRLFEENAELLNMF-T-EQQS-TSMELAEHAK-TVMSTLDE-GIKSL--FLTYLHEVGASHTKGFNRQYFWKIEKPFLDAVELRVE-NIYKLTIKFII----------------------------------

>Nasonia_wasp_GbXL

--------------------------------------------PLTARQKYLLTASWKAI-AMEP---TGIYMFVKLFEENAELLNMF-S-EEQS-KSVELAEHAE-KVMNTLDE-GIQGL--FLTFIHQIGATHTKGFDREYFWKIESPFLAAMQLRVE-NIYKLTIKFII----------------------------------

>Midge_GbXL

--------------------------------------------PFTARQKYTMVASWKGI-AIET---TGVNMFIKLFEEHADLLNMF-T-EEQA-TSEELAEHAT-KVMETMDE-SIRSL--FFQFLHETGAIHTRGFTSDLFWKIEKPFLKAVSLRVE-GIYKITIKFVI----------------------------------

>Scarce_chaser_GbXL

--------------------------------------------PLSAKQKYNIMASWKGI-AMEP---TGVNMFVKLFEEHQELLSLF-E-EEQR-NSMELAEHAT-QVMRTLDE-GIKGL--FLEFVRQVGGTHHRGFHKDYFWRIETPFLDSVKLRMD-TIYKVTIKFII----------------------------------

>Twisted-wing_parasite_GbXL

--------------------------------------------PLTAKQKYNMLASWKGI-AMEP---TGVLMFIKLFEEHQELLNMF-D-EEQA-SSMELVEHAT-KVMRTLDD-SIKSL--FFDYVHQVGFSHQRGYKSDFFLKIEKPFLEAARLRIE-NIYQITIKFIL----------------------------------

>Body_louse_GbXL

--------------------------------------------PLTAKQKYNLVASWKGI-AMEP---TGITMFIKLFEQNEELLNLF-E-EQQA-SSMELQEHAM-NVMKTVDE-AIREL--FFIYLHQVGSSHRKGFKPDYFLKIEQPFLQAVKLRVE-RIYNITIKLII----------------------------------

>Green_crab_GbXL

--------------------------------------------PLTARQRFNIIKSWKGI-AIEP---TGVNMFVKLFENHSELITFF-T-DEQA-ESLELAEHAT-IVMNSIDE-GIKAM--FFDLLHQIGASHRKGFKKEYFWKIEHPFLEAVRLRMD-NIYRITIKLLI----------------------------------

>Scud_GbXLa

--------------------------------------------PLTARQKFSILKSWKGI-ALEP---TGVTMFVKLFERNAELLSLF-V-DEQA-ESLELAEHAT-VVMSSIDE-GIRAM--FFDLLHQIGGSHVKGFKKEYFWKIERPFLEAVRLRMD-QIYQLTIKFVL----------------------------------

>Mayfly_GbXL

--------------------------------------------PLTAKQKYNIIASWKGI-AMEP---TGVYMFIKLFEEHEDLLNLF-S-EQQQ-SSLELAEHAT-KVMGTLDE-AIRAL--LIAYLEAVGASHRRTFERNHFHRIEKPFLDAVRLRVD-VIYQATIKFII----------------------------------

>Muga_silkmoth_GbXL

--------------------------------------------PLTTKQQYCMLASWKGI-QIET---TGILLFIKLFEENEDLLHLF-D-EDQA-QSEELAEHAT-KVMHTLDE-GIKGL--FFAYVRHVGATHHQGFKAENFWKIEQPFLQAAKLRIE-NIYKLTIRFIL----------------------------------

>Armyworm_GbXLb

--------------------------------------------PLTTKQQYCMLASWKGI-QIEK---TGILLFIKLFEENEDLLHLF-E-EAQM-SSEELAEHAT-KVMHTLDE-GIKGL--FFAYIRHVGATHHQGFKAENFWKIEQPFLQAAKLRIE-EIYKKTIRFIL----------------------------------

>Silkmoth_GbXLb

--------------------------------------------PLTTKQQYCMLASWKGI-QIEK---TGVLLFIKLFEENEDLLHLF-E-EDLS-QSEELAEHAN-KVMHTLDE-GIKGL--FLAYIQHVGATHHQGFKAENFWKIEQPFLQAAKLRVE-NIYKLTIKFIL----------------------------------

>Monarch_butterfly_GbXLb

--------------------------------------------PLTAKQQYCMLASWKGV-QVEK---TGILLFVKLFEENEELLHLF-E-EAIV-SSAELAEHAT-QVMHTLDE-GIKGL--FFTYVRHVGGTHRQGFKAENFMKIEQPFLEAAKLRIE-NIYKLTIRFIL----------------------------------

>Postman_butterfly_GbXLb

--------------------------------------------PLTAKQQYCIMASWKGI-QIEK---TGIILFIKLFEENEELMHLF-E-EAIV-SSAELVEHAT-KVMHTLDE-GIKGL--FFAYVRHVGGTHRQGFKAENFLKIEQPFLEAAKLRIE-NIYKLTIRFIL----------------------------------

>European_centipede_GbXL

--------------------------------------------PLNARQLFQIGKSWKGI-AMEY---TGVNMFIKLFEEHNELLNLF-T-EQQA-ESLELQEHAT-LVMTTLDE-SIQAL--FTAYLHQVGRSHTRGYKKEYFWRIQKPFLEAVSLRME-TIYTVTIQFIL----------------------------------

>Bark_scorpion_GbXLa

--------------------------------------------PLTAKQCFSISKSWKGI-AMEP---TGINMFVKLFQDNEDLLDLF-E-ESNF-ESMELAQHAS-IVMSTLDE-SIRSL--LLDYLHSVGKLHHKGFQREYFWRIEKPFLAAVQLRME-TIYKITIHFIL----------------------------------

>Bark_scorpion_GbXLb

--------------------------------------------PLTARQRFSISKSWKGI-AMES---TGINMFIKLFEDNEDILHLF-K-EQQR-DSMELAQHAS-IVMSTLDE-GIRSL--FLDYLHSVGKLHRKGFNRDLXXKIEKPFLSAVQLRMD-SIYKVTIRFIL----------------------------------

>Velvet_spider_GbXLa

--------------------------------------------PLTARQLFNISKSWKGI-AMEP---TGITMFVKLFEDNEDILHLF-Q-EFHR-DSMELAQHAG-IVMSTLDE-SIKKL--FMDYLHSVGKLHTKGFQRDYFWRIERPFLEAVQLRME-NIYKITIRYIL----------------------------------

>Black-legged_tick_GbXL

--------------------------------------------PLTARQIFSISKSWKAI-AMEP---TGIEMFVRLFQEKEDLLDLF-E-ESQR-ESMELAQHAS-VVMTTLDE-GINAL--FMSYLHNAGRLHYKGFKKEYFWHIEGPFLAAVSLRIE-NIYKITIRFIL----------------------------------

>Western_predatory_mite_GbXL

--------------------------------------------PLTTRQIFSISKSWKAI-AMEP---TGVEMFVRLFKQNEELLDLF-T-ESQR-ESMELGQHAS-LVMTTLDE-GINSL--FLEYLHNAGGMHYKGFKKEYFWLIEKPFLEAVKLRIE-NIYNTTIHFIL----------------------------------

>Velvet_spider_GbXLb

--------------------------------------------PLTARQKFSISKSWKAI-AMEQ---TGVTMFTKLFEENEELLELF-E-EERE-QSEELREHAT-TVMTTLDE-SIMSL--CIDYLRNVGRSHRKGFKSEYFWKMEAPFLAAVKLRME-SIYKITIHFIL----------------------------------

>Itch_mite_GbXL

--------------------------------------------PLTVRQKFNLSKSWKGI-EMEM---TGVLMFVKLFEETPEILNLF-T-DSQM-KSMELAEHAT-KVMTNLDE-MINSL--FFRHLHSLGKYHRRGFHKDNFLKLEKPFIEAVKLRMA-NIYNIIIKLIL----------------------------------

>Acorn_worm_Gb15

--------------------------------------------PLTARQKFQITKSWKGI-NMEN---TGKSMFMRLFQSNIELKNMF-T-EDMR-ESQQLENHAS-LVMYTIDE-AIASI--VVELLGKIGRTHTR-FNPQLFWRIEQPFLSAVKLRIE-EIYKITFRFIV----------------------------------

>Acorn_worm_Gb6

--------------------------------------------PLTARQKFSIQKSWKAI-NMEG---VGMDIFIRLFKAHPEYQDLF-P-EKLR-NSINFETHVG-IFMNVIDE-CIDSL--VINLLTKKGRKHAN-VKPEFISDIEEPFLASVKLRIE-EIYKLTIKFIL----------------------------------

>Amphioxus_Gb12

--------------------------------------------KLDAKEKFFLEKSWKTV-NEDV---AAMAMFINLFRSSPEIKDKW-P-DEMR-DSPYLQKLSV-RILGAMDH-VIDSL--LIPALEKLGQMHADIILPEDLWKLEGPFLRAVGLRYQ-DIYQKFIIFVL----------------------------------

>Amphioxus_Gb3

--------------------------------------------PLDARQKFHLEKSWKSV-NIDR---AGMFMFLRLFRDCPEMIEKY-P-EELR-NSQFLQEHSQ-RVLDAFDH-TIDSL--VIQLLKKIGQMHAD-LKPDDMWKLEQPFLAAVALRFQ-EIYSKLITFII----------------------------------

>Amphioxus_Gb6

--------------------------------------------PLDPWQKFYLEKSWKTV-NIDK---AGMIMFVKLLRDYPEIQQKW-P-EEVT-KSVYLMNLAT-RIFDTLDH-AIDSL--LIPLLKRLGQMHADIMDPEDIWKMERPFLESVRLRYE-EIYSKFIIFII----------------------------------

>Amphioxus_Gb13

--------------------------------------------PLDAWQRFYLQKSWKTV-KSDQ---AARTVFLRMLQDNPGLRQKW-P-EEIP-TSPYIKFLGE-RIFDCLDY-IIDNL--VISELTKLGRQHSDVMTPEDVWAIEAAFLAGVQLRYE-EIYSRFIVFVI----------------------------------

>Amphioxus_Gb14

--------------------------------------------PLTQKQKFLLLKSWKGV-QISQ---CGKTMLIRLFKDDPQLMAVF-Q-DVLY-QDAILDAHAA-TVMEALHE-AITHL--VMKVLHDVGKMHQR-VDPSVFLKVEKPFLTAVSLRME-EIYTITIKFIL----------------------------------

>Muga_silkmoth_HbL

--------------------------------------------GLTRREIYAVQKSWAPV-NTVA---NGTEFFRRLFQTSPETKEFF-K-EEYL-QSPQFRAHAI-NLMTSLNL-AVNNL--VAAMMNKLGESHKR-IKKKHFGELKQVIVKIFIL-TL-TAWDKTVTFWY----------------------------------

>Eri_silkmoth_HbL

--------------------------------------------GLTRREIYAVQKSWAPV-NSIP---NGAELLRRLFQTFPETKEFF-K-EEYI-QNPQFRAHVI-NLMTSLNL-AVNNL--VAAMMNKLGESHKR-IKERHFGDLKQVIVTMFIL-TL-TAWDKTVTFWY----------------------------------

>Silkmoth_HbL

--------------------------------------------GLTRREIHAVQKSWAPV-NSFA---TGSELLRRLFNTYPDTKEYF-K-EEYS-QNPQFKAHVI-NLMTSLNL-AVNNL--VAAMMTKLGESHRR-IKEKNFHELKEVIVKLFIL-TL-SAWGKTVEFWY----------------------------------

>Tobacco_budworm_HbL

--------------------------------------------GLTRREISLVQKSWVPV-DAIN---TGAELLKRFFIAFPESKDFF-K-DQYL-QNPQFKAHVI-NLMTSLNL-AVENL--VAAMMNKLGESHGR-IQEKNFNQLKEVIVKMFIL-TL-GAWGKTVEFWY----------------------------------

>Armyworm_HbL1

--------------------------------------------GLTRREVNLIQKSWAPV-DKAA---NGAELLRRFFTAYPAAKEFF-K-EQYL-ENPQFKAHVI-NLMTALNL-AVENL--VAAMMNKLGESHGR-IKEQNFQDLKQVIVKMFIL-TL-GAWGKAVDFWY----------------------------------

>Cotton_leafworm_HbL

--------------------------------------------GLTRREVYLVQKSWAPV-DKVN---NGAELLRRFFTAFPASKEFF-K-DQYX-TNPQFKAHVI-NLMTSLNL-AVENM--VAAMMNKLGESHGR-IQEKNFLELKQVIVKMFIL-TL-GAWGKTVDFWY----------------------------------

>Squinting_Bush_Brown_HbL

--------------------------------------------GLTRREVYAVQQSWAPV-NSVA---NGTELLKRLFRAYSETKEFF-K-HEYA-DNPQFKAHVI-NLMSSLNL-AVNNL--VAAMMSKLGESHGK-IQREHFYDLKDVLVKMFIL-TL-AAWGKAVEFWY----------------------------------

>Monarch_butterfly_HbL3

--------------------------------------------GLTKREVYAIQQSWAPV-NSVA---NGTELLKRLFRAYPETKEFF-K-DEFI-GNPQFRAHVI-NLMSSLNL-AVGNL--VSAMMNKLGESHGR-IQEKHFHDLKDVIVKMFIL-TL-NAWGKAVDFWY----------------------------------

>Postman_butterfly_HbL2

--------------------------------------------GLSRREVYAVQQSWAPV-SSVT---NGTELLRRLFQAYPETKEFF-K-EEYS-QNPQFKAHVI-NLMGSIDL-AVTNL--VAAMMNKLGESHGR-IQREHFYGLKDVIVKMFIL-TL-TAWDKTVDFWY----------------------------------

>Monarch_butterfly_HbL1

--------------------------------------------GLSRRDVFAVQKSWAIV-NPLA---NGSELLKRYFRAHPESKEFF-R-NEFD-DNHQFKAHVM-SLMSSLNL-AITNL--VVAMMNKLGESHGR-IDEQNFHNLKGIIVKMFIL-NL-ASWGKAVDFLY----------------------------------

>Postman_butterfly_HbL1

--------------------------------------------GLSKRDIYVVQKTWAVA-DSVG---TGNELLKRYFRAYPETKDFF-R-EKFT-ENFQFKAHVI-NLMSALDL-AVKNL--VAAMMAKLGESHGR-IQEKQFNELTIVIVQLFKL-TL-AAWGRVVGFWY----------------------------------

>Monarch_butterfly_HbL2

--------------------------------------------GLSHRDIYTVQKTWAVV-NAAE---NGIEIFKRLFHANPETKNFF-I-EELD-KSHQFRAHVI-NLMSSLNL-AITNL--TAALMNKLGESHGK-IREEHLLSLKDVMLEMLNL-AL-VSWNKTIDFIY----------------------------------

>Armyworm_HbL2

--------------------------------------------GMSLRDVHNVQKSWAVI-NSNG---NGFLMFFRLFEAEPETKLFF-K-AEMS-ANVSFRAHII-NIMSSFDT-SIQNL--VVAWMQKLGDSHRR-IEKRHFHVFKDVLVTILQL-VV-ASWDRYVEFIY----------------------------------

>Honey_bee_HbL

--------------------------------------------GLTERQKKLVQNTWAVV-DEVA---SGIAVMTAFFKKYPEYQRYF-T-NELP-ANKRFQAHCA-GVITALNN-VIDFL--MEASLIGLVERHKK-QTKEEFQNLKEVMLEVLRLQVA-EAWNKTLDMMF----------------------------------

>Bumblebee_ HbL1

--------------------------------------------GLTEKQKKLVQNTWAVI-DEVA---SGIAVMTTFFKTYPEYQRYF-S-DELP-ANKRFQAHCV-SVITALNS-VIDSL--MEASLISLGERHKR-QTKEEFENLKGVVLKVLSLQVA-EAWSKTLDGVF----------------------------------

>Nasonia_wasp_HbL

--------------------------------------------GLTGRQKKLVQNMWAIV-EPIP---NGVAIMLAYFKKYPEYQKVF-T-EELS-ANKKFQAHCL-NIVTALNN-LIDSI--LEANLVAIGERHHR-QTKEQFLHLKEVIAEVLRLKTA-EAWNKTIDAAY----------------------------------

>Carpenter_ant_HbL

--------------------------------------------GMTEKQKRLVQNTWAIA-DEVS---AGVAIMIALFKQYPEYQKQF-K-DELP-KNKRFQAHCV-NIISAISK-LIEQM--MQATLINLIEKHKN-QTQEQFENLRQLLAKLFPFQAE-EAWKKLLDLMY----------------------------------

>Red_fire_ant_HbL

--------------------------------------------GLTEKQKRLVQNTWAIV-DEVS---IGVALVLAYFKQYPEAQKEF-K-DELS-KNKRFQAHCA-NIVATIGK-VIEQM--MEASVINFTEKHKN-QTQKQFENLKQMMLDVFPFQVQ-EAWKKMLGLIY----------------------------------

>Bumblebee_ HbL2

--------------------------------------------GLTNKEKRIIRETWGVL-NSVK---VGVDIMISYFKRFPQHHRAF-P-DDLL-DNKKFHAHCQ-GIMSTLND-AIDAL--MNAILHTTGKRHGR-QGRQEFIDLKGVVLDAMRFKVE-VAWDKAIDVLF----------------------------------

>German_cockroach_HbL1

--------------------------------------------GLTPRERQIVKDTWALA-NSKS---VGVELFIQLFTTYPHHQQKF-P-SEMK-GNKKLEAHAT-NVMYSLAT-LVDNL--LIELCSKIGENHLR-VEQQAFLDVKTVLMKLLKLSGE-EAWNKTLDLAN----------------------------------

>Subterranean_termite_HbL

--------------------------------------------GLTPRERQIVVDTWGVV-NAKE---AGVEMFTRLFEAHPQYQKLF-P-SVLR-TSKKLAAHAT-NVMYSLTS-VIDNL--LKELLIKLGKNHGR-VYEKQFHDLELVLMELLKLQGE-VAWKKTIDIVY----------------------------------

>Termite_HbL1

--------------------------------------------GLTPRERQAVVDTWAIM-DAKR---AGVELFIQLFEAHPEYQKLF-R-QELE-KSAKLSAHAT-NVMYSLTS-VIDNL--LTELLIKLGQNHDR-VSEKEFNDLKVVLMKLLKLKAE-AAWSKTIDVAY----------------------------------

>German_cockroach_HbL2

--------------------------------------------GLTPREKNAIRRNWELV-DIKQ---NGIDLLMLFFEENPSYQQFF-N-KELP-KNPKFHAHCT-SVMYALSS-VVDNL--LVEMLSKLGENHHR-ISRQEFINLKAVVLKLLKLKDE-AAWNKTLDVAY----------------------------------

>Jumping_bristletail_HbL

--------------------------------------------GLTPREKRAVTESWAII-DLKG---NGMIFLLMFFDDYPDYQKFF-R-SQLP-DDKRLMAHVT-SVMYALSN-IVDNL--LVETLKKLGENHGR-ITLQEFENLKAVVIKFLKVKAE-AAWVKTLDSAV----------------------------------

>Scarce_chaser_HbL3

--------------------------------------------GLTPRQKRAVAVTWDIV-DLKG---NGVELLHRFFTKHPQYQKNF-K-DELP-NSKKFQAHAN-SVVYAVTS-IVDNL--LVEMLRKLGQNHGQ-IPEQAFLDLKAVLMKMLKLHEE-ESWDKTMDTAF----------------------------------

>Cricket_HbL

--------------------------------------------GLTPRQKKFVSDTWQLV-DIKG---NGIELFIRFFEMRPEGQNRF-S-NELR-HSKRLQAHTN-SVMYALDG-VVMPL--MHEMLLKIGMNHGR-ITEEEFHELKIVLMNLLKLHGE-EAWSKTIDVFY----------------------------------

>Migratory_locust_HbL1

--------------------------------------------GLTPREKHFVVTTWAAV-DITS---NGVQLFLRFFDKLPAAQKRF-S-DELA-ASKRLKAHAN-SVMYSIDS-IVCNL--LEEMLLKIGNNHGR-IPEDEFMVLKDVLMQLLRLHGE-QAWSKAIDVMY----------------------------------

>Velvet_spider_HbL

--------------------------------------------GLTPRQKDIVRNTWKSI-DTRN---NGIKLFLKFFEAYPEYQLLF-K-SDLP-RNGRLLGHVT-SVMYALNS-VVDNL--LIEILQKTGISHRP-VNRQHFNNLKVVLIKLLVLNSAVEAWEKTLDVAN----------------------------------

>Scarce_chaser_HbL2

--------------------------------------------GLTPAQIQAVRSTFDVL-DPKD---FGVDLFLSLFDAHPNYQKLF-R-SELP-GNKRFLAHAS-TVVYSLMS-VIDNL--LVEMLVRIGQNHGR-VQPESFEHLKSVIMGLLKLRAE-ESWSKTLDVAN----------------------------------

>Blue_winged_olives_ HbL

--------------------------------------------GLTLRQKGLVRSTWALV-NIRT---VSVDVLITFFETFPQYYPLF-T-TDLR-SNKKFTAHAT-TVFHALAS-LVDAL--LVELCEKVGKAHIS-VPPGAFDDLKVTALKVLSLKAA-EAWDKTLTAAF----------------------------------

>Mayfly_HbL1

--------------------------------------------GLTPRQKRAVVDTWALV-DLKA---TGIAVLIALFEAHPEHQRLF-S-SELR-GSKRFAAHAS-SVMHAIAS-LVDTL--LVELLTKIGVNHAK-VPPHAFSDLQAVILKLFQLRAA-EAWDKTLTVAN----------------------------------

>Orchid_beetle_HbL

--------------------------------------------GLTPREKSLVVNSWAIV-DMIG---NGTELFILFFTKFPKYLTYF---DQLR-ENKKVHAHAV-NVMYALSS-IVDNL--LVNLLSKTGEAHGR-IPEQSFGDLKVTVLELLRLKGI-DAWNKTLEVAI----------------------------------

>Black-legged_tick_HbL1

--------------------------------------------EMTSQEKHVVRDTWAIF-EVQT---SGVAIFVVLFFKHPAYQKLF-V-AELP-QNPRAIAHAL-TVAYAITS-IIDTL--SAELVRKVATNHVRTISGAQFEHMGQAVVEVLALAAV-GSWQKFFAFVV----------------------------------

>Black-legged_tick_HbL3

--------------------------------------------GLTPREKGLVRDTWALV-DVKA---NAIAIFLTLFQRHPEYQKLF-S-EALS-TNPRLGAHAM-SVAYAITS-LVDSL--LVELVRKVAVSHTR-VSVTHFENLTVVIVDTLKLKAV-AAWEKTLRLVV----------------------------------

>Bark_scorpion_HbL1

--------------------------------------------GLTKKEKDGIKYTWDIV-DIPK---NGVALFIMFFKTNPDHQKVF-T-SELP-KNKKLMAHAS-SVLYSISS-LVDSL--LKEMVIKIAHNHLR-VDDKHFSSLGESIISFMELKK--EAWQKFYSVVV----------------------------------

>Mountain_pine_beetle_HbL1

--------------------------------------------GLTSKEKYLVRTSWAKI-NPAD---SGVALLCLLFERHPEYVQLF---SEFK-TNVRFRAHAN-SVVYALSS-IVDAL--LVQILTKTGSSHVP-VTADAFIHLKEVTIELFS--EV-AAWKKTFEVAF----------------------------------

>Red_flour_beetle_HbL1

--------------------------------------------GLTSRDRYVIQTSWAPV-DLTG---NGVALLLLYFEKFPATKNYF---EKLK-TDKKFHAHCN-SVMVTLDS-LIANL--IVSLLEKLGKNHKR-IKDDAYDQLKETVIELFS--EL-ETWDKLLKVAF----------------------------------

>Red_flour_beetle_HbL2

--------------------------------------------TLTSREVFLVQSSWDPI-DLTG---YGVQLLLFLFKKYPEEQQNF---EELG-ASKKFHAHCS-NVMYAVDS-IIDSL--LVNILEKIGRNHHR-VKPISFWHVKETMLEFFK--TL-KAWDKALQVAF----------------------------------

>Whipscorpion_HbL

--------------------------------------------GITLREKTLIRESWDLI-DLKG---NGIAFFIKLFDEFPEYQKLF-K-EELP-TNKRMIAHAT-TVMYGFAS-FVDSL--LEGLIEKIVTNHAR-ITKENFKNIGIVLENYLKLKGI-QAWRKLCDVVQ----------------------------------

>Black-legged_tick_HbL2

--------------------------------------------GLTTSDKCAIKDTWTMF-ETRT---NALSLFVALFSRYPEYQKMF-P-KDMM-QCPSLTAHAL-TVIYALAS-IIESI--MVELIKKNIRNHVR-VTPEHFVNINNLLIEVMQLRVI-VSWKKFFAMHD----------------------------------

>Vietnamese_centipede_HbL

--------------------------------------------GITARQKLVVRENYGRG-NLKS---NGVEFFVALFTKHESLKKYF-V-EALP-SSKKLQAHST-TVMMAISG-LVDNL--LKELLLKIGENHSR-VSIEEFKKLAVVFVDFLELGAR-KAWEEVFRVMN----------------------------------

>European_centipede_HbL

--------------------------------------------GLTLRQKKVVTEIWDLV-DIKQ---NGIDFFIEFFKAFPLNLNNF-K-DQLR-KSKKLEAHAT-NVMYAIST-VVDNL--LTELLSTIGRNHIK-ITPVQFDQVGITFIKFLELRCR-NAWEVTFKVMN----------------------------------

>Scorpion_HbL

--------------------------------------------GLSLRDRKEITDSWHIL-DIKS---AGTQFFIKLFIEHPTLQKLF-P-SELQ-TNKKLIAHGT-IVMYSISS-MVDNL--FKVLAANVAQSHYN-VTYEHFSKLGPVMLSLLELSTE-VAWQKFLSVLV----------------------------------

>Western_corn_rootworm_HbL

--------------------------------------------GLTSRDIYLIKNSWNKV-QPTE---NGIKFFMRLFEIAPKHKLTF---EDLP-RNKKFHAHVN-SVMYSISS-IVNSL--VAAIIDKIGRNHAR-VDLQALKDVKRALLDIFA--EL-AAWNKMLDYFA----------------------------------

>Remipedia_Speleonectes_HbL

--------------------------------------------GLSSAQGKAVADTWAVV-DLKQ---HGTKILIELFKAHPQYQAKF-K-EDLP-RNKKLHAHAC-NIMFTIDN-MICNL--LTEVVMKVGRSHKP-LTMSDLQNLAKVAQDYLALQGK-EGWAKVFAVVT----------------------------------

>Green_crab_HbL

--------------------------------------------GLTLRHRTAIYRTWDLV-NPKL---HGINLFLTMFQEEPVLQTRF-K-EELK-NSKRLAAHGT-TVVMAITA-MVDNL--LVELLKNTGANHRD-VPKGDFELLAPVLVRFLKLAAE-EAWTQAMKVIN----------------------------------

>Water_flea_HbH

--------------------------------------------GLSQRERDYIQQSWHHV-DLKA---AGLGFFQAFFKAHPDYQLKF-K-DQLA-DNKSFLVHAM-SVMNAVTM-VVDSL--LVNELKNLGKNHGR-IKTENFRNLTVVLVAFLELQDVKQSWIKALDVVV----------------------------------

>Flower_thrips_HbL

--------------------------------------------GLSPRDKHLVRTTWAIV-DASS---NGLYLFQLLFTKHTDVRDMF-P-AEYR-DDPRMRAHAN-AVMYALTS-YIDQL--LDAMVRKLADSHLK-VTPEHFKALGAVVMQALQLSAV-TAWTRTYGLVL----------------------------------

>Itch_mite_HbL1

--------------------------------------------SLTNRDKEIIVSTWSLI-DSDQ---AGIHLFKRFFEANPDYVKYF---EKIL-VDPRLKWHAS-RVMAALST-IVDNL--FEDSLQKVLSSHLN-IQLYHFENLKKALVCLFMLDTI-EAWSKAYDVIL----------------------------------

>Water_flea_HbA

--------------------------------------------VLNSVNVAAVQSTWAVI-DINT---FAPQFYVALLTAHPEYQAMF-P-GQLL-NNAALITLSV-NVVTKLSE-IIDSL--LNGKLVDLANQHKQ-TTRAHFDNMATVLLGFLALAAK-QAWTSTMQGIN----------------------------------

>Water_flea_HbD

--------------------------------------------VLKSVNVAAVQSTWAIV-DLNT---HAPKFYVALLTAHPEYQPMF-P-GELL-NNAALKTLSV-NVLSKLSE-LIDGM--LNAQLVELAKQHKN-TTRTHFDNLAKVLVDFLALAAK-QAWTATMQGIN----------------------------------

>Mayfly_HbL2

--------------------------------------------GMTPRDRRVVSRTFAVC-KVRE---VAMDIFVTLFTKHPEHQKLF-P-EELR-TSKRLTAHAS-TAAHGLAA-IVECI--LTAMLNKLGDNHKR-VSPSAFPDFKDVLMEVLKLLAE-LSWDRAMNFVN----------------------------------

>Mountain_pine_beetle_HbL2

--------------------------------------------GLTSRYISVLKTTWKRITGTLE---IGTAIFTNLFEKHPEYQQLF---EELK-TSNKFRAHCI-SVMYALTC-IVENV--LEQLLIKQSTSHVL-VPDQAYWDIKTVILSIVA--EV-FVWEKFLKFAF----------------------------------

>Mediterranean_fruit_fly_Glob1

--------------------------------------------ALNAEDIAEIKKTWAIP-TPTD---SGAAILIRFFTKYPSNLEKF-P-AELN-NSARFRAHCG-RIIKTFDQ-SISQLE-IQDIWQGIASSHVQNIPKPSYFELREAIVEVLSC-QA-EAWNKLLDIVY----------------------------------

>Fruit_fly_Glob1

---------------------------------------------MNSDEVQLIKKTWEIP-TPTD---SGAAILTQFFNRFPSNLEKF-P-EELS-GNARFRAHAG-RIIRVFDE-SIQVLQ-LDEIWTKIAVSHIP-VSKESYNQLKGVILDVLTC-QA-ATWAKLVDHVY----------------------------------

>Tsetse_fly_Glob1A

---------------------------------------------MNSDEVYEIKRTWEIP-TPTE---SGVAILIRFFTKYPSNLQKF-S-DELK-NNPRFKAHAN-RIMKVFDD-SIKTLD-LEEIWTKIAQSHFN-IEKQSFNELKEVILEVLVC-QT-EIWLKLLDFVY----------------------------------

>Tsetse_fly_Glob1B

---------------------------------------------MNSDEVSEIKKTWEIP-SPTE---SGVAILIQFFTKYPSNLEKF-S-DELK-ESPRFKAHAN-RVIKVFDD-SVQALD-LEEIWVKVTQSHFN-IEKHSFNELKEVILEVLTC-QI-EIWIKLMDFIY----------------------------------

>Australian_sheep_blowfly_Glob1

---------------------------------------------MNCDEVYEIKKTWEIP-TPTE---SGVAILLKFFTKYPSNLEKF-Y-DELK-NNARFKAHAV-RIIKVFDE-SIQMLH-LEEMWSKVAVSHFN-IEKQSFNELKEVILEVLTC-QT-AAWIKLMDIVY----------------------------------

>House_fly_Glob1

---------------------------------------------MNTDEVLEIKRTWDIP-NPTE---SGSAILMLFFKRYPSNLQKF-S-DELS-TNARFRAHAS-RIIKVFDE-SIQMLH-LEETWSKIATSHFN-IEKKSFNELKEVILEVLTC-QI-QAWTKLMDTVY----------------------------------

>Humpbacked_fly_Glob1a

--------------------------------------------ELSDFEVIEIKNTWKIP-DPSG---SGQAILLKFFERYPHNKLKF-Q-DQLK-TCPKFKAHAS-RIVRTFNE-AINVLT-LHEIFSKVAISHHK-ISKASYNELKEVILEIVVC-QK-CAWEKLMETIY----------------------------------

>Humpbacked_fly_Glob1b

--------------------------------------------ELSDNDILEIKATWKIP-NPSE---SGEAILLKFFERYPSNLEKF-K-DELK-VCPRFKAHAS-KIIRTFDE-AINILT-LQEIWSKVALSHHK-ISKSSYNELKEIIIEILVC-QK-KAWEKLLICVY----------------------------------

>Humpbacked_fly_Glob1c

--------------------------------------------ELSEAEILEIQNTWKIP-DPLA---SGQAVYLKLFKRYPSNQLKF-I-EDLK-DSPRFKFQAL-RLMRTFDK-AINALT-LHEIFAKVAVSHHK-ITKVAHDQLKEVLIEILIC-QK-TAFEKLMEATF----------------------------------

>Humpbacked_fly_Glob1d

--------------------------------------------DISEKEVLEIKRTWKIP-DPLT---SGETMLLKLFERYPANQKKF-Q-KDLK-DSPKFKFHSV-RIMKAFDE-AIQSLT-LHEIFEKVAVSHHK-ISKESHNQLKEVIIETLVC-QK-GAWEKVMESIF----------------------------------

>Bot_fly_Glob1

---------------------------------------------MNSEEVNDIKRTWEVV-KMTE---AGVEMLKRYFKKYPHNLNHF-P-DDLP-ENARFKTHGT-RILRQVDE-GVKALV-FDDVWKKLAQTHHE-VERRSYNELKDIIIEVVCV-QV-HAYHKFFDRAY----------------------------------

>Lutzomyia_sandfly_Glob1

--------------------------------------------GLTPEQIEIVKSTWQLV-APED---AGEAILMRFFEKFPDNQKYF---ENLK-GSVMFRSHAG-RVVAVFQK-AVDAFT-LVEIWTEIARTHFR-IKQKSFDELKEVVLEILTC-QQ-TAWAVTLDTIF----------------------------------

>Phlebotomus_sandfly_Glob1

--------------------------------------------SLTPEQIEIVKSTWVTV-APED---SGEAILLRFFEKYPHNQKYF---ENLK-GSAMFRSHAG-RVIAVFQK-SVDAFT-LVEIWTEIAKTHFK-ITQTSFNELKEVILEVLTC-QQ-TAWAVTLDTIF----------------------------------

>Hessian_fly_Glob1

--------------------------------------------SLTPHQIALIQSTWSIP-VPID---SGEAILLAYFEKYPQNQQKF-N-LSLK-GTPGFRTHAG-RIITVLDE-AISNLK-LERIWNQIGESHNR-ISRQSFNELRDILVHTLIC-GK-LAWNTLMDIIY----------------------------------

>Pea_aphid_HbL

--------------------------------------------SLSPLQISQLKDSWSVL-DPSQ---LASALVIRLFKENPEYQSLF-K-DELA-SNPQFMSHAS-KVGAALAS-TIDHL--LEKLLTNLGIKHKK-LSAKHFQVIGDVLIAMITISLL-DLWKSSLTSVL----------------------------------

>Peach_aphid_HbL

--------------------------------------------SLSPLQISQLKDSWSVL-DPSQ---LASALVIRLFKENPEYQSLF-K-DELA-SNPQFMSHAS-KVGAALGL-TIDHL--LEKILTNLGIKHKK-LTAKHFQVIGDVLVAMISISLL-DLWKSSLTSVL----------------------------------

>Asian_citrus_psyllid_HbL

--------------------------------------------NISDDRLQAVVLSLDII-DIND---FGTKVFKTLFKEHPEYQSQF-P-DKLD-ANKSFTHHVN-AVVLAIAN-SVVNL--VLPELEKLGTSHQR-IRPEQFEVVTNIILKVLKVPVL-KTWQEILTILA----------------------------------

>Hibiscus_mealybug_HbL

--------------------------------------------IFADEVIKDVKTTWATI-DLQQ---VGYEIFNRLFNAFPTYQQLF-R-GELQ-SNKDYSKHAL-AVAKALNA-SIENL--LVSILTTVGKNHVK-VTPEHYSNAQKIILEVIGLEIL-SSWNEVLAVAV----------------------------------

>Amphioxus_Gb1

--------------------------------------------GLTPTQSRLVKESWKMF-KKRE---NGFVIFRVLFTDYPVTRKLF-K-GQLE-SSITLRAHVT-RFMHSFDT-YMESL--LKQLLYDTGKSHLI-IKPEYFDVLETVLMKSLRFKLE-EAWQTAYSHLK----------------------------------

>Amphioxus_Gb2

--------------------------------------------GLTPTQVRLLQQTWKVI-HKKQ---NGFLIFKILFTDYPMTKKLF-K-EQYE-KTTSMRAHVT-RFINSFDS-FMECL--LKSLLYDTGKAHLR-TKPEHFDDLEVVMMKSLKLKVE-EAWRTAFAFFV----------------------------------

>Amphioxus_Gb5

--------------------------------------------GLTANQIRHIRETWQVV-NKRA---NGFAIFRILFTDYPFTKKLF-R-EQFE-KNIALRAHIT-RFLHSFDT-YVSNL--LQQLLYDTGKSHLR-VKPEYFDALGNVLMKGLTLDVQ-GAWGTAWGFFV----------------------------------

>Amphioxus_Gb9

--------------------------------------------GLTANQIQLIRDTWQIV-NKRE---NCFAIFRILFTDHPSTKSLF-R-GEFE-KNVAARAHMV-RFMHSFAT-FMDTL--LRQLLYDLGKNHAK-VGPELFDALGPILMKALPLKVK-TAWLTAYTFMS----------------------------------

>Acorn_worm_Gb1

--------------------------------------------TLTSDEVAAIKSSWSAV-KKKE---SGVTLFVKLFTENPSFKSQF-G-GDMK-TLPALENHGV-KVMDRINE-WMGNL--LVKQLKHLGTTHIA-VTEDNFNAMDSVLMYTLQGAAK-AAWQKAWGVMK----------------------------------

>Acorn_worm_Gb3

--------------------------------------------ALSAGEIKLVTDSWTAV-NKKA---NGVALFVRLFSENPGFQSQF-R-AAIE-KTPALGDHAV-KVMDTINS-WIGSL--MVAKLTALGTSHIA-VTPANFDAMGPVLLWMLQAAAK-DAWAKGWDLMK----------------------------------

>Acorn_worm_Gb4

--------------------------------------------SLSAGEIKLVKDSWAPV-NKKE---SGIALFVRLFSENPGFQSQF-R-AAIE-KTPALADHGV-KVMDTVNS-WVGSL--LVKQLTALGTSHIA-VTPANFDAMGPVLLWTLQAAAK-DAWAKGWDLMK----------------------------------

>Acorn_worm_Gb2

--------------------------------------------ILTPKEVKAISESWKVV-KKKE---NGVALFIRLFQSVPGSKSLF-K-EKLR-NHPRLKAHGF-RVMSSVNS-LIESL--LVQLLKDLGSSHSK-VTSSHFDALGPVIIWLLQNSVK-NAWLKGWGVMK----------------------------------

>Acorn_worm_Gb5

--------------------------------------------TLTPSEAIAIQSTWLFV-DKEE---NGVELFVKLFTEHPDYQALF-G-ENIK-NVPFLRVHAS-HVLIYLNT-MLESL--LVELLKTLGYTHVG-LTPEHFDALGPILISLLQGSAE-KAWLKGWGVMK----------------------------------

>Amphioxus_Gb8

--------------------------------------------SLTEGEKATIRRTWAVA-DMMG---NGANILLKMFEINPDTKKVF-A-NQLQ-STPRFRAHVT-RVMASIGT-VVNSL--LLDLFKDIGKKHYP-VPTEYFDVIAGAILCMLQLGVD-SAWTKLYGSLG----------------------------------

>Amphioxus_Gb4

--------------------------------------------PLTQEQVHGIKETWAIL-DPVE---RGVDLFMKIFEEDPDLKKLF---ELSR-EDQRMRSHGE-RVMEAVGG-AVDSL--VVPVLTELGALHHK-VQPSYFDTVGAALIYILELKIR-QGWVLVYGIVG----------------------------------

>Human_Ngb

---------------------------------------------MERPEPELIRQSWRAV-SPLE---HGTVLFARLFALEPDLLPLFNR-EDCL-SSPEFLDHIR-KVMLVIDA-AVTNV--LEEYLASLGRKHRA-VKLSSFSTVGESLLYMLELATR-AAWSQLYGAVV----------------------------------

>Zebrafish_Ngb

--------------------------------------------KLSEKDKGLIRDSWESL-NKVP---HGIVLFTRLFELDPALLTLF-S-PECL-SSPEFLEHVT-KVMLVIDA-AVSHL--LEDFLLNLGRKHQA-VNTQSFALVGESLLYMLQLALR-QAWLTMYSIVV----------------------------------

>Cotton_aphid_GbX

--------------------------------------------PLTMRQKELLTEMWKLL-DIAK---VGVITFVSLFETHPDVQQSF-M-EDLK-HSRQLRDHAL-RVMAFVQK-AVARL--LETLLRDLGKKHYH-AKQKYVDLIGPQFIMAIQLRMH-SAWTALFLNMA----------------------------------

>Pea_aphid_GbX

--------------------------------------------PLTMRQKELLTEMWKLL-DIAK---VGVITFVSLFETHPDVQQSF-M-EDLK-HSRQLRDHAL-RVMAFVQK-AVARL--LETLLRDLGKKHYH-AKQKYVDLIGPQFIMAIQLRMH-SAWTALFLNMA----------------------------------

>Silverleaf_whitefly_GbX

--------------------------------------------PLTDAQKSMLVDTWKAL-DIAK---VGVITFISLFETHPDVQQVF-M-EDLK-HSKQLRAHAL-RVMAFVQK-AIARI--LDTLLKDLGRKHYS-AKVKYVDLIGPQFIQAIQLRLH-QAWACLFQFMA----------------------------------

>Asian_citrus_psyllid_GbX

--------------------------------------------PLTEDEKKLLIETWKIL-DIAK---VGVITFISLFETHPDVQQSF-M-EDLK-HSKQLRAHAL-RVMAFVQK-AIARL--LDTLLRDLGKKHYT-AKAKYVDLIGPQFISAIQLRLN-NAWIHLFGYMA----------------------------------

>Hackberry_petiole_gall_psyllid_GbX

--------------------------------------------PLTDEEKKMLSETWKVL-DIAK---VGVITFISLFETHPDVQQVF-M-EDLK-HSKQLRAHAL-RVMAFVQK-AIARL--LDTLLRDLGKKHFT-AKAKYVDLIGPQFISAIQLRLH-NAWVHLFAYMA----------------------------------

>Bed_bug_GbX

--------------------------------------------ALTKEEMDHLTRTWKLL-DIAK---VGVITFISLFETHPDVQQVF-M-EDLK-HSKQLRAHAL-RVMAFVQK-AIARL--LEQLLKELGKKHHG-AKVQYVDLVGPQFIQAIQLEVA-DAWKLLFAHVG----------------------------------

>Brown_marmorated_stink_bug_GbX

--------------------------------------------PLSDLQKAELTRTWKLL-DIAK---VGVITFISLFETHPDVQQVF-M-EDLK-HSKQLRAHAL-RVMAFVQK-CISRL--LEQLLRELGKKHHS-AKAKYVDLVGPQFIQAIQLEVS-EAWILLFAHIN----------------------------------

>Milkweed_bug_GbX

--------------------------------------------PLSDQQRAHLTRTWKLL-DIAK---VGVITFISLFETHPDVQQVF-M-EDLK-HSKQLRAHAL-RVMAFVQK-CISRL--LEQLLRELGKKHYG-AKAKYVDLVGPQFIQAIQLEVS-AAWVLLFANIS----------------------------------

>German_cockroach_GbX

--------------------------------------------PLSERQKELLVETWKEL-NIAQ---VGVITFISLFETHPDVQQVF-M-EELK-HSKQLRAHAL-RVMAFVQK-AVARL--LEKLLQDLGKKHYA-AKQKYVDLIGPQFIQAIQLQLN-TAWVQLFQYMA----------------------------------

>Termite_GbX

--------------------------------------------ELTERQKELLEETWKEL-NIAK---VGVITFISLFETHPDVQQVF-M-EDLK-HSKQLRAHAL-RVMAFVQK-AVARL--LETILQELGKKHYT-AKQKYVDLIGPQFIQAIQLRLQ-EAWIHLFKYMA----------------------------------

>Scarce_chaser_GbXa

--------------------------------------------PLTEEQMGLLQENWKEL-NIAK---VGVITFISLFETHPDVQEVF-L-EELR-HSKQLKAHAL-RVMAFVQK-AVARL--LDVLLRELGKKHYT-AKQEYIDYIAPQFIQAIKMKLE-GAWTALFNYMG--------------------TQQSFA-----L--

>Scarce_chaser_GbXb

--------------------------------------------PLTEKQMAMLQENWKEL-NIAK---VGVITFISLFETHPDVQDVF-I-DELR-HSKQLKAHAL-RVMAFVQK-AVARL--LDLLLRELGKKHYS-AKQQYIDYIAPQFIQAIKLRLQ-DAWIALFNYMG---------------APVNPTAPPTPPPPPVI--

>Scarce_chaser_GbXc

--------------------------------------------KLTERQKDLLTETWKEL-NIAR---VGVIMYIGLFETHPDVQEAF-M-TELK-HSKQLRSHAL-RVMGFVQK-AVARL--LDVLLRDLGKKHFS-AKPEYVDLIGPQFIQAIKLKIH-EAWTTLFEYMA--------------------NRPTHN--------

>Body_louse_GbX

-------------------------------KPSNNQKQRR---ELTTREKELLIETWKEL-NIAK---VGVITFVSLFETHPDVQESF-M-EDLK-HSKQLRAHAL-RVMAFVQK-AVARL--LETLLKELGRKHVG-AKQKYVELVGPQFILAIKLQLD-DAWTHLFKIIE----------------------------------

>Mayfly_GbX

--------------------------------------------PLTEEQCVLLAETWKEL-NIAK---VGVITFISLFETHPDVQQTF-M-EELK-QSKQLRAHAL-RVMAFVQK-AVARL--LEALLRDLGQKHYF-AKPEYVELIGPQFIQAIRLRLQ-DTWLQLFRYIS----------------------------------

>Bark_scorpion_GbX1

--------------------------------------------FVTYQQKVALVQTWNVL-NLSR---VGVIAFMRLFETHPDVQEIF-I-ESLR-NSKELRAHAL-RVMSFVQK-VVARL--LEMLLGELGKSHLN-AKAEYIEKIGPQFIYAVKLHVE-NAWLQLFRYIT----------------------------------

>Bark_scorpion_GbX2

--------------------------------------------DLTNQQKALVLNTWKLL-NISR---VGVITFMSLFETHPDVQEVF-M-EELS-RSTDLRAHAL-RVMGFVQK-IVARL--AEQLLGDLGKKHVM-AKPDYVDLIGPQFVYAVKLHIE-EAWLQLFRFIA----------------------------------

>Scud_GbXb

--------------------------------------------ELTQEQKIIIKETWAIV-NVER---VGVIMFTNLFETHPDVQEVF-L-NALL-DNKKLRNHAL-RVMGFVEK-AVGRL--LQALLETCGRNHCG-AALHHIDLVGPQLLEAIKLRIS-TAWTLLMDNIA----------------------------------

>Acorn_worm_Gb7

--------------------------------------------SLTDQHRVILLDSWKVI-DIAK---VGVIMFMGLFETHPECKEVF-M-DDLR-WSSALKAHGL-RVMAVIER-VLARI--IEEHLKALAKKHVE-ANSDLVRLFGPQFIGSMKLSMQ-DAWTVLFDIII----------------------------------

>European_centipede_GbX

--------------------------------------------VLDEREIEHVIFTWKLV-NIAK---VGVITFLGLFETHPAVQSVF-L-EQLG-SSAKLEAHAL-KVMNFIQK-IIARI--VHALLRQLGKNHFH-VKREYIDLVGPQFVIAIRMAIQ-DSWLHLFASMS----------------------------------

>Artic_lamprey_GBX1

--------------------------------------------APSESQRRLVRDSWLAL-DIAR---VGVIMFVRLFETHPECKDVF-Y-QKLK-MNKQLQAHGL-RVMSFIEK-SVARL--LEQLIVEMGRKHYK-ASPKYYSFVGIEFIATVQLKVE-DAWQCLFRYIA----------------------------------

>Elephant_shark_GbX1

--------------------------------------------SLSDRQTQLVKETWRLV-DIAK---VGIIMFVRLFETHPECKDAF-F-QQLR-KSKGLRAHGL-RVMSFIEK-TVARL--LQQLALELGKSHFR-AAPKYYPYVGNEFICAVQLKVE-EAWKGLFHYLT----------------------------------

>Gar_GbX2

--------------------------------------------HLSDHHIEQIKDSWKVI-DIAK---VGIIMFVRLFETHPECKDVF-F-ERLR-ASKELRAHGLFRVMSFIEK-TVARL--LDQLALELGKSHYR-APPKYYGYVGTEFICAVQLKVE-EAWQTLFLYVT----------------------------------

>Frog_GbX

--------------------------------------------NLSEQQQQLLVESWRLI-DIAK---VGVILFVRLFETHPECKDVF-F-QALR-ANKDLRAHGL-RVLSFVEK-SVARI--LEELALELGRSHYR-APPRYYQYVGTEFISAVCLKVE-EAWKGLFAYIC----------------------------------

>Gar_GbX1

--------------------------------------------PLTESQKDLIRESWKVV-DIAR---LGIIMFIRLFETHPECKDVF-F-QELK-MSKELQAHGL-RVMSFIEK-SVARL--LEQIALELGKCHCR-APPKYYEYVGVQFISAVKLSVE-QAWESLFAYLA----------------------------------

>Elephant_shark_GbX2

--------------------------------------------TLSVEQKDLVRQSWERL-DIAR---VGIVLFIGLFETHPECKEVF-F-QQLK-TRKELQSHGL-RVMSFIEK-SVARL--LEQLIFDLGRSHQR-VDPKYYEFVGKEFIDAVKLEVE-GAWKCLFLYLT----------------------------------

>Artic_lamprey_GBX2

------------------------------------------------------------------------------------------------------------RVMSLIEK-TVARL--LEQLIFELGRKHYK-APPKYY-FVGAEFISAVKLRVD-DAWQAPS---S----------------------------------

>Acorn_worm_Gb10

--------------------------------------------KLTVEQKRLIIDSWKEL-DLER---IGMLMFMGMFGTHPQTREFF---DDPK-NTQRLREHGL-RFMSLVKK-ILVFI--LDAMLLDLGRRHQE-ADFNLIDVFGEQFILSVRLSVE-SAWAQLFKYIS----------------------------------

>Acorn_worm_Gb16

--------------------------------------------MITKEQTKILTSTWHSI-DLEK---IGLLMFMGMFDNYPETRQFF-L-EDPA-VIQKIREHGL-RFMTTARK-LVMNL--FDRILLDLGRRHHG-ADVDLIEVFGQQFIASIQLNVG-EAWEQLFKCVS----------------------------------

>Acorn_worm_Gb9

--------------------------------------------SLSKEQEKILVQTWLSI-DLER---IGLLMFTGLFEHHPEAKVMF-L-KDKE-NTALIKEHGL-RFMNVVRD-VLTLI--AECVLIDLGRRHCS-ADINLIDVFGQQFIASIQLSVE-DAWIQLFKYIA----------------------------------

>Acorn_worm_Gb8

AISNK---------------------------------------ELTKEQKDTLIQTWQNL-DLER---IGMLMFMGLFEHNPEIKEFF-A-EELR-YNEKLQEHGI-RVMGLVEK-IISSM--IDQMVVDLGKRHLG-VHIPFIDLFGRQFVFAIKLHVE-EAWTQLFKYIG----------------------------------

>Elephant_shark_Cygb

--------------------------------------------YLSDTDRDIIRQTWSRV-CCED---VGVRVLIRFFSKFPSAKQYF-S-QEMQ-HSSQLRQHAR-RVMGAINS-VVEKL--VRSVLALVGRAHAIKVDPMYFQLLSGVILEVFVYYAQ-SAWSQLMALIC----------------------------------

>Human_Cygb

--------------------------------------------ELSEAERKAVQAMWARL-NCED---VGVAILVRFFVNFPSAKQYF-S-LEME-RSPQLRKHAC-RVMGALNT-VVENL--VSSVLALVGKAHALKVEPVYFKILSGVILEVVAFDTQ-RAWAKLRGLIY----------------------------------

>Elephant_shark_GbY

--------------------------------------------GITEADKENIHFIWEKL-NPEE---NGKTIVLRMFTDYPETKMYF-Q-EEMK-KSPQIKRHGK-IVMSALNK-LIANL--LSSLLAKMAERHINKVDLHNFQIIFNIIIAILEFAIR-ETWTKLFGVIY----------------------------------

>Frog_GbY

--------------------------------------------DLTGADIENINEVWSKI-NPEE---SGRTVVISLFLTYPQTKIYF-K-QEMQ-DNAGIRAHGK-RVMGALNH-VIENL--VCSALSHLAKRHQDKVEVNNFELLFLVIISVFKLGQS-KSWEKLFSITY----------------------------------

>Chicken_GbE

--------------------------------------------SFSEAEVQSARGAWEKM-DAED---NGTAVLVRMFTEHPDTKSYF-T-EEMK-QSDQVRGHGK-RVFTAIND-MVQHL--FLGILNPLGQKHATKIDPKNFRIICDIILQLMEF-CK-ASFEKVTNEIC----------------------------------

>Turkey_GbE

--------------------------------------------SFSEAEVQSARGAWEKI-DAED---NGTAVLIRMFTEHPDTKSYF-T-EEMK-QSDQVRGHGK-RVFTAIND-MVQHL--FLGILNPLGQKHATKIDPKNFRIICDIILQLMEF-CK-TSFEKVTNEIC----------------------------------

>Platypus_GbY

--------------------------------------------QVTDVEKANIQSIWSKM-NLEK---NGIDIFTRLFREYPETKKYF---GNLQ-EDPLLRSHGR-RVMVALNR-IIQNL--VCKILNPLAEKHKISVDVENFQFMLKCVGDVCQLCIA-ESFQKLQSSLY----------------------------------

>Elephant_shark_Mb

-----------------------------------------------MCDWDLINKVWAKV-DLAG---NGQTVLLRLFEEHPETKAHF-P-GQLT-SNADVKTHGN-TVFKALGD-VVKQK----SNLQALATTHINKIPPQNFTLITNVILKVFAFEAQ-EAFSKAFKAIC----------------------------------

>Human_Mb

--------------------------------------------GLSDGEWQLVLNVWGKV-DIPG---HGQEVLIRLFKGHPETLEKF-D-DEMK-ASEDLKKHGA-TVLTALGG-ILKKK----AEIKPLAQSHATKIPVKYLEFISECIIQVLQHDAQ-GAMNKALELFR----------------------------------

>Elephant_shark_HbA

--------------------------------------------VLSKTDKALLSSSVGKI-QAQA---TGSDVLARMFASFPQTKVYF-V---TA-KGPRVQKHGL-TVMTKIIE-GIQYL--LRSFLDALSAKHAHMVDPVNFGFLGECVLSSLAL-MH-CAWDKYLCEFA----------------------------------

>Human_HbA

---------------------------------------------LSPADKTNVKAAWGKV-HAGE---YGAEALERMFLSFPTTKTYF-P-DLSH-GSAQVKGHGK-KVADALTN-AVAHV----NALSALSDLHAHRVDPVNFKLLSHCLLVTLALEVH-ASLDKFLASVS----------------------------------

>Human_HbB

--------------------------------------------HLTPEEKSAVTALWGKV-NVDE---VGGEALGRLLVVYPWTQRFF-E-DAVM-GNPKVKAHGK-KVLGAFSD-GLAHL----GTFATLSELHCDHVDPENFRLLGNVLVCVLAFEVQ-AAYQKVVAGVA----------------------------------

>Elephant_shark_HbB

--------------------------------------------QWSQAELDVIQGKWAAL-DPEK---FGGKALARMFVVYPWTKRYF-G---FK-SDPVVMEHGA-KVMGKMQV-AAK----IKEIFEYLSKRHSDHVDPENFKLLGSCMLVEMAK-IE-AINRKFVDVSI----------------------------------

>Black-legged_tick_HLb4

--------------------------------------------GLSKRDTKLIRNSWSML-QHPK---ADQLIFKALFTKHPDFMALF-Q-GVVL-SDPQFALHSS-AIIQAFGT-IIRSL--VVALIRKNATDHTTGVQPSHFEAMLNVVLEVLQLRAI-TAWEKFIEVGK----------------------------------

>Acorn_worm_Gb11

--------------------------------------------SFTDRETAILRSTWPLL-DMTR---NGGKIFLQIFAVAPHVKDLF---DMLQ-QNEIFKMHGR-RFMQSVGA-VIENI--ISILLHNLGKRHTDEVDGAYFDIYTDCMMHTWRLEVG-QVWHKLFDFII----------------------------------

>Acorn_worm_Gb14

--------------------------------------------RFSSEQISELRRTWPKL-DLTG---NGAQVFLQIFAINENIKILF---DILS-QNEVFRGHSR-RFMQAVGA-CVENL--VTTLFVGLGKKHIHGFKVDYFSTYVTSMQTVWDLHTK-QSWTQIFEFVI----------------------------------

>Acorn_worm_Gb12

--------------------------------------------LLDDRQKRIVRKTWRPL-DMTE---NGQKIFINIFESHPEIKYMF---DNLS-ANPHFRMHSS-RFMQSVGA-AIDNL--LRPLLVKLAKTHVRGFKPDYFDAFEEAMLSVWQLRVE-ESWKLLFFYIK----------------------------------

>Acorn_worm_Gb13

--------------------------------------------IFTERQRRIVRKTWRPL-DMTG---NGTKVFLHIFEMNPKVKQLF---EELL-KDLNFKGHAS-RFMQSVGA-AVDNL--LAPLLMNLGKSHNHGFELNYFDSFTGAMLHVWELRVM-EAWKLVFDYMM----------------------------------

>Brine_shrimp_HbT1-9

--------------------------------------------AFSAYDIQAVQRTWALA-DLMG---KGAMVFKQLFTDH-GYQPLF-S-TGLE-GSPELNTHAR-NVMAQLDT-LVGSL--LGQSLAQLGKDHVP-VNRVHFKDFAEHFIPLMKLEAE-SAWKKAFDVMI----------------------------------

>Body_louse_HbL1

--------------------------------------------PLTADELERVQNSWKVV-NAEE---NGMFIFKTFLLKH-NYFPYF-K-EELE-ENQAFRNHAN-NIIQALDN-VILNL--IQRELTALGKMHGK-ISEQQFQELKICILEILDF-DL-QAWSKTLNNAF----------------------------------

>Sea_squirt_Gb1

--------------------------------------------PFTDEELKLLRDSWDEV-GMKE---VGLHIFTGLLNAAPSLRTLF-YDDVMR-ENKKVVAHAT-RIANAISK-FIKFL--LEKLLTSLGESHAR-VDPESFEYVAPVILSVIGLPTL-QAWVKAYGVLR----------------------------------

>Sea_squirt_Gb2

--------------------------------------------GLTTEEIGLLRSSWNEM-GMKE---LGLLIFHRLFSDVPRIRKMF-YDEAMR-SNQKMSRHAT-RIATSIST-YLKLA--LKTFLNGLGELHAG-VEPEDFEYLAPVMLAVIGLNIL-QAWVKAYGVLR----------------------------------

>Sea_squirt_Gb3

--------------------------------------------SLTSEQVVLLRSSWQTI-GMSN---VGLAVLHRLFNDVPETLPFF-H-EVLK-SNAKVVRHAS-RVGLSIDK-IINLL--LVKYLLFLGQVHVK-IPRKYFSAMGPVLLSVISLDVM-QAWATAYGVIE----------------------------------

>Sea_squirt_Gb4

-----SGLNCWTCNVLNVCRN--------------------CC-GLKRSDIINIQDSWNTL-GYET---VGMLVLHRLFNDAPQTRYLF-S-EQMR-NNSRVVYHAN-RVARAVGR-LVDLI--FTDHLVWLGQRHAY-VAPVNFDYMGPVLLETIKLPTL-SAWAKAYGVIK----------------------------------

>Silverleaf_whitefly_HbL

--------------------------------------------GLSQTEIDLIRESWQPF-DLQE---TGITFFLAFFKRQADYQEAF---SELR-QNESFRRHAK-AVLQFIDT-AIASL--ILSMLESNGKSHGRGLTWSHYEHLEFTLLDVIDYELEKETWAKFIRSVT----------------------------------

>Bark_scorpion_HbL2

--------------------------------------------NLTSRQIELVTETWQIV-DMAN---VGVIIFXXLLTQHPELCKLF-K-DGTY-DLGGMERHAL-LVMQALEA-AIDNL--LSGILFELGCKHAR-VQEDMFDKLWDALKSGLELQVT-QAWFSVFRYIS----------------------------------

>Bark_scorpion_HbL3

--------------------------------------------NLTPQQIQLVRSTWSIV-DMVG---VGTIVFQRFLTRHPELCKMF-R-DGTY-DLEELQRHAL-LVMQALEA-AIDNL--LAGILYDLGRRHAR-VQEQMFDKLWEALRYGLELRVN-QAWFAVFKYIS----------------------------------

>Black-legged_tick_HbL5

--------------------------------------------SLTARQAELVRSTWAIV-DLAG---TGVVVFKRLLTRYPELCRLF-R-DGTY-DMEGLQRHAL-LVMQGLEA-AVENL--LADILYELGRKHAR-VHEDMFDKLWHALKFGLELRVA-QAWFIIFRFLS----------------------------------

>Water_flea_Hb1-1

--------------------------------------------LLSSHDRSIIRKTWDQA-D-GD---VPPKILFRFIVANPEYQKMF-K-NELL-GNGNFLAQAY-TILAGLNV-VIQSL--LANQINALGGAHQP-ATPIMFEQFGAITEEVLALAAR-QAWKNGMRALV----------------------------------

>Water_flea_HbB

---------------------------------------------MKEADRTLVQGTWRIA-N-GN---IAPKAFIRYFKLKPEAQKQF-A-ADLP-TNSHFLNQVY-TCLAGLNA-YMENL--QCPHLSPVFKA----VKPDDLKLFGEVMFTVMELSAR-KAWKDGLIACD----------------------------------

>Water_flea_HbG

--------------------------------------------FLNERDEATIRSTWNTA-N-GN---IGPKTFLRYFELKPEAQKMF-P-MKLP-TNEDFLAQAQ-NCVSGLNS-YVEHL--NCPFIKAKGKYH-----HEDLKLLGVTLMGVLELGTK-EAWKKGLRAMN----------------------------------

>Water_flea_HbC

---------------------SGGIGSGSYR-------------ILSENDISVLVNSWHIL-R-SD---FAPKVFMRYFKAKPEAQKLF-S-TDLP-NNHDFLNAAY-SCISSLEF-ILPHL--RCPALTDLKNK----YSVVDLKRFVPIWMAAMQMAVR-DVWKKAFSAFT----------------------------------

>Water_flea_HbE

---------------------PGGVESGG---------------SFSQKDVDVIVNTWNTL-R-GD---FAPKVFIRYFKAKPESQKMF-P-TELP-TNHDFLNSAY-TCITSLNY-LIPYL--RCPAFKHLKDK----YNAVDLKKLGSIWMTAMQMAVR-DVWKKAVMAVI----------------------------------

>Water_flea_HbF

--------------------------------------------SLSQMDVDIIVNSWNIL-R-GN---FAPKVFIRYFKAKPESQKLF-P-TDLP-TNPDFLNSAF-TCVNSLNY-LIPFL--RCPSFKQIKDN----YNEVDVKKLGSIWMMAMQMDVR-DAWKKAVMAVI----------------------------------

>Water_flea_HbK

--------------------------------------------SLSDSDINLIVSSWNFL-RLSS---FAPKVFIGYLEARTDSKKMF-P-AELA-TNVEFRSRAC-NCVASLNY-IIPHL--QCPALKNLKTKYNQ----DILKSLGIIWVKAMQLKVR-VVWKKLFSVLK----------------------------------

>Aedes_mosquito_Glob1

--------------------------------------------GITPDQRHVLVDAWKLV-DVVT---HGTNIFLKFFEKNPEYLGYF-D-KELK-DNRSLHAHAL-NVMNFFGA-IIDYL--YKSSLSKMVINHKR-VSKPDVAIVCAIIKDYCLL-LE-DAFTALLDSVA----------------------------------

>Culex_mosquito_Glob1a

--------------------------------------------GLTNHQKAALVGAWSLV-DMVS---HGVNVFIRLFEEHPKYLEYF-D-EELR-ENKSLHAHAL-NVMHLIGA-LIDYL--FKCSLSKMMKNHKK-VNKEDVTIVCGIIMEYCLLRLE-EAFSSFMKSIA----------------------------------

>Culex_mosquito_Glob1b

--------------------------------------------GLNNHQKVALIGAWSLV-DIIS---HGRNIFVRFFEEHPQYLNYF-D-SEIG-ENKSLHAHAL-NVMHFIGT-LIDYL--FKCSLSKMMKNHKK-VHKKDVTIVCEVIMKYCLLHLE-AAFKSLMRSIA----------------------------------

>Anopheles_mosquito_Glob1

--------------------------------------------GLTKSQKVALIAAWSIV-DLVT---HGRNIFVMFFEEYPQYLDYF-D-GELG-ENRSLHAHAL-NVMNFIGT-LIDYL--LKCSLGKLVRNHRK-VTKEDVAAVGGVIMRYSLLHLE-EAFGAFLGTVA----------------------------------

>Aedes_mosquito_Glob2

--------------------------------------------GLTGKQKITLLSAWGLL-ESSL---HGRNMMFLLFREHPRYLPYF-D-SNLA-DNKSFHLHAV-NVMGAIGT-LIECL--FRKKLFHLVEVHKA-VTPLDVQLFSEIITDYLVLQLA-DALGKLFDQFA----------------------------------

>Culex_mosquito_Glob2

--------------------------------------------GLTGKQKITLLSAWGLI-DLDL---HGRNIMLLIFREHPHFIPYF-D-TSLS-ENRALQAHSL-NLIMALGA-LIEYL--FECTLAKLVKNHKT-VTSQDVKMFGEVILMYFALQLP-TAFNRLIEQIA----------------------------------

>Anopheles_mosquito_Glob2

--------------------------------------------GLTASEKITLFSAWGLI-DLDV---HGRNVLLLLFHKHPRYIAYF-D-QSLV-DNKSLYDQAI-HVFKAVGA-LIEYF--FDATLRKITRRHKD-VYTEDILTIGEVLLNYLELQLP-DAFWKLFQTIA----------------------------------

>Sea_louse_HbL

--------------------------------------------ILTSNELSLISESWKLV-DLEH---HGLSFFLKLFEEYPTYQEKF------Q-DERKIQRHGA-IVLKSVGK-LVAFL--LVDAIKRLATNHSR-VLREQFYPACRILLEYLALHGA-LAWKRFLGTFV----------------------------------

>Salmon_louse_HbL

--------------------------------------------LFCEHEVKLISESWKLM-DLDN---HGLNFFLKLFKEYPVYEEKF------G-DRKKLKRHGG-IVMKALGK-LVGFL--IVNTIKGIANSHSK-VLVQQFTPICDILLKYLGFQGT-ATWKKFLDIFV----------------------------------

>Itch_mite_HbL3

--------------------------------------------ELTSKEIDFVRNTWSLL-DIARFKFLGGELFVRFFTKYPDYQRQF-K-RDIR-FNKKLMAHGT-YVMYTIGM-LVDNL--MEQMLKRLARNHYR-ISLIAFDRLRNTFLEHLALKVS-IAWSKAFNYLL-----MHLAARNDLV-----------------EE

>Yellow_lupin_LegHb

--------------------------------------------ALTESQAALVKSSWEEF-NIPK---HTHRFFILVLEIAPAAKDLF-S-EVPQ-NNPELQAHAG-KVFKLVYE-AAIQL--TDATLKNLGSVHVS-VADAHFPVVKEAILKTIKVKLN-SAWTIAYDELA----------------------------------

>Alfalfa_LegHb

--------------------------------------------SFTDKQEALVNSSWEAF-NLPR---YSVFFYTVVLEKAPAAKGLF-S-AEVQ-DSPQLQAHAE-KVFGLVRD-SAVQL--VVLGDATLGAIHVR-VVDPHFVVVKEALLKTIKAKLN-TAWEVAYDALA----------------------------------

>Mussel_shrimp_HbL1

--------------------------------------------GLTPHQKAAMRESWSKL-DKRT---NGTDFFAQLITRYPEYQKFF-R-DQFR-SSRKLQAHSM-QFMHGVSN-LLDNV--FGELMDKMALRHRPKLGKDDFDKATNLLIEVLLSKLK-EAWSKAFEVIN----------------------------------

>Mussel_shrimp_HbL2

--------------------------------------------GLTPHQKAGLRESWLKL-DMRS---NGTTFFAQLITRYPEYQKFF-R-DQYT-NSRKLQAHSM-QFMRGVTN-LLDNL--LGELMDKMALRHKPKLGKDDFNKATNLLIEILLPQLK-DAWTKTFELVN----------------------------------

>Mussel_shrimp_HbL3

--------------------------------------------GLTPRDKDILRKSWGVA-DFRG---NGTEFFYQFFKRYPKYIDTF-K-DEAK-ANKRLQVHAM-GFMHGVAN-VLENL--LIELLERLGRRHAP-LKKEDMENATDLFSELLLLIVA-STWEKAFKVIN----------------------------------

>Western_predatory_mite_HbL1

--------------------------------------------KLSTDEVDAVQAAWQVV-DQRS---IGQQVMMTLFSENPEYIHKF-K-DQLP-YHTALRAHSL-SILYVIHS-LIDSM--MRELIRKVALTHKP-VNRDNFQRFEDAFILVLKGRTE-EAFHKCILYFT----------------------------------

>Amphioxus_Gb10

--------------------------------------------SLSAADKKAVSDSWAKM-SFQD---AGERVFLKLLKK-DSTKAMF-K-ERLA-GNAALREHGG-KVVQALDD-FIKGL--GHETVRNVGRIHKA-MTNDNINLMKPILLELL-V--K-AAWDKLWNLFM----------------------------------

>Amphioxus_Gb15

--------------------------------------------GLTSEDKSAVLDSWAKM-TFQD---AGEKVFLLLLKT-DSTKALF-P-DQLA-GHPDVRDHGG-KVMQVLDD-FIKGL--GDGAVQKVGLLHKG-VSHDNINLMKPVLMTLL-L--A-GAWENLWARFM----------------------------------

>Amphioxus_Gb7

--------------------------------------------SLSAADKKLVQESWDKV-SFAD---AGERVFLKLFQRNESTKAHF-K-DQLA-GQAVVRDHGE-KVCKVLDD-FIKGL--GDEAVKKVGRMHKG-MSNEQIDQMKGAIIEVL-A-YK-GAWGKLWDRFM----------------------------------

>Amphioxus_Gb11

--------------------------------------------ALSAAELATVKQAWAKL-SFED---AGEKVFLALLKD-PNIKANF-K-ASLP-GNTDMRAHGK-KVCTVLDK-FIKGD-----AAKSTGTMHKG-MSNDQIGAMRGALVAVL-A--V-PAWNKLFDHFM----------------------------------

>Midge_Hb2B

--------------------------------------------PLSADEASLVRGSWAQV-S-------EVDILYYIFKANPDIMAKF-P-ETLK-GTGQFATHAG-RIVGFVSE-IVALM-SMETLIKDMAANHKA-IPKAQFNEFRASLVSYLQV-LG-AAWTQGLDNVF----------------------------------

>Midge_Hb6

--------------------------------------------VLTTEQADLVKKTWSTV-N-------EVDILYAVFKAYPDIMAKF-P-DSIK-DSAAFATHAT-RIVSFLSE-VISLA-DIQNLAKELATSHKP-VSKDQFTEFRTALFTYLKI-TE-TAWTLALDTTY----------------------------------

>Midge_HbE

--------------------------------------------ALSGDQIGLVQSTYGKV-D-------SVGILYAVFKADPTIQAAF-P-DAIK-GGAEFSTHAG-RIVGFLGG-VIDDL--IGKHVDALVATHKP-VTHAQFNNFRAAFIAYLKV-VE-AAWGATFDAFF----------------------------------

>Midge_Hb3A

--------------------------------------------SMTDAQVAAVKGDWEKI-S-------GVEILYFFLNKFPGNFPMF-K-AAAK-GTAEFKDQAD-KIIAFLQG-VIEKL--AKALLNQLGTSHKA-ITKDQFDQFRQALTELLGL----GAWNATVDLMF----------------------------------

>Water_flea_Hb1-2

--------------------------------------------KLTPHQIHDVQRSWENI-NRNS---LVSAIFVKLFKETPRVQKHF-A-DSLP-GNADYEKQVA-LVADRLDT-IISAM--LLGNINYMRYTHQPAIPRQTFEDFARLLIDGLT-GDM-DSWKGVLTIFV----------------------------------

>Backswimmer_HbL

--------------------------------------------SMTDREVEVINQSWNQI-QELV---VGLQMFKLLFQRYPQYERLF-T-KSLY-EGDRFQHHVV-NIMSSINK-VIEQL--APRTLQEMGVRHKK-VHRKHFESFVPFVVDAMVRMVA-SAWTKLMDAIA----------------------------------

>Glassy-winged_sharpshooter_HbL3

--------------------------------------------SLTDRDLRLGRATWFKN-ATPD---FGMVIFKELFRQYPEVESYF-L-GSIF-DSRTFRSHMT-RVVPKLKE-VFEAL--LNEVMTKLGLYHAK-VSGHLVENMLSVILDALKMKEE-TAVRTCLKSAFA-Y-------------------------------

>Brown_planthopper_HbL

--------------------------------------------EPSPKDLANVKEAWCEI-NKGC---YAKAIFTEVFKKYPDYAQLF-A-TDIL-KNEKFSEHLK-NVMDEMGN-VIKKM--AKSMASDIGKKHVK-VKPKHFENTEKIFIDVLKMKGA-KSLENVIKSVF----------------------------------

>Glassy-winged_sharpshooter_HbL2

--------------------------------------------AITDRDKELAREAWVQV-NYVL---ISKNLFVDWFTQYPEHVNFF-K-DDIF-TSPKFARHMA-SLLPNLGI-IIRNL--FRSHILKVAWSHVEDLNSDHLDILKGLILRTLKLGHE-VALFKIITAAFK---------------------------------

>Mayfly_HbL3

--------------------------------------------DLHSEEISGVEDIIKNV-DLAT---HGPALFLELFKLEPGYQKLF-T-DSLK-DNAALKAHSV-NILGKFAGCVLENI--MQGCFKGQAETHKK-VGYDHAKVLSKAIQNYLSLGVK-TGWNKVLYVMT----------------------------------

>Mediterranean_fruit_fly_Glob2

--------------------------------------------GLTITERRSLQNGWSII-KQRR---AALTIYVNLFTEHENLYEVF---DGVL-NIEFASQHQK-EVLTVFQM-IIEQV--VKTMLKELALRHEA-VTNTQWQLYTNEVRKYFLLAFV-HALDKLMNFVCN---------------------------------

>Tsetse_fly_Glob2

--------------------------------------------GFTPLEIVALQNIWRLF-RFKY---HSMQIFLAFFNQNHKLIERF-R------QLSHLCQHSE-KMLLLYEN-VIDKL--FHGVMAEVTVKHQR-VKYEEIILKTEHVRRYILFQLV-SALAKLSEHFNDR--------------------------------

>Australian_sheep_blowfly_Glob2

--------------------------------------------GFTSTEIVALQNGWHLI-RLYY---HSTKIFKDFFSEHYLLLERF-R------NLSNLHQHPG-QLMNIYGR-LIESL--INMLLSDVGQRHKL-VTYDDVKLLTNHIRLYVILIFV-NGLTKLSELINER--------------------------------

>House_fly_Glob2

--------------------------------------------GFTATEIASLRNGWRHF-RFGY---HSKQIFMKFYQEHEQMLEKF-R------NMQQLHRHPQ-ELLQVYGN-LIEQL--MHVLMTAISQRHRM-VTGYEIKLQTDHITLYILLIFV-SGLEKLSRLINAC--------------------------------

>Fruit_fly_Glob2

--------------------------------------------EFTMVEKASLRNAWRLI-FQRR---FGKENFYSFLTRNEDLINFF-R------NLSKLHGHAM-AMMKLMSK-LVQTL--FRLALDENLPTHLK-IDPDYMRMLATALKSYILIHLS-NGLARLVEIVGE---------------------------------

>Fruit_fly_Glob3

--------------------------------------------GFTLSERLALRQAWNLV-FERR---YGQDVFYSFLNDYYWGIKKF-R------NVKALHSHAL-RFINFFGL-LIEE---FQLMINDNNHTHNR-VGSVNIGHLAQALVDYVLFVLE-QGLSKLVEKFQND--------------------------------

>Itch_mite_HbL2

--------------------------------------------EFEREEIEVLREQWDRI-HQEC---FGMKLFQRLLQLHPEYRPLF---EEIQ-NTQRLKAHGI-NVVYMLNM-LFDNF--IDELIFKLVKLHMM-IDQIWLDDIIEPFELVLEFK---EVLRKAFIFIK---NV-----------------------------

>Body_louse_HbL2

--------------------------------------------------MNVVLNDWPKI-NYKK---IFIDSFINYFAENPNYKLLF-P-DDLP-FNHCFRLHCF-AVYKAINF-LMSNL--DSKILPVIGKTHFD-ITLEMMNLYKHSIVYSCNLN---LSWQTVFDHIFD---------------------------------

>Body_louse_HbL3

--------------------------------------------ENLSLAVKIVTPTWESI-DFDW---YCTKIEETFFQNDTTKKELF-P-EELTDVNKRLFKHSS-AVLNFMEC-IVQFM--TKPVLFVLGRNHYT-VNEKLFLEMKDAICSVIKIENA-KAWDTILQYIL----------------------------------

>Seed_shrimp_(Puriana)_HbL2

---------------------------------------------LTDDQKCLIHQTWCCS-KRPT---VGVAYLLTVFSTYPKTQCFM---ENMC-TNPELRTVAW-QIMQKMSN-LIESL--FEDLVQEMTELVCR-IYTPDFRRLIDLFLCIMEEKAV-MAWRTFRDIII----------------------------------
