# Supplementary material for: The Globin Gene Family in Arthropods: Evolution and Functional Diversity
Source: Front Genet. 2020 Aug 13;11:858. doi: 10.3389/fgene.2020.00858 (PMC7457136; doi:10.3389/fgene.2020.00858)
Supplement: TABLE S5 — Results of RNA-Seq analysis. [file Table_5.docx]

| ***accession number*** | ***sequencing technique*** | ***tissue*** | ***reads before editing*** | ***reads after editing*** | ***mapped reads (%)*** | ***RPKM***  ***GbXL*** |
| --- | --- | --- | --- | --- | --- | --- |
| SRX025530 | 454 GS FLX | embryo | 1.136.885 | 1.104.287 | 84,44% | 5,85 |
| SRX025533 | 454 GS FLX | larvae | 1.088.688 | 1.050.014 | 91,15% | 0 |
| SRX025527 | 454 GS FLX | adult | 418.025 | 398.065 | 42,13% | 0 |
| SRX025528 | 454 GS FLX | abdomen | 622.279 | 613.795 | 68,88% | 0 |
| SRX025529 | 454 GS FLX | antennae | 1.135.230 | 1.116.158 | 88,23% | 3,88 |
| SRX025532 | 454 GS FLX | testes | 1.317.973 | 1.278.705 | 88,99% | 1,92 |
| SRX016658 | 454 GS FLX | queen ovary | 1.357.383 | 1.352.855 | 80,21% | 1 |
| SRX025531 | 454 GS FLX | brain& ovary | 1.556.239 | 1.374.997 | 70,62% | 18,53 |
| SRX030492 | Illumina Genome Analyzer II | forager brain 1 | 15.738.118 | 14.393.600 | 74,09% | 57,67 |
| SRX030493 | Illumina Genome Analyzer II | forager brain 2 | 14.679.014 | 13.475.546 | 74,11% | 46,63 |
| SRX030494 | Illumina Genome Analyzer II | forager brain 3 | 13.917.711 | 12.744.741 | 73,12% | 34,46 |
| SRX030495 | Illumina Genome Analyzer II | forager brain 4 | 25.440.947 | 23.246.199 | 73,99% | 64,82 |
| SRX030496 | Illumina Genome Analyzer II | forager brain 5 | 28.566.976 | 26.043.856 | 73,88% | 56,7 |
| SRX030487 | Illumina Genome Analyzer II | nurse brain 1 | 9.912.179 | 9.075.465 | 74,30% | 48,84 |
| SRX030488 | Illumina Genome Analyzer II | nurse brain 2 | 30.680.546 | 27.869.494 | 75,25% | 16,93 |
| SRX030489 | Illumina Genome Analyzer II | nurse brain 3 | 16.911.515 | 15.452.828 | 74,39% | 42,41 |
| SRX030490 | Illumina Genome Analyzer II | nurse brain 4 | 14.514.311 | 13.292.947 | 74,88% | 39,33 |
| SRX030491 | Illumina Genome Analyzer II | nurse brain 5 | 11.451.917 | 10.487.496 | 68,87% | 52,39 |

Supplementary Table S5. Results of RNA-Seq analyses
