# Supplementary material for: The Globin Gene Family in Arthropods: Evolution and Functional Diversity
Source: Front Genet. 2020 Aug 13;11:858. doi: 10.3389/fgene.2020.00858 (PMC7457136; doi:10.3389/fgene.2020.00858)
Supplement: DATA SHEET S1 — Full version of Figure 1 phylogenetic tree including species names. [file Data_Sheet_1.PDF]

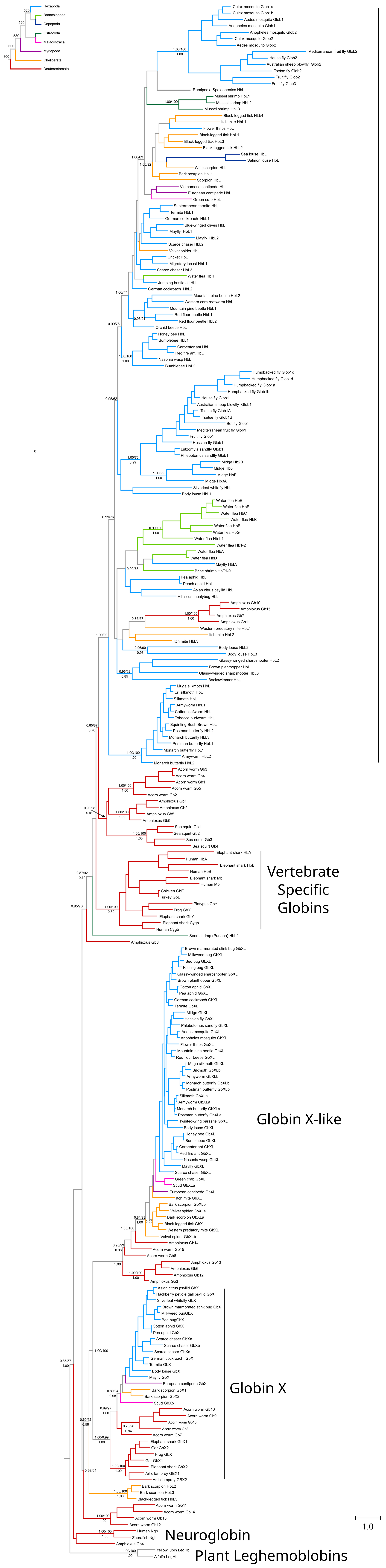
Hexapoda
Branchiopoda
Copepoda
Ostracoda
Malacostraca
Myriapoda
Chelicerata
Deuterostomata
Arthropod HbLs
Vertebrate Specific Globins
Globin X-like
Globin X
Neuroglobin
Plant Leghemoblobins
1.0
