# Supplementary material for: The Globin Gene Family in Arthropods: Evolution and Functional Diversity
Source: Front Genet. 2020 Aug 13;11:858. doi: 10.3389/fgene.2020.00858 (PMC7457136; doi:10.3389/fgene.2020.00858)
Supplement: DATA SHEET S2 — Analysis of globin domains in arthropod Adgb. [file Data_Sheet_2.PDF]

A

class	order	species	Fugue			NCBI CDD-search
			best globin hit (PDB code)*	Z-score**	confidence level**	e-value globin domain
Mammalia	Rodentia	*Mus musculus*	glob	7.81	CERTAIN	6.79e-07
Insecta	Lepidoptera	*Danaus plexippus*	no globin hit	-	-	no globin domain identified
		Spodoptera frugiperda	no globin hit	-	-	no globin domain identified
		Bombyx mori	no globin hit	-	-	no globin domain identified
	Coleoptera	*Pogonus chalceus*	hs3mvca	4.35	LIKELY	no globin domain identified
		Dendroctonus ponderosa (partial sequence)	no globin hit	-	-	no globin domain identified
	Hymenoptera	*Apis mellifera*	hs1h97a	2.69	GUESS	no globin domain identified
		Apis florea	hs3mvca	2.52	GUESS	no globin domain identified
		Bombus terrestris	hs1h97a	2.72	GUESS	no globin domain identified
		Megachile rotundata	hs3mvca	2.83	GUESS	no globin domain identified
		Camponotus floridanus	hs3mvca	2.74	GUESS	no globin domain identified
		Solenopsis invicta	Bac_globin	2.77	GUESS	no globin domain identified
		Acromyrmex echinatior	no globin hit	-	-	no globin domain identified
	Psocoptera	*Pediculus humanus*	hs4bjaa	4.00	MARGINAL	no globin domain identified
	Hemiptera	*Acyrthosiphon pisum*	no globin hit	-	-	no globin domain identified
	Polyneoptera	*Blatella germanica*	no globin hit	-	-	no globin domain identified

*PDB code	**Z-score
glob = average profile of 41 globins	Recommended cutoff : ZSCORE >= 6.0 (CERTAIN 99% confidence)
hs3mvca = Caenorhabditis elegans globin 6	Other cutoff : ZSCORE >= 4.0 (LIKELY 95% confidence)
hs1h97a = Paramphistomum epiclitum globin 3	Other cutoff : ZSCORE >= 3.5 (MARGINAL 90% confidence)
Bac_globin = Protozoan/cyanobacterial globin	Other cutoff : ZSCORE >= 2.0 (GUESS 50% confidence)
hs4bjaa = Caenorhabditis elegans globin 12	Other cutoff : ZSCORE < 2.0 (UNCERTAIN)

B

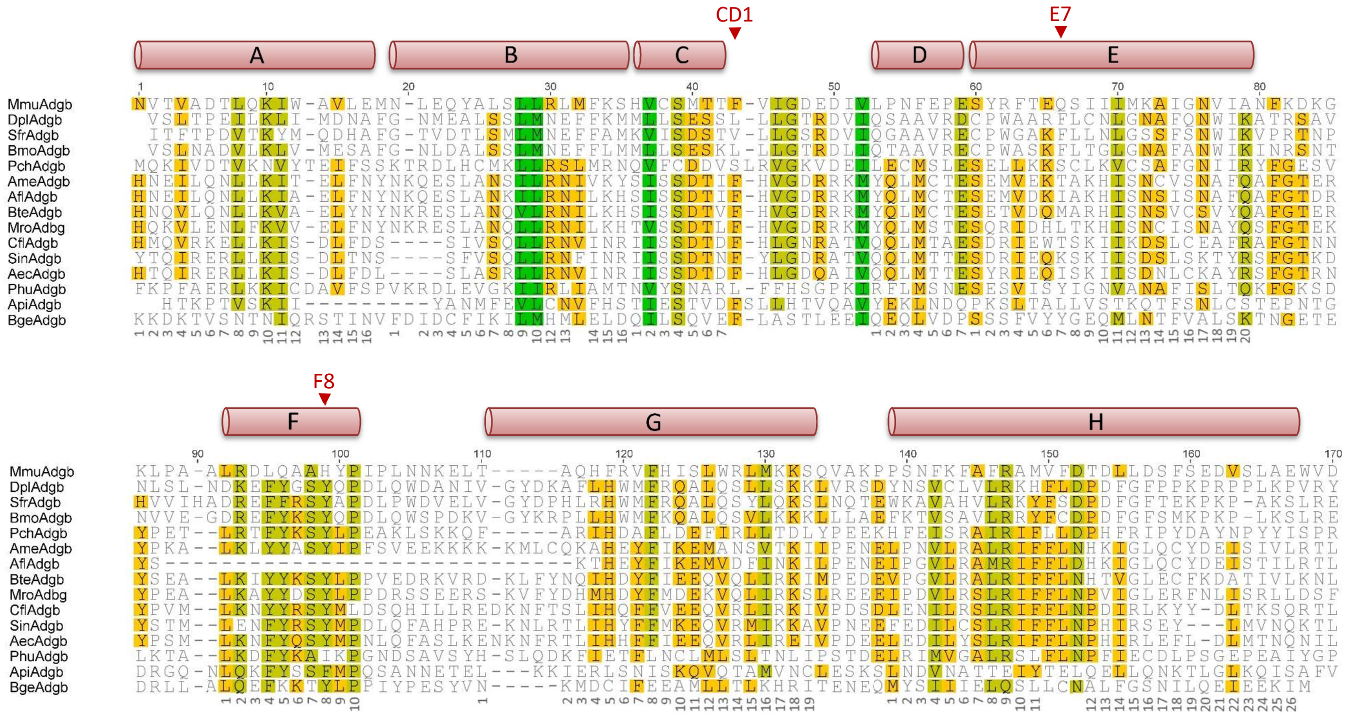

Supplementary Figure S2. Analysis of globin domains in arthropod Adgb. (A) Confirmation of globin domain identity using CDD) (Marchler-Bauer et al. 2015) and FUGUE (Shi et al. 2001). (B) Amino acid alignment of the rearranged globin domains of androglobin (Adgb) from mouse and hexapods. Residues with high similarity are colored (dark green: 100% similar, light green: 80 to 100% similar, yellow: 60 to 80% similar; Blosum62). Numbering of residues is taken from Hoogewijs et al. 2012.
